# Supplementary material for: Emodin, a rising star in the treatment of glycolipid metabolism disorders: a preclinical systematic review and meta-analysis
Source: PeerJ. 2025 May 15;13:e19221. doi: 10.7717/peerj.19221 (PMC12085882; doi:10.7717/peerj.19221)
Supplement: Supplemental Information 2 [file peerj-13-19221-s002.docx]

Supplementary Files S2

| PubMed search strategy |
| --- |
| #1. Diabetes Mellitus, Type 2 [mh]  #2. Glucose Metabolism Disorders[mh]  #3. Insulin Resistance [mh]  #4. Glucose Intolerance [mh]  #5. Type 2 Diabetes [mh]  #7. T2D [mh]  #8. T2DM [mh]  #9. #1 OR #2 OR#3 OR #4 OR #5 OR #6 OR #7 OR #8 OR  #10. emodin [mh]  #11. frangula emodin [mh]  #12.Frangulic Acid [mh]  #13. Emodin, Frangula [mh]  #14. Rheum Emodin [mh]  #15.Archin [mh]  #16. #10 OR #11 OR #12 OR #13 OR #14 OR #15 OR  #17. #9 AND #16  #18. Animals [tiab] not humans [tiab]  #19. Rat. [tiab]  #20. Mice [tiab]  #21. Experiment [tiab]  #22. #18 OR #19 OR #20 OR #21  #23. #17 AND #22 |
