# Supplementary material for: Emodin, a rising star in the treatment of glycolipid metabolism disorders: a preclinical systematic review and meta-analysis
Source: PeerJ. 2025 May 15;13:e19221. doi: 10.7717/peerj.19221 (PMC12085882; doi:10.7717/peerj.19221)
Supplement: Supplemental Information 3 [file peerj-13-19221-s003.docx]

**Supplementary Files S3-- Steps for the GetData operation**

1. Import Image

1.1 Click File > Open in the menu bar, and select the image file from which you need to extract data (supporting formats such as BMP, JPEG, PNG, etc.).

1.2 After the image is loaded, use the Zoom In and Zoom Out tools in the toolbar to adjust the image to ensure that the chart is clearly visible.

2. Set up the coordinate system

2.1 Click the "Set the scale" button in the toolbar.

2.2 Click on the starting and ending points of the X-axis and Y-axis in the image, and enter the corresponding coordinate values respectively.

- For example, if the X-axis range is from 0 to 10 and the Y-axis range is from 0 to 100, enter these values respectively.

2.3 After confirming that the coordinate settings are correct, click OK.

3. Extracting Data Points

3.1 Click the "Digitize area" button in the toolbar to select the area from which you want to extract data.

3.2 Use either the "Point capture mode" or "Line capture mode" to extract data points:

- Point capture mode: Manually click on the points in the chart, and the software will automatically record the coordinates.

- Line capture mode: Click on multiple points along the curve, and the software will automatically fit the curve and extract the data.

3.3 The extracted data points will be displayed in the data list on the right side.

4. Adjust and Verify Data

4.1 If the extracted data points are inaccurate, you can use the Edit points tool to manually adjust the positions of the points.

4.2 Check whether the values in the data list are consistent with the chart to ensure the accuracy of the extraction.

5. Export Data

5.1 Click on File > Export Data in the menu bar, and select the export format (such as TXT, CSV, Excel, etc.).

5.2 Specify the save path and file name, and click Save to complete the data export.
