# Supplementary material for: Emodin, a rising star in the treatment of glycolipid metabolism disorders: a preclinical systematic review and meta-analysis
Source: PeerJ. 2025 May 15;13:e19221. doi: 10.7717/peerj.19221 (PMC12085882; doi:10.7717/peerj.19221)
Supplement: Supplemental Information 4 [file peerj-13-19221-s004.docx]

Supplementary Files S4 Analysis risk of bias

| **Study** | **A** | **B** | **C** | **D** | **E** | **F** | **G** | **H** | **I** | **J** | **Total** |
| --- | --- | --- | --- | --- | --- | --- | --- | --- | --- | --- | --- |
| Xiaoyan Zhao 2009(Zhao et al., 2009) | - | ？ | ？ | ？ | - | - | - | ＋ | ？ | ＋ | 2 |
| Jianfeng Xue 2010(Xue et al., 2010) | - | ？ | ？ | ？ | - | - | - | ＋ | ？ | ＋ | 2 |
| Yuejing Wang 2012(Wang et al., 2012) | - | ？ | ？ | ？ | - | - | - | ＋ | ？ | ＋ | 2 |
| Lichang Zhou 2012 | - | ？ | ？ | ？ | - | - | - | ＋ | ？ | ＋ | 2 |
| Bing Song 2012(Song & Liu, 2012) | - | ＋ | ？ | ？ | - | - | - | ＋ | ？ | ＋ | 3 |
| Qing Xiang 2014(Xiang et al., 2014) | - | ？ | ？ | ？ | - | - | - | ＋ | ？ | ＋ | 2 |
| Aditya Arvindekar 2015(Arvindekar et al., 2015) | - | ？ | ？ | ？ | - | - | - | - | ？ | ＋ | 1 |
| Yuping Song 2017(Song et al., 2017) | - | ＋ | ？ | ？ | - | - | - | ＋ | ？ | ＋ | 3 |
| Sameer Abu Eid 2017(Abu et al., 2017) | - | ？ | ？ | ？ | - | - | - | ＋ | ？ | ＋ | 2 |
| Dan Xiao 2018(Xiao et al., 2019) | - | ？ | ？ | ＋ | - | - | - | ＋ | ？ | ＋ | 3 |
| Song Bing 2018(Xuezheng et al., 2018) | - | - | ？ | ＋ | - | - | - | ＋ | ？ | ＋ | 3 |
| Fei Gao 2019(Gao, 2019) | - | ？ | ？ | ＋ | - | - | - | ＋ | ？ | ＋ | 3 |
| ***Note:*** A: Sequence generation; B: Baseline characteristics; C: Allocation concealment; D: Random housing; E: Blinding (performance bias); F: Random outcome assessment; G: Blinding (detection bias); H: Complete outcome data; I: Selective outcome reporting; J: Other sources of bias; - : Not mentioned; ? : Uncertain; + : Clear statement | | | | | | | | | | | |
