# Supplementary figures and images for: Emodin, a rising star in the treatment of glycolipid metabolism disorders: a preclinical systematic review and meta-analysis
Source: PeerJ. 2025 May 15;13:e19221. doi: 10.7717/peerj.19221 (PMC12085882; doi:10.7717/peerj.19221)

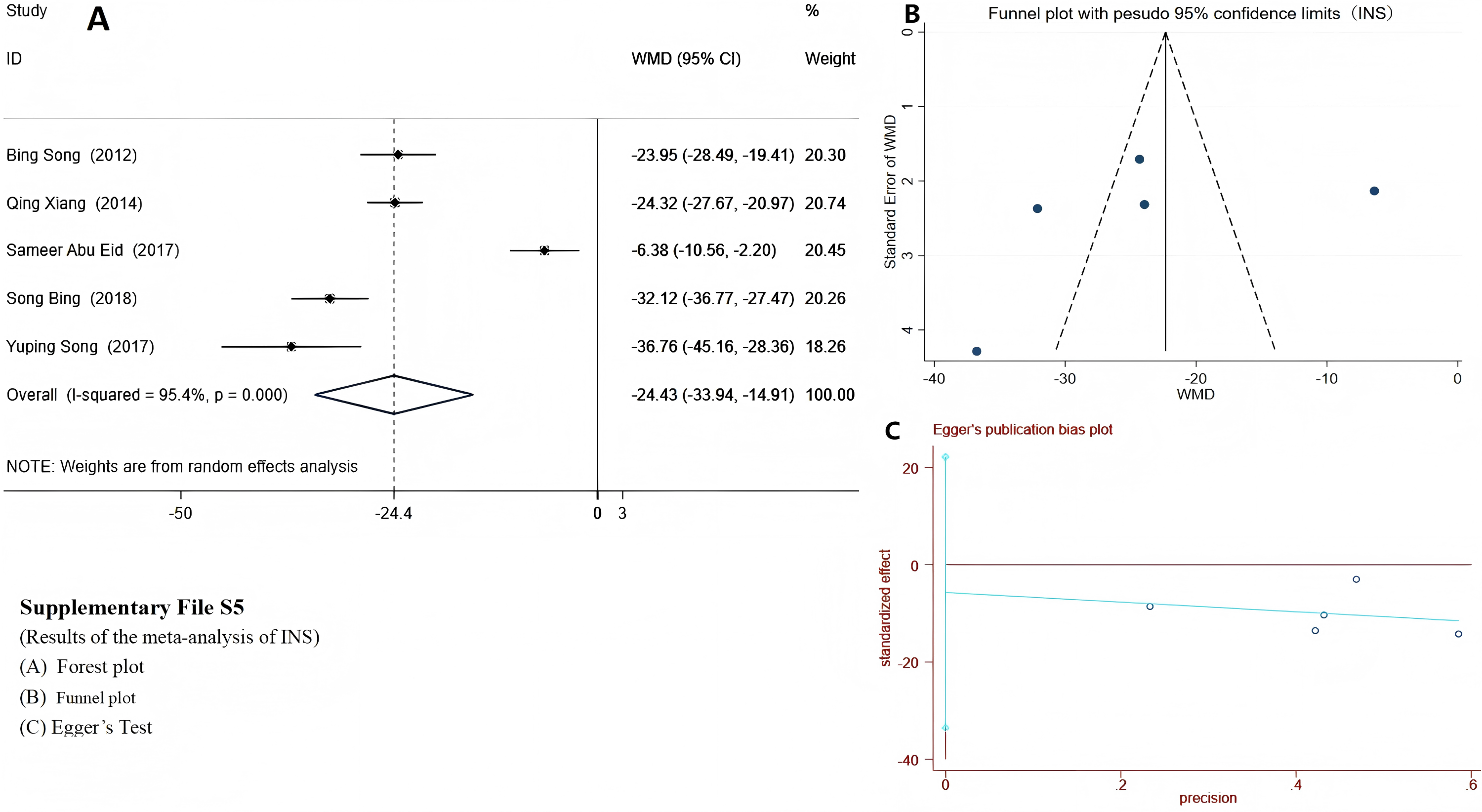

Supplement: Supplemental Information 5 — Group treated with emodin; control: DM animal group. [file peerj-13-19221-s005.png]

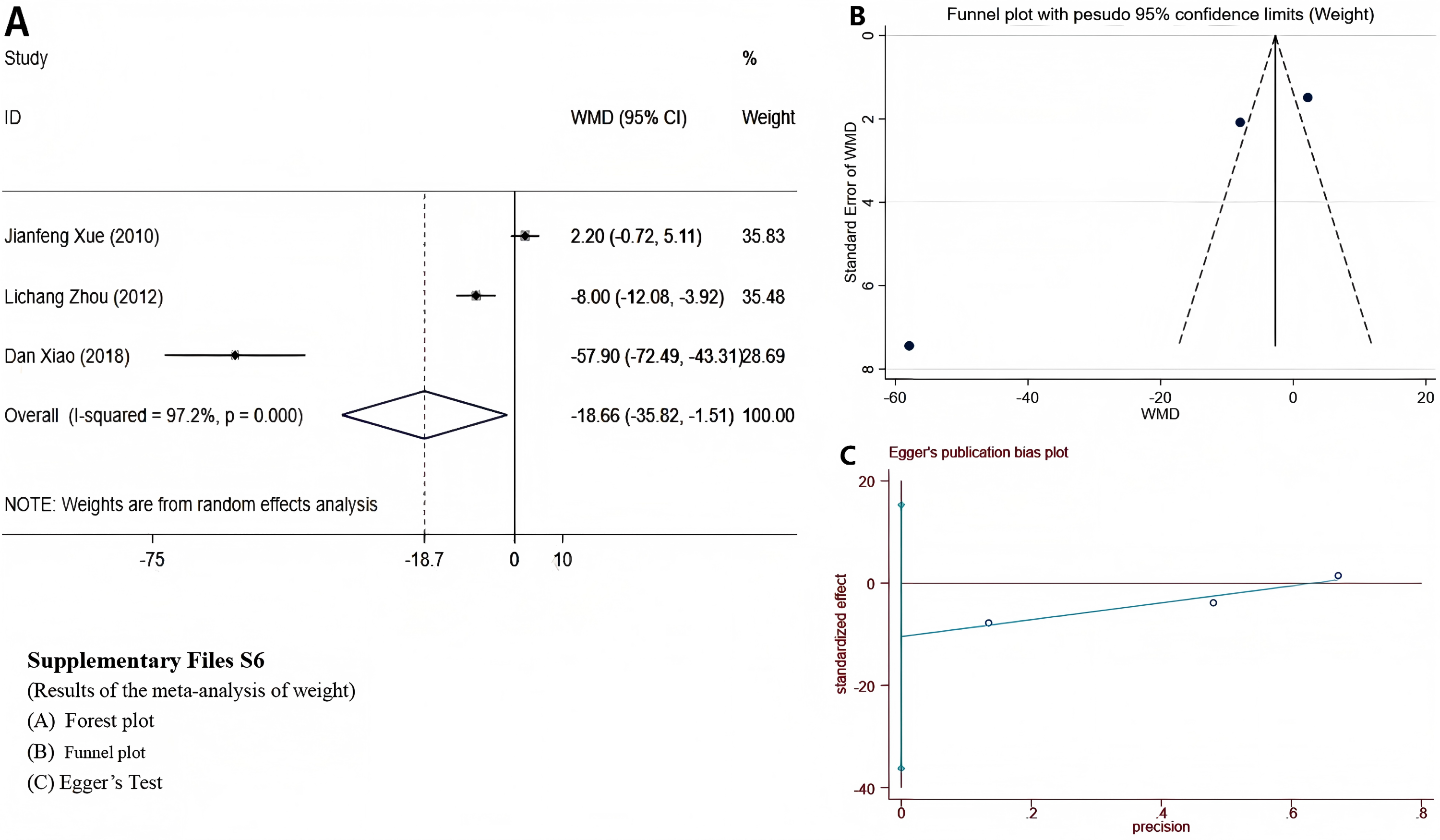

Supplement: Supplemental Information 6 — Experimental: group treated with emodin; control: DM animal group. [file peerj-13-19221-s006.png]

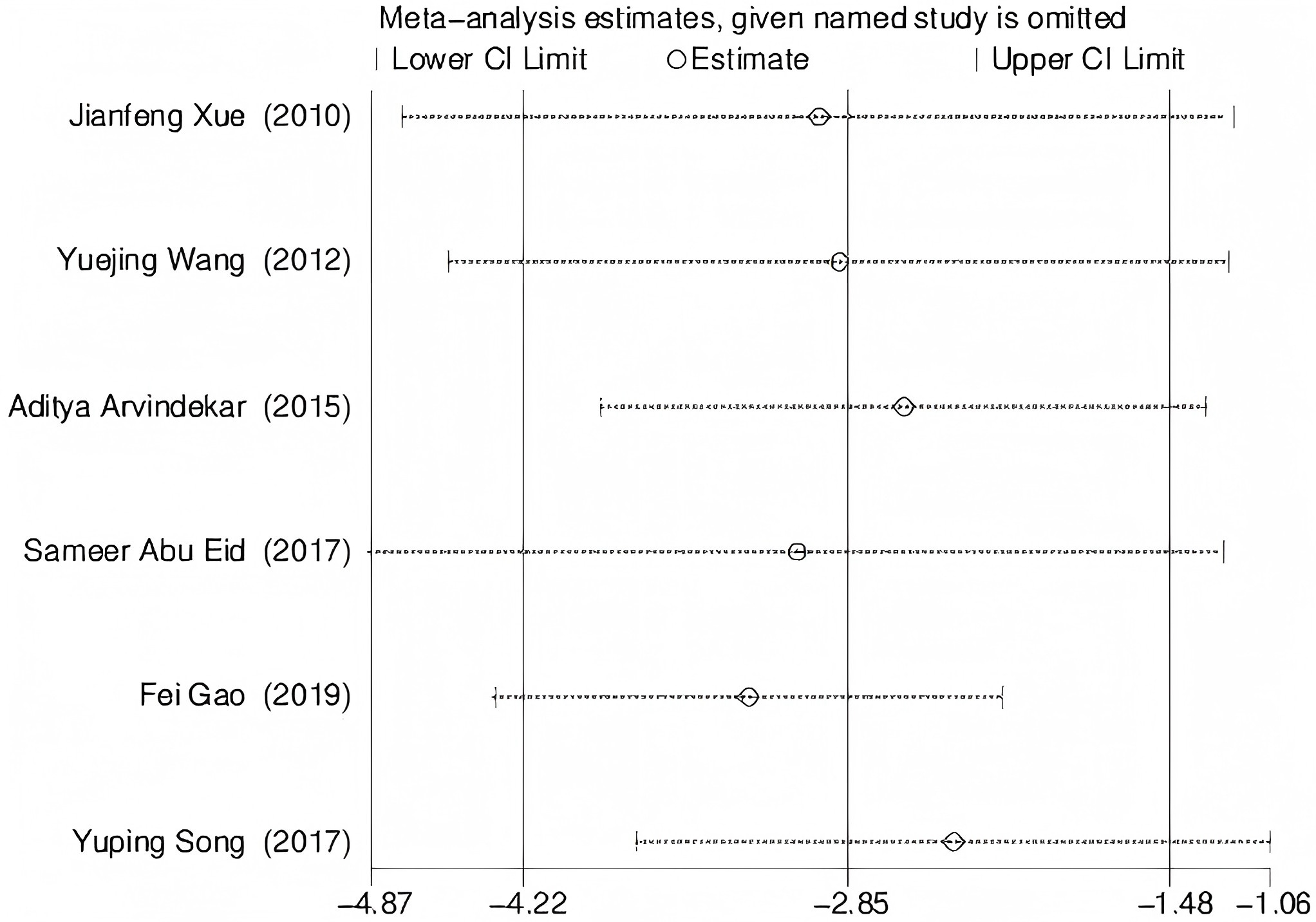

Supplement: Supplemental Information 9 [file peerj-13-19221-s009.png]

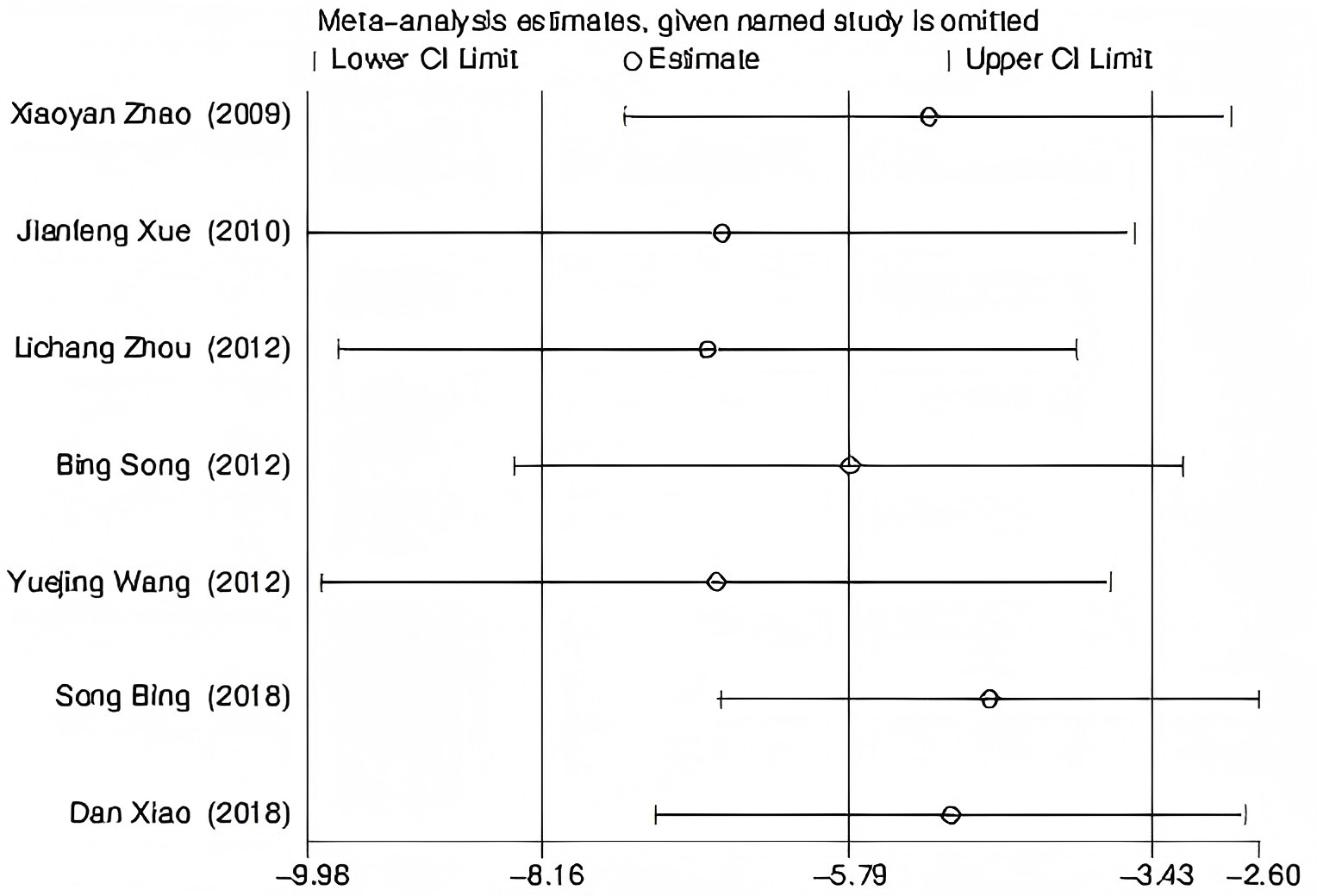

Supplement: Supplemental Information 10 [file peerj-13-19221-s010.png]

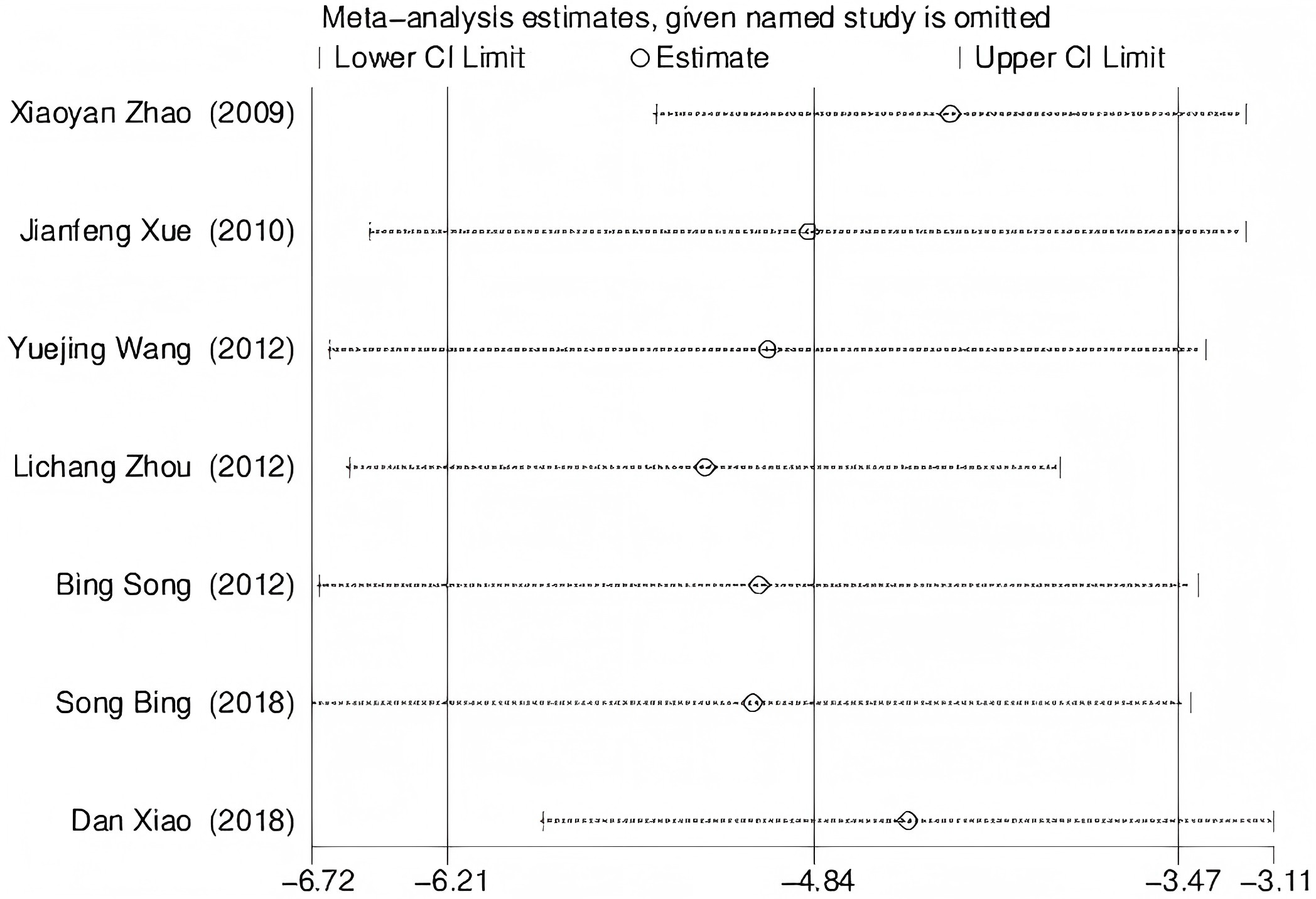

Supplement: Supplemental Information 11 [file peerj-13-19221-s011.png]

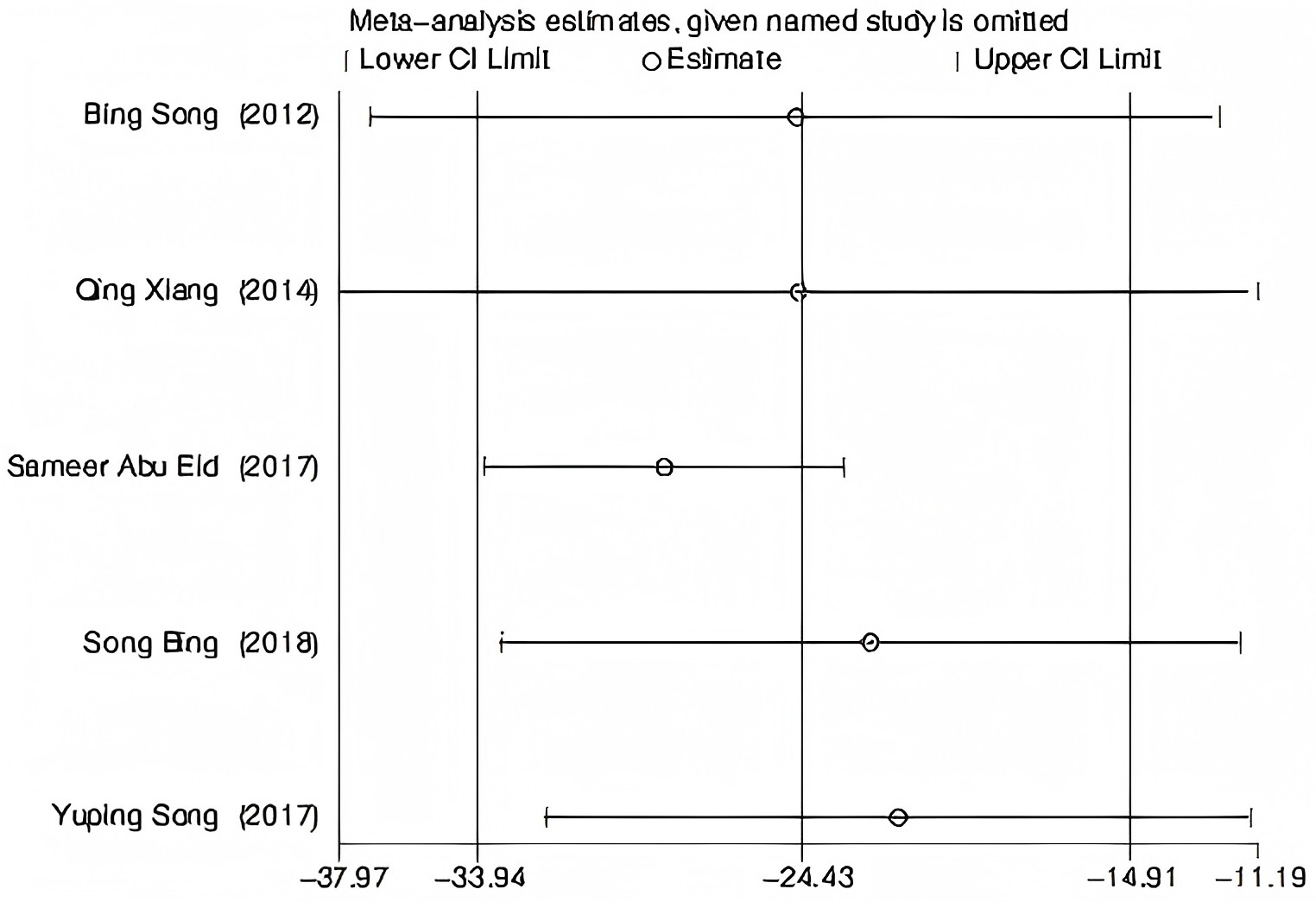

Supplement: Supplemental Information 12 [file peerj-13-19221-s012.png]

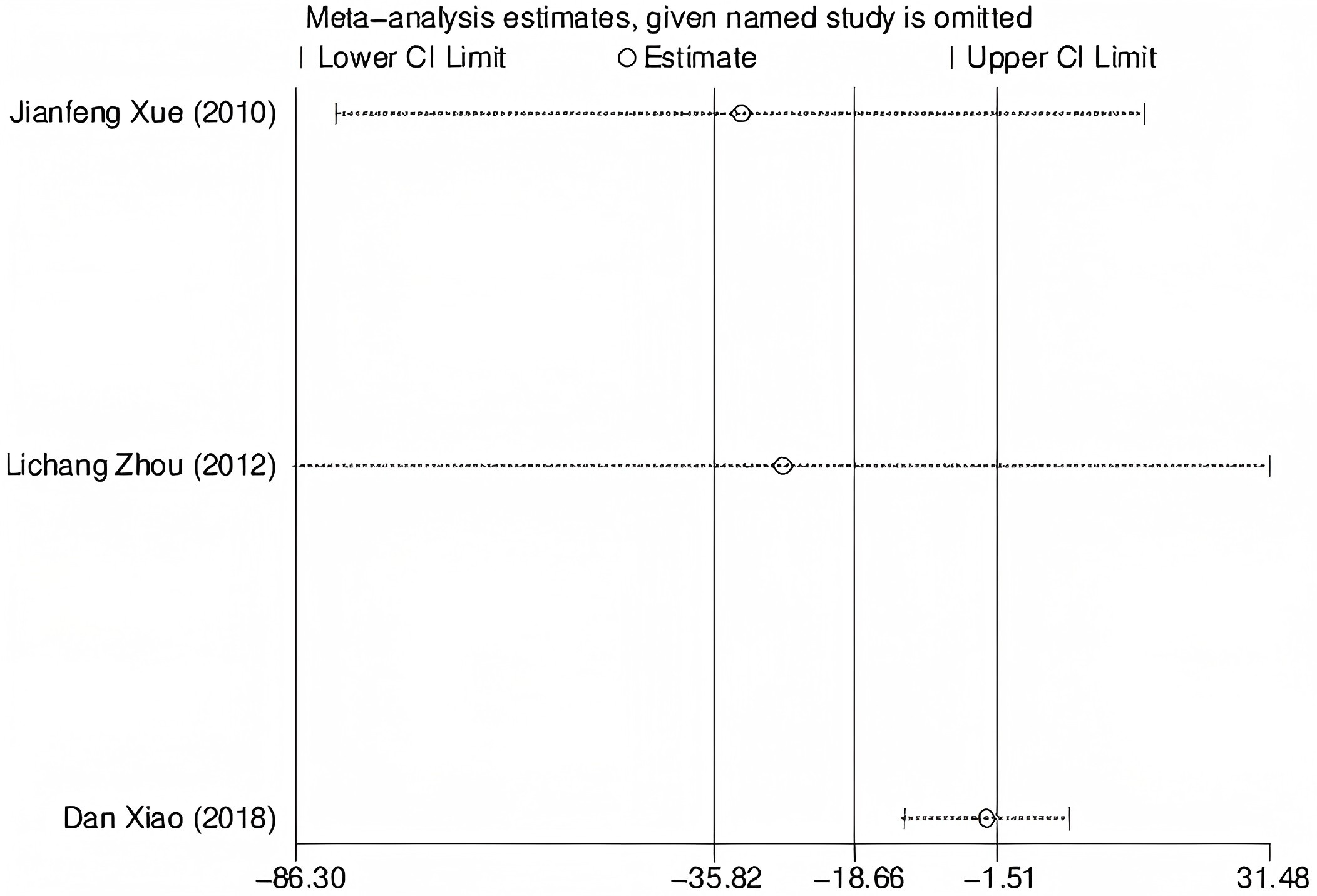

Supplement: Supplemental Information 13 [file peerj-13-19221-s013.png]

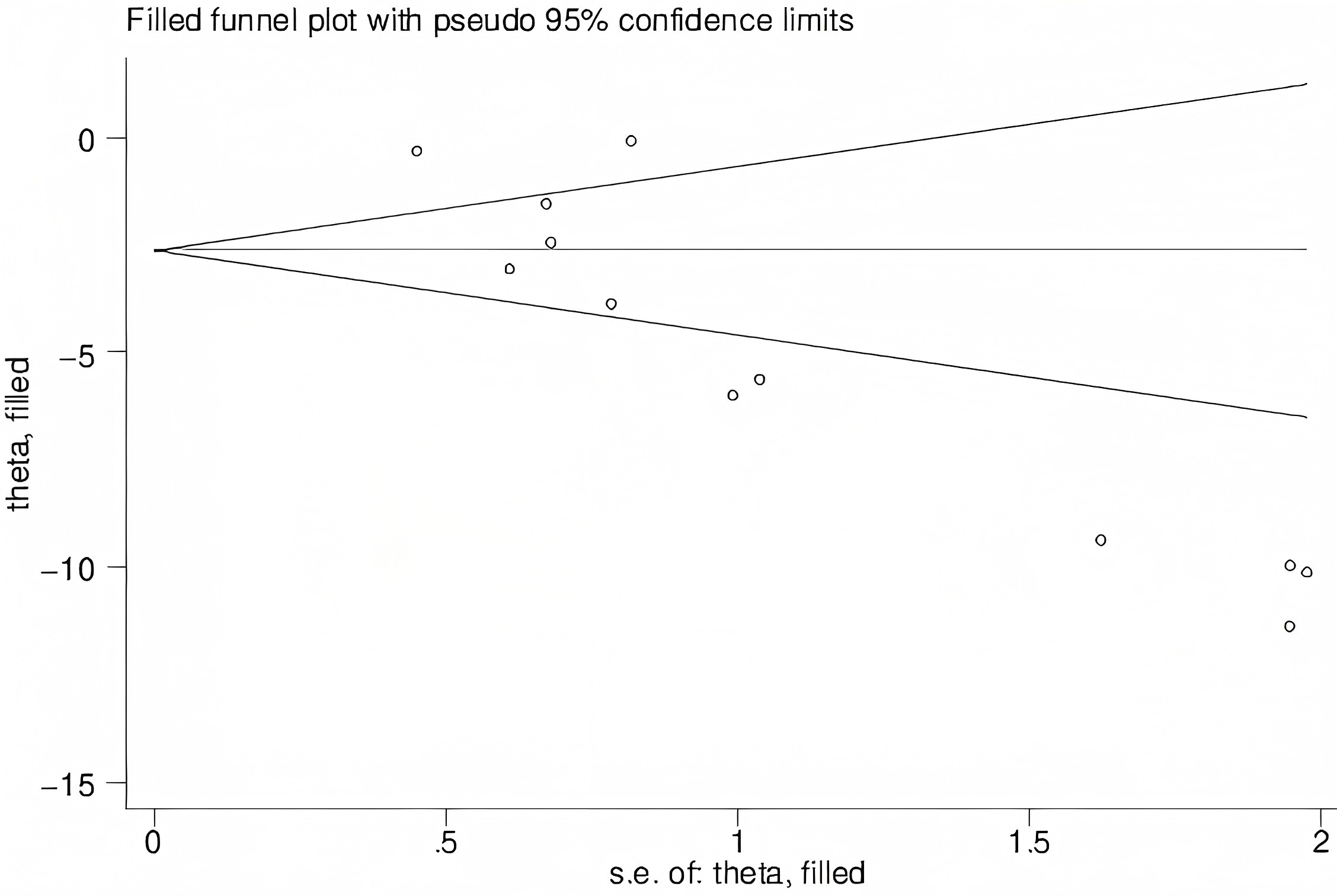

Supplement: Supplemental Information 14 [file peerj-13-19221-s014.png]

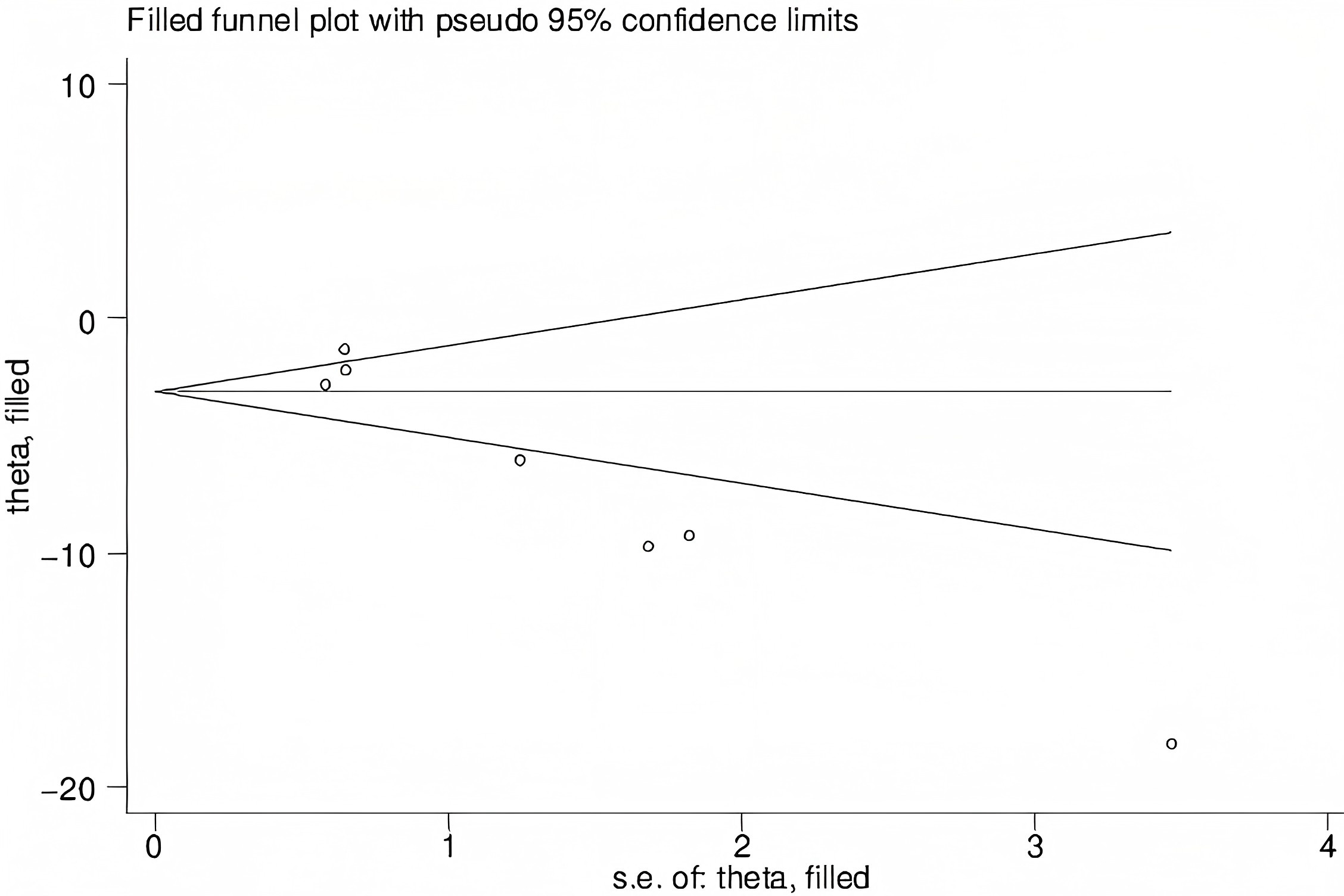

Supplement: Supplemental Information 15 [file peerj-13-19221-s015.png]

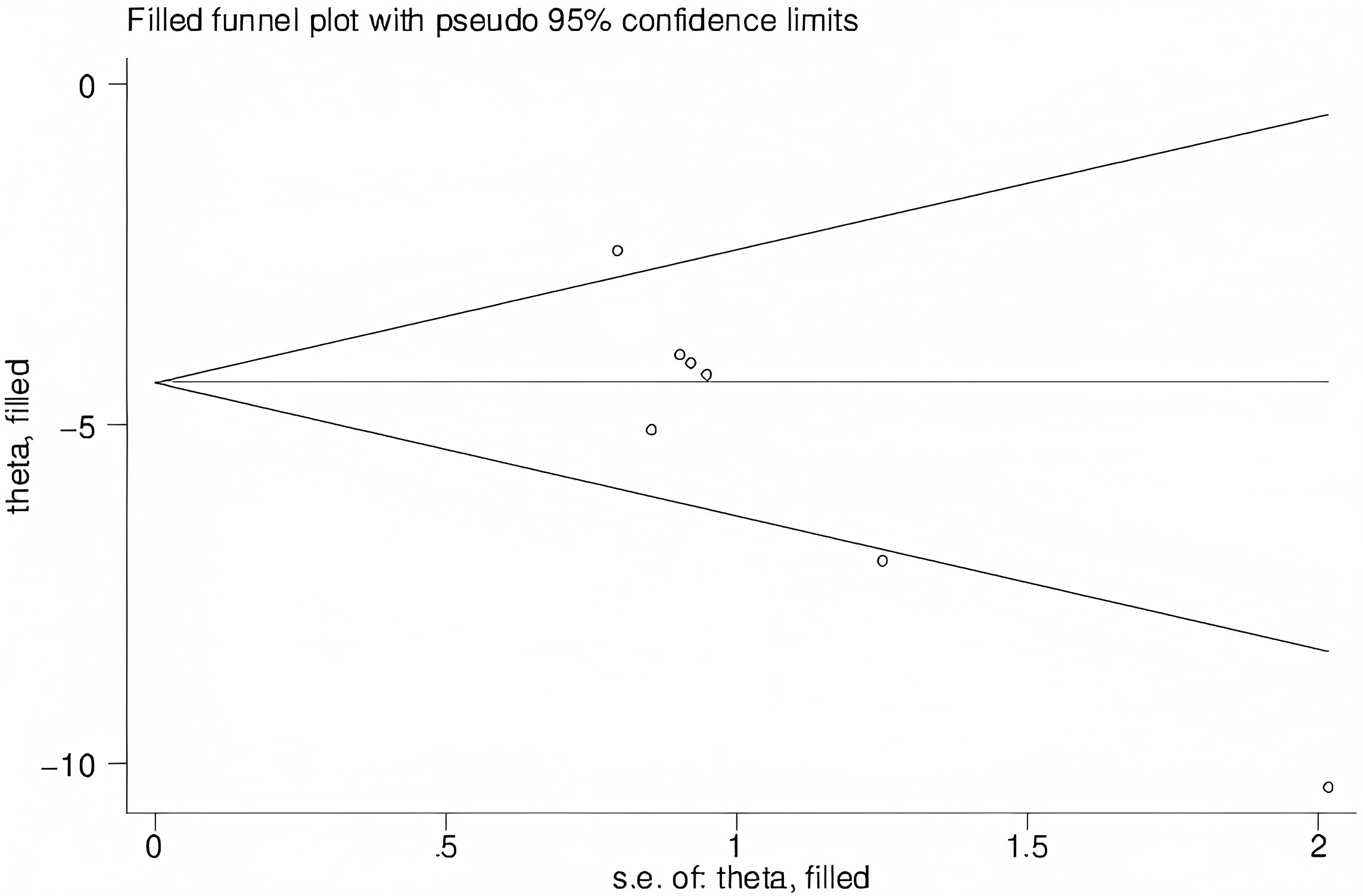

Supplement: Supplemental Information 16 [file peerj-13-19221-s016.png]
