# Supplementary material for: Emodin, a rising star in the treatment of glycolipid metabolism disorders: a preclinical systematic review and meta-analysis
Source: PeerJ. 2025 May 15;13:e19221. doi: 10.7717/peerj.19221 (PMC12085882; doi:10.7717/peerj.19221)
Supplement: Supplemental Information 7 [file peerj-13-19221-s007.docx]

Supplementary Files S7 Results of the Meta-Regression Analysis

| Metareg | Statistics | Dosage | Duration | Species |
| --- | --- | --- | --- | --- |
| FBG | Coefficient | -3.58 | -0.41 | -0.51 |
|  | t | -1.61 | -0.17 | -0.21 |
|  | P>\|t\| | 0.139 | 0.868 | 0.838 |
| IPGTT | Coefficient | 0.67 | 1.03 | -2.13 |
|  | t | 0.32 | 0.51 | -1.59 |
|  | P>\|t\| | 0.764 | 0.636 | 0.187 |
| TC | Coefficient | -4.38 | -1.70 | 4.28 |
|  | t | -0.72 | -0.37 | 0.91 |
|  | P>\|t\| | 0.503 | 0.729 | 0.403 |
| TG | Coefficient | 0.14 | 2.40 | 4.10 |
|  | t | 0.05 | 1.73 | 3.19 |
|  | P>\|t\| | 0.959 | 0.145 | 0.024 |
| INS (WMD) | Coefficient | -22.21 | -0.25 | -0.25 |
|  | t | -3.44 | -0.02 | -0.02 |
|  | P>\|t\| | 0.041 | 0.988 | 0.988 |
| Weight (WMD) | Coefficient | -55.10 | 12.32 | -55.10 |
|  | t | -4.83 | 0.33 | 4.83 |
|  | P>\|t\| | 0.130 | 0.770 | 0.130 |

***NOTE:*** SMD: Standardized Mean Difference; WMD: Weighted Mean Difference. IPGTT: intraperitoneal glucose tolerance test; OGTT: Oral glucose tolerance test
