# Supplementary material for: Emodin, a rising star in the treatment of glycolipid metabolism disorders: a preclinical systematic review and meta-analysis
Source: PeerJ. 2025 May 15;13:e19221. doi: 10.7717/peerj.19221 (PMC12085882; doi:10.7717/peerj.19221)
Supplement: Supplemental Information 8 [file peerj-13-19221-s008.docx]

| Outcome Measures | Subgroup | Criteria for Classification | Doasage | | Duration | | Species | | Studies | Quantity of Studies | N (E/C) | Heterogeneity | | Meta-analysis | |
| --- | --- | --- | --- | --- | --- | --- | --- | --- | --- | --- | --- | --- | --- | --- | --- |
|  |  |  | < 30 mg/kg | ≥ 30 mg/kg | ≤ 4 weeks | > 4 weeks | rats | mice |  |  |  | *I*2 *%* | *p* | SMD/WMD | *95% CI* |
| ***TG*** | Species | rats |  | |  | | **√** | | (Xiao et al., 2019; Zhao et al., 2009) | 2 | 18/18 | 49.3 | 0.160 | -8.30 | -11.49, -5.12 |
|  |  | mice |  |  |  |  |  |  | (Song & Liu, 2012; Wang et al., 2012; Xue et al., 2010; Xuezheng et al., 2018; Zhou et al., 2012) | 5 | 45/40 | 27.1 | 0.241 | -3.93 | -4.83, -3.02 |
| ***INS*** | *Doasage* | < 30 mg kg | **√※** | |  | |  | | (Abu et al., 2017) | 1 | 12/12 | NA | NA | -6.38 | -10.56, -2.20 |
|  |  | ≥ 30 mg kg |  |  |  |  |  |  | (Song & Liu, 2012; Song et al., 2017; Xiang et al., 2014; Xuezheng et al., 2018) | 4 | 34/34 | 78.6 | 0.003 | -28.50 | -33.72, -23.27 |
| *NOTE：*N:Number; E:emodin group; C: control group; √: A complete subgroup analysis was conducted; √※: Owing to the scarcity of articles, a comprehensive subgroup analysis was not feasible; SMD: Standardized Mean Difference; WMD: Weighted Mean Difference | | | | | | | | | | | | | | | |
|  |  |  |  |  |  |  |  |  |  |  |  |  |  |  |  |

**Supplementary Files S8 The result of subgroup**
