# Supplementary material for: Emodin, a rising star in the treatment of glycolipid metabolism disorders: a preclinical systematic review and meta-analysis
Source: PeerJ. 2025 May 15;13:e19221. doi: 10.7717/peerj.19221 (PMC12085882; doi:10.7717/peerj.19221)
Supplement: Supplemental Information 17 [file peerj-13-19221-s017.docx]

Supplementary Files S17 The assessment results of the GRADE system

| **Certainty assessment** | | | | | | | **№ of patients** | | **Effect** | | **Certainty** | **Importance** |
| --- | --- | --- | --- | --- | --- | --- | --- | --- | --- | --- | --- | --- |
| **№ of studies** | **Study design** | **Risk of bias** | **Inconsistency** | **Indirectness** | **Imprecision** | **Other considerations** | **Experimental group** | **Model Group** | **Relative** | **Absolute** |  |  |
|  |  |  |  |  |  |  |  |  | **(95% CI)** | **(95% CI)** |  |  |
| **FBG** | | | | | | | | | | | | |
| 12 | randomised trials | seriousa | seriousb | seriousc | not serious | publication bias strongly suspectedd | 110 | 105 | - | SMD  4.79(-6.47, -3.1) | ⨁◯◯◯ Very lowa,b,c,d | CRITICAL |
|  |  |  |  |  |  |  |  |  |  |  |  |  |
| **2hPG** | | | | | | | | | | | | |
| 6 | randomised trials | seriousa | seriousb | seriousc | seriouse | none | 58 | 53 | - | SMD  2.85(-4.22, -1.48 ) | ⨁◯◯◯ Very lowa,b,c,e | CRITICAL |
|  |  |  |  |  |  |  |  |  |  |  |  |  |
| **TC** | | | | | | | | | | | | |
| 7 | randomised trials | seriousa | seriousb | seriousc | seriouse | publication bias strongly suspectedd | 63 | 58 | - | SMD  5.97(-8.16, 3.43) | ⨁◯◯◯ Very low*^a,b,c,d,e^* | IMPORTANT |
|  |  |  |  |  |  |  |  |  |  |  |  |  |
| **TG** | | | | | | | | | | | | |
| 7 | randomised trials | seriousa | not serious | seriousc | very seriouse | publication bias strongly suspectedd | 63 | 58 | - | SMD  4.84(-6.21, -3.47) | ⨁◯◯◯ Very low*^a,c,d,e^* | IMPORTANT |
|  |  |  |  |  |  |  |  |  |  |  |  |  |
| **INS** | | | | | | | | | | | | |
| 5 | randomised trials | seriousa | seriousb | seriousc | seriouse | none | 46 | 46 | - | WMD  12.63(-21.03, -4.24) | ⨁◯◯◯ Very low*^a,b,c,e^* | IMPORTANT |
|  |  |  |  |  |  |  |  |  |  |  |  |  |
| **Weight** | | | | | | | | | | | | |
| 3 | randomised trials | seriousa | seriousb | seriousc | seriouse | none | 31 | 26 | - | WMD  -18.66 (-35.82, -1.51) | ⨁◯◯◯ Very low*^a,b,c,e^* | IMPORTANT |
|  |  |  |  |  |  |  |  |  |  |  |  |  |
| ***NOTE:* CI:** confidence interval; **SMD**: standardised mean difference; **WMD**: Weighted mean difference. **Explanations:** a. Despite the fact that the results of some of the original studies reported that the baseline data for the experimental group and the control group were consistent, most of the studies did not clearly describe the process of generating the random sequence, concealing the allocation scheme, and blinding, which may lead to a high risk of bias in various fields. b. The overlap ratio is poor, I2> 50%. c. Most of the studies included in this review used various rat or mouse models, so the findings from lower-order animals may be somewhat indirect in relation to relevant outcome measures. d. The P-value for the Eggers test is less than 0.05. e. There are fewer samples. | | | | | | | | | | | | |
