# Supplementary material for: Emodin, a rising star in the treatment of glycolipid metabolism disorders: a preclinical systematic review and meta-analysis
Source: PeerJ. 2025 May 15;13:e19221. doi: 10.7717/peerj.19221 (PMC12085882; doi:10.7717/peerj.19221)
Supplement: Supplemental Information 19 [file peerj-13-19221-s019.zip › 中文纳入的文献/大黄素对2型糖尿病大鼠脂肪...葡萄糖转运蛋白4表达的影响_向青.pdf]

# 大黄素对 2 型糖尿病大鼠脂肪组织葡萄糖转运蛋白 4 表达的影响

向 青,赖文芳,许 文,张小琴
(福建中医药大学生物医药研发中心,福建 福州 350122)

关键词:大黄素;2 型糖尿病大鼠;胰岛素抵抗;葡萄糖转运蛋白 4
中图分类号:R285.5 文献标志码:A 文章编号:1004-5627(2014)05-0022-03
DOI:10.13261/j.cnki.jfutcm.002960

葡萄糖的利用障碍和生成增加导致 2 型糖尿病的发生,胰岛素介导下的葡萄糖转运蛋白,尤其是葡萄糖转运蛋白 4(GluT-4)作为葡萄糖进入组织细胞的关键因素,与葡萄糖的摄取障碍和胰岛素受体后缺陷疾病密切相关。我们通过实验观察大黄素对 2 型糖尿病大鼠皮下脂肪组织细胞膜上 GluT-4 表达的影响,探讨其降血糖的作用机制。

## 1 实验材料

1.1 动物 SPF 级健康雄性 Wistar 大鼠 30 只,体质量 180~220 g,3 月龄,购自上海斯莱克动物实验有限公司,许可证号:SCXK(沪)2012-0002。

1.2 药物 大黄素购自陕西森弗生物技术有限公司,纯度≥98%,批号:20111120006。

1.3 主要试剂和仪器 链脲佐菌素(STZ)(美国 Sigma 公司);细胞膜蛋白与细胞浆蛋白抽提试剂盒(中国碧云天生物技术研究所);兔抗鼠多克隆 GluT-4 抗体(英国 Abcam 抗体公司);ChemiDoc XRS+凝胶成像系统(美国 Bio-rad 公司);HM325 石蜡切片机(美国 Thermo Fisher 公司);LSM710 激光共聚焦显微镜(德国 Zeiss 公司)。

## 2 实验方法

2.1 大鼠模型的建立与分组 SPF 级健康雄性大鼠 30 只,适应性喂养 1 周后,随机分为正常对照组 10 只、造模组 20 只。正常对照组给予基础饲料喂养,自由摄水;造模组给予高脂高糖饲料(饲料组成:15%蔗糖、10%猪油、4%胆固醇、10%蛋黄粉、0.3%胆酸盐、60.7%基础饲料)喂养。喂养 6 周后造模,造模前禁食不禁水 12 h,造模组大鼠按 25 mg/(kg/m$^2$)(>200 g 部分按体表面积计算)腹腔注射 1% STZ;正常对照组腹腔注射同等量的生理盐水。72 h 后造模组大鼠尾静脉采血,用罗氏血糖仪检测空腹血糖值(FBG),96 h 后检测随机血糖值(RBG)。以 FBG≥7.8mmol/L 和 RBG≥16.7 mmol/L 为 2 型糖尿病大鼠成模标准。有 4 只大鼠血糖未达标,舍去,成功复制模型大鼠 16 只。将造模成功的大鼠随机分为模型组 8 只,给药组 8 只。

2.2 给药方法 给药组灌胃 100 mg/kg 大黄素混悬液(用 0.5%羧甲基纤维素钠溶液配制),模型组和空白对照组灌胃等量的 0.5%羧甲基纤维素钠溶液。每日 1 次,连续 4 周后处死。实验期间,自由饮水,每周检测空腹血糖。

2.3 标本收集 大鼠禁食不禁水 12 h,称体质量后,按 1 mL/100 g 腹腔注射 0.3%异戊巴比妥钠麻醉,腹主动脉采血,收集血标本 4 000 r/min 离心 10 min,取上清液存于-80℃冰箱。迅速取皮下脂肪组织置于冷冻管中,投入液氮暂时保存,取材结束后转移至-80℃冰箱保存;皮下脂肪组织切成 1 cm×1 cm×0.2 cm 小块,置于 4%多聚甲醛溶液中固定,进行免疫荧光实验。

2.4 检测指标与方法 血糖用罗氏血糖仪测定;胰岛素用 $^{125}$I 放射免疫分析试剂盒测定。

2.5 GluT-4 蛋白表达的检测 固定后皮下脂肪组织经酒精梯度脱水、浸透、石蜡包埋、切片、按常规免疫荧光法染色,用兔抗鼠多克隆 GluT-4 抗体作为一抗(稀释倍数 1:100),加羊抗兔 IgG-FIFC 二抗进行荧光染色,用 4′,6-二脒基-2-苯基吲哚(DAPI)染核。每张切片染色后,用激光共聚焦显微镜观察、拍照。

2.6 GluT-4 蛋白表达的检测 用细胞膜蛋白与

收稿日期:2014-04-29
作者简介:向青(1985—),女,助理实验师,主要从事中药药理学研究。

© 1994-2021 China Academic Journal Electronic Publishing House. All rights reserved. http://www.cnki.net

细胞浆蛋白抽提试剂盒提取皮下脂肪组织的细胞膜蛋白,将蛋白样品定量,变性;进行聚丙烯酰胺凝胶电泳;转膜、碧云天封闭液封闭 2 h;洗膜,用兔抗鼠多克隆 GluT-4 抗体作为一抗(稀释倍数 1:1 000),4 ℃孵育过夜, 以考马斯亮蓝染色作为膜蛋白内参(细胞膜上无内参蛋白)。加入辣根过氧化物酶标记的二抗(稀释倍数 1:1 000)后,室温孵育 2 h,然后用 TBS 缓冲液洗膜。加入 1:1 的 ECL 发光液,用凝胶成像系统进行曝光,计算目标条带的灰度值。

## 3　实验结果

**3.1　3 组大鼠空腹血糖 (FBG)、空腹胰岛素 (FINS)、胰岛素抵抗指数(HOMA-IR)检测**　结果见表 1。HOMA-IR 采用 Homa Model 公式[1]计算:

$$HOMA-IR = FBG\,(mmol/L) \times FINS\,(\mu IU/mL) / 22.5$$

表 1　3 组大鼠 FBG、FINS、HOMA-IR 检测结果($\bar{x}\pm s$)

| 组　别 | $n$ | FBG /(mmol/L) | FINS/(μIU/mL) | HOMA-IR |
|---|---|---|---|---|
| 正常对照组 | 10 | 5.7 ± 0.17 | 25.83 ± 2.85 | 6.54 ± 1.25 |
| 模　型　组 | 8 | 13.55 ± 1.00 | 45.21 ± 4.27 | 27.23 ± 3.26 |
| 给　药　组 | 8 | 8.79 ± 1.43$^{1)}$ | 20.89 ± 2.26$^{2)}$ | 8.16 ± 1.37$^{1)}$ |

注:与模型组比较,1) $P<0.05$,2) $P<0.01$。

**3.2　GluT-4 蛋白表达的检测**　结果见图 1。

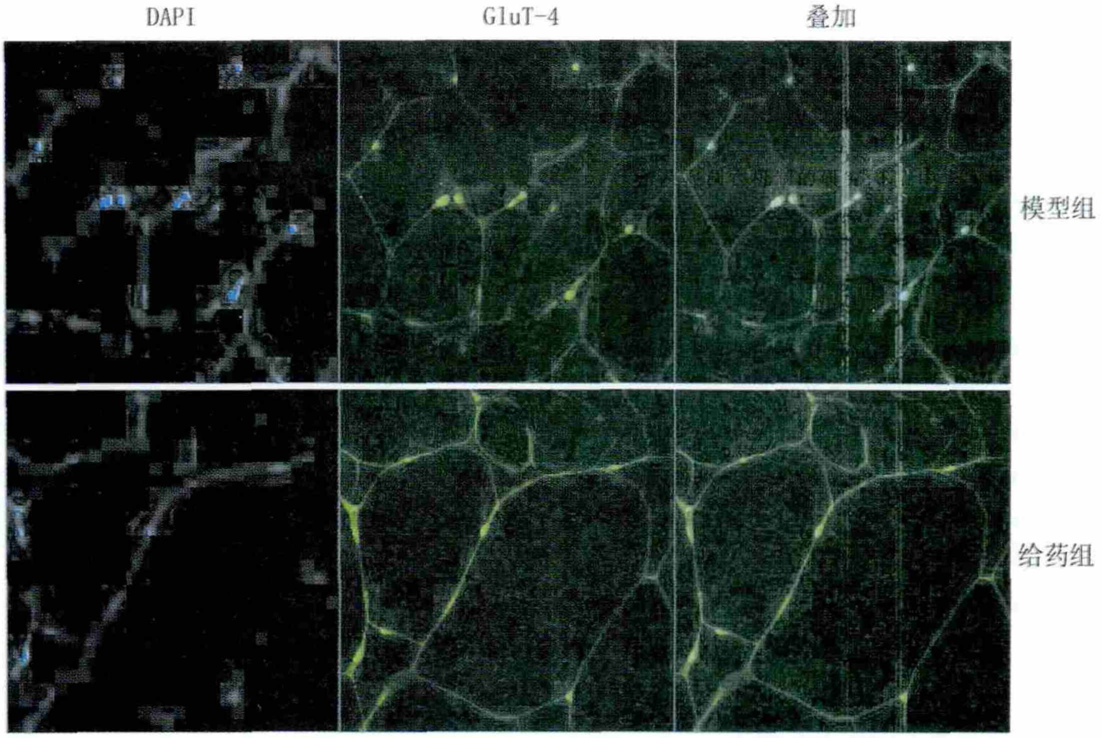


图 1　大鼠皮下脂肪组织细胞膜 GluT-4 蛋白表达图(×400)

免疫荧光染色后,GluT-4 呈现 FIFC 绿色荧光标记,细胞核呈现 DAPI 蓝色荧光标记。结果显示,给药组大鼠皮下脂肪细胞膜上 GluT-4 荧光强度明显较模型组强, 表明给药组大鼠皮下脂肪细胞膜上 GluT-4 蛋白表达量较模型组高。

**3.3　western blot 检测皮下脂肪组织细胞膜 GluT-4 蛋白的表达**　结果图 2。

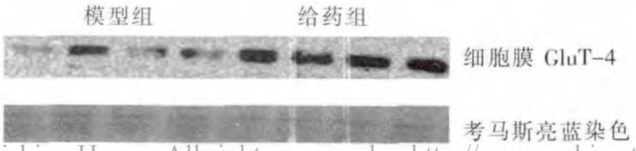


图 2　大鼠皮下脂肪组织细胞膜 GluT-4 蛋白表达图

(C)1994-2022 China Academic Journal Electronic Publishing House. All rights reserved.　http://www.cnki.net

与模型组相比，大黄素能明显增加 T2DM 大鼠皮下脂肪组织细胞膜 GluT-4 表达水平。

## 4 讨 论

4.1 葡萄糖转运子 (glucose transporter，GluT)是受胰岛素特异性调节的葡萄糖摄取的效应蛋白，哺乳类动物细胞中现发现 6 种葡萄糖转运子，分别是 GluT-1～5 和 7。其中 GluT-4 主要分布于细胞内，在肌肉和脂肪组织中转运葡萄糖，它对胰岛素反应敏感而迅速。当胰岛素与靶细胞表面的胰岛素受体 α 亚基结合后，诱发 β 亚基的酪氨酸残基磷酸化，并激活酪氨酸激酶，引起胰岛素受体底物-1 磷酸化。胰岛素受体底物蛋白的多个酪氨酸磷酸化后，与磷脂酰肌醇 3 激酶(phosphatidylinositol 3-kinase，PI3K)亚单位 p85 结合，导致 p110 亚单位被激活。PI3K 被激活后，导致蛋白激酶 B (PKB)磷酸化。磷酸化的 PKB 激活 GluT-4 由细胞质移位至细胞膜，引起肝脏、脂肪和骨骼肌组织对葡萄糖的摄取、胞内糖原的合成，并抑制肝脏葡萄糖异生及输出[2]。

4.2 大黄素是从大黄属、蓼属、鼠李属和番泻叶中分离出来的主要有效单体，其化学名为 1′3′8-三羟基-6-甲基蒽醌，化学结构属于羟基蒽醌类。大黄素药理作用广泛，主要表现为抗肿瘤、抗菌消炎、抑制细胞增殖、提高免疫、保肝、抗肾纤维化等[3]。研究发现，大黄素是过氧化物酶体增殖物激活受体的配体，能促进脂肪细胞分化，抑制前脂肪细胞增殖，促进脂肪细胞的葡萄糖转运[4]，提示大黄素在治疗糖尿病方面具有潜在作用。

4.3 本研究结果显示，大黄素能显著降低 T2DM 大鼠空腹血糖、空腹胰岛素及胰岛素抵抗指数，提示大黄素能够增强 T2DM 大鼠胰岛素敏感性。免疫荧光 western blot 结果显示，大黄素能显著上调 T2DM 大鼠皮下脂肪组织细胞膜上 GluT-4 蛋白的表达。研究结果提示，大黄素能降低 T2DM 大鼠血糖并且改善胰岛素抵抗，其作用机制可能与上调皮下脂肪组织细胞膜上 GluT-4 蛋白表达，进而促进脂肪组织对葡萄糖的摄取有关。大黄素是一种极具研究和开发价值的药物，可能会为以改善胰岛素抵抗为靶点防治糖尿病及其并发症开辟新的途径。

参考文献：

[1] LI G W，LI C M，SUN S X，et al. Is insulin resistance a common pathway for hereditary and environmental factors-induced hypertension[J]. Zhonghua Nei Ke Za Zhi，2003，42(1)：11-15.

[2] KATOME T，OBATA T，MATSUSHIMA R，et al. Use of RNA interference-mediated gene ilencing and adenoviral overexpression to elucidate the roles of AKT/protein kinase B isoforms in insulin actions[J]. J Biol Chem，2003，278(30)：28312-28323.

[3] 黄伟锋. 大黄素的药理作用研究进展[J]. 柳州医学，2013，26(4)：241-244.

[4] YANG Y，SHANG W，ZHOU L，et al. Emodin with PPARgamma ligand-binding activity promotes adipocyte differentiation and increases glucose uptake in 3T3-Ll cells[J]. Biochem Biophys Res Commun，2007，353(2)：225-230.

·简 讯·

## 刘献祥副校长到附属第二人民医院指导科研课题设计

8 月 19 日下午，为提高医院临床科研课题设计水平，进一步加强医院科研课题申报工作，刘献祥副校长应邀到附属第二人民医院作题为“科研选题的方向、范围、原则与方法”的科研讲座，医院科研骨干人员 200 多人参加，部分在医院实习的研究生也聆听了讲座。

附属第二人民医院

(C)1994-2022 China Academic Journal Electronic Publishing House. All rights reserved. http://www.cnki.net
