# Supplementary material for: Emodin, a rising star in the treatment of glycolipid metabolism disorders: a preclinical systematic review and meta-analysis
Source: PeerJ. 2025 May 15;13:e19221. doi: 10.7717/peerj.19221 (PMC12085882; doi:10.7717/peerj.19221)
Supplement: Supplemental Information 19 [file peerj-13-19221-s019.zip › 中文纳入的文献/大黄素对KKAy糖尿病小鼠...γ及GluT-2表达的影响_宋冰.pdf]

大黄素对 KKAy 糖尿病小鼠肝脏 PPAR-γ 及 GluT-2 表达的影响

宋 冰[1]，刘学政[2]
[1] 辽宁医学院附属第一医院，锦州 121000 ；[2] 辽宁医学院，锦州 121000

摘要 ：目的 探讨大黄素改善 KKAy 糖尿病小鼠肝脏胰岛素敏感性及降血糖的作用机制。方法 将 SPF 级 KKAy 小鼠 20 只按血糖值随机分为模型组 (DM)，大黄素治疗组 (EM 组，按 50mg/kg · d 剂量灌胃)，并选 10 只 C57BL/6J 小鼠为正常对照组 (NC)，连续灌胃给药 8 周。8 周后测定血清空腹血糖 (FPG)、空腹胰岛素 (Fins) 并计算胰岛素敏感指数 (ISI)、甘油三酯 (TG)、总胆固醇 (TC) 及过氧化物酶体增殖物激活受体 γ (PPAR-γ) 及葡萄糖转运蛋白 -2mRNA(GluT-2mRNA) 在肝脏的表达水平。结果 与 NC 组比较，DM 组 FPG、TG、TC 明显升高，ISI 明显降低，肝脏 PPAR-γ 及 GluT-2mRNA 的表达丰度明显降低 ($P<0.05$) ；与 DM 组比较 EM 组 FPG、TG、TC 明显降低，ISI 明显升高，肝脏 PPAR-γ 蛋白表达明显升高，积分光密度有统计学差异，GluT-2mRNA 的表达丰度明显升高 ($P<0.05$)。结论 大黄素可以上调 KKAy 糖尿病小鼠肝脏 PPAR-γ 及 GluT-2mRNA 表达，降血糖并改善胰岛素敏感性。

关键词 ：大黄素 ；KKAy 糖尿病小鼠 ；胰岛素敏感性 ；过氧化物酶体增殖物激活受体 γ ；葡萄糖转运蛋白 -2
中图分类号 ：R 587.1 文献标识码 ：A 文章编号 ：1005-1139(2012)07-0775-04

Effect of emodin on expression of PPAR-γ and GLuT-2 in hepatic tissue of mice with KKAy diabetes mellitus

SONG Bing, LIU Xue-zheng
[1]First Affiliated Hospital of Liaoning Medical College, Jinzhou 121000, Liaoning Province, China; [2]Liao Ning Medical University, Jinzhou, 121000, China
Corresponding author: LIU Xue-zheng. Email: jinyisandy@tom.com

Abstract: Objective To study the effect of emodin (EM) on insulin sensitivity in hepatic tissue of mice with KKAy diabetes mellitus (DM) and its mechanism of decreasing blood glucose. **Methods** Twenty specific pathogen free (SPF) KKAy mice were randomly divided into model group (DM group) and EM treatment group according to their plasma glucose level. Ten normal C57BL/6J mice served as a normal control (NC) group. The mice received intragastric lavage with EM for 8 weeks. Then, their fasting serum glucose (FPG) and insulin levels were measured, insulin sensitivity index(ISI), total triacylglycerol(TG), and total cholesterol(TC) were detected, and expression levels of PPAR-γ and GluT-2mRNA in their hepatic tissue were measured by immunohistochemistry or RT-PCR. **Results** The FPG, TG and TC levels were higher while the ISI and expression levels of PPAR-γ and GluT-2mRNA were lower in DM group than in NC group($P<0.05$). The FPG ,TG and TC levels were significantly lower while the ISI and expression levels of PPAR-γ were significantly higher in EM treatment group than in DM group ($P<0.05$). A significant difference was found in the integrated optical density(IOD) between EM treatment group and DM group ($P<0.05$). **Conclusion** EM can up-regulate the expression levels of PPAR-γ and GluT-2mRNA, and down-regulate the insulin sensitivity in mice with KKAy DM.

Key words: emodin; KKAy diabetic mice; insulin sensitivity; peroxisome proliferator activated receptor-γ; glucose transport protein-2

胰岛素抵抗既是多种疾病的共同危险因素又是 2 型糖尿病、代谢综合征的主要病理生理学基础，它已经成为人们最为关注的问题之一。近年来应用胰岛素增敏剂 - 噻唑烷二酮类药物治疗胰岛素抵抗取得了一定疗效，但由于其价格昂贵，增加体质量、增加水液潴留等不良反应 [1]，远期疗效尚待进一步深入研究。中医药在对糖尿病的防治方面有着悠久的历史和确切的疗效，但缺乏对胰岛素抵抗作用机制方面的深入研究。本研究从分子水平探讨大黄素改善 KKAy 糖尿病小鼠胰岛素敏感性 (ISI) 及降血糖的可能机制。

材料和方法

1 实验动物 自发性 2 型糖尿病 KKAy 小鼠 SPF (specific pathogen free) 级 50 只，10 周龄，体质量 (35±2)g 和同周龄的 C57BL/6J 小鼠 10 只，体质量 (29±2) 克，均为雌性，购自中国医学科学院动物研究所。自由摄食、饮水，动物室内设置 12h 昼夜循环，并维持室温 (22±2)℃，饲养于辽宁医学院实验动物中心 SPF 实验室。质量合格证

收稿日期 ：2011-10-09 修回日期 ：2011-11-13
作者简介 ：宋冰，女，在读博士，主治医师。研究方向为胰岛素抵抗。Email: songbing1978@163.com
通信作者 ：刘学政，男，博士，教授，博士生导师。Email: jinyisandy@tom.com

(C) 1994-2022 China Academic Journal Electronic Publishing House. All rights reserved. http://www.cnki.net

号：SCXK 京 2009-004。基础饲料：中国医学科学院动物研究所小鼠全价营养颗粒饲料，用于正常 C57BL/6J 小鼠喂养；高能量饲料：含 10% 蔗糖、10% 脂肪的 KKAy 小鼠专用高能量饲料，用于 KKAy 小鼠喂养。

2 药物与试剂仪器 大黄素购于长沙上禾生物科技有限公司，批号：20101120006。小鼠胰岛素放免试剂盒 (Linco 公司)；兔抗鼠过氧化物酶体增殖物激活受体 γ(PPAR-γ) 多克隆抗体 (Santa Cruz 公司)，RT-PCR 试剂盒 (宝信公司)。低温高速离心机，德国 Hettich 公司；日立 7600 全自动生化分析仪；美国强生血糖仪及配套试纸。

3 动物分组及给药 KKAy 小鼠断尾取血，测随机血糖值≥ 13.9mmol/L 者入选，共 16 只入选。随机将其分为模型组 (DM) 8 只，大黄素治疗组 (EM) 8 只，10 只 C57BL/6J 小鼠为正常对照组 (NC)。大黄素治疗组每日按大黄素 50mg/kg 灌胃共 8 周，NC 组及 DM 组每日按 20ml/kg·d 灌服无菌水 8 周。

4 检测指标 8 周后 KKAy 糖尿病小鼠禁食 6h 后留取血清：空腹血糖 FPG、甘油三酯 TG、总胆固醇 TC 用日立 7600 全自动生化分析仪测定；空腹血清胰岛素 (FINS) 用放射免疫分析法测定。胰岛素敏感指数 (ISI) 采用李光伟法[2]，ISI=1/ 空腹血糖 (FPG)× 空腹血清胰岛素 (FINS)，取自然对数正态化后进行分析。处死 KKAy 糖尿病小鼠，速取部分肝脏，用 4% 多聚甲醛固定，备制石蜡包埋组织切片进行免疫组化染色检测 PPARγ 蛋白表达；另取部分肝脏置入液氮中以备 RT-PCR 检测 GluT-2mRNA 表达。

5 免疫组化染色及结果判定图像分析 采用免疫组织化学链霉抗生物素蛋白 - 过氧化物酶法，一抗 (兔抗鼠 PPAR-γ 多克隆抗体) 工作浓度为 1∶200；采用 3，3 '- 二氨基联苯胺 (DAB) 显色，脂肪细胞核内出现棕黄色颗粒为阳性。采用 Image-ProPlus 6.0 图像分析系统，每张切片随机选择 5 个视野，在分辨率、对比度、亮度等同条件下测定 PPAR-γ 的积分吸光密度值 (IOD)[3]。

6 RT-PCR 方法及结果判定 取各组 KKAy 小鼠肝组织加液氮研碎，采用 Trizol 一步法提取总 RNA。用核酸蛋白定量仪检测所提总 RNA 的纯度与浓度，取 OD260/OD280 值在 1.8-2.0 之间的 RNA 进行实验。根据 GenBank 的小鼠 GluT-2 和 β-actin 基因序列，利用生物软件 Primer 5.0 设计引物。引物由上海生工生物工程技术服务有限公司合成。首先按照 RT 试剂盒要求逆转录合成 cDNA，然后取 1μl 逆转录产物做为模板在 20μl 体系中进行 PCR 扩增。然后 cDNA 第一链的合成：Mgcl 22μl，10*RT Buffer 1μl，Rnase Free DH_2O 3.75μl，dNTP Mixture(各 10mmol/L) 1μl，Rnase inhibitor 0.25μl，AMV Reverse Transcriptase 0.5μl，Oligo dT-Adaptor Primer 0.5μl。实验样品 RNA 1μl，Total 10μl/sample，反转录条件：42°C -55°C，30min，99°C，5min，一个循环，5°C，5minPCR 反应体系及条件：反应体系 (20μl 体系)，Buffer 3μl，dNTP 1μl，目的基因引物上游 1μl，β-actin 上游 1μl，β-actin 下游 1μl，taq 酶 0.2μl，CDNA 模板 1μl，超纯水 10.8μl。引物及反应条件：GluT-2：5'-TACGGCAATGGCTTTATC-3'，208bp，5'-CCTCCTGCAACTTCTCAAT-3'。PCR 条件 94 °C 5min，94 °C 45s，54 °C 45s，72 °C 45s，最后延伸：72 °C 10min，4 °C 保存。β-actin：5-ATCATGTTTGAGACCTTCAACA-3 '，308bp，5 '-CATCTCTTGCTCGAAGTCCA-3 '。PCR 结束后将反应产物进行 1% 琼脂糖凝胶电泳，然后用凝胶成像分析系统拍照，并利用图像分析软件 Gene Tools 分析结果，利用 β-actin 作为 RT-PCR 的内标，以特异基因条带亮度与内标的比值作为表达丰度，分析比较 GluT-2 基因在 KKAy 小鼠肝组织中的表达情况。

7 统计学方法 应用 SPSS15.0 统计软件分析。计量资料以 x±s 表示，进行方差分析并进行组间两两比较，P<0.05 为差异有统计学意义。

结 果

1 各组小鼠一般状态 NC 组小鼠状态良好，表现活跃，反应灵敏。DM 组及 EM 组随周龄增长逐渐出现不同程度的精神萎糜、倦怠、反应迟钝，行动缓慢等，DM 组最为明显，EM 组较之有所改善，实验中无动物死亡。

2 大黄素对 KKAy 糖尿病小鼠 FPG、Fins、ISI、TC、TG 的影响 与 NC 组比较，DM 组 FPG、Fins、TC、TG 水平均明显升高 (P<0.05)，ISI 明显降低 (P<0.05)；与 DM 组比较，EM 组 FPG、Fins、TC、TG 水平均明显降低 (P<0.05)，ISI 明显升高 (P<0.05)，见表 1。

3 大黄素对 KKAy 糖尿病小鼠肝脏 PPAR-γ 的影响 NC 组肝小叶结构完整，肝细胞排列清楚整齐，胞浆丰富，胞核清晰。DM 组肝小叶结构紊乱，肝细胞胞浆有明显空泡，肝细胞有脂肪变性。免疫组化法检测三组小鼠肝脏 PPAR-γ 表达结果显示：与 NC 组比较，DM 组肝脏 PPAR-γ 核

(C)1994-2022 China Academic Journal Electronic Publishing House. All rights reserved. http://www.cnki.net

阳性表达明显降低 (P<0.05); 与 DM 组比较，EM 组肝脏 PPAR-γ 核阳性表达明显升高 (P<0.05)，见图 1。

4　大黄素对 KKAy 糖尿病小鼠肝脏 GluT-2mRNA 表达的影响　与 NC 组比较，DM 组肝脏 GluT-2mRNA 表达明显降低 (P<0.05)，与 DM 组比较，EM 组肝脏 GluT-2mRNA 表达明显升高 (P<0.05)，见图 2、图 3。

表 1　各组 FPG、Fins、ISI、TC、TG 的比较

Tab 1　FPG, Fins, ISI, TC, and TG levels in different groups ($\bar{x}\pm s$)

Group	FPG(mmol/L)	Fins(mU/L)	ISI	TC(mmol/L)	TG(mmol/L)
NC(n=10)	5.29±0.20	18.37±1.79	-1.23±0.09	2.88±0.26	0.51±0.09
DM(n=8)	8.60±0.33^a	54.13±6.23^a	-1.83±0.14^a	5.63±0.57^a	2.80±0.57^a
EM(n=8)	5.65±0.30^b	30.18±2.02^b	-1.67±0.09^b	3.04±0.21^b	1.06±0.19^b

^{a}P<0.05, vs NC group; ^{b}P<0.05, vs DM group

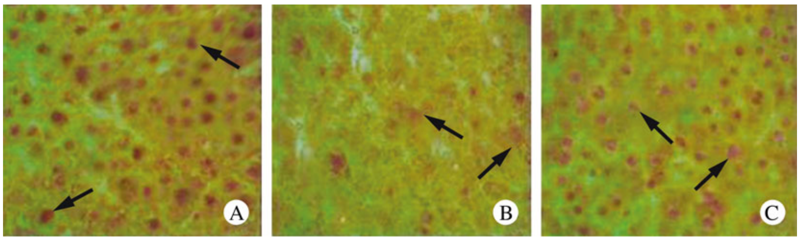

图1　各组肝脏PPAR-γ 的表达(A为NC组,B为DM组，C为EM组)

Fig 1　Expression of PPAR-γ in hepatic tissue of NC group (A),DM group (B), and EM treatment group(C)

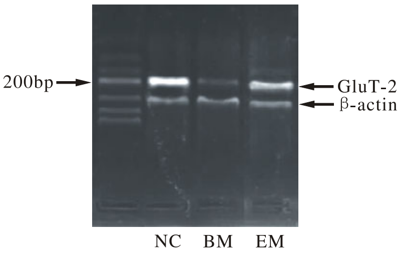

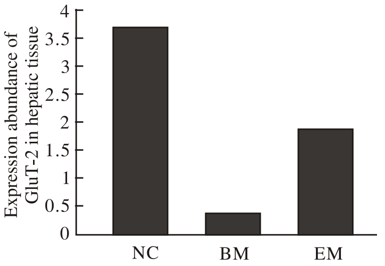

图 2　各组肝脏GluT-2的表达(左)

图 3　各组肝脏GluT-2表达丰度的比较(右)

Fig2　Expression of GluT-2 in hepatic tissue of different groups(left)

Fig3　Expression abundance of GluT-2 in hepatic tissue of different groups(right)

讨　论

KKAy 小鼠是在 KK 小鼠的基础上转入突变毛色基因 (ay) 而成，其 ay 基因不仅影响小鼠毛色而且还可引起代谢紊乱，并出现肥胖、高血糖、脂质代谢紊乱和高胰岛素血症等代谢异常综合征，故发病是在遗传易感的基础上加环境因素而诱发 [4-5]，和人类 2 型糖尿病的表现极为相似，是一种较理想的自发性 2 型糖尿病动物模型。C57BL/6J 小鼠呈黑色，与 KK 小鼠具有基因同源性而作为正常对照组 [6]。

PPAR-γ 是一类核激素受体，在调节糖脂代谢，减轻炎症进而改善胰岛素抵抗 (IR) 方面起着重要的作用。PPAR-γ 在脂肪、肝脏、骨骼肌等组织中有不同程度的表达 [7]，在体内与配体结合后被激活，通过与目的基因启动子内 PPRE 作用，激活或抑制下游基因转录 [8]。GLuT-2 作为 PPAR-γ 下游可调控的靶基因之一，其表达增强能改善肝脏的葡萄糖摄取和利用 [9]。

大黄素 (emodin) 化学名称为 6- 甲基 -1，3，8- 三羟基蒽醌，是从大黄属等中药中分离后得到的主要有效单体。现代药理研究认为大黄具有纠正血脂代谢紊乱、抗炎、改善微循环、调节免疫等作用 [10]。已有细胞学实验发现大黄素是 PPARγ2 的配体，能促进脂肪细胞分化，抑制前脂肪细胞增殖，促进脂肪细胞的葡萄糖转运 [11]，提示大黄素在治疗糖尿病方面的潜在作用。

本研究结果提示，与糖尿病组比较，大黄素能够有效降低 KKAy 糖尿病小鼠的血糖和血脂并能上调 KKAy 糖尿病小鼠肝脏 PPARγ 及 GluT-2mRNA 表达。综上所述，本研究证实了大黄素可以降低血糖并且改善胰岛素敏感性，可以增强 KKAy 糖尿病小鼠肝脏 PPAR-γ 的表达，并上调 GluT-2mRNA 表达。大黄素可能通过正向调节 PPAR-γ 和 GluT-2mRNA 的表达，来改善肝脏葡萄糖利用不足和过度释放，从而降低血糖并改善胰岛素敏感性。因此，大黄素作为一种新型 PPAR-γ 激动剂，是一种极具研究和开发价值的药物，可能成为以改善胰岛素抵抗为靶点防治糖尿病及其并发症开辟新的途径。

参考文献

1　周丽斌，陈名道．胰岛素增敏剂噻唑烷二酮类药物的研究进展［J］．实用糖尿病杂志，2005，1（6）：58-59．

2　李光伟，潘孝仁，Lillioja S，等．检测人群胰岛素敏感性的一项新指数［J］．中华内科杂志，1993，13（10）：656．

3　吕宏升，朱庆生，王军，等．全自动显微镜及图象分析系统处理免疫组化图像［J］．中国体视学与图象分析，2004，9（1）：37-40．

4　Mita T，Goto H，Azuma K，et al. Impact of insulin resistance on enhanced monocyte adhesion to endothelial cells and atherosclerogenesis Independent of LDL cholesterol level［J］．Biochem Biophys Res Commun，2010，395（4）：477-483.

（下转790页）

(C)1994-2022 China Academic Journal Electronic Publishing House. All rights reserved.　http://www.cnki.net

讨　论

从表 1 可看出 70% 以上的老年患者依从情况相对一致，他们意识到减少陪护可以减少感染、噪音等不利因素，服从医护人员的专业治疗和管理模式更利于他们的疾病恢复和健康生活习惯的建立。说明大多数患者信任医院有能力给予他们生理心理全方面的照顾。

从表 2 可看出患者在失去陪护的情况下排序最高的是安全问题，担心意外事件发生。说明面对先进医疗技术迅速发展和患者法律意识不断增强及护理模式的改变，患者自我保护意识也在提升[1]。当医院在完善管理流程，加强风险评估的同时，患者同样对保护自身安全予以重视，从第 3 项可以看出他们认为有专人看护更能获得安全感。表 2 中第 2、5 项涉及到患者的基础护理和生活照料，虽然由护士代替陪护来完成基础护理可以减轻家属负担，但医院以往的床护配比中，临床护理人员的配置无法满足病人的需求[2]。尽管做好基础护理是护士的本职也是患者的期望，但由于长期以来主客观因素的影响，基础护理工作一直做的不够好[3]，所以在实施无陪护管理模式的初期，患者的担忧依然强烈。从第 4、6 项表现出患者对亲情有强烈的渴望，加之固定单调的治疗程序，患者更渴望亲人的关怀，这种感情也是医护人员不能替代的。排序的后 4 位则体现了患者的个性化需求，老年患者思维模式固定，性格固执敏感，加之长期的生活习惯，使他们不愿意被约束和被统一管理，其中一部分更愿意住院期间的管理模式回归家庭化或养老院模式。

表 2 可见高龄老年组和中低龄老年组均有差异的项目 4 项。涉及到亲情需要的 1 项，个性化需求的 3 项。高龄老年组患者较中低龄患者往往体质更加虚弱，合并症多，生活自理能力差，孤僻忧郁感强。加之老年患者家庭观念重，高龄老年人表现出更多的精神需要和亲情依赖。而相对低龄的老年患者病情相对较轻，行动方便，性情任性偏执，他们更希望被人重视尊敬，当从熟悉的家庭来到陌生的环境中，放弃原来的生活习惯会产生抵触和不愉快的情绪。

从患者担心的问题入手，无陪化管理不是一刀切，不是强制管理。对入院患者进行风险预见性评估，对于精神障碍，意识不清，情绪不稳，病情危重患者准许留陪，使病房保持一定的留陪率，待风险解除后逐步过渡到无陪。完善患者生活上的照顾，配备合理床护比，落实患者基础护理的同时，科室也要加强环节质量管理和监控，切实保证护理质量。而针对患者个性化需求要在充分理解关心患者的基础上帮助他们尽快适应医院环境和制度，教授他们健康知识，掌握科学合理的自我管理方法，自觉改变不良的生活方式，尊重他们的权利和需要，提供人性化的硬件设施，改善住院条件，增加康复活动场地，减少患者单调乏味感，从而提高患者对无陪护管理模式的依从性。

参考文献

1　袁景馨，魏艳丽，尤丽娜. 护理风险评估与护理管理干预［J］. 中国误诊学杂志，2011，11（5）：1116-1117.

2　李运梅，陈静，何冰，等. 医院陪护人员现状调查［J］. 护理研究，2008，22（29）：2704-2705.

3　李惠玲，杨惠花，眭文洁，等. 建立“心中有病人”的基础护理服务链［J］. 中国护理管理，2010，10（4）：25.

（上接777页）

5　Chen R，Liang F，Morimoto S，et al. The effects of a PPARalpha agonist on myocardial damage in obese diabetic mice with heart failure［J］. Int Heart J，2010，51（3）：199-206.

6　Khan AH，Capilla E，Hou JC，et al. Entry of newly synthesized GLUT4 into the insulin-responsive storage compartment is dependent upon both the amino terminus and the large cytoplasmic loop［J］. J Biol Chem，2004，279（36）：37505-37511.

7　Dubois M，Pattou F，Kerr-Conte J，et al. Expression of peroxisome proliferator-activated receptor gamma（PPARgamma）in normal human pancreatic islet cells［J］. Diabetologia，2000，43（9）：1165-1169.

8　Uchimura K，Nakamuta M，Enjoji M，et al. Activation of retinoic X receptor and peroxisome proliferator-activated receptor-gamma inhibits nitric oxide and tumor necrosis factor-alpha production in rat Kupffer cells［J］. Hepatology，2001，33（1）：91-99.

9　Kim HI，Ahn YH. Role of peroxisome proliferator-activated receptor-gamma in the glucose-sensing apparatus of liver and beta-cells［J］. Diabetes，2004，53 Suppl 1：S60-S65.

10　李淑娟，黄晓华，武海霞，等. 大黄及其有效成份药理作用研究进展［J］. 医学综述，2005，11（1）：76-78.

11　Yang Y，Shang W，Zhou L，et al. Emodin with PPARgamma ligand-binding activity promotes adipocyte differentiation and increases glucose uptake in 3T3-Ll cells［J］. Biochem Biophys Res Commun，2007，353（2）：225-230.

(C)1994-2022 China Academic Journal Electronic Publishing House. All rights reserved.　http://www.cnki.net
