# Supplementary material for: Emodin, a rising star in the treatment of glycolipid metabolism disorders: a preclinical systematic review and meta-analysis
Source: PeerJ. 2025 May 15;13:e19221. doi: 10.7717/peerj.19221 (PMC12085882; doi:10.7717/peerj.19221)
Supplement: Supplemental Information 19 [file peerj-13-19221-s019.zip › 中文纳入的文献/大黄素对糖尿病大鼠血糖和周围神经病变的影响_高霏.pdf]

基金项目：

甘肃省中医药管理局课题（编号 GZK-2017-34）。

作者简介：

雒玉辉（1978－），女，硕士学位，主治医师，研究方向为皮肤病的中医药诊疗及皮肤病理。

编辑：田杏茹 编号：EA-4180601002（修回：2019-03-02）

大黄素对糖尿病大鼠血糖和周围神经病变的影响

Efficacy of emodin on blood glucose and peripheral neuropathy in diabetic rats

高　霏

（南阳医学高等专科学校，河南　南阳，473000）

中图分类号：R587.1　文献标识码：A　文章编号：1674-7860（2019）07-0024-03　证型：DGB

【摘　要】目的：观察大黄素对链尿佐菌素诱导大鼠糖尿病血糖和周围神经病变的影响及可能作用机制。方法：链脲佐菌素诱导糖尿病大鼠模型，随机分为模型组和治疗组，每组 10 只。另设对照组大鼠 10 只。三组连续给药 8 周。观察药物对糖尿病大鼠空腹血糖、糖耐量、坐骨神经山梨醇水平以及脊髓致炎细胞因子水平。结果：大黄素可显著抑制脊髓致炎细胞因子肿瘤坏死因子 -α（TNF-α）和白介素 1β（IL-1β）水平，明显降低糖尿病大鼠血糖和各时间点血糖曲线下面积（$P < 0.01$）；治疗组坐骨神经山梨醇水平明显低于模型组（$P < 0.01$）。结论：大黄素对糖尿病大鼠周围神经病变具有明显的改善作用，其作用机制可能与改善体内山梨醇代谢和抑制脊髓致炎细胞因子水平有关。

【关键词】大黄素；糖尿病；血糖；神经病变

【Abstract】Objective: To observe possible mechanism and efficacy of emodin on blood glucose and peripheral neuropathy in STZ diabetic rats. Methods: STZ diabetic rats were divided into the model group and the treatment group, 10 rats per group. In addition, the control group was established with 10 rats. All groups were administered for 8 weeks. Results: Emodin can significantly inhibit the levels of inflammatory cytokines TNF-α and IL-1β in the spinal cord, significantly reduce the blood glucose level and the area under the blood glucose curve at each time point in diabetic rats (P<0.01); the level of sciatic nerve sorbitol was significantly lower than that in the model group (P<0.01). Conclusion: Emodin has a significant improvement effect on peripheral neuropathy in diabetic rats, and the mechanism may be related to the improvement of sorbitol metabolism and inhibition of spinal cord proinflammatory cytokine levels.

【Keywords】Emodin; Diabetes; Blood sugar; Neuropathy

doi:10.3969/j.issn.1674-7860.2019.07.008

糖尿病是一种危害性较大的常见疾病，是由多种病因引起的代谢异常症。糖尿病最常见的并发症之一便是糖尿病周围神经病变，其发病率高达 40% ～ 90%[1]。由于其发病机制较为复杂，临床常用药物如抗抑郁、抗癫痫、离子通道阻断药等均不能令人满意[2]。因此，研究新的作用机制、寻找新的药物是目前的研究热点。

大黄素是中药大黄、虎杖、决明子等药材的有效成分之一。近年来大量药理学研究证实大黄素具有多种不同的生物学功能，包括抗肿瘤、抗炎、抗菌、抗病毒、抗过敏、降血糖、免疫抑制、神经保护、护肝等[3-5]。前期研究显示大黄素具有较为明显的止痛作用，但相关的报道文献较少。本实验观察了大黄素对糖尿病大鼠血糖和周围神经病变的影响，为其临床治疗提供理论基础。

1　材料与方法

1.1 动物

7 周龄雄性 SD 大鼠，体质量 180 ～ 220 g 及 SPF 级饲料，均购于广州中医药大学实验动物中心，合格证号 SYXK（粤）2013-0092。适应环境 1 周后进行实验。

1.2 药物与试剂

大黄素（成都曼斯特生物科技有限公司，纯度＞ 98%；链脲佐菌素由美国 Sigma 公司提供；TNF-α 和 IL-1β ELISA 试剂盒，美国 Abcam 公司提供。

1.3 分组和给药

取 SD 大鼠，禁食 12 h 后，1% 链脲佐菌素溶液，腹腔给药，60 mg/kg，72 h 后于大鼠眼静脉取血，测定空腹血糖，当血糖值大于 16.7 mmol/L 确定为糖尿病大鼠。将糖尿病大鼠随机分为模型组和治疗组，每组 10 只。另设对照组 10 只。治疗组给予大黄素 100 mg/kg 灌胃，模型组和对照组灌胃同体积的生理盐水，连续给药 8 周。

1.4 观察指标

1.4.1 空腹血糖

大鼠眼静脉取血，于造模后使用测利得血糖测定仪及配套试纸测定空腹血糖。

(C)1994-2021 China Academic Journal Electronic Publishing House. All rights reserved.　http://www.cnki.net

1.4.2 糖耐量测定

实验第 8 周末各组大鼠灌胃葡萄糖 2.0 g/kg，在 0、0.5、1、2 h 测定血糖值，观察血糖曲线下面积的变化。血糖曲线下面积=（a + 2b + 3c + 2d）/4。

1.4.3 坐骨神经山梨醇

大鼠腹腔注射戊巴比妥 45 mg/kg 进行麻醉，然后将大鼠俯卧固定在固定架上，剖开双侧股部皮肤，暴露出坐骨神经，用无菌剥离器剥离坐骨神经后取出。将坐骨神经组织称定质量，加冷生理盐水，制备成 20% 的匀浆液。取 200 μL 大鼠空白坐骨神经匀浆液，采用高效液相色谱法测定坐骨神经山梨醇水平。

1.4.4 TNF-α 和 IL-1β 测定

各组大鼠麻醉灌注后直接取脊髓 L4-L6 段，置于含蛋白酶抑制剂和磷酸酶抑制剂的裂解液中，将组织匀浆裂解，裂解液在 4℃下，以 12000 r • min^{-1} 离心 5 min，取上清液，BCA 法测定蛋白浓度。根据 TNF-α 和 IL-1β ELISA 试剂盒说明书进行测定。

1.5 统计学方法

采用 SPSS 21.0 分析数据。计量资料用（$\bar{x} \pm s$）表示。两两组间的比较用 LSD-t 检验。以 $P < 0.05$ 为差异有统计学意义。

2 结 果

2.1 大黄素对糖尿病大鼠空腹血糖的影响

大鼠腹腔给药链脲佐菌素 72 h 后，与对照组比较，模型组的空腹血糖明显高于对照组（$P < 0.01$）；而治疗组的空腹血糖和模型组比较差异无统计学意义（$P > 0.05$）。见表 1。

表 1 大黄素对糖尿病大鼠空腹血糖的影响（$\bar{x} \pm s$）

组别	鼠数	空腹血糖（mmol • L^{-1}）
对照组	10	5.93±1.82
模型组	10	20.76±4.31^{aa}
治疗组	10	19.03±6.42

注：与对照组比较，$^{aa}P < 0.01$。

2.2 大黄素对糖尿病大鼠糖耐量的影响

8 周后，与模型组比较，治疗组明显降低糖尿病大鼠各时间点血糖曲线下面积（$P < 0.05$）。见表 2。

表 2 大黄素对糖尿病大鼠血糖和糖耐量的影响（$\bar{x} \pm s$）

组别	鼠数	空腹血糖（mmol • L^{-1}）	餐后 0.5 h 血糖（mmol • L^{-1}）	餐后 1 h 血糖（mmol • L^{-1}）	餐后 2 h 血糖（mmol • L^{-1}）	血糖曲线下面积
对照组	10	5.69±1.06	8.72±0.97	13.49±3.41	7.83±2.21	19.56±4.59
模型组	10	20.91±5.82^{aa}	31.26±4.76^{aa}	30.07±5.20^{aa}	29.97±4.67^{aa}	61.81±6.42^{aa}
治疗组	10	9.87±3.25^{bb}	25.71±4.85^{b}	27.01±5.62	26.73±5.69	54.98±7.52^{b}

注：与对照组比较，$^{aa}P < 0.01$；与模型组比较，$^{b}P < 0.05$，$^{bb}P < 0.01$。

2.3 大黄酸对坐骨神经山梨醇、脊髓 L4-L6 段 TNF-α 和 IL-1β 水平的影响

与对照组大鼠比较，模型组大鼠脊髓 TNF-α 和 IL-1β 表达水平显著升高，坐骨神经山梨醇水平也明显升高（$P < 0.01$）；与模型组比较，治疗组可显著降低糖尿病大鼠脊髓背角 TNF-α 和 IL-1β 的表达水平，也能降低坐骨神经山梨醇水平（$P < 0.01$）。见表 3。

表 3 大黄酸对坐骨神经山梨醇、脊髓 L4-L6 段 TNF-α 和 IL-1β 水平的影响

组别	鼠数	坐骨神经山梨醇（mmol • L^{-1}）	TNF-α（pg • mg^{-1}）	IL-1β（pg • mg^{-1}）
对照组	10	1.38±0.06	75.93±14.04	2.38±0.32
模型组	10	2.37±0.43^{aa}	163.29±24.49^{aa}	4.85±0.93^{aa}
治疗组	10	1.32±0.39^{bb}	109.28±19.38^{bb}	3.41±0.71^{bb}

注：与对照组比较，$^{aa}P < 0.01$；与模型组比较，$^{bb}P < 0.01$。

3 讨 论

糖尿病周围神经病变指的是因糖尿病引发的慢性高血糖状态及其所致的各种病理生理改变导致的神经系统损伤。有报道认为确诊 1 年的糖尿病患者中，神经病变的发生率为 7%，而病程 25 年以上者，发病率达 50%[6]。这种损伤可累及全身周围神经系统任何部分，高血糖引起的糖基化终末产物形成增加 [7]，使得多元醇代谢异常是导致糖尿病周围神经病变的重要原因之一。

现代医学认为，糖尿病神经病变发生发展的关键因素之一就是山梨醇代谢异常 [8]。长期的高血糖状态使多元醇代谢途径被激活，多元醇的代谢有两个关键酶：醛糖还原酶和山梨醇脱氢酶。当葡萄糖进入细胞后被醛糖还原酶还原为山梨醇，然后山梨醇脱氢酶再将其氧化为果糖。神经组织内缺少果糖激酶，则这些过多的果糖不能分解，使山梨醇和果糖聚集，造成神经细胞内渗透压升高，神经细胞水肿、变性、坏死。同时高血糖可以竞争性地抑制神经组织摄取肌醇，使神经组织肌醇水平降低，磷酸肌醇也减少，导致神经传导功能受损。由此可见，蓄积的山梨醇可直接产生神经毒性作用。所以体内山梨醇的水平可以作为考察糖尿病神经病变的一个指标。本实验发现大黄素能改善糖尿病大鼠糖耐量，减轻坐骨神经髓鞘水肿，改善神经病变。因此大黄素对糖尿病大鼠周围经病变具有明显的改善作用，其作用机制可能与改善体内山梨醇代谢有关。

近年研究表明，糖尿病性神经病理性疼痛与脊髓背角小胶质细胞密切相关。受环境影响，脊髓背角小胶质细胞可分泌多种细胞因子和趋化因子，呈现活化态。活化的脊髓背角小胶质细胞可爆发性分泌大量神经毒素和炎症介质，如细胞毒性物质如一氧化氮、氧自由基和炎性因子（如 IL-1β、TNF-α 和 IFN-γ）等 [9]。TNF-α 作为一种致炎细胞因子，在疼痛通路中起着重要的作用，可通过 TNF 受体，传递信号使神经元异位放电并产生痛觉过敏，也可激活核转录因子（NF-κB），促进致炎细胞因子的合成，参与传递调控痛觉信号 [10]。IL-1β

(C)1994-2021 China Academic Journal Electronic Publishing House. All rights reserved. http://www.cnki.net

也是与疼痛关系密切的一种致炎细胞因子，可增强兴奋性突触传递和 / 或降低抑制性突触传递增强痛觉传递[11,12]。实验观察，大黄素可降低与疼痛关系密切的致炎细胞因子 IL-1β 和 TNF-α 水平，提示大黄素抗糖尿病性神经病理性疼痛可能与其抑制脊髓背角小胶质细胞活化有关。综上所述，大黄素可以作为一种治疗或辅助治疗糖尿病及其周围神经病变的药物，其作用机制可能是通过降低致炎细胞因子水平或改善体内山梨醇代谢发挥作用，也可能与其降血糖有关，本研究为大黄素的开发利用提供了参考。

参考文献：

[1]刘硒碲,夏宁.糖尿病周围神经病变研究进展[J].广西医科大学学报,2010,27(02):317-319.

[2]率红莉,汪津洋.糖尿病周围神经病变治疗药物研究进展[J].中国药房,2014,25(04):377-382.

[3]DONG X,FU J,YIN X, *et al.* Emodin: A Review of its Pharmacology, Toxicity and Pharmacokinetics[J].Phytother Res,2016,30(08):1207-1218.

[4]WEI WT,LIN SZ,LIU DL, *et al.* The distinct mechanisms of the antitumor activity of emodin in different types of cancer (Review)[J].Oncol Rep,2013,30(06):2555-2562.

[5]Shrimali D,Shanmugam MK,Kumar AP, *et al.* Targeted abrogation of diverse signal transduction cascades by emodin for the treatment of inflammatory disorders and cancer[J].Cancer Lett,2013,341(02):139-149.

[6]郑方道.糖尿病神经病变[J].开卷有益:求医问药,1999(04):9.

[7]吴道爱,徐浣白,汪运生.糖尿病亚临床周围神经病变与病程、糖基化血红蛋白的关系[J].蚌医学院学报,2010,35(08):782-783.

[8]游建明,纪云西,黄贵华,等.糖尿病周围神经病变病机演变初探[J].新中医,2011,43(03):1-2.

[9]尹丽鹤,赵钢.小胶质细胞的活化与细胞因子的研究进展[J].神经损伤与功能重建,2008,3(06):422-424.

[10]林健清,张志坚,林财珠.神经病理性疼痛与细胞因子IL、TNF-α、NGF的研究进展[J].临床麻醉学杂志,2009,25(04):363-365.

[11]钟敏,曾因明,柳垂亮,等.脊髓TNF-α、IL-1β和IL-6在神经病理性疼痛过程中的表达变化[J].中国疼痛医学杂志,2010,16(01):42-44.

[12]谢人明,范引科,张红,等.强筋健骨胶囊对急性痛风模型炎症因子的影响[J].西北药学杂志,2013,28(02):165-167.

编辑：弓良彦 编号：EA-4180518154（修回：2019-03-06）

探讨黄连阿胶汤与朱雀汤的关系

An analysis of the relationship between the Huanglian E'jiao decoction and the Zhuque decoction

陈芒华[1] 谢俊宇[2] 卢 敏[3]

（1. 广西中医药大学附属瑞康医院，广西 南宁，530000；2. 广西中医药大学，广西 南宁，530000；3. 广西国际壮医医院，广西 南宁，530011）

中图分类号：R222.16 文献标识码：A 文章编号：1674-7860（2019）07-0026-03

【摘 要】《伤寒杂病论》以四方神兽命名的方剂有青龙汤、白虎汤，大量文献分析已证明玄武汤即真武汤，唯缺朱雀汤，历代医家多赞同确有此方，然其究竟为何方则莫衷一是。根据文献和对朱雀汤及黄连阿胶汤的分析，讨论两方之间的关系。

【关键词】黄连阿胶汤；《伤寒杂病论》；四神汤；朱雀汤

【Abstract】The prescriptions of *Shanghan Zabing Lun* (《伤寒论》) named with four god beasts have the Qinglong decoction (青龙汤), the Baihu decoction (白虎汤), and the Xuanwu decoction (玄武汤) which has been proved is the Zhenwu decoction (真武汤) by a large number of literature; just lack of the Zhuque decoction (朱雀汤). Lots of doctors agree it must be included in *Shanghan Zabing Lun*, but no one knows what is it. On the basis of literature and analyzing of mechanism of the Huanglian E'jiao decoction, discuss the relationship between the Huanglian E'jiao decoction and the Zhuque decoction.

【Keywords】The Huanglian E'jiao decoction; *Shanghan Zabing Lun*; The Sishen decoction (四神汤); The Zhuque decoction

doi:10.3969/j.issn.1674-7860.2019.07.009

以中医基础理论为指导，从中医与四方、四象、五行、八卦的联系中论述《伤寒杂病论》中青龙汤、白虎汤、真武汤（玄武汤）的药理方义，讨论朱雀汤和黄连阿胶汤的联系。

四神汤即以四方神兽命名之方剂，分别为青龙、白虎、朱雀、玄武，既是四神兽，亦代表四方，青龙为东方之神，白虎为西方之神，玄武（龟、蛇）为北方之神，朱雀为南方之神，四兽各执一方，其中青龙汤、白虎汤原方首载于《伤寒杂病论》，玄武汤经后世医家论证并认定即为《伤寒杂病

(C)1994-2021 China Academic Journal Electronic Publishing House. All rights reserved. http://www.cnki.net
