# Supplementary material for: Emodin, a rising star in the treatment of glycolipid metabolism disorders: a preclinical systematic review and meta-analysis
Source: PeerJ. 2025 May 15;13:e19221. doi: 10.7717/peerj.19221 (PMC12085882; doi:10.7717/peerj.19221)
Supplement: Supplemental Information 19 [file peerj-13-19221-s019.zip › 中文纳入的文献/大黄素改善肥胖型2型糖尿病小鼠糖脂代谢的实验研究_周丽嫦.pdf]

DOI:10.13863/j.issn1001-4454.2012.09.048

剧烈运动后肌肉中过多乳酸的清除代谢，从而达到延缓疲劳或加速疲劳的消除的作用。

糖是肌肉组织的重要能量来源，体内的糖储备包括肌糖原、肝糖原和血糖三类。有研究表明，运动导致的体力衰竭总是和肌糖原的耗竭同时发生的，随着肌糖原消耗的不断增加，机体为维持血糖水平，将动用肝糖原而导致肝糖原减少[6]。因此，肝糖原和肌糖原含量是反映疲劳程度的敏感指标。实验结果显示，荔枝多糖能够维持运动后小鼠的肝糖原和肌糖原含量，从而为机体提供较好的能量储备。另外，糖原贮备的提高有利于机体耐力速度的提高，并减少蛋白质和含氮化合物的分解代谢，降低血 BUN 的形成。血 BUN 是蛋白质的代谢产物，一般运动时间不超过 30 min 时，蛋白质较少参与供能，BUN 变化不明显。当机体长时间运动不能通过糖、脂肪分解代谢获得足够的能量时，蛋白质和氨基酸分解代谢才加强，BUN 会明显增加，且 BUN 含量随运动负荷的增加而增加，身体对负荷的适应性越差，则产生的 BUN 越多[7]。由此可见，BUN 与机体运动适应能力、疲劳程度以及负荷量的大小密切相关，因此可用来作为评定疲劳的指标。荔枝多糖能使游泳运动后休息 60 min 时小鼠的血 BUN 显著低于模型组，表明荔枝多糖能使小鼠耐力增加，对负荷的适应性增强，延缓疲劳的产生。

综上所述，荔枝多糖能够提高小鼠的运动能力，增加肝糖原储备以及减少运动后尿素氮和乳酸的蓄积，从而具有良好的抗疲劳作用，有关其具体的抗疲劳机制有待进一步研究。

参 考 文 献

[1] 陈奇. 中药药理研究方法学 [M]. 北京: 人民卫生出版社，1996. 780-781.

[2] 郭敏，陈靠山. 牛蒡低聚果糖对小鼠抗疲劳作用的研究 [J]. 天然产物研究与开发，2007，(4)：642-644.

[3] 昌友权，郑鸿雁，曲红光，等. 柳黄酮抗疲劳作用的实验研究 [J]. 食品科学，2006，27(8)：251-253.

[4] 赵敬国. 肌肉疲劳的细胞学机制及应对措施 [J]. 山东体育科技，2001，23(1)：26-29.

[5] Xu GG，Luo QM. A study on the relationship between blood acid lactate in motion and hemoglobin saturation density-a new hemoglobinmetry [J]. *Wuhan Institute Physi Edu*，2001，35(3)：40-42.

[6] 王曦，许晓燕，郑林用，等. 乳酸菌发酵黄芪的抗疲劳保健功能研究 [J]. 时珍国医国药，2011，22(6)：1422-1424.

[7] 汪海涛，嵇扬，徐永祥，等. 枳椇子乙醇提取物抗疲劳作用的实验研究 [J]. 解放军药学学报，2008，24(2)：121-123.

大黄素改善肥胖型2型糖尿病小鼠糖脂代谢的实验研究

周丽嫦，徐艳燕，陈伟标，曾婉君，王叶茗，欧阳勇
(广州市中西医结合医院药剂科，广东 广州 510800)

摘要 目的：探索大黄素对肥胖型2型糖尿病小鼠糖、脂代谢的影响。方法：雄性肥胖型2型糖尿病小鼠(diabetes mouse，db/db 小鼠) 18 只，分大黄素高剂量(100 mg/kg)干预组，大黄素低剂量(25 mg/kg)干预组及普通饮食对照组，喂养过程中每周监测体质量、血压、血脂、空腹血糖等，共干预 12 w，于等 12 周行胰岛素耐量实验，实验结束后空腹取血测血脂、GLP-1、糖化血红蛋白水平。结果：大黄素能防止 db/db 小鼠的体质量、空腹血糖的上升，与对照组比较，$P<0.05$；降低血脂水平及糖化血红蛋白水平，增加 GLP-1 的释放，但对血压无显著的影响。结论：大黄素可显著改善肥胖型2型糖尿病小鼠的糖脂代谢。

关键词 大黄素；2型糖尿病；db/db 小鼠；糖代谢；脂代谢
中图分类号：R285.5 **文献标识码**：A **文章编号**：1001-4454(2012)09-1487-04

糖尿病是严重危害人民健康的重大疾病，是心脑血管疾病、截肢的重要原因。目前全球已有超过 3 亿的糖尿病患者，我国糖尿病患者也接近 1 亿，居世界的首位[1,2]。目前的糖尿病防治方案并不能有

收稿日期：2012-01-09
作者简介：周丽嫦(1970-)，女，主管中药师，主要从事中药药理研究。

(C)1994-2022 China Academic Journal Electronic Publishing House. All rights reserved. http://www.cnki.net

效降低糖尿病的发生率，故积极寻求行之有效的治疗方案有重要的现实意义。我国是中医药大国，中医药的应用有几千年的历史，对于糖尿病的治疗积累了丰富的经验。近年来关于中草药活性成分防治糖尿病的研究方兴未艾，取得了一些突破性的进展。例如黄连素可促进胰岛素及 GLP-1 分泌改善糖代谢[3]，肉桂醛可以防治糖尿病肾脏损害等[4]。笔者及他人的前期研究表明，大黄素具有降低血脂，防止糖尿病大鼠肾脏纤维化，体外实验具有促进肠道降糖激素分泌的作用，但大黄素对 2 型糖尿病模型影响的系统整体的实验研究并未见相关的报道。为此，在本研究中笔者采用目前公认的肥胖型 2 型糖尿病小鼠（db/db 小鼠）探索大黄素对肥胖型 2 型糖尿病动物模型的糖、脂代谢的影响，现报道如下。

1 材料与仪器

1.1 实验动物 8 周龄雄性肥胖型 2 型糖尿病小鼠（db/db 小鼠）18 只，8 周龄雄性 C57BL/KsJ 小鼠 6 只，均购自南京大学模式实验中心。

1.2 药物与试剂 大黄素；TG（甘油三酯）、TC（总胆固醇）、FFA（游离脂肪酸）ELISA 试剂盒购于深圳晶美公司；乌拉坦（国药集团化学试剂有限公司）；用灭菌去离子水配制 10% 的乌拉坦溶液。

1.3 主要仪器 鼠尾血压测量仪（Powerlab2/25 澳大利亚）；强生稳豪血糖仪（美国强生公司）。

2 方法

2.1 动物分组及给药 动物分为正常对照组、db/db 小鼠模型对照组及大黄素高、低剂量组，每组 6 只。大黄素高剂量组，在给予正常饮食的基础上每天以 100 mg/kg 的生理盐水大黄素溶液灌胃；大黄素低剂量组在给予普通饲料喂养的基础上，每天给予 25 mg/kg 的生理盐水大黄素溶液灌胃；正常对照组的 C57BL/KsJ 小鼠给予普通饲料喂养，给予前两组等体积的生理盐水灌胃，1 次/d；db/db 对照组同样给予普通饲料喂养，每天给予等体积的生理盐水灌胃；所有小鼠自由饮水进食。自然昼夜采光，室温 22 ~ 24 ℃，每日早晚喂食、换水，观察时间为 12 w。

2.2 观察指标及检测方法 应用无创方法，通过鼠尾血压测量仪（Powerlab2/25 澳大利亚）测定小鼠清醒、安静状态下鼠尾收缩压，每 2 周测定 1 次；以便携式血糖计每 2 周进行一次空腹血糖检测；干预结束后，小鼠以 10% 乌拉坦麻醉后于颈动脉取血，离心后取血浆于 −20 ℃ 下保存备用；TG、TC、FFA 等以 ELISA 法进行检测，方法按说明书进行。

2.3 统计学处理 实验数据以 $\bar{x} \pm s$ 表示，用 Students′t 检验比较组间差异的显著性；多组间比较采用 One-way ANOVA 检验，以 $P < 0.05$ 为有统计学差异。

3 结果

3.1 大黄素对 db/db 小鼠体质量的影响 db/db 小鼠体质量显著高于正常对照组的 C57BL/KsJ 小鼠（$P < 0.01$）；干预前，各组 db/db 小鼠的体质量无显著性差异（$P > 0.05$）；高剂量的大黄素可显著抑制 db/db 小鼠体质量的增长速度，至第 10 周及第 12 周，高剂量组与模型组差异显著（$P < 0.05$，$P < 0.01$）；而大黄素低剂量组虽亦有抑制 db/db 小鼠体质量上升的趋势，但差异不显著（$P > 0.05$）。见表 1。

表 1 大黄素对 db/db 小鼠体质量的影响（g，$\bar{x} \pm s$，$n = 6$）

组别	0 周	2 周	4 周	6 周	8 周	10 周	12 周
正常对照组	18.7 ± 1.1	19.3 ± 1.0	20.4 ± 1.2	21.3 ± 1.1	22.4 ± 1.2	24.4 ± 1.3	26.7 ± 1.4
模型组	33.8 ± 2.2##	35.1 ± 2.8	38.6 ± 2.6	40.5 ± 3.3	42.8 ± 3.3	45.7 ± 2.9	50.8 ± 4.2
大黄素高剂量组	33.3 ± 2.1##	32.8 ± 2.9	35.3 ± 3.0	37.3 ± 2.7	39.3 ± 3.2	41.3 ± 2.5*	42.8 ± 2.9**
大黄素低剂量组	33.7 ± 2.3##	34.6 ± 2.7	37.2 ± 2.9	38.8 ± 3.0	41.1 ± 3.1	43.4 ± 3.1	46.5 ± 3.8

注：与正常对照组比较，##$P < 0.01$；与模型组比较，*$P < 0.05$，**$P < 0.01$

3.2 大黄素对 db/db 小鼠空腹血糖的影响 与 C57BL/KsJ 小鼠比较，db/db 小鼠出生后 6 w 即可出现明显的肥胖和空腹血糖增加（$P < 0.01$）；干预前，各组 db/db 小鼠的空腹血糖无显著性差异（$P > 0.05$）；大黄素的预防性干预可显著抑制 db/db 小鼠空腹血糖的上升，在干预 8 w 后，大黄素高剂量组小鼠与 db/db 对照组小鼠差异显著（$P < 0.05$）。见表 2。

3.3 大黄素对 db/db 小鼠血压的影响 肥胖与血压高度相关，笔者在本研究中同时探讨了大黄素对肥胖小鼠血压的影响。但以上 2 种剂量的大黄素对 db/db 小鼠的血压无显著性影响。此外，db/db 小鼠的血压与正常对照小鼠的血压也无显著性差异（$P > 0.05$）。见表 3。

(C)1994-2022 China Academic Journal Electronic Publishing House. All rights reserved. http://www.cnki.net

表2 大黄素对db/db小鼠空腹血糖的影响(mmol/L,$\bar{x}\pm s$,n=6)

组别	0周	2周	4周	6周	8周	10周	12周
正常对照组	4.5±0.6	5.1±0.5	5.3±0.9	5.5±0.6	5.9±0.7	5.6±0.4	5.9±0.9
模型组	9.0±1.8##	10.4±1.3	12.3±1.1	11.4±2.1	12.9±2.1	13.4±2.1	16.6±3.1
大黄素高剂量组	9.7±3.6##	10.8±1.0	12.8±1.6	11.0±1.7	10.4±1.5*	11.1±1.2*	12.7±1.8*
大黄素低剂量组	9.5±2.3##	10.7±1.7	11.9±2.1	12.1±2.3	12.0±1.9	12.8±2.7	14.6±2.9

注:与正常对照组比较,##$P<0.01$;与模型组比较,*$P<0.05$

表3 大黄素对db/db小鼠血压的影响(mmHg,$\bar{x}\pm s$,n=6)

组别	0周	2周	4周	6周	8周	10周	12周
正常对照组	113±8	110±6	116±7	119±10	122±9	125±13	123±10
模型组	116±6	115±5	118±8	123±11	117±10	119±11	118±8
大黄素高剂量组	114±9	117±8	119±9	120±8	124±12	123±10	119±9
大黄素低剂量组	112±7	114±7	115±6	121±9	121±11	116±9	122±11

3.4 大黄素对db/db小鼠血脂水平的影响 与正常对照小鼠比较,db/db小鼠血浆中的TG、TC、FFA水平显著升高($P<0.05$)。高剂量大黄素的预防性干预可有效控制db/db小鼠血浆TG、TC、水平的升高($P<0.05$),低剂量大黄素虽有控制血脂上升的趋势,但在干预12周内作用并不显著($P>0.05$)。见表4。

表4 大黄素对db/db小鼠血脂水平的影响($\bar{x}\pm s$,n=6)

组别	TG/(mmol/L)	TC/(mmol/L)	FFA/(mmol/L)
正常对照组	1.05±0.14	3.37±0.58	1.12±0.13
模型组	1.51±0.17##	4.57±0.67	1.23±0.16
大黄素高剂量组	1.16±0.11**	3.89±0.31*	1.14±0.12
大黄素低剂量组	1.32±0.14	4.32±0.54	1.18±0.14

注:与正常对照组比较,##$P<0.01$;与模型组比较,*$P<0.05$,**$P<0.01$

3.5 大黄素对db/db小鼠胰岛素敏感性的影响 db/db小鼠存在明显的胰岛素抵抗,与正常对照组比较差异显著($P<0.01$);与db/db对照组比较,大黄素可有效改善db/db小鼠胰岛素的敏感性($P<0.05$)。见图1。

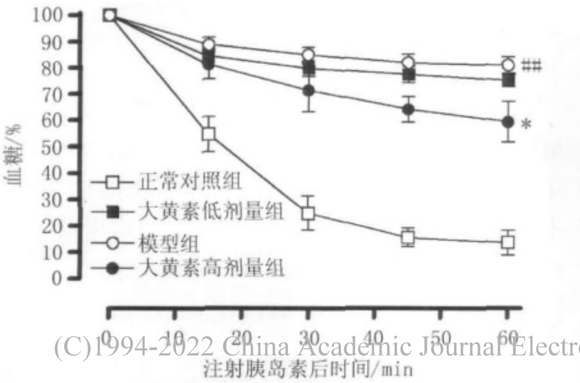

图1 大黄素对db/db小鼠胰岛素敏感性的影响

注:与正常对照组比较,##$P<0.01$;与模型组比较,*$P<0.05$

4 讨论

目前的研究证实大黄素具有较好的改善糖代谢及脂质代谢的作用,但未见有关于大黄素对肥胖性2型糖尿病小鼠模型的影响的系统研究,机制也不清。笔者的实验研究表明,大黄素的干预可显著防止肥胖型2型糖尿病小鼠的体质量、空腹血糖、血脂水平的升高,促进GLP-1的分泌,但对糖尿病小鼠的血压并无显著的影响。

db/db小鼠由C57BL/KsJ近亲交配株常染色体隐性遗传衍化而来,由于leptin基因受体缺陷而引起体内糖脂代谢紊乱,与人2型糖尿病发生相似,是目前公认的用于研究2型糖尿病的小鼠模型。动物在一个月时开始贪食及发胖,继而出现显著的胰岛素抵抗,产生高血糖、高血胰岛素,胰高血糖素也升高,而GLP-1分泌显著减少。该类型小鼠一般在10个月内死亡。大黄素在大黄、虎杖等中药中含量丰富,目前研究证实具有良好的抗微生物,抗炎,抗氧化、清除自由基等活性,具有保肝、抗肿瘤、抗肾脏纤维化、抗缺血-再灌注损伤等作用[5]。笔者的前期研究发现,大黄素体外可促进STC-1细胞分泌GLP-1,体内急性实验表明可促进GLP-1的分泌。GLP-1是肠道,尤其是远端回肠分泌的一种降糖激素,具有抑制胰岛β细胞凋亡,恢复胰岛β细胞的生理功能,促进胰岛素分泌,降低血糖作用,还能扩张血管,降低血压,抑制心肌重构。但体内GLP-1很快被DPP4降解,半衰期很短,只有5 min左右,目前已利用基因重组技术合成了GLP-1类似物,用于治疗糖尿病,尤其是2型糖尿病患者[6]。GLP-1还具有改善外周胰岛素抵抗,减肥和无低血糖反应的优点[7],故针对GLP-1这个特点近年来研究者们着重研究如何促进内源性的GLP-1分泌,从而改善2型糖尿病患者的糖脂代谢。目前已取得一些令人惊喜的研

(C)1994-2022 China Academic Journal Electronic Publishing House. All rights reserved. http://www.cnki.net

究成果，例如，Daoudi 等[8]发现 PPARβ 激动剂可促进内源性 GLP-1 分泌；YU 等[3]证实黄连素可通过 PKC 信号通路促进内源性 GLP-1 的分泌；Thomas 等[9]发现一种 G 蛋白偶联受体 TGR5 可促进 GLP-1 分泌从而控制葡萄糖稳态。

本试验结果表明大黄素可改善肥胖性 2 型糖尿病小鼠的胰岛素抵抗，降低体质量，防止空腹血糖的升高及降低血脂水平，这可能与大黄素促进内源性 GLP-1 的分泌增加有关。大黄素对于肥胖性 2 型糖尿病有较好的疗效，值得进一步开发。

参 考 文 献

[1] Rathmann W, Giani G. Global prevalence of diabetes: estimates for the year 2000 and projections for 2030 [J]. *Diabetes Care*, 2004, 27: 2568-2569; author reply 2569.

[2] Shaw JE, Sicree RA, Zimmet PZ. Global estimates of the prevalence of diabetes for 2010 and 2030 [J]. *Diabetes Res Clin Pract*, 2010, 87(1): 4-14.

[3] Yu Y, Liu L, Wang X, *et al*. Modulation of glucagon-like peptide-1 release by berberine: *in vivo* and *in vitro* studies [J]. *Biochem Pharmacol*, 2010, 79: 1000-1006.

[4] Subash Babu P, Prabuseenivasan S, Ignacimuthu S. Cinnamaldehyde-a potential antidiabetic agent [J]. *Phytomedicine*, 2007, 14: 15-22.

[5] LIU Han, GAO Yun. Research progress in molecular mechanism of the pharmacological actions of emodin [J]. *Chinese Pharmacological Bulletin*, 2009, 25(12): 1552-1555.

[6] Buteau J. GLP-1 receptor signaling: effects on pancreatic *beta*-cell proliferation and survival [J]. *Diabetes Metab*, 2008, 34 Suppl 2: S73-77.

[7] Gupta R, Walunj SS, Tokala RK, *et al*. Emerging drug candidates of dipeptidyl peptidase IV (DPP IV) inhibitor class for the treatment of Type 2 Diabetes [J]. *Curr Drug Targets*, 2009, 10: 71-87.

[8] Z Daoudi M, Hennuyer N, Borland MG, *et al*. PPAR*beta/delta* activation induces enteroendocrine L cell GLP-1 production [J]. *Gastroenterology*, 2011, 140: 1564-1574.

[9] Thomas C, Gioiello A, Noriega L, *et al*. TGR5-mediated bile acid sensing controls glucose homeostasis [J]. *Cell Metab*, 2009, 10: 167-177.

左旋虾青素对四氯化碳致小鼠急性肝损伤保护作用的研究

罗文基[1]，黄丽娟[1]，陈逸生[1]，张坤水[2*]

（1. 中山大学附属第五医院，广东 珠海 519000；2. 中山大学孙逸仙纪念医院药学部，广东 广州 510120）

摘要 目的：探讨左旋虾青素对四氯化碳致小鼠急性肝损伤的保护作用。方法：通过皮下注射四氯化碳（CCl_4）诱导小鼠急性肝损伤，检测小鼠血清中丙氨酸氨基转移酶（ALT）、天门冬氨酸氨基转移酶（AST）的含量、肝脏中超氧化物歧化酶（SOD）的活性以及丙二醛（MDA）的含量，考察左旋虾青素的保肝作用。结果：左旋虾青素能显著降低小鼠血清中 ALT、AST 的活性与肝脏中 MDA 含量，提高小鼠肝脏的 SOD 活性。结论：左旋虾青素对 CCl_4 所致肝脏毒性具有一定保护作用。

关键词 虾青素；四氯化碳；氨基转移酶；超氧化物歧化酶；丙二醛

中图分类号：R285.5 **文献标识码**：A **文章编号**：1001-4454(2012)09-1490-03

左旋虾青素（Astaxanthin）是一种红色的胡萝卜素，主要来自雨生红球藻，分子式为 $C_{40}H_{52}O_4$，经证实有极强的抗氧化作用，也具较强的清除自由基能力。据报道[1]，左旋虾青素的抗氧化活性是维生素 E 的 550 倍，是 B_2 胡萝卜素、玉米黄素、叶黄素和角黄素等类胡萝卜素的 10 倍，被誉为“超级维生素 E”、“超级抗氧化剂”。虾青素具有 3 种异构形态（左旋、右旋、消旋），近年来研究证实左旋虾青素有最强的抗氧化作用，能够清除自由基，具有与 SOD 相似的作用。本文考察了左旋虾青素对四氯化碳致小鼠急性肝损伤的保护作用，现将结果报道于下。

1 实验材料

1.1 实验动物 SPF 级昆明小鼠，体质量 20～25 g，雌雄各半，购自广东省医学实验中心，许可证号：

收稿日期：2012-04-10

作者简介：罗文基（1973-），男，主管药师，主要从事医院药学研究；Tel：0756-2528278，E-mail：luowenji2005@21cn.com。

* 通讯作者：张坤水，Tel 020-84113095，E-mail：pnhunter@sina.com。

(C)1994-2022 China Academic Journal Electronic Publishing House. All rights reserved. http://www.cnki.net
