# Supplementary material for: Emodin, a rising star in the treatment of glycolipid metabolism disorders: a preclinical systematic review and meta-analysis
Source: PeerJ. 2025 May 15;13:e19221. doi: 10.7717/peerj.19221 (PMC12085882; doi:10.7717/peerj.19221)
Supplement: Supplemental Information 20 [file peerj-13-19221-s020.zip › 8篇英文文献/Aditya Arvindekar2015.pdf]

View Article Online
View Journal

Food & Function

Accepted Manuscript

This article can be cited before page numbers have been issued, to do this please use: A. Arvindekar, T. More, P. V. Payghan, K. Laddha, N. Ghoshal and A. U. Arvindekar, *Food Funct.*, 2015, DOI: 10.1039/C5FO00519A.

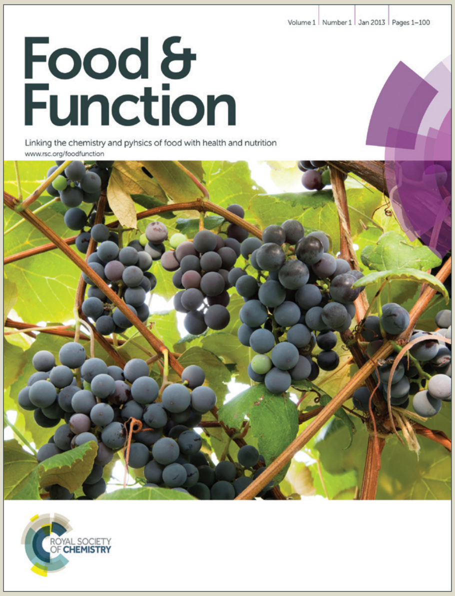

This is an *Accepted Manuscript*, which has been through the Royal Society of Chemistry peer review process and has been accepted for publication.

Accepted Manuscripts are published online shortly after acceptance, before technical editing, formatting and proof reading. Using this free service, authors can make their results available to the community, in citable form, before we publish the edited article. We will replace this *Accepted Manuscript* with the edited and formatted *Advance Article* as soon as it is available.

You can find more information about *Accepted Manuscripts* in the Information for Authors.

Please note that technical editing may introduce minor changes to the text and/or graphics, which may alter content. The journal's standard Terms & Conditions and the Ethical guidelines still apply. In no event shall the Royal Society of Chemistry be held responsible for any errors or omissions in this *Accepted Manuscript* or any consequences arising from the use of any information it contains.

ROYAL SOCIETY OF CHEMISTRY

www.rsc.org/foodfunction

Please do not adjust margins

View Article Online
DOI: 10.1039/C5FO00519A

Journal Name

ARTICLE

Evaluation of anti-diabetic and alpha glucosidase inhibitory action of anthraquinones from *Rheum emodi*

Received 00th January 20xx,
Accepted 00th January 20xx

DOI: 10.1039/x0xx00000x

www.rsc.org/

Aditya Arvindekar[a], Tanaji More[b], Panan Payghan[c], Kirti Laddha[a], Nanda Ghoshal[c] and Akalpita Arvindekar[b,*]

Rheum emodi is used as a culinary plant across the world and finds an eminent role in the Ayurvedic and traditional Chinese system of medicine. The plant is known to principally contain 1,8-dihydroxyanthraquinones (DHAQs) like rhein, aloe emodin, emodin, chrysophanol and physcion that possess diverse pharmacological and therapeutic actions. The present work deals with developing a platform technology for isolation of these DHAQs and evaluating their anti-diabetic potential. Herein, we report the anti-hyperglycemic activity and alpha glucosidase (AG) inhibitory actions of five isolated DHAQs from *R. emodi*. All the five isolated DHAQs showed good anti-hyperglycemic activity with aloe emodin exhibiting maximum lowering of blood glucose in oral glucose tolerance test. However, on evaluation of the AG inhibitory potential of the DHAQs only emodin exhibited potent intestinal AG inhibition (93 ± 2.16%) with an IC_{50} notably lower than acarbose. Subsequent kinetic studies indicated a mixed type of inhibition for emodin. *In-vivo* studies using oral maltose load showed almost total inhibition foe emodin when compared to acarbose. Molecular docking studies revealed the presence of an allosteric topographically distinct 'Quinone binding site' and showed that interaction with Ser 74 occurs exclusively with emodin, which is vital for AG inhibition. The net benefit from glucose lowering effect and mixed type inhibition by emodin would enable requirement of small dosage that is safe and non toxic in case of prolonged use in treating diabetes.

Introduction

Rheum emodi (Rhubarb; family: Polygonaceae) constitutes an important food source and is consumed across the world in form of Rhubarb pie, tarts and crumbles. It is also used in preparation of fruit wine and many baked food preparations. The plant is well-known in the traditional Chinese medicine and finds a mention in several other systems of medicine. Rhubarb has been used as a purgative, astringent tonic and in quick healing of ulcers. It is also well known for its laxative, anti-bacterial, haemostatic, anti-spasmodic and anti-carcinogenic activity in traditional Chinese medicine and also the Indian Ayurvedic system of medicine.[1] Li et al. (2004) observed Rhubarb to be one of the 22 medicines that often appear in the Chinese traditional prescriptions for clinical treatment of diabetes and its complications.[2] Hence it is intended in the present study to evaluate the anti-diabetic potential of the culinary plant with an attempt to understand the underlying mechanisms.

A major chemical class of phenolic compounds from *Rheum emodi* constitute the 1,8-dihydroxyanthraquinones (DHAQs) viz. rhein, aloe-emodin, emodin, chrysophanol and physcion (Fig. 1). These occur in the free form (aglycones) and as O-glycosides.[1] The total anthraquinone content in *R. emodi* is known to be about 4.5% w/w of which the free anthraquinone aglycones constitute 1.9% w/w and the rest by anthraquinone glycosides in the dried drug.[3] The anthraquinone class of compounds exhibit diverse pharmacological effects like anti inflammatory, anti-viral and anti-cancer properties.[4-6] As *R. emodi* was often prescribed for clinical treatment of diabetes in traditional Chinese medicine which contains DHAQs as major chemical class of phenolic compounds; the aim was to isolate these pharmacologically important compounds and study their anti-diabetic potential.

Literature reveals various methods of extraction using sophisticated analytical methodologies for extraction of the bioactive DHAQs from *R. emodi* like ionic liquid / salt aqueous two-phase system, ionic liquid-based ultrasonic / microwave assisted extraction, thin-layer chromatography, counter-current chromatography and capillary electrophoresis.[7-10] However, these methods of extraction are laborious, time consuming, offer low yield and are not suitable for commercial applications.

The escalating number of diabetics in the world needs development of newer and effective therapy options. The current therapy panacea recommends usage of combinatorial treatment consisting of a secretagogue or an insulin sensitizer with an alpha glucosidase inhibitor (AGI). The latter plays a major role in preventing postprandial glucose spikes and hence augments the action of the former agents to help maintain a good glycemic regime. AGIs are widely studied and isolated from different sources such as plants and microbes. Currently, acarbose is commonly used AGI. However, being a competitive inhibitor it is needed in large quantity and known to cause intestinal disturbances and discomfort.[11]

a. Medicinal Natural Products Research Laboratory, Department of Pharmaceutical Sciences & Technology, Institute of Chemical Technology, Mumbai, India.
b. Department of Biochemistry, Shivaji University, Kolhapur, India.
c. Structural Biology and Bioinformatics Division, CSIR-Indian Institute of Chemical Biology, Kolkata, India.
Electronic Supplementary Information (ESI) available.
See DOI: 10.1039/x0xx00000x

Published on 18 June 2015. Downloaded by University of Connecticut on 21/06/2015 10:55:03.

Food & Function Accepted Manuscript

Published on 18 June 2015. Downloaded by University of Connecticut on 21/06/2015 10:55:03.

View Article Online
DOI: 10.1039/C5FO00169B

Food & Function Accepted Manuscript

The initial part of our work aimed at developing a platform technology for isolation of the bio-active DHAQs and identifying their anti-diabetic potential. Five isolated DHAQs were estimated for their anti-hyperglycemic activity and also evaluated for AG inhibitory action by *in-vitro*, *in-vivo* and kinetic studies. The putative mechanism of DHAQs at the molecular level was revealed by molecular recognition of DHAQs at the intestinal AG, which was studied using an *in-silico* approach. The binding site identification was done in a systematic manner. Binding mode analysis was carried out using docking studies for the isolated DHAQs.

Materials and methods

Materials

The dried rhizomes of *R. emodi* Wall. ex Meissn were obtained from M/s. Yucca Enterprises, Mumbai, India and authenticated by Prof. H. M. Pandit, Khalsa College, Mumbai; a voucher specimen was deposited in Medicinal Natural Products Research Laboratory, Institute of Chemical Technology, Mumbai (voucher code: ICT/MNPRL/2014/RE/01). All chemicals used were of AR grade, unless specified, and were obtained from S. D. Fine Chemicals Limited, Mumbai. HPLC grade Methanol was sourced from Merck (India). Water for HPLC was made in-house after filtration through 0.45 µm filter. The rhizomes of *R. emodi* were powdered and used for extraction purpose.

TLC was performed on 0.20 mm silica gel G60 F_{254} (E. Merck) aluminum-supported plates. Compounds were visualized either under UV light at 254 nm or with Borntrager's reagent. IR spectra were recorded on a JASCO FT/IR-5300 infrared spectrophotometer. Mass spectra were recorded on Micromass Q-TOF MS Mass Spectrometer. ^{1}H-NMR spectra were recorded on a JOEL 400-MHz instrument with an internal standard of tetramethylsilane (Me_4Si). Reagent grade solvents were used for extraction and isolation; HPLC grade solvents were employed for chromatographic analysis. HPLC analysis was performed with a Jasco (Hachioji, Tokyo, Japan) system, using a 250 mm × 4.6 mm i.d., RP-18 (5µm particle size) column and mobile phase comprising of methanol: water containing 0.1% *ortho*-phosphoric acid (85:15) at UV detector wavelength 254 nm.

Isolation of DHAQs from *R. emodi*

The isolation of DHAQs from *R. emodi* was carried out with certain modifications in the process mentioned by Zhang and Lu (2004); considering a larger scale of operation and shown in flowchart (Figure 1).[12] At every stage TLC was done for preliminary identification of isolated compounds, the mobile phase for which consisted of light petroleum: ethyl acetate: formic acid (75:25:1). TLC analysis confirmed the presence of rhein, aloe emodin, emodin, chrysophanol and physcion with R_f values of 0.15, 0.25, 0.4, 0.6, 0.75 respectively. Compounds were confirmed by determination of melting point and spectral analysis including IR spectroscopy, Mass spectrometry and ^{1}H-NMR spectroscopy (results presented in supplementary data). HPLC analysis was done to ascertain the purity of the isolated DHAQs.

Experimental animals

Male Wistar albino rats weighing between 180–200 g were used. The experimental protocol used was approved by the Animal Ethics Committee (Registration No. 233/CPCSEA). Animals were provided with standard rat diet purchased from Amruth India and water *ad-libitum*. Diabetes was induced by single intraperitoneal injection of streptozotocin (70 mg in 0.1 M citrate buffer, pH 4.5 prepared freshly). Rats showing blood glucose above 200 mg/dl after 14 days of induction were considered diabetic and used in the experimentation. Results presented are average values of experiments conducted on set of three rats.

Oral glucose tolerance test

The animals were categorized in three sets: normal, diabetic control and drug treated ones; each set containing three rats. The rats were subjected to oral glucose tolerance test (OGTT) by feeding them with glucose at a dose of 3 mg/g body weight. Chrysophanol, physcion, emodin, rhein and aloe-emodin were administered to diabetic rats at a dose of 2 mg/kg body weight with intragastric tube. Blood was withdrawn from tail vein at defined time intervals (0, 30, 60 and 120 min) and blood glucose measured using Accu-check™ glucometer (Roche, Germany).

Preparation of rat intestinal AG

All procedures for preparation of rat intestinal AG were performed at 4^{0}C. The small intestinal mucosal tissue was collected by scraping the luminal surface firmly with a spatula. The mucosal scrapings were homogenized with 0.2 M sodium phosphate buffer pH 7.0 containing 1% triton-X 100, and then centrifuged at 12000 rpm for 15 min. The supernatant fraction contained rat small intestinal AG. Butanol was added to the supernatant fraction in 1: 1 proportion and centrifuged at 15000 rpm for 15 min. The aqueous layer was dialyzed overnight against the same buffer. After dialysis, the concentrated enzyme was stored at 4^{0}C. This crude enzyme was used in the study to observe inhibition by different DHAQs.

In-vitro AG inhibition

The activity of the rat small intestinal AG was determined by measuring the formation of glucose using glucose oxidase method.[13] The standard incubation mixture contained 0.2 M sodium phosphate buffer (pH 7.0), 25 mM maltose and 0.2 ml enzyme (approximately 0.1 units). After incubation at 37^{0}C for 30 min, the liberated glucose was measured by GOD / POD. One unit of enzyme activity was defined as the quantity of enzyme producing 1 mmol glucose per min at 37^{0}C at pH 7.0. In all the inhibitor studies, enzyme was pre-incubated with the inhibitor for 3 min before addition of the substrate. The concentration of the inhibitors used were; acarbose (100 µg) and all DHAQs at 50 ug concentration. Assays were performed in quadruplet. IC_{50} were determined for acarbose and emodin. In a similar manner, sucrase inhibition was carried out by using sucrose as the substrate.

Kinetics studies

As emodin was solely found to possess AG inhibition, kinetics of this inhibition was studied. For kinetics study varying concentrations of maltose (10, 25, 50, 75, 100, 150, 200, 250 and 300 mM) were incubated with AG in the absence of inhibitor and with 25 and 50 µg/mL of emodin in phosphate buffer (100 mM, pH 7.0) at 37^{0}C. Amount of glucose formed was determined and the data obtained was presented as

View Article Online
DOI: 10.1039/C5FO00510H

Published on 18 June 2015. Downloaded by University of Connecticut on 21/06/2015 10:55:03.

Lineweaver Burk plots to study the nature of inhibition. The inhibition of AG was studied using 25 µg and 50 µg of emodin (in 0.1% of DMSO), with increasing concentration of the substrate.

Oral maltose load

Five sets of each containing three rats were used to study the effect of oral maltose load (normal, diabetic control, positive control (acarbose) and two doses of emodin). The animals were subjected to 3 mg/g body weight of maltose load in presence of emodin (2 mg/kg body weight) and using acarbose (60 mg/kg body weight) as a positive control. Subsequently blood glucose measurements were done at specified intervals (0, 30, 60, 90 and 120 min).[14]

Molecular recognition of DHAQs at maltase-glucoamylase

To understand the structural basis of molecular recognition, the first challenge was to find the binding site for DHAQs, as no previous binding data was available. Binding site identification was done by using two different bioinformatic tools *viz.* CASTp: surface topography identification for locating the functional regions of the protein and PatchDock: docking algorithm which works on shape complementarities principles with simultaneous minimization of steric clashes for blind docking.[15,16]

Human intestinal maltase-glucoamylase apo form was obtained from RCSB Protein Data Bank (PDB ID 2QLY) with the resolution of 2.0 Å.[17] The ligands, emodin (CID 3220), aloe-emodin (CID 10207), rhein (CID 10168), chrysophanol (CID 10208) and physcion (CID 10639) were retrieved from PubChem compound database.[18] Maltase–glucoamylase enzyme was prepared using Discovery Studio 3.5. The prepared protein was submitted to PatchDock server for docking along with the ligands. The redundant solutions were discarded by the application of RMSD clustering. The clustering RMSD was kept at 1.5 Å. Top ranked 20 poses with best geometric shape complementarity scores were considered for the analysis. All the ranked poses were screened based on the number of interactions it showed and consistency in binding pattern. PatchDock results were further compared with the CASTp results in order to finalize the important residues defining the binding cavity.

The insights gained about binding site were further used to obtain the consistent binding modes of all the DHAQs using GOLD (Genetic Optimisation for Ligand Docking) version 5.2 that works on the principle of genetic algorithm. While docking it considers the conformational flexibility of ligands and rotational flexibility of selected receptor hydrogens. The binding site was defined with the residues: Arg29, Tyr46, His50, Ser74, Asn81, Phe78, Leu160 and Phe165, with detect cavity option to restrict the selection with solvent accessible surface. Twenty genetic algorithm runs were submitted for each of the anthraquinone derivative. We used GoldScore and ChemScore scoring functions separately with default setup of the parameters (except for 20 GA run setting). The molecular mechanics-like function GoldScore generates reliable poses where binding is strongly driven by hydrogen bonding and could not give optimum results where binding involves the consideration of hydrophobic sites.[19] As DHAQ derivatives have a large hydrophobic contact area, use of a molecular mechanics based scoring function ChemScore would result in more reliable binding at this sterically demanding site that works by estimating total free energy of binding ($\Delta G_{binding}$) of a ligand at a binding site. The results were analyzed based on ChemScore and number of interactions. The results obtained were compared with maltase-glucoamylase-acarbose complex (PDB ID 2QMJ) to locate the differences in molecular recognition of anthraquinones versus acarbose.

Statistical analysis

Results were expressed as Mean ± S.D. for OGTT, *in-vivo* experiments (n=3) and *in-vitro* experiments (n=4). Statistical significance was evaluated by One way ANOVA and individual comparisons were done by t-test using GraphPad Prism 6 software.

Results

Isolation of DHAQs

The isolation process yielded chrysophanol (7.2 g), physcion (1.2 g) and emodin (1.8 g) in abundance while rhein (50 mg) and aloe emodin (75 mg) were obtained in lesser quantities from 300 g powder of the obtained sample of Indian Rhubarb (Fig. 1). HPLC analysis confirmed high purity of the isolated compounds, the percent purity of which was found out to be ≥ 98% w/w basis. HPLC chromatogram of the isolated DHAQs has been provided in Fig. 1 of the supplementary data. The spectroscopic characterization of the isolated DHAQs using IR, NMR, MS and melting point has been provided in the supplementary data, which matched the ones in literature.[20,21]

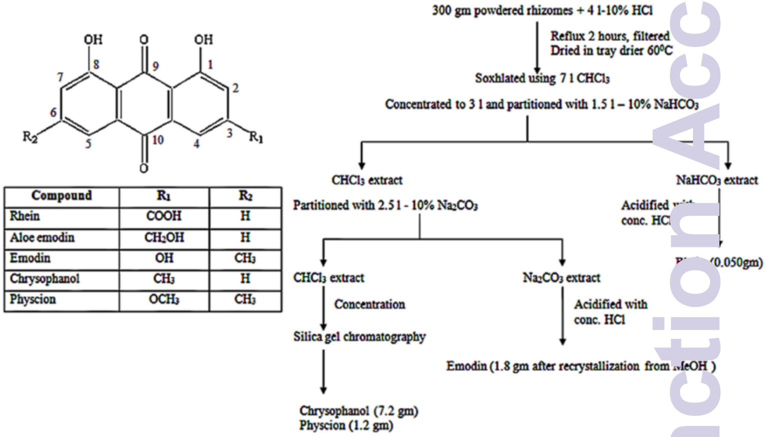

Compound	R_1	R_2
Rhein	COOH	H
Aloe emodin	CH_2OH	H
Emodin	OH	CH_3
Chrysophanol	CH_3	H
Physcion	OCH_3	CH_3

Fig. 1 Flowchart for isolation of DHAQS from *R. emodi* along with isolated compounds.

Effect of DHAQs on OGTT

OGTT with the isolated DHAQs revealed that emodin, rhein, chrysophanol and physcion could reduce blood glucose by almost 150 mg/dl as compared to the diabetic control at the end of 2 hr (Fig. 2), demonstrating a prominent anti-hyperglycemic activity. Among the isolated five DHAQs, aloe emodin was found to be most potent anti-hyperglycemic agent and was statistically significant ($p < 0.05$) than the diabetic control. Aloe emodin lowered the blood glucose level even below the starting value by 84 mg/dl after 2 hr. The other DHAQs also exhibited good anti-hyperglycemic effect in the order aloe emodin > rhein > emodin > physcion > chrysophanol.

Food & Function Accepted Manuscript

View Article Online
DOI: 10.1039/C5FO00519A

Published on 18 June 2015. Downloaded by University of Connecticut on 21/06/2015 10:55:03.

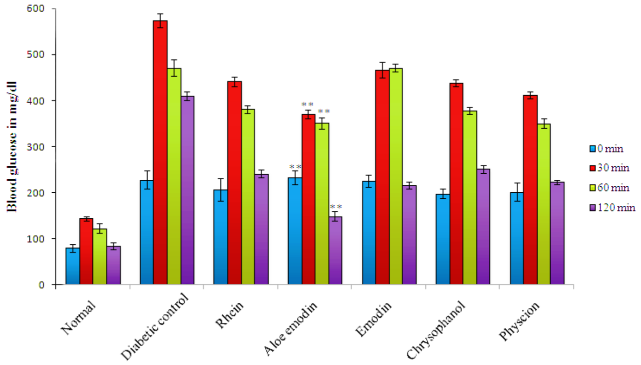

Fig. 2 Effect of isolated anthraquinones on oral glucose tolerance test. (Values are mean ±SD; n= 3. **Difference between diabetic control and aloe-emodin is statistically significant at p <0.05).

In-vitro inhibition of intestinal AG by DHAQs

Analyzing the isolated DHAQs for AG inhibition revealed that emodin demonstrated potent inhibition while the other compounds demonstrated modest to negligible activity as shown in Table 1. Emodin was found to inhibit maltase action to the tune of 93 ± 2.16%, while it inhibited sucrase to the extent of 95% (results not shown). Acarbose demonstrated 100% inhibition for both the enzymes. The IC_{50} value for emodin was found to be 30 µg/ml while IC_{50} for acarbose was found to be 60 µg/ml which suggests that emodin is significantly effective at a lower concentration.[14] It is evident from Table 1 that among the DHAQs, only emodin showed a strong intestinal AG inhibition; while the other DHAQ had minimal or negligible AG inhibitory activity.

Table 1
Observed percentage inhibition of intestinal alpha glucosidase*

Inhibitors used#	% Inhibition
Acarbose	100
Emodin	93 ± 2.16
Aloe-emodin	7.4± 1.79
Rhein	15.88 ± 3.2
Physcion	2.0 ± 0.4
Chrysophanol	0.0

Values expressed as mean ± SD (n = 4). *Maltase action using maltose as substrate was studied. #Concentration of the inhibitors used were; acarbose (100 µg) and isolated DHAQs (50 µg).

Kinetic Studies

Study of enzyme inhibition using varying concentrations of the emodin using Lineweaver-Burk plot revealed a mixed type of inhibition. With an increase in the concentration of the inhibitor the value of K_m increased while that of V_{max} decreased, as shown in Fig. 3.

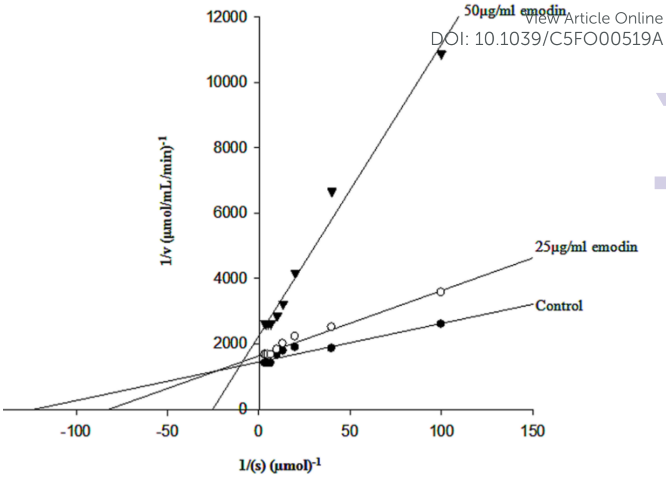

Fig. 3 Lineweaver Burk plot for kinetic analysis of alpha glucosidase inhibition by varying concentrations of emodin.

In-vivo maltase inhibition studies of emodin

As starch is the major food component of human diet it is more vital to study the maltase inhibition. It can be observed from Fig. 4 that acarbose was able to prevent post prandial spikes at 30 mins but showed a minor reduction in blood glucose value at 2 hr when compared to the diabetic control. Emodin at 1 mg/kg body weight demonstrated a good inhibition (reduction in blood glucose by 171.6 mg/dl than diabetic control) however, at a dose of 2 mg/kg it showed almost total inhibition (reduction by 275.7 mg/dl than diabetic control); as compared to acarbose (reduction in blood glucose by 92.6 mg/dl than diabetic control). A pattern comparable to normal animals was observed.

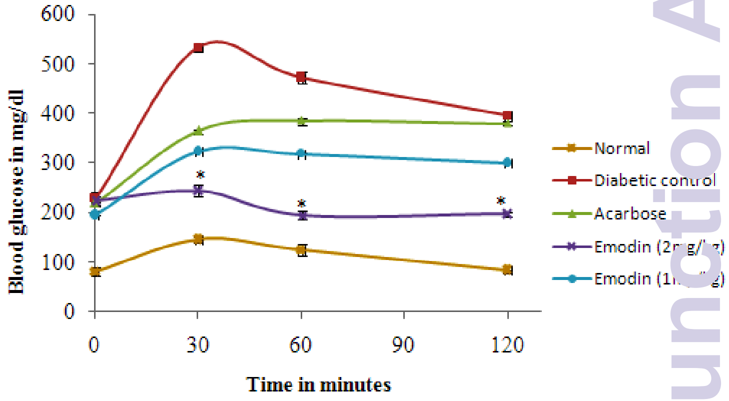

Fig. 4 Effect of maltose load (3 mg/g of body weight) on diabetic rats in absence of inhibitor and in presence of emodin, acarbose and normal. (Values are mean ±SD; n= 3. **Difference between diabetic control and emodin are statistically significant at p < 0.05).

Binding mode analysis

Binding of DHAQs versus acarbose

The exact binding site of DHAQs at the maltase enzyme is unknown. Molecular recognition studies with the use of docking approach is well known and is a proven alternative. Acarbose is a well-known competitive inhibitor and binds at the catalytic site of the enzyme. Here the quest to follow was whether the binding site for DHAQs is same to that of the

Food & Function Accepted Manuscript

This journal is © The Royal Society of Chemistry 20xx

Please do not adjust margins

View Article Online

acarbose binding site, or a separate binding site exists. Hence, a comparison based on the binding of the DHAQs with that of acarbose was essential. Combined analysis from the results of CASTp, PatchDock and GOLD exhibited following key observations (Fig. 5):

a. Binding site for DHAQs derivatives is not the same as that for acarbose.

b. The location of the quinone binding site is ~37 Å away from the acarbose binding site, based on ligand centroid distances.

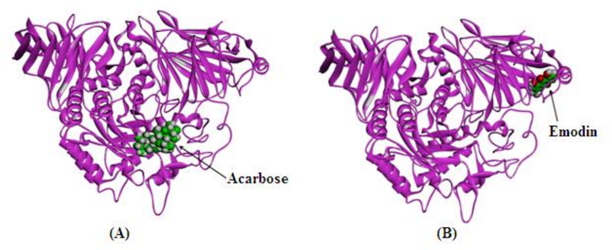

Fig. 5 Binding site location for acarbose (A) and emodin (B) on alpha glucosidase (maltase). Figure prepared using Discovery Studio Visualizer 3.5.

Characteristics of 'Quinone binding site' for DHAQs

The putative binding site comprises of residues from N-terminal domain of the protein (shown in Fig. 6A). Residues Arg29, Tyr46 and His50 are part of a trefoil Type-P domain (colored purple). Tyr46 and His50 are on the loop that connects the helix with first beta sheet**.** The residues Ser74, Val77, Phe78 and Asn81 are from the N-terminal β-sandwich domain (colored yellow). They are positioned on a long loop that connects third and fourth beta sheet of the protein.

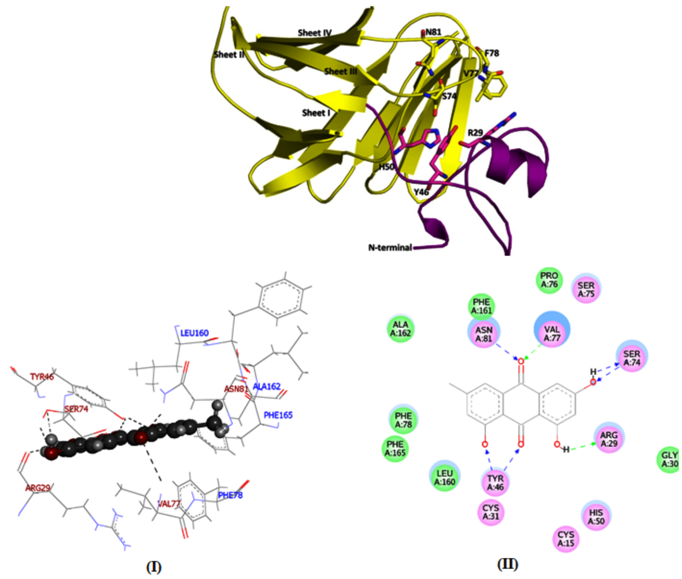

Fig. 6 A. Structural elements and residue positioning of the 'Quinone binding site'. (For clarity purpose other structural domains have not been shown. Figure prepared using PyMol [http://www.pymol.org]). **B.** Interactions of the most active emodin at Quinone binding site of alpha glucosidase (maltase): **I)** 3D view: H-bonding residues labeled in red and hydrophobic residues in blue and bonds as black dotted lines. **II)** 2D View: Residues involved in H-bonding shown by pink circles, those in van der Waals contacts by green circles, H-bonds with amino acid main chains shown by green dashed arrows directed towards the electron donor, H-bonds with amino acid side chains are shown by blue dashed arrows directed towards the electron donor.

We extensively studied binding mode for the most active compound emodin. Maximum interactions were evident for the most active compound emodin, as shown in Fig. 6B. Emodin showed interactions with all the important predicted residues, which defines the putative binding site. Residue Tyr46 forms polar contact on the 1,8-dihydroxy side of the DHAQs (Fig. 6B-II) whereas, Asn81 and Val77 interact at the opposite side. It was very interesting to note that Ser74 forms two hydrogen bonds with the third unsubstituted hydroxyl substituent of emodin. In general, the binding site is underlined by the residues such as Arg29, Tyr46, Ser74, Val77 and Asn81 and seems to be the essential counterpart for binding of DHAQs and underlines the binding cavity. Along with the polar contacts, hydrophobic contacts from the residues Phe78, Leu160, Ala162 and Phe165 seem to be indispensable in defining a binding cavity. The overall majority of binding site residue contribution comes from loop region which traps the quinone moiety to facilitate the flat-fit in the most favorable confirmation (as shown in Fig. 2 in the supplementary data).

Discussion

The major constituents isolated from the rhizomes of *R. emodi* include chrysophanol, physcion and emodin which are also present in their corresponding glycosidic form. An enrichment of DHAQs to the extent of two to three folds by using prior acid hydrolysis has been observed.[3] Isolation of the DHAQs on a larger scale from Indian Rhubarb was described earlier by Kelly et al. (1983); however, the work which was carried out on commercially obtained Indian Rhubarb extract did not mention the occurrence of aloe emodin and physcion in *R. emodi*.[22] In the present study, we were unable to separate the mixture of chrysophanol and physcion using fractional crystallization or by chemical methods of separation, and hence used silica gel chromatography. It was also difficult to separate chrysophanol and physcion using the eluent solely as light petroleum ether or a mixture containing 0.5 - 1% ethyl acetate as reported earlier.[23] The two compounds however, were satisfactorily separated when a mixture of light petroleum ether: toluene: chloroform (4:1:4) was employed as an eluent by column chromatography. The method employed by us enabled complete separation and isolation of the DHAQs from *R. emodi*. HPLC analysis demonstrated a high purity of the isolated DHAQs (≥ 98% w/w). The acid hydrolysis method used in the extraction process gave a yield of 3.4% quinones which is very lucrative for industrial production of these quinones as against the chemical synthesis of these compounds. The proposed isolation method can be easily developed into a platform technology considering the simplicity, minimum requirement of utilities and feasibility at an industrial scale-up than the recent extraction/isolation methods reported in literature.

Earlier Chen et al. (2012) showed emodin to act on multiple pathways by activating PPAR-γ that lowers blood glucose, serum cholesterol and triglyceride levels besides activating the

Published on 18 June 2015. Downloaded by University of Connecticut on 21/06/2015 10:55:03.
Food & Function Accepted Manuscript

This journal is © The Royal Society of Chemistry 20xx

View Article Online
DOI: 10.1039/C5FO00519A

Published on 18 June 2015. Downloaded by University of Connecticut on 21/06/2015 10:55:03.

Food & Function Accepted Manuscript

AMPK; and hence was expected to be most active in the OGTT.[24] However, aloe emodin was found to be most potent in OGTT and was able to reduce blood glucose almost close to normal levels. The precise mechanism for such a blood glucose lowering in OGTT by the DHAQs needs further evaluation. All DHAQs could reduce blood glucose by almost 150 mg/dl as compared to the diabetic control at the end of 2 hr (Fig. 2), demonstrating a prominent anti-hyperglycemic activity, however the *in-vitro* experiments for AG inhibition showed only emodin to be active. Emodin, thus has the advantage of having blood glucose lowering action in addition to controlling postprandial spikes.

The IC_{50} value for emodin was found to be 30 μg/ml for AG inhibition which is much less than acarbose (60 μg/ml) proving high efficacy of emodin at low concentrations. A potent inhibition of sucrase was also observed with emodin. Moreover, the AG inhibition being mixed type, emodin can act as a potent inhibitor at small concentration, unlike acarbose which is a competitive inhibitor and needed in large amounts. A recent study by Yang, et al. (2014) reported emodin, aloe-emodin, physcion and rhein to show strong α-glucosidase inhibitory activities with IC_{50} values ranging from 4.12 μM to 5.68 μM.[25] However these studies were performed on yeast alpha glucosidase. The yeast glucosidase and mammalian intestinal enzyme are structurally very different and inhibitors of yeast alpha glucosidase may not necessarily inhibit the mammalian enzyme.[26] Further it is also known that the rat intestinal enzyme is very similar to the human alpha glucosidase.[27] Hence although all the DHAQs were shown to inhibit the commercial yeast alpha glucosidase, our study showed only emodin to inhibit the mammalian enzyme and hence could be beneficial in treatment of diabetes.

Diabetic patients suffer from upregulation of intestinal AG as well as GLUT-2 transporters that lead to rapid breakdown of disaccharides and absorption of glucose, leading to much higher blood sugar levels as compared to normal individuals. This enhanced sugar-spike takes more time for clearance, thus causing protein glycation and initiating secondary complications. Inhibitors of AG therefore, play an important role in control of postprandial spikes. Our results indicated that, acarbose was able prevent post prandial spikes at 30 mins but showed a slight reduction in blood glucose value at 2 hr, when compared to diabetic control (Fig. 4). However, emodin demonstrated almost total inhibition and did not allow the blood glucose to increase in 2 hr. This is a unique potent action in comparison to majority of the known AGIs. It is likely this potent action of emodin at 2 mg/kg body weight may also be compounded by glucose lowering activity as observed in Fig. 2 in addition to the AG inhibition (Table 1). This gives the advantage to emodin as a potent anti-diabetic agent that can act at multiple targets to lower blood glucose.

Given that all the DHAQs share the same core structure and differ only at R_1 and R_2 positions, it was expected that all DHAQs would inhibit the AG. However emodin was found to be the sole potent inhibitor of intestinal AG. Docking studies were therefore initiated to observe differences in binding – if any. Docking based binding mode analysis confirmed the presence of separate binding site for the DHAQ derivatives. We call it the 'Quinone binding site' (Fig. 5B), which is not the catalytic site (Fig. 5A) and hence a different mode for anti-hyperglycemic action DHAQs exists at the AG enzyme complex. We believe that the DHAQs based AG inhibitory action is a result of mixed type inhibition. The activity is due to binding at an allosteric 'Quinone binding site' which is in accord with our kinetics study. A deeper analysis of the quinone binding site confirmed that the site is defined by the polar residues such as Arg29, Tyr46, His50, Ser74, Val77 and Asn81. On the other hand, cavity has been lined by hydrophobic residues like Phe78, Leu160, Ala162 and Phe165 assisting in the flat-fit of anthraquinone moiety consisting of three fused planar rings (Fig. 6). DHAQs share the same basic ring scaffold *i.e.* the 1,8-dihydroxyanthraquinone part, differing only at the R_1 and R_2 positions. It appears that the differences in the R_1 and R_2 positions produce a significantly differential AG inhibitory action. Docking study also highlighted that the presence of polar group either at R_1 and R_2 is a prerequisite for efficient binding, which is also evident by % inhibition values from Table 1. We believe, for effective inhibition at AG, the hydroxyl group (at R_1) facing the solvent exposed side that interacts with Ser74 is crucial, which is present only in emodin and absent in the other DHAQs (Fig. 6B-II). This explains the molecular recognition of the most active compound emodin at the AG while the other anthraquinones demonstrated minimal action. Our study provides insights regarding the quinone binding site that can pave a way for designing new molecules using the structure activity relationship approach. It also opens up an opportunity for chemical intervention at the R_1 and R_2 position to generate potent AG inhibitors.

Conclusion

The study demonstrates generation of a platform technology to isolate the DHAQs from rhizomes of *R. emodi* in a fast and efficient manner. OGTT revealed excellent anti-hyperglycemic action of the isolated DHAQs. Aloe emodin was found to possess maximum glucose lowering action while emodin was found to be the sole potent AG inhibitor amongst the isolated compounds. Emodin exhibited a mixed type of inhibition that could effectively prevent postprandial spikes, moreover acted as a potent inhibitor at small concentration and also possessed glucose lowering action. Docking studies revealed the presence of 'Quinone binding site' providing enough evidence for a mixed type of inhibition by emodin and highlighted that interaction at Ser74 is crucial for AG inhibition. The net benefit from glucose lowering effect and mixed type inhibition by emodin would enable requirement of small dosage that is safe and non toxic in case of prolonged use in treating diabetes. Also our results propose the existence of 'Quinone binding site', an allosteric site responsible for the binding of anthraquinone class of ligands at the intestinal maltase glucoamylase which can also assist in designing promising new chemical entities for treatment of diabetes.

Conflict of interest

The authors declare that they have no conflicts of interest to disclose.

Acknowledgements

The authors are thankful to University Grant Commission – Special Assistance Programme (UGC-SAP), New Delhi, India and Council of Scientific & Industrial Research (CSIR), New

 This journal is © The Royal Society of Chemistry 20xx

Please do not adjust margins

Delhi, India for providing financial support for the present work.

References

1 B. A. Zargar, M. H. Masoodi, B. Ahmed and S. A. Ganie, Phytoconstituents and therapeutic uses of *Rheum emodi* wall. ex Meissn, *Food Chem.*, 2011, **128**, 585-589.

2 W. L. Li, H. C. Zheng, J. Bukurub and N. D. Kimpe, Natural medicines used in the traditional Chinese medical system for therapy of diabetes mellitus, *J. Ethnopharmacol.*, 2004, **92**, 1–21.

3 A. U. Arvindekar, G. R. Pereira and K. S. Laddha, Assessment of conventional and novel extraction techniques on extraction efficiency of five anthraquinones from *Rheum emodi*, *J. Food Sci. Technol.*, 2015 DOI: 10.1007/s13197-015-1814-3.

4 H. R. Xiong, J. Luo, W. Hou, H. Xiao and Z. Q. Yang, The effect of emodin, an anthraquinone derivative extracted from the roots of *Rheum tanguticum*, against herpes simplex virus in vitro and in vivo, *J. Ethnopharmacol*., 2011, **133**, 718-723.

5 B. Hu, H. Zhang, X. Meng, F. Wang and P. Wang, Aloe-emodin from rhubarb (*Rheum rhabarbarum*) inhibits lipopolysaccharide - induced inflammatory responses in RAW264.7 macrophages, *J. Ethnopharmacol*., 2014, **153**, 846-853.

6 Q. Huang, G. Lu, H. M. Shen, M. C. Chung and C. N. Ong, Anti-cancer properties of anthraquinones from rhubarb, *Med. Res. Rev.*, 2007, **27**, 609-630.

7 H. Lü, J. Wang, X. Wang, X. Lin, X. Wu and Z. Xie, Rapid separation and determination of structurally related anthraquinones in Rhubarb by pressurized capillary electrochromatography, *J. Pharm. Biomed. Anal.*, 2007, **43**, 352-357.

8 S. Tong and J. Yan, Large-scale separation of hydroxyanthraquinones from *Rheum palmatum* L. by pH-zone-refining counter-current chromatography, *J. Chromatogr. A*, 2007, **1-2**, 163-168.

9 C. Lu, H. Wang, W. Lv, C. Ma, P. Xu, J. Zhu, J. Xie, B. Liu and Q. Zhou, Ionic Liquid-Based Ultrasonic/Microwave-Assisted extraction combined with UPLC for the determination of anthraquinones in Rhubarb, *Chromatographia*, 2011, **74**, 139-144.

10 Z. Tan, F. Li and X. Xu, Isolation and purification of aloe anthraquinones based on an ionic liquid/salt aqueous two-phase system, *Sep. Purif. Technol.*, 2012, **98**, 150-157.

11 J. Buse, K. Hart and L. Minasi, The PROTECT study: final results of a large multicenter postmarketing study in patients with type 2 diabetes, *Clin. Ther.,* 1998, **20**, 257-269.

12 H. X. Zhang and M. C. Liu, Separation procedures for the pharmacologically active components of rhubarb, *J. Chromatogr. B Analyt. Technol. Biomed. Sci.*, 2004, **812**, 175-181.

13 P. Trinder, Determination of glucose in blood using glucose oxidase with an alternative oxygen acceptor, *Ann. Clin. Biochem.*, 1969, **6**, 24-27.

14 V. Ghadyale, S. Takalikar, V. Haldavnekar and A. Arvindekar; Effective Control of Postprandial Glucose Level through Inhibition of Intestinal Alpha Glucosidase by *Cymbopogon martinii* (Roxb.), *Evid. Based Complement Alternat. Med.*, 2012, Article ID 372909, DOI:10.1155/2012/372909.

15 J. Dundas, Z. Ouyang, J. Tseng, A. Binkowski, Y. Turpaz and J. Liang, CASTp: computed atlas of surface topography of proteins with structural and topographical mapping of functionally annotated residues, *Nucleic Acids Res.*, 2006, **34**, W116-W118.

16 D. Schneidman-Duhovny, Y. Inbar, R. Nussinov and H. J. Wolfson, PatchDock and SymmDock: servers for rigid and symmetric docking, *Nucleic Acids Res.*, 2005, **33**, W363-W367.

17 L. Sim, R. Quezada-Calvillo, E. E. Sterchi, B. L. Nichols and D. R. Rose, Human intestinal Maltase–Glucoamylase: Crystal structure of the N-Terminal catalytic subunit and basis of inhibition and substrate specificity, *J. Mol. Biol.*, 2008, **375**, 782-792.

18 E. E. Bolton, Y. Wang, P. A. Thiessen and S. H. Bryant, PubChem: Integrated Platform of Small Molecules and Biological Activities in *Annual Reports in Computational Chemistry*, ed. R. A. Wheeler, Elsevier B.V., 2008, pp. 217-241.

19 E. Perola, W. P. Walters and P. S. Charifson, A detailed comparison of current docking and scoring methods on systems of pharmaceutical relevance, *Proteins*, 2004, **56**, 235-249.

20 R. Liu, A. Li and A. Sun, Preparative isolation and purification of hydroxyanthraquinones and cinnamic acid from the Chinese medicinal herb *Rheum officinale* Baill by high-speed counter-current chromatography, *J. Chromatogr. A*, 2004, **1052**, 217–221.

21 Y. Caro, L. Anamale, M. Fouillaud, P. Laurent, T. Petit and L. Dufosse, Natural hydroxyanthraquinoid pigments as potent food grade colorants: an overview, *Nat. Prod. Bioprospect*., 2012, **5**, 174-193.

22 T. R. Kelly, N. S. Chandrakumar, N. Walters and J. Blancaflor, Practical procedure for the isolation of emodin and chrysophanol, *J. Org. Chem*., 1983, **48**, 3573-3754.

23 S. C. Verma, N. P. Singh and A. K. Sinha, Determination and locational variations in the quantity of hydroxyanthraquinones and their glycosides in rhizomes of *Rheum emodi* using high-performance liquid chromatography, *J. Chromatogr. A*, 2005, **1097**, 59-65.

24 Z. Chen, L. Zhang, J. Yi, Z. Yang, Z. Zhang and Z. Li, Promotion of adiponectin multimerization by emodin: A novel AMPK activator with PPARγ-agonist activity, *J. Cell. Biochem*., 2012, **113**, 3547-3558.

25 D. Yang, J. Zhao, S. Liu, F. Song and Z. Liu, The screening of potential α-glucosidase inhibitors from the *Polygonum multiflorum* extract using ultrafiltration combined with liquid chromatography-tandem mass spectrometry, *Anal. Methods*, 2014, **6**, 3353-3359.

26 T. Oki, T. Matsui and Y. Osajima, Inhibitory effect of alpha glucosidase inhibitors varies according to its origin, *J. Agric. Food Chem.*, 1999, **47**, 550-553.

27 T. Oku, K. Tanabe, S. Ogawa, N. Sadamori and S. Nakamura, Similarity of hydrolyzing activity of human and rat small intestinal disaccharidases, *Clin. Exp. Gastroenterol*., 2011, **4**, 155-161.

View Article Online
DOI: 10.1039/C5FO00519A

Published on 18 June 2015. Downloaded by University of Connecticut on 21/06/2015 10:55:03.

Food & Function Accepted Manuscript
