# Supplementary material for: Emodin, a rising star in the treatment of glycolipid metabolism disorders: a preclinical systematic review and meta-analysis
Source: PeerJ. 2025 May 15;13:e19221. doi: 10.7717/peerj.19221 (PMC12085882; doi:10.7717/peerj.19221)
Supplement: Supplemental Information 20 [file peerj-13-19221-s020.zip › 8篇英文文献/Dan Xiao2018.pdf]

Accepted Manuscript

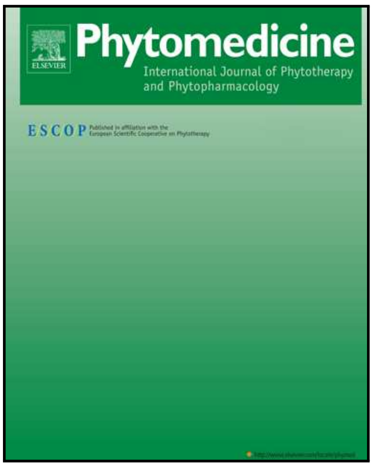

Emodin improves glucose metabolism by targeting microRNA-20b in insulin-resistant skeletal muscle

Dan Xiao , Yingying Hu , Yujie Fu , Rui Wang , Haiying Zhang , Mingqi Li , Zhange Li , Ying Zhang , Lina Xuan , Xin Li , Chaoqian Xu , Yong Zhang , Baofeng Yang

PII: S0944-7113(18)30575-0
DOI: https://doi.org/10.1016/j.phymed.2018.11.018
Reference: PHYMED 52758

To appear in: *Phytomedicine*

Received date: 28 August 2018
Revised date: 27 October 2018
Accepted date: 15 November 2018

Please cite this article as: Dan Xiao , Yingying Hu , Yujie Fu , Rui Wang , Haiying Zhang , Mingqi Li , Zhange Li , Ying Zhang , Lina Xuan , Xin Li , Chaoqian Xu , Yong Zhang , Baofeng Yang , Emodin improves glucose metabolism by targeting microRNA-20b in insulin-resistant skeletal muscle, *Phytomedicine* (2018), doi: https://doi.org/10.1016/j.phymed.2018.11.018

This is a PDF file of an unedited manuscript that has been accepted for publication. As a service to our customers we are providing this early version of the manuscript. The manuscript will undergo copyediting, typesetting, and review of the resulting proof before it is published in its final form. Please note that during the production process errors may be discovered which could affect the content, and all legal disclaimers that apply to the journal pertain.

ACCEPTED MANUSCRIPT

Emodin improves glucose metabolism by targeting microRNA-20b in insulin-resistant skeletal muscle

Dan Xiao[a], Yingying Hu[b], Yujie Fu[a], Rui Wang[a], Haiying Zhang[a], Mingqi Li[a], Zhange Li[a], Ying Zhang[a], Lina Xuan[a], Xin Li[a], Chaoqian Xu[c], Yong Zhang[a, d]*, Baofeng Yang[a, e]*

[a]Department of Pharmacology (State-Province Key Laboratories of Biomedicine-Pharmaceutics of China, Key Laboratory of Cardiovascular Research, Ministry of Education), Harbin Medical University, Harbin 150081, China

[b]Department of Pharmacy, the First Affiliated Hospital of Harbin Medical University, Harbin 150081, China

[c]Mudanjiang medical university, 157000, China

[d]Institute of Metabolic Disease, Heilongjiang Academy of Medical Science, Harbin, 150086, China

[e]Department of Pharmacology and Therapeutics, Melbourne School of Biomedical Sciences, Faculty of Medicine, Dentistry and Health Sciences, the University of Melbourne, Melbourne, 3010, Australia

*****Corresponding authors

Correspondence to Yong Zhang, PhD, Department of Pharmacology, Harbin Medical University, 157 Baojian Road, Nangang District, Harbin, Heilongjiang 150081, China. Tel.: 86-451-86671354. Fax: 86-451-86669482. E-mail: hmuzhangyong@hotmail.com or Baofeng Yang, PhD, Department of Pharmacology, Harbin Medical University, 157 Baojian Road, Nangang District, Harbin, Heilongjiang 150081, China. Tel.: 86-451-86671354. Fax: 86-451-86669482. E-mail: yangbf@ems.hrbmu.edu.cn.

Word count: 4990

ACCEPTED MANUSCRIPT

ACCEPTED MANUSCRIPT

Abstract

Background: Emerging evidence has indicated the therapeutic potential of emodin with its multiple pharmacological effects.

Purpose: To evaluate role of emodin in regulating insulin resistance (IR) and to elucidate the underlying molecular mechanisms.

Study Design/Methods: Fasting blood glucose (FBG) and lipid levels were measured before and after intragastric administration of emodin in type 2 diabetes mellitus (T2DM) rats. Glucose consumption was determined in L6 cells to investigate the effect of emodin on glucose metabolism. Expression of miR-20b and SMAD7 was quantified by real-time PCR for mRNAs or western blot analysis for proteins.

Results: Emodin ameliorated hyperglycemia and dyslipidemia in T2DM rats, and glucose metabolism in a concentration- and time-dependent manner. MiR-20b was markedly upregulated in the setting of IR and overexpression of miR-20b disrupted glucose metabolism by repressing SMAD7 in L6 cells. Knockdown of this miRNA produced the opposite effects. Emodin abolished the abnormal upregulation of miR-20b and indirectly upregulated SMAD7.

Conclusion: Emodin improves glucose metabolism to produce anti-IR effects, and downregulation of miR-20b thereby upregulation of SMAD7 is an underlying mechanism for the beneficial effects of emodin.

Key words: Insulin resistance; skeletal muscle; emodin; miR-20b; SMAD7.

ACCEPTED MANUSCRIPT

ACCEPTED MANUSCRIPT

Abbreviations

IR: insulin resistance, T2DM: type 2 diabetes mellitus, FBG: fasting blood glucose, microRNA: miRNA, GLUT4: glucose transporter 4, VEGF: vascular endothelial growth factor, NIH: National Institutes of Health, HFD: high fat diet, i.p.: intraperitoneal, STZ: streptozocin, TG: triglyceride, TC: total cholesterol, LDL-C: low density lipoprotein-cholesterol, HDL-C: high density lipoprotein-cholesterol, MET: metformin, DMSO: dimethyl sulfoxide, FBS: fetal bovine serum, DMEM: Dulbecco's modified eagle medium, MEM: minimum essential medium, AMO-20b: anti-miR-20b anti-sense inhibitor, NC: negative control

ACCEPTED MANUSCRIPT

ACCEPTED MANUSCRIPT

Introduction

Patients with type 2 diabetes mellitus (T2DM) can cause various micro-/macrovascular complications and increase the rate of mortality (Lu et al., 2013). Insulin resistance (IR) is the most critical issue of T2DM, characterized by glucose metabolic disorders in skeletal muscle cells (Nankar and Doble, 2017). Because of the large mass, changes of glucose homeostasis in skeletal muscles can affect whole body metabolism (Yan and Li, 2013), and abnormal glucose metabolism in skeletal muscle contributes to peripheral IR (Nishikawa et al., 2012).

Whilst anti-diabetic medications are common, natural medicines have been shown to improve blood glucose control, reduce complications and favorably affect cardiovascular functions (Zhao et al., 2017). Increasing attention has been shifting to natural medicine, since its high safety profiles and multiple pharmacological functions (Zhu et al., 2017). Emodin, a bio-active anthraquinone derivative isolated from rhizomes has been reported to produce a broad-spectrum of function including anti-oxidation, anti-aging, anti-inflammation, and anti-virus effects (Monisha et al., 2016), as well as its beneficial actions in obesity-related metabolic diseases (Cao et al., 2016; Wu et al., 2014). However, whether emodin can elicit favorable effects in skeletal muscle with IR remained unknown.

Emerging evidence has indicated that microRNAs (miRNAs) are involved in T2DM pathogenesis by regulating blood glucose levels and the onset of IR (Sung et al., 2018). For instance, upregulation of miR-30d, miR-106b, and miR-93 diminishes the translocation of glucose transporter 4 (GLUT4) thereby eliminating glucose-stimulated response in T2DM skeletal muscles (Zhou et al., 2016). Our pilot study demonstrated that

emodin altered the expression of miR-20b, and it is noted that miR-20b shares identical seed sequence with miR-106b. We therefore proposed that emodin could affect the expression of miR-20b to regulate glucose metabolism. This study was designed to test this hypothesis and to delineate the underlying cellular signaling mechanisms. Our data suggest that emodin upregulated miR-20b that represses its target gene *Smad7* to produce anti-IR effects in a rat model of T2DM and a cellular model of IR with L6 skeletal muscle cells.

Materials and Methods

Drugs

Emodin (⩾98% in purity, molecular weight: 270.24) was purchased from Chengdu Pufei DE Biotech Co. (Chengdu, China, batch no. 17042501). The structure of emodin and its high-performance liquid chromatography are shown in Figure S1. Metformin (MET) was purchased from Shandong Sidebang Pharmaceutical Co. (Shandong, China). For *in vitro* experiments, emodin was dissolved in dimethyl sulfoxide (DMSO, Sigma, Louis, MO, USA). In parallel, control cells were treated with a non-toxic final concentration of 0.1% DMSO.

Ethics statement

Animal studies were approved by the Ethic Committees of Harbin Medical University. All procedures comply with the Guide for the Use and Care of Laboratory Animals published by US National Institutes of Health (NIH, Publication No. 85-23, revised 1996).

Animals and experimental design

ACCEPTED MANUSCRIPT

Twelve-week-old Wistar rats (male, 180-220 g) were purchased from the Animal Center of the 2nd Affiliated Hospital of Harbin Medical University (Harbin, Heilongjiang China) and housed in a dedicated room with 12 h dark/light cycle, controlled temperature (22 ± 1°C), and constant humidity (55 ± 5%). After one-week acclimatization, rats were randomly divided into six groups (n=10 for each group): the control group, the T2DM group, the T2DM+emodin groups (the low-, middle-, and high-dose groups received a 20 mg/kg, 40 mg/kg, and 80 mg/kg of emodin, respectively), and the T2DM+MET in group (100 mg/kg). Emodin (dissolved in distilled water with 0.5% sodium carboxymethylcellulose) and MET were administered via gastric gavage for 4 weeks.

Rat model of T2DM

All T2DM groups were fed with a high fat diet (HFD) for 1 month and then subjected to intraperitoneal (i.p.) injection of 25 mg/kg/d streptozocin (STZ), dissolved in 0.1M citrate buffer solution (pH4.2) for three days. Fasting blood glucose (FBG) level was measured to confirm the successful T2DM model induction with FBG >16.7 mM on days 3 and 7 (Zhang et al., 2016).

Body weight and blood testing

Four-weeks after administration with emodin, body weight was monitored at the time points of 0, 1, and 4 weeks, and the data from 4 weeks were selected for analysis. Blood samples were collected for FBG and blood lipid analyses, including triglyceride (TG), total cholesterol (TC), low density lipoprotein-cholesterol (LDL-C), and high-density lipoprotein-cholesterol (HDL-C). Blood lipid assay kits were purchased from Nanjing Jiancheng Biological Engineering Research Institute (Nanjing, Jiangsu, China).

Cell culture and treatment

ACCEPTED MANUSCRIPT

ACCEPTED MANUSCRIPT

L6 myoblasts (rat skeletal muscle cell line) were purchased from Shanghai Institute for Biological Sciences (Shanghai, China) and cultured in Dulbecco's modified eagle medium (DMEM, 25mM glucose, Sigma, Louis, MO, USA) supplied with 10% fetal bovine serum (FBS, Gibco, Carlsbad, CA, USA) and 1% penicillin/streptomycin (100 μg/ml) in 5% CO_2 at 37°C. Medium was replaced with minimum essential medium (MEM, Missouri, Sigma-Aldrich, USA) containing 2% FBS and 1% penicillin/streptomycin for differentiation when cells reached 70% confluence. Cell medium was refreshed every other day, and differentiation was detected 10 days post initiation of differentiation. Fully differentiated L6 cells with aligned and fused myotubes were used. Before treatment, cells were incubated with FBS-free restriction/treatment medium overnight. Insulin-resistant L6 myotube cells (IR-L6) were induced by 750 μM palmitic acid (PA, Sigma, US) for 12 h as previous described in previous studies (Song et al., 2014; Wu et al., 2015).

Cell transfection

L6 myotube cells were starved in serum-free medium for 24 h, then transiently transfected with miR-20b mimic, anti-miR-20b anti-sense inhibitor (AMO-20b) or negative control (NC) (RiboBio Co., Ltd., Guangzhou, Guangdong, China) using X-treme GENE siRNA transfection reagent (catalog #04476093001, Roche, Indianapolis, USA) according to the manufacturer's instructions.

Real-time PCR

Total RNA was harvested using TRIzol reagent (Invitrogen, Carlsbad, CA, USA) according to the protocols described in detailed in previous studies (Zhang et al., 2014; Zhang et al., 2015). cDNA synthesis was performed using a High Capacity cDNA

Reverse Transcription Kit (Applied Biosystems, Carlsbad, CA, USA, Cat. no. 4368814) according to the manufacturer's instructions. The levels of miR-20b and SMAD7 mRNA were determined by SYBR Green I incorporation method and ABI 7500 fast Real Time PCR system (Applied Biosystems, Carlsbad, CA, USA). Primer pairs for miR-20b were designed by RiboBio Co., Ltd. (Guangzhou, Guangdong, China). U6 and GAPDH were used as internal controls for miR-20b and SMAD7, respectively.

Western blot

Western blot analysis was performed as previously described (Li et al., 2018; Xu et al., 2014). After blocking, the membrane was incubated with the primary antibody for SMAD7 (Wanlei Bio, Shenyang, Liaoning, China), followed by incubation with a florescence-labeled secondary antibody. The blotted proteins were detected and quantified by Odyssey Infrared Imaging System (LI-COR, Lincoln, NB, USA). GAPDH (Santa Cruz Biotechnology, Dallas, Texas) was used as internal control.

Immunofluorescent staining

Immunofluorescent staining was performed in L6 cells as previously described (Li et al., 2014). SMAD7 was stained with primary antibody for 24 h at 4℃. Then, the cells were probed with florescence-labeled secondary antibody and DAPI for nuclei, and examined under a confocal laser scanning microscope (FV300, Olympus, Tokyo, Japan).

Glucose consumption assay

Cells were cultured in 6-well plates. After appropriate treatment, the glucose concentration of cells in the medium was measured by the glucose oxidase method (F006, Nanjing Jiancheng Biological Engineering Research Institute, Nanjing, Jiangsu, China). The experimental procedures were essentially the same as those described previously

ACCEPTED MANUSCRIPT

ACCEPTED MANUSCRIPT

(Zhou et al., 2016).

Measurement of cell viability

L6 cells were seeded in 96-well plate. After appropriate treatment, MTT assay was used to evaluate cell viability. MTT solution (20 μL) was added to each well and incubated for 4 h. The medium was displaced by 150 μL DMSO. Absorbance values were recorded at 570 nm.

Statistical analysis

All values are presented as mean ± S.E.M. Statistical comparisons were performed by Student's t-test between two groups or one-way ANOVA for multiple comparisons (Shen et al., 2014). $p < 0.05$ was considered to indicate a significant difference. Data were analyzed using the GraphPad Prism 5.0 software.

Results

Emodin improves glucose metabolism in T2DM rats and in IR L6 cells

T2DM rats are characterized by excess body weight increase and constant hyperglycemia and hyperlipidemia due presumably to glucose metabolic dysfunction. Four weeks after intragastric administration of emodin, FBG level remained unaltered in control rats relative to the baseline value whereas it was significantly elevated in T2DM rats on days 3 and 7 compared with day 0 (Figure 1a). Emodin conspicuously attenuated hyperglycemia and excess increase of body weight (Figure 1b, c), as well as hyperlipidemic state (Figure 1d, e and Figure S2a, b).

While the above results suggest emodin might have anti-IR effects, our subsequent experiments indeed provided supporting evidence for this notion. As shown in Figure 2a

ACCEPTED MANUSCRIPT

& b, emodin significantly boosted up glucose consumption in a concentration- and time-dependent manner in treated IR L6 cells compared with non-treated IR L6 cells.

Interestingly, but perhaps not surprisingly, emodin also enhanced glucose metabolism in normal (non-IR) L6 cells with significant effects occurring at a concentration of as low as 5 μM. Higher concentrations of emodin (≤20 μM) did not cause further increases in the level of glucose metabolism (Figure S3a). These results implied that emodin may function as an antagonist against a specific factor with its level maintaining at a low range under normal conditions, but increasing during IR in L6 cells.

We also noticed that higher concentrations of emodin (40 μM and 80 μM) inhibited glucose consumption levels, and we assumed that such high concentrations of emodin might cause cytotoxicity. We therefore measured the effects of emodin on viability of L6 cells using MTT assay. As illustrated in Figure S3b, exposure of IR L6 cells to emodin at concentrations ≤20 μM for 24 h did not affect cell viability, but at 40 μM and 80 μM, emodin significantly decreased cell viability. Based upon these results, we utilized this 20 μM emodin in our subsequent *in vitro* experiments.

MiR-20b mediates the beneficial effects of emodin on glucose metabolism in skeletal muscle

Next, we wanted to elucidate how emodin regulates glucose metabolism. To this end, we choose to study the potential role of miR-20b because this miRNA is known to be associated with skeletal muscle tissue of diabetic rats (Zhou et al., 2016), and is positively regulated by emodin (Lin et al., 2015). We first evaluated the effects of emodin on miR-20b levels in T2DM skeletal muscle tissues and IR L6 cells. Figure 3a & b clearly

ACCEPTED MANUSCRIPT

demonstrated that miR-20b level was markedly elevated in both in T2DM rats and IR L6 cells, and this elevation was reversed by emodin treatment.

To investigate if the upregulation of miR-20b in T2DM and IR has any functional outcomes, we first employed gain-of-function approach by transfecting miR-20b mimic into L6 cells and monitored the changes of glucose consumption. As depicted in Figure 3c, miR-20b produced remarkable reduction of glucose consumption in treated normal L6 cells, an alteration resembling that caused by IR (Figure 3d). On the other hand, in IR-L6 cells, transfection of AMO-20b to knockdown endogenous miR-20b restored the impaired glucose consumption (Figure 3d), similar to the effect elicited by emodin (Figure 2).

The efficiency of miR-20b mimic transfection was confirmed by 3-fold increase in cellular miR-20b level (Figure S4).

MiR-20b suppresses SMAD7 expression in skeletal muscle cells

While the above results indicate that emodin improves glucose consumption by downregulating miR-20b, we decided to delineate the targeting mechanisms of miR-20b. Our computational analysis using bio-informatics interrogation miRNADA (http://www.microrna.org) indicated SMAD7 a theoretical target of miR-20b with broadly conserved site both in the species of human and rat (Table 1) and SMAD7 is a well-recognized inhibitor of the TGF-β/SMADs signaling pathway, which can produce beneficial effects against IR (Wang et al., 2017) . We therefore performed the following experiments to verify the relationship between miR-20b and SMAD7 in L6 cells.

We first observe that overexpression of miR-20b significantly reduced mRNA and protein levels of SMAD7, and co-transfection with AMO-20b reversed the effects (Figure 4).

ACCEPTED MANUSCRIPT

ACCEPTED MANUSCRIPT

We then found that SMAD7 mRNA and protein levels were considerably decreased in IR-L6 cells compared with non-IR L6 cells for control (Figure 5a, b).

Notably, miR-20b knockdown by AMO-20b robustly upregulated the mRNA and protein levels of SMAD7 in IR cells (Figure 5c, d).

Enrichment of SMAD7 attributes to pharmacological functions of emodin in insulin-resistant skeletal muscle

It appears from the above-presented data that SMAD7 is an effector mediating the effect of miR-20b thereby emodin on glucose consumption. If this true, then we expect to see a decrease in SMAD7 levels in T2DM and a restoration of its levels after emodin treatment. To test this notion, we next turned to look at the relationship between emodin and SMAD7.

Our results revealed significant lower levels of SMAD7 mRNA and protein in both T2DM (Figure 6a, b) and IR-L6 cells (Figure 6c, d), and the loss of SMAD7 expression was rescued by emodin in both *in vivo* and *in vitro* models of IR.

Moreover, our immunocytochemical fluorescent analysis provided another piece of evidence for the positive regulation of SMAD7 expression by emodin in IR-L6 cells (Figure 6e, f).

Discussion

The present study generated several lines of evidence for the beneficial effect of emodin on IR with a rate model of T2DM and a cellular model of IR in skeletal muscles. First, emodin improved glucose metabolism in T2DM rats and in IR skeletal muscles as well. Second, emodin significantly restored the abnormally upregulated expression of miR-20b

ACCEPTED MANUSCRIPT

ACCEPTED MANUSCRIPT

caused by T2DM and IR. Third, we experimentally established SMAD7 as a target gene for miR-20b and found that miR-20b impaired glucose consumption by repressing SMAD7 expression. Notably, knockdown of miR-20b by its antisense inhibitor rescued the depressed glucose consumption. Taken together, it allowed us to draw a conclusion that emodin produces anti-IR effects by downregulating miR-20b thereby upregulating SMAD7 due to derepression from miR-20b inhibition. Thus, the miR-20b/SMAD7 axis appears to be at least partly the signaling mechanism for the beneficial action of emodin in IR.

Insulin-dependent IR is one of the most important causes resulting in the continuous high blood glucose level, which is causally associated with multiple severe complications (Mao et al., 2009). Emodin is a natural anthraquinone and it possesses multiple favorable effects such as anti-tumor, anti-bacterial, diuretic and vasorelaxant agent with high selectivity towards diseased lesions (Dai et al., 2017; Huang et al., 2013). In spite of long history of emodin since first reported in 1925 (Monisha et al., 2016), relatively little is known about this compound until recent decades. A published study showed that treatment with 20 μM emodin effectively reduces lipid accumulation in insulin-resistant L6 cells (Cao et al., 2016). However, the role of emodin in regulating glucose consumption in the setting of IR has not been previously reported. This study thus represents the first to unravel the anti-IR property of emodin in animal and cellular models.

Recent studies have demonstrated that miR-17 family is linked to diabetic diseases. Zhang et al found that miR-17 improves insulin sensitivity in adipose tissue (Zhang et al., 2018). Zhu et al identified miR-20b as a biomarker in maternal plasma to predict

gestational diabetes mellitus (Zhu et al., 2015). MiR-20b is related to diabetic complications (Qin et al., 2016). The present study further revealed that miR-20b played an important role in regulating glucose consumption in IR skeletal muscles and overexpression of this miRNA might be responsible for IR-induced glucose metabolic disorders. Notably, our data indicate that downregulation of miR-20b likely contributes to the beneficial effects of emodin.

Furthermore, our experimental results suggest that miR-20b decreased glucose consumption via repressing the expression of its target gene SMAD7, and downregulation of miR-20b by emodin derepressed SMAD7 to improve glucose metabolism. SMAD7 is commonly known as an antagonist in the TGF-β/SMADs pathway, exerting its inhibitory effect through impeding SMAD3 activity (Xu et al., 2013). The TGF-β signaling pathway prompts the development of diabetic complications and aggravates glucose metabolic disorders (Wang et al., 2014). Therefore, our study implied that emodin could act on the TGF-β/SMADs pathway via the miR-20b/SMAD7 axis.

Conclusions

In this study, we found that emodin improves glucose metabolism by regulating the miR-20b/SMAD7 axis in insulin-resistant skeletal muscle tissues. Our findings suggest that emodin might be considered as a potential medication for the treatment of IR.

Acknowledgments

This work was supported by the National Key R&D Program of China

ACCEPTED MANUSCRIPT

ACCEPTED MANUSCRIPT

(2017YFC1702003), the National Natural Science Foundation of China (81570399/81773735/81811530117), and Heilongjiang Outstanding Youth Science Fund (JJ2017JQ0035).

Conflicts of Interest: The authors declare that there is no conflict of interest regarding the publication of this article.

References

Cao, Y., Chang, S., Dong, J., Zhu, S., Zheng, X., Li, J., Long, R., Zhou, Y., Cui, J., Zhang, Y., 2016. Emodin ameliorates high-fat-diet induced insulin resistance in rats by reducing lipid accumulation in skeletal muscle. Eur J Pharmacol 780, 194-201.

Dai, J.P., Wang, Q.W., Su, Y., Gu, L.M., Zhao, Y., Chen, X.X., Chen, C., Li, W.Z., Wang, G.F., Li, K.S., 2017. Emodin Inhibition of Influenza A Virus Replication and Influenza Viral Pneumonia via the Nrf2, TLR4, p38/JNK and NF-kappaB Pathways. Molecules 22.

Huang, P.H., Huang, C.Y., Chen, M.C., Lee, Y.T., Yue, C.H., Wang, H.Y., Lin, H., 2013. Emodin and Aloe-Emodin Suppress Breast Cancer Cell Proliferation through ER alpha Inhibition. Evid Based Complement Alternat Med 2013, 376123.

Li, X., Du, N., Zhang, Q., Li, J., Chen, X., Liu, X., Hu, Y., Qin, W., Shen, N., Xu, C., Fang, Z., Wei, Y., Wang, R., Du, Z., Zhang, Y., Lu, Y., 2014. MicroRNA-30d regulates cardiomyocyte pyroptosis by directly targeting foxo3a in diabetic cardiomyopathy. Cell Death Dis 5, e1479.

Li, X., Edwards, M., Swaney, K.F., Singh, N., Bhattacharya, S., Borleis, J., Long, Y., Iglesias, P.A., Chen, J., Devreotes, P.N., 2018. Mutually inhibitory Ras-PI(3,4)P2 feedback loops mediate cell migration. Proc Natl Acad Sci U S A.

Lin, S.Z., Xu, J.B., Ji, X., Chen, H., Xu, H.T., Hu, P., Chen, L., Guo, J.Q., Chen, M.Y., Lu, D., Wang, Z.H., Tong, H.F., 2015. Emodin inhibits angiogenesis in pancreatic cancer by regulating the transforming growth factor-beta/drosophila mothers against decapentaplegic pathway and angiogenesis-associated microRNAs. Mol Med Rep 12, 5865-5871.

Lu, J., Xie, G., Jia, W., 2013. Metabolomics in human type 2 diabetes research. Front Med 7, 4-13.

Mao, X.Q., Yu, F., Wang, N., Wu, Y., Zou, F., Wu, K., Liu, M., Ouyang, J.P., 2009. Hypoglycemic effect of polysaccharide enriched extract of Astragalus membranaceus in diet induced insulin resistant C57BL/6J mice and its potential mechanism. Phytomedicine 16, 416-425.

Monisha, B.A., Kumar, N., Tiku, A.B., 2016. Emodin and Its Role in Chronic Diseases. Adv Exp Med Biol 928, 47-73.

Nankar, R.P., Doble, M., 2017. Hybrid drug combination: Anti-diabetic treatment of type 2 diabetic Wistar rats with combination of ellagic acid and pioglitazone. Phytomedicine 37, 4-9.

Nishikawa, S., Hosokawa, M., Miyashita, K., 2012. Fucoxanthin promotes translocation and induction of glucose transporter 4 in skeletal muscles of diabetic/obese KK-A(y) mice. Phytomedicine 19, 389-394.

Qin, B., Liu, J., Liu, S., Li, B., Ren, J., 2016. MiR-20b targets AKT3 and modulates vascular endothelial growth factor-mediated changes in diabetic retinopathy. Acta Biochim Biophys Sin (Shanghai) 48, 732-740.

Shen, N., Li, X., Zhou, T., Bilal, M.U., Du, N., Hu, Y., Qin, W., Xie, Y., Wang, H., Wu, J., Ju, J., Fang, Z., Wang, L., Zhang, Y., 2014. Shensong Yangxin Capsule prevents diabetic myocardial fibrosis by inhibiting TGF-beta1/Smad signaling. J Ethnopharmacol 157, 161-170.

ACCEPTED MANUSCRIPT

ACCEPTED MANUSCRIPT

Song, Y., Shi, J., Wu, Y., Han, C., Zou, J., Shi, Y., Liu, Z., 2014. Metformin ameliorates insulin resistance in L6 rat skeletal muscle cells through upregulation of SIRT3. Chin Med J (Engl) 127, 1523-1529.
Sung, H.C., Liu, C.W., Hsiao, C.Y., Lin, S.R., Yu, I.S., Lin, S.W., Chiang, M.H., Liang, C.J., Pu, C.M., Chen, Y.C., Lin, M.S., Chen, Y.L., 2018. The effects of wild bitter gourd fruit extracts on ICAM-1 expression in pulmonary epithelial cells of C57BL/6J mice and microRNA-221/222 knockout mice: Involvement of the miR-221/-222/PI3K/AKT/NF-kappaB pathway. Phytomedicine 42, 90-99.
Wang, J., Gao, Y., Duan, L., Wei, S., Liu, J., Tian, L., Quan, J., Zhang, Q., Yang, J., 2017. Metformin ameliorates skeletal muscle insulin resistance by inhibiting miR-21 expression in a high-fat dietary rat model. Oncotarget 8, 98029-98039.
Wang, J.Y., Gao, Y.B., Zhang, N., Zou, D.W., Wang, P., Zhu, Z.Y., Li, J.Y., Zhou, S.N., Wang, S.C., Wang, Y.Y., Yang, J.K., 2014. miR-21 overexpression enhances TGF-beta1-induced epithelial-to-mesenchymal transition by target smad7 and aggravates renal damage in diabetic nephropathy. Mol Cell Endocrinol 392, 163-172.
Wu, W., Bi, Y., Tangsun, Y., Yin, W., Chen, Y., Zhu, D., 2015. [Effects of transcription factor sterol regulatory element binding protein-1c in palmitate acid-induced L6 cells insulin resistance and its mechanism]. Zhonghua Yi Xue Za Zhi 95, 611-615.
Wu, Z., Chen, Q., Ke, D., Li, G., Deng, W., 2014. Emodin protects against diabetic cardiomyopathy by regulating the AKT/GSK-3beta signaling pathway in the rat model. Molecules 19, 14782-14793.
Xu, C., Hu, Y., Hou, L., Ju, J., Li, X., Du, N., Guan, X., Liu, Z., Zhang, T., Qin, W., Shen, N., Bilal, M.U., Lu, Y., Zhang, Y., Shan, H., 2014. beta-Blocker carvedilol protects cardiomyocytes against oxidative stress-induced apoptosis by up-regulating miR-133 expression. J Mol Cell Cardiol 75, 111-121.
Xu, L., Zheng, N., He, Q., Li, R., Zhang, K., Liang, T., 2013. Puerarin, isolated from Pueraria lobata (Willd.), protects against hepatotoxicity via specific inhibition of the TGF-beta1/Smad signaling pathway, thereby leading to anti-fibrotic effect. Phytomedicine 20, 1172-1179.
Yan, W., Li, X., 2013. Impact of diabetes and its treatments on skeletal diseases. Front Med 7, 81-90.
Zhang, C., Qian, D., Zhao, H., Lv, N., Yu, P., Sun, Z., 2018. MiR17 improves insulin sensitivity through inhibiting expression of ASK1 and anti-inflammation of macrophages. Biomed Pharmacother 100, 448-454.
Zhang, Y., Li, X., Li, J., Zhang, Q., Chen, X., Liu, X., Zhang, H., Yang, H., Hu, Y., Wu, X., Ju, J., Yang, B., 2016. The anti-hyperglycemic efficacy of a lipid-lowering drug Daming capsule and the underlying signaling mechanisms in a rat model of diabetes mellitus. Sci Rep 6, 34284.
Zhang, Y., Li, X., Zhang, Q., Li, J., Ju, J., Du, N., Liu, X., Chen, X., Cheng, F., Yang, L., Xu, C., Bilal, M.U., Wei, Y., Lu, Y., Yang, B., 2014. Berberine hydrochloride prevents postsurgery intestinal adhesion and inflammation in rats. J Pharmacol Exp Ther 349, 417-426.
Zhang, Y., Qin, W., Zhang, L., Wu, X., Du, N., Hu, Y., Li, X., Shen, N., Xiao, D., Zhang, H., Li, Z., Yang, H., Gao, F., Du, Z., Xu, C., Yang, B., 2015. MicroRNA-26a prevents endothelial cell apoptosis by directly targeting TRPC6 in the setting of atherosclerosis. Sci Rep 5, 9401.
Zhao, Y., Liu, B., He, L., Bai, W., Yu, X., Cao, X., Luo, L., Rong, P., Li, G., 2017. A novel classification method for aid decision of traditional Chinese patent medicines for stroke treatment. Front Med 11, 432-439.
Zhou, T., Meng, X., Che, H., Shen, N., Xiao, D., Song, X., Liang, M., Fu, X., Ju, J., Li, Y., Xu, C., Zhang, Y., Wang, L., 2016. Regulation of Insulin Resistance by Multiple MiRNAs via Targeting the GLUT4 Signalling Pathway. Cell Physiol Biochem 38, 2063-2078.
Zhu, Y., Tian, F., Li, H., Zhou, Y., Lu, J., Ge, Q., 2015. Profiling maternal plasma microRNA expression in early pregnancy to predict gestational diabetes mellitus. Int J Gynaecol Obstet 130, 49-53.
Zhu, Z., Gui, Y., Wang, L., Wang, T., Yang, Y., Niu, Y., Fu, D., Wang, J., Cui, T., 2017. Innovative development path of ethnomedicines: a case study. Front Med 11, 297-305.

Table legends

ACCEPTED MANUSCRIPT

Determined corresponded pairing of target region (top) and miRNA(bottom)	
Position 1167 of Smad7 3' UTR<br>*rno-miR-20b*	*3' ...uggacgugauacucGUGAAAc ...5'*<br>*5' ... aaugagcaugcucaCACUUUa ...3'*
position 1428 of Smad7 3' UTR<br>*rno-miR-20b*	*5' ...uggacgugaUACUCGUGAAAc ...3'*<br>3'*...aauaaagaaAAGAGCACUUUg…5'*
Position 1350 of Smad7 3' UTR<br>*hsa-miR-20b*	*3'...gauggacgugaUACUCGUGAAAc...5'*<br>*5'...aaaauaaagaaAAGAUCACUUUg...3'*

Sequences marked in red indicating the corresponding pairs between miR-20 and Smad7

Table 1. Sequence alignment between miR-20b and SMAD7 mRNA showing the complementarity of the seed region.

Sequences marked in red indicating the base pairing between miR-20 and SMAD7.

Figure legends

ACCEPTED MANUSCRIPT

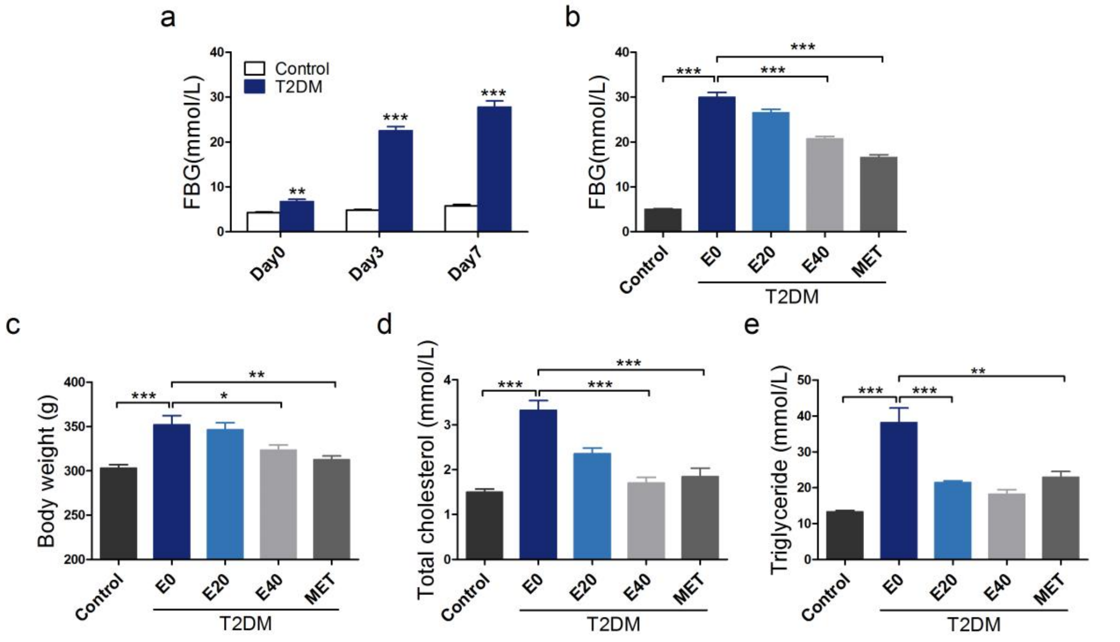

Figure 1. Emodin treatment attenuates glucose metabolic dysfunction in T2DM rats. **(a)** Fast blood glucose (FBG) levels in control and high fat diet/streptozocin (HFD/STZ)-induced T2DM rats. Day 0 represents STZ intraperitoneal injection, n = 10 for control group and n = 40 for T2DM group. The data are presented as the mean ± S.E.M., $**p < 0.01$, $***p < 0.001$ versus T2DM group. **(b-e)** FBG, body weight, total cholesterol (TC) and triglyceride (TG) levels. Intragastrically treatment with emodin (20 mg/kg, 40 mg/kg), or MET (100 mg/kg) showed different improvements compared with non-treated T2DM rats, n = 10 in each group, $*p < 0.05$, $**p < 0.01$, $***p < 0.001$.

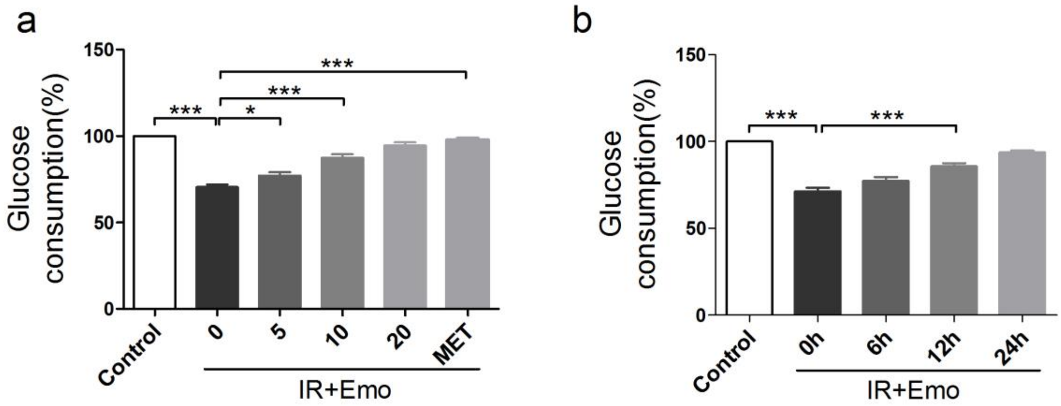

Figure 2. Emodin ameliorates reduced glucose metabolism in insulin-resistant L6 cells. (a) Glucose consumption in insulin-resistant L6 cells with different concentration of emodin (5 µM, 10 µM, 20 µM) or MET. Non-treated L6 cells were used as control group. The degree of basal glucose consumption in L6 cells was set to 100 %, n = 10, **p* < 0.05, ****p* < 0.001. **(b)** Glucose consumption at different time points with emodin concentration of 20 µM, n = 10, ****p* < 0.001.

ACCEPTED MANUSCRIPT

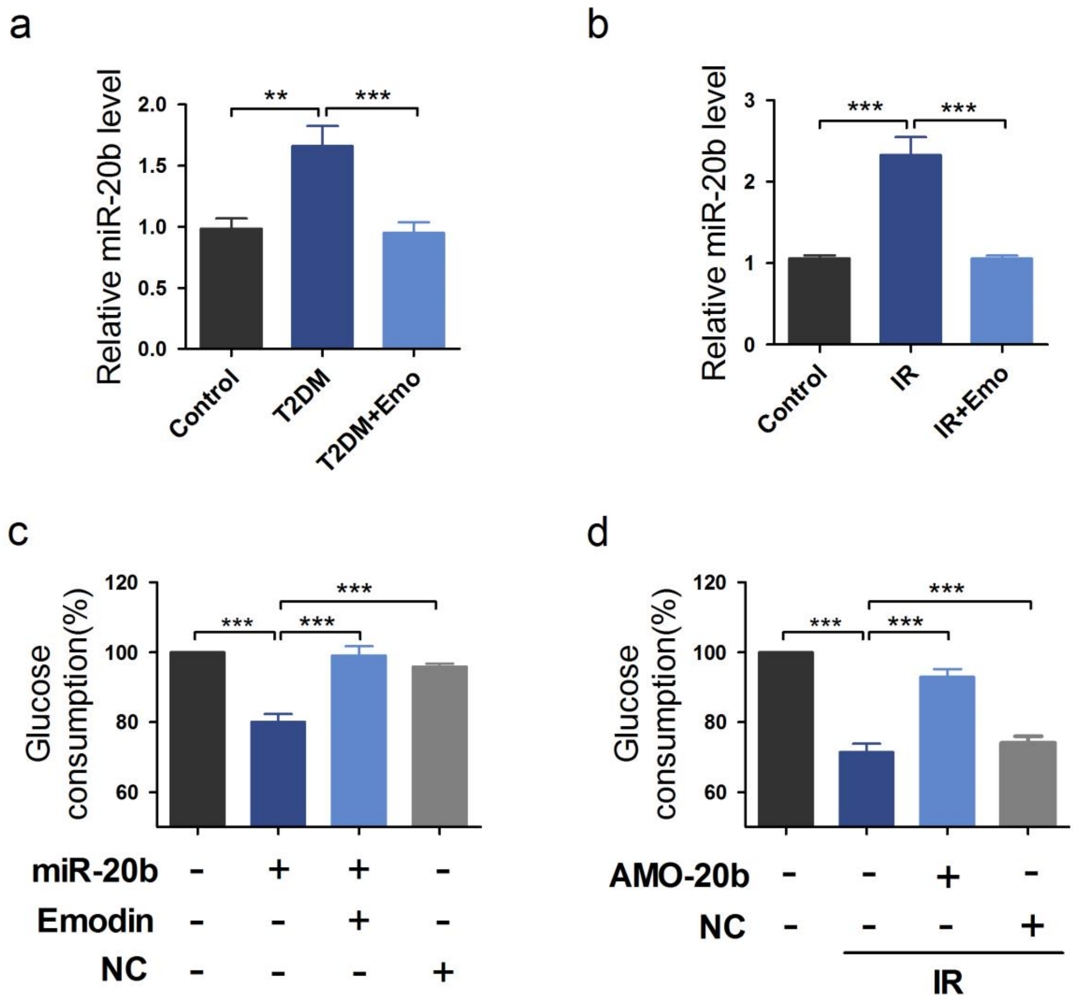

Figure 3. Emodin improves glucose metabolism via downregulating miR-20b. (a) MiR-20b expression detected by real-time PCR in rat skeletal muscle tissues, normalized to U6 expression, $^{**}p < 0.01$, $^{***}p < 0.001$, n = 10 batches in each group. **(b)** MiR-20b expression in L6 cells, normalized to U6 expression, $^{***}p < 0.001$, n = 15 batches for each group. **(c, d)** Glucose consumption in L6 cells. The degree of basal glucose consumption was set to 100%, $^{***}p < 0.001$. **(c)** n = 10, and **(d)** n=7 batches for each group.

ACCEPTED

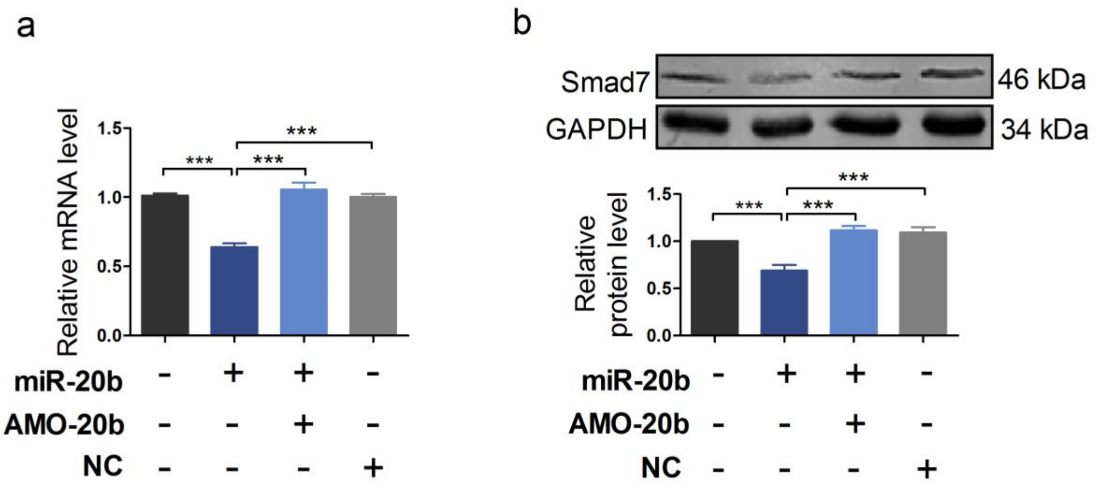

Figure 4. MiR-20b inhibits SMAD7 expression in L6 cells. Cells were transfected with miR-20b mimic, AMO-20b or negative control (NC). **(a)** SMAD7 mRNA expression in L6 cells, normalized to GAPDH, n = 13, ***$p < 0.001$. **(b)** SMAD7 protein levels detected by western blotting in L6 cells, normalized to GAPDH. The data were presented as relative levels, n = 8, ***$p < 0.001$.

ACCEPTED MANUSCRIPT

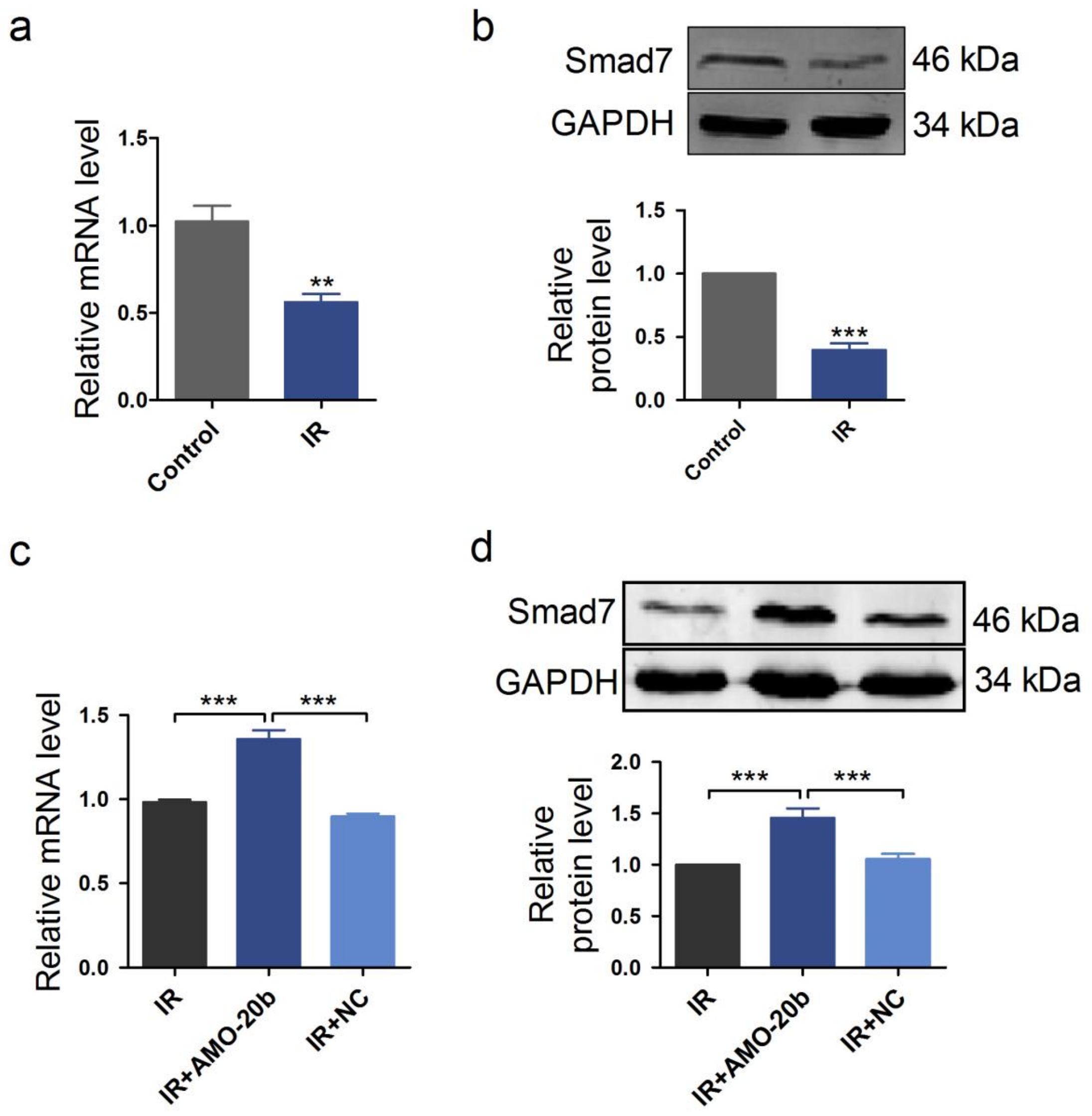

Figure 5. Effects of miR-20b on SMAD7 expression in insulin-resistant L6 cells. (a-b) SMAD7 mRNA and protein levels in L6 cells normalized to GAPDH, (a) n = 5, **p < 0.01 and (b) n = 6, ***p < 0.001. **(c, d)** Insulin-resistant L6 cells were transfected with AMO-20b or NC. SMAD7 mRNA and protein expression normalized to GAPDH, (c) n = 7, and (d) n = 8, ***p < 0.001.

ACCEPTED

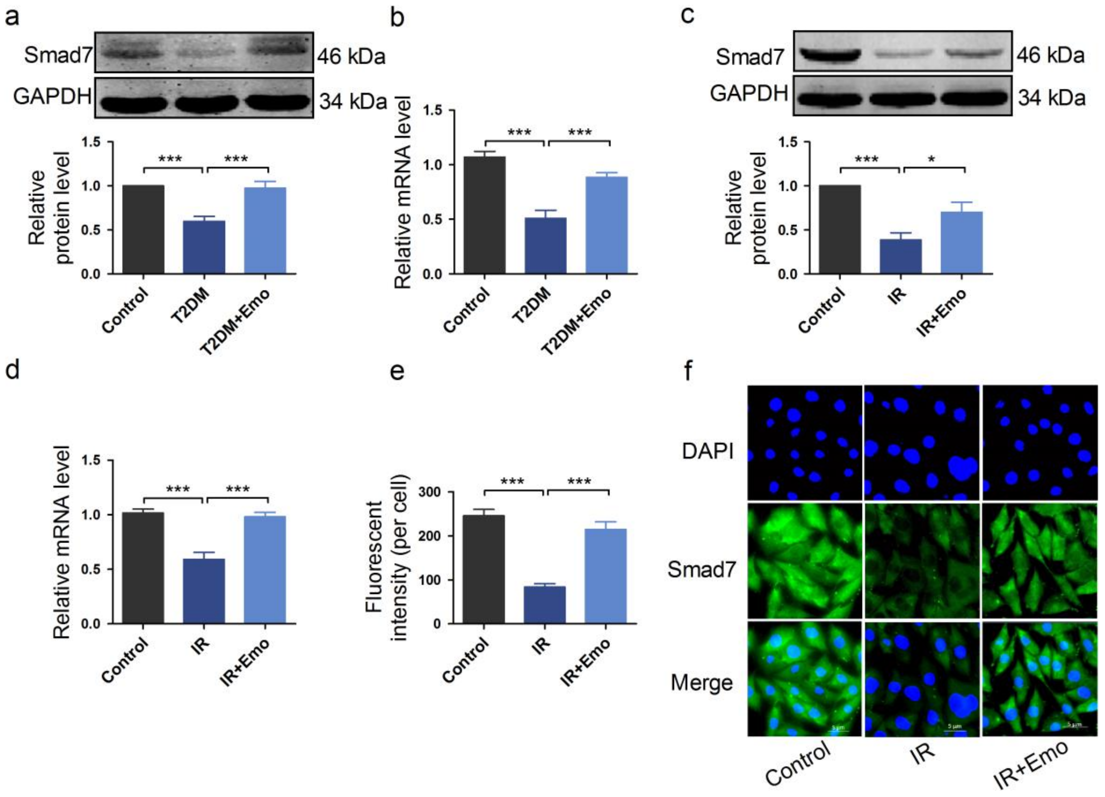

Figure 6. Effect of emodin on SMAD7 expression in skeletal muscle. (a, b) SMAD7 protein and mRNA expression normalized to GAPDH in rats skeletal muscle tissues, n = 8, ***$p < 0.001$. **(c, d)** SMAD7 protein and mRNA expression normalized to GAPDH in L6 cells, **(c)** n = 7 and **(d)** n = 8, *$p < 0.05$, ***$p < 0.001$. **(e, f)** Immunofluorescent staining showing the effects of emodin on the expression of SMAD7 protein (green) in insulin-resistant L6 cells. Nuclei were stained by DAPI (blue). Scale bar indicates 5 µm. **(e)** Statistic results of fluorescent intensity and **(f)** representative images are shown, n = 5 for each group, ***$p < 0.001$.

graphical abstract

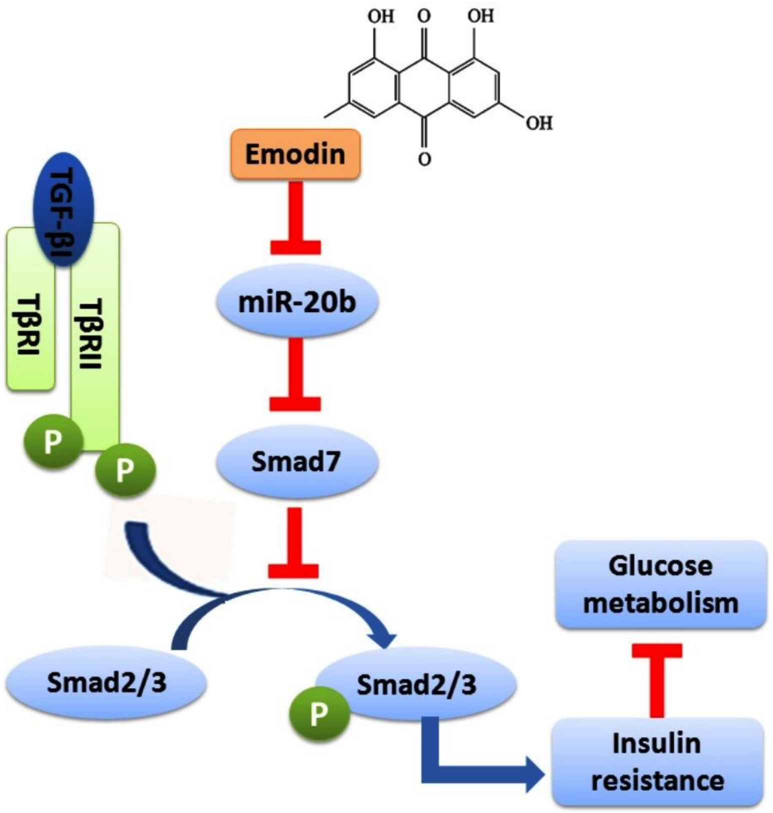

ACCEPTED
