# Supplementary material for: Emodin, a rising star in the treatment of glycolipid metabolism disorders: a preclinical systematic review and meta-analysis
Source: PeerJ. 2025 May 15;13:e19221. doi: 10.7717/peerj.19221 (PMC12085882; doi:10.7717/peerj.19221)
Supplement: Supplemental Information 20 [file peerj-13-19221-s020.zip › 8篇英文文献/Jianfeng Xue2010.pdf]

Fitoterapia 81 (2010) 173–177

Contents lists available at ScienceDirect

Fitoterapia

journal homepage: www.elsevier.com/locate/fitote

Anti-diabetic effects of emodin involved in the activation of PPARγ on high-fat diet-fed and low dose of streptozotocin-induced diabetic mice

Jianfeng Xue [a], Wenjun Ding [a,*], Yan Liu [b]

[a] College of Life Sciences, Graduate University of Chinese Academy of Sciences, No 19 A, Yu Quan Road, Beijing 100049, China
[b] Xinjiang Institute of Ecology and Geography of Chinese Academy of Sciences, Urumqi, Xinjiang 830011, China

ARTICLE INFO

Article history:
Received 29 March 2009
Accepted in revised form 11 August 2009
Available online 20 August 2009

Keywords:
Emodin
Peroxisomal proliferator-activated receptor γ (PPARγ)
Diabetic mice

ABSTRACT

Rheum palmatum Linn has been widely applied in the clinical treatment of diabetes mellitus. It has been found that emodin as the major bioactive component of *R. palmatum L* exhibits the competency to activate peroxisomal proliferator-activated receptor-γ (PPARγ) *in vitro*. So the aim of this study was to evaluate the anti-diabetic effects of emodin through the activation of PPARγ on high-fat diet-fed and low dose of streptozotocin (STZ)-induced diabetic mice. The diabetic mice were intraperitoneally injected with emodin for three weeks. No changes of food consumption and the body weight in emodin-treated mice were monitored daily during the entire experiment. At the end of experiment, the levels of blood glucose, triglyceride and total cholesterol in serum were significantly decreased after emodin treatment. However, serum high-density lipoprotein cholesterol (HDLc) concentration was significantly elevated. The glucose tolerance and insulin sensitivity in emodin-treated group were significantly improved. Furthermore, the results of quantitative RT-PCR analysis showed that emodin significantly elevated the mRNA expression level of *PPARγ* and regulated the mRNA expressions of *LPL*, *FAT/CD36*, *resistin* and *FABPs* (*ap2*) in liver and adipocyte tissues. No effects on the mRNA expressions of *PPARα* and PPARα-target genes were observed. Taken together, the results suggested that the activation of PPARγ and the modulation of metabolism-related genes were likely involved in the anti-diabetic effects of emodin.

© 2009 Elsevier B.V. All rights reserved.

1. Introduction

Diabetes mellitus is characterized by chronic hyperglycemia, disorders of lipid and carbohydrate and lipid metabolism, and microvascular pathology in the retina, renal glomerulus and peripheral nerves [1]. Therefore, numerous therapies in diabetes patients have been developed to maintain the normal blood glucose level [2]. Herb medicines have been widely applied in hyperglycemia treatment. However, the exact mechanisms of improving hyperglycemia and hypertriglyceridemia by herbs are not fully clear. In recent years, it has been reported that the effects of herbs on peroxisomal proliferator-activated receptors (PPARs) are associated with the regulation of glucose and lipid metabolism. For example, berberine inhibits 3T3-L1 adipocyte differentiation through the PPARγ pathways [3]. Extracts from *Astragalus membranaceus* and *Pueraria thomsonii* significantly activate PPARα and PPARγ [4]. Several isoprenols from herbs have the dual actions on both PPARα and PPARγ *in vitro* [5]. These findings are significant not only for the elucidation of herbal anti-diabetic mechanism but also for the development of novel PPARs agonists in diabetes therapy.

Rheum palmatum Linn is one of traditional Chinese herbs with multi-applications in anti-diabetes, anti-bacteria and anti-renal disorders [6]. A class of hydroxyanthraquinones

Abbreviations: ALT, Alanine aminotransferase; FABPs (ap2), Adipocyte fatty acid binding protein; FAT/CD36, Fatty acid translocase; GTT, Glucose tolerance test; HDLc, High-density lipoprotein cholesterol; ITT, Insulin tolerance test; LDLc, Low-density lipoprotein cholesterol; LPL, Lipoprotein lipase; LSD, Least significant difference; PPARs, Peroxisome proliferator-activated receptors; PPARγ, Peroxisome proliferator-activated receptor-γ; STZ, Streptozotocin; TZDs, Thiazolidinediones; UCP, Uncoupling protein.

* Corresponding author. Tel./fax: +86 10 88256290.
E-mail address: dingwj@gucas.ac.cn (W. Ding).

0367-326X/$ – see front matter © 2009 Elsevier B.V. All rights reserved.
doi:10.1016/j.fitote.2009.08.020

(HAQs), including emodin, rhein, aloe-emodin and chrysophanol have been identified as the major bioactive components of *R. palmatum L* [6]. Yang and coworkers have reported that emodin presents a very high binding affinity to PPARγ *in vitro* [7]. Therefore, the present study was performed to investigate the anti-diabetic effects of emodin which was involved in the activation of PPARγ on high-fat diet-fed and low dose of STZ-induced diabetic mice. Moreover, we evaluated the biochemical parameters in serum of diabetic mice.

2. Materials and methods

2.1. Animals and experimental procedures

Male C57/BL6J mice of 8-week-old were purchased from Laboratory Animal Center of the Academy of Military Medical Sciences (Beijing, China). The animals were maintained under standard conditions (12 h light–dark cycle, 24 °C) and had free access to water and standard laboratory chow. All mice were cared for in according to the standards of the Guide for the Care and Use of Laboratory Animals.

The inducement of diabetic animals was carried out according to a previous reported procedure [8]. In brief, the animals were fed with high-fat diet for 4 weeks before intraperitoneal (i.p) injection with 120 mg/kg streptozotocin (STZ, sigma, USA). An equal volume of vehicle was injected into the control rats ($n=8$). The blood glucose levels in serum were determined by a glucose measuring kit (Biosino Bio-technology and Sicence, China) using the glucose oxidase method. The mice with fasting glucose level higher than 11 mmol/L were considered as diabetic mice, and the diabetic mice with consecutive 10-day hyperglycemia (11 mmol/L or greater) were used for the experiment. The diabetic mice were randomly divided into two groups: emodin-treated group ($n=15$) and diabetic group ($n=10$). The untreated mice ($n=8$) were matched in age and weight as the control group. The mice in emodin-treated group were intraperitoneally injected with emodin (98% pure, Nanjing Zelang Medical Technology Co, China) (Fig. 1) at a dose of 1.5 mg/kg body weight daily for 3 weeks. The diabetic group was intraperitoneally given the vehicle solvent (sterilized 0.9% NaCl). Body weight and food consumption in each group were monitored daily. Blood glucose levels were checked every 4 days. Blood samples were obtained from the tail vein of the mice and blood glucose levels in serum were determined as described earlier.

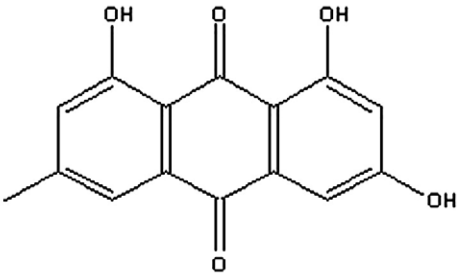

Fig. 1. Schematic structure of emodin.

2.2. Glucose tolerance test (GTT) and insulin tolerance test (ITT)

The glucose tolerance test (GTT) and insulin tolerance test (ITT) were carried out at the end of experiment. In the ITT, mice were intraperitoneally administrated with 0.5 U/kg body weight of bovine insulin (Sigma, USA) after 15 h fast. Blood samples (10 µl) were collected from a cut in the tail vein of the mice at 0, 15, 30, 45, 60, 90 and 120 min after insulin administration, respectively. In the GTT, mice were intraperitoneally injected with 2 g/kg body weight of D-glucose after 15 h fast. Blood samples (10 µl) were taken from a cut in the tail vein of the mice at 0, 30, 60, 90 and 120 min after glucose administration, respectively. The GTT and ITT were carried out on awake mice without anesthetization. The glucose levels in serum were determined as described earlier.

2.3. Blood biochemical analysis and tissue preparation

All animals were sacrificed after 3 weeks treatment of emodin. Blood samples (1 ml) were obtained from the abdominal vein with a microsyringe. Serum was separated at 3000 rpm for 10 min. Serum triglyceride, total cholesterol, high-density lipoprotein cholesterol (HDLc) and low-density lipoprotein cholesterol (LDLc) levels were measured by using corresponding commercially available kits (Beyotime Inc, China) and an OLYMPUS AU400 chemistry analyzer. Liver, subcutaneous adipocyte and skeletal muscle were immediately separated, collected and stored in liquid nitrogen.

2.4. Quantitative analysis of gene expression

Total RNA was extracted from these frozen liver, subcutaneous adipocyte and skeletal muscle by TRIzol Reagent (Invitrogen). Then 1 µg of total RNA was subjected to the reverse transcription reaction. The cDNA was used as a template to examine the mRNA expression levels of *PPARγ*, *PPARα*, *FAT/CD36*, *resistin*, *adiponectin*, *UCP2*, *UCP3*, *FABPs (ap2)* and *LPL* by using Syber green universal PCR master mix (Toyobo, Japan). The PCR cycle was as follows: initial denaturation at 95 °C for 5 min, followed by 40 cycles of denaturation at 95 °C for 30 s, annealing at 55 °C for 30 s and extension at 72 °C for 30 s. The amount of target genes was analyzed using the ΔCt method following the normalization through β-actin. The primers for target genes are shown in Table 1.

2.5. Statistical analysis

All data are presented as the mean ± the standard deviation. Non-parametric Kruskal–Wallis test was used to analyze the data from the groups. If significant differences were detected, the individual groups were compared with each other by the Mann–Whitney test by SPSS 13.0 software. The comparison of the changes in gene expression levels was performed using one way ANOVA followed by the least significant difference (LSD) for the multiple comparisons. A probability of value of $p<0.05$ was considered to denote a significant difference between groups.

Table 1
Primers for real-time PCR analysis.

Gene	Forward primer	Reverse primer
β-Actin	TTACTGCCCTGGCTCCTA	ACTCATCGTACTCCTGCTTG
PPARγ	ATCGAGGACATCCAAGAC	CAATCTGCCTGAGGTCTG
PPARα	TTCGCTATGAAGTTCAATGCCC	CCTATGTTTAGAAGGCCAGGCC
FAT/CD36	GCAAAACGACTGCAGGTCAAC	TGGTCCCAGTCTCATTTAGCCA
Resistin	TCAACTCCCTGTTTCCAAATGC	GCTCAAGACTGCTGTGCCTTCT
Adiponectin	GTATCGCTCAGCGTTCAGTGTG	TGCTGCCGTCATAATGATTCTG
UCP2	CAGGTCACTGTGCCCTTACCAT	CACTACGTTCCAGGATCCCAAG
UCP3	GGATTTGTGCCCTCCTTTCTG	AGATTCCCGCAGTACCTGGACT
FABPs (ap2)	TGAAGAGCATCATAACCCTG	CACATTCCACCACCAGCT
LPL	GCGCTCCATCCATCTCTTCAT	GGCAGAGCCCTTTCTCAAATG

3. Results and discussion

In the present study, we sought to evaluate the anti-diabetic effects of emodin on high-fat diet-fed and low dose of STZ-induced diabetic mice. We found that the body weight of STZ-treated mice was badly restricted after STZ administration and started to be lower than that of the untreated mice (Fig. 2A).

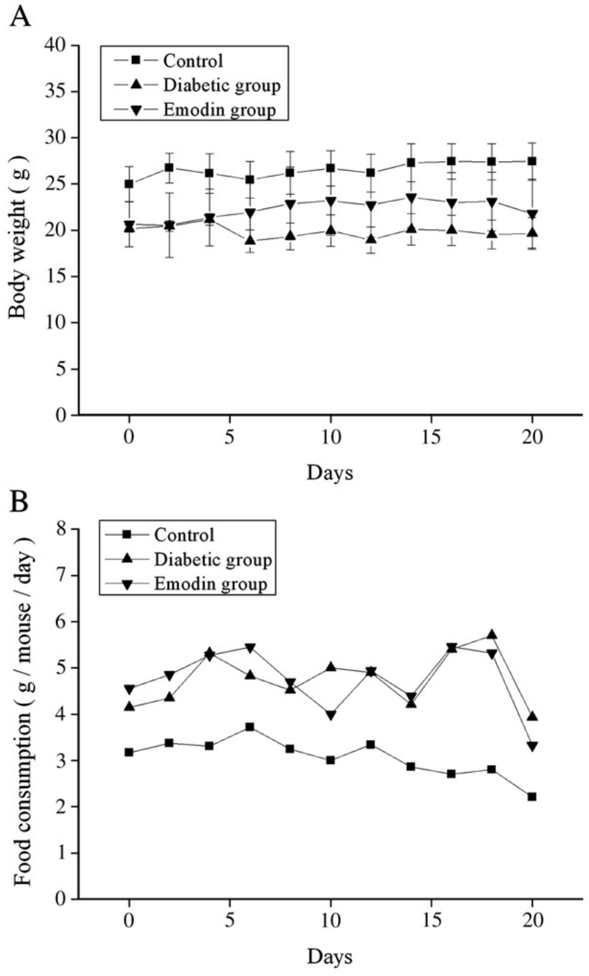

Fig. 2. Effects of emodin on body weight (A) and food consumption (B) in the control group, the diabetic group and the emodin-treated diabetic group (Emodin group). The results were represented as mean ± S.D.

From day 6 on, the body weight of emodin-treated mice was rescued by emodin treatment although there was no statistical significant difference between the emodin-treated group and the diabetic group (Fig. 2A). The food consumption and fluid intake (data not shown) in the diabetic group were higher than those of the controls (Fig. 2B).

Serum glucose level in emodin-treated group was significantly lower than that in diabetic group ($p < 0.01$). The concentrations of serum triglyceride and cholesterol were significantly decreased after emodin treatment. However, the level of serum HDLc in emodin-treated group was significantly increased compared to the diabetic group. No significant difference in LDLc was observed between the emodin-treated group and the diabetic group (Table 2). Moreover, we investigated the effects of emodin on the glucose homeostasis and insulin sensitivity in diabetic mice, the GTT and ITT were performed on day 21. As shown in Fig. 3A, the blood glucose level in the diabetic group increased to a maximum at 60 min, and then decreased slowly. In contrast, the fasting blood glucose in emodin-treated group declined significantly. To the ITT, the serum glucose level in diabetic group slightly decreased compared to the control group and the emodin-treated group (Fig. 3B). Herein, these results indicated that the glucose tolerance and insulin sensitivity in diabetic mice were significantly improved by emodin treatment. The ameliorations of emodin on glucose homeostasis and hyper-triglyceridemia in diabetic mice were agreement with the anti-diabetic and lipid-modulating effects of emodin in dyslipidaemic–diabetic rats induced by high-fat, low dose of STZ [9,10].

The RNA expression profiles of liver, subcutaneous adipocyte and skeletal muscle in mice were assayed to investigate the molecular mechanism of anti-diabetic effect of emodin (Table 3). Our results showed that emodin significantly increased the mRNA expressions of *PPARγ* in the liver of diabetic mice. In addition, we found that emodin treatment significantly elevated the expression of *LPL*, and significantly decreased the expression of *FAT/CD36* in liver compared to the diabetic group. It has shown that the mass of preheparin LPL is positively related to the insulin sensitivity [11]. The level of fatty acid in the liver is reduced by the reversed expression of *FAT/CD36* through controlling the influx. Therefore, the adjustment in the mRNA expressions of LPL and *FAT/CD36* by emodin contributed to the effects in the improvement of hyperglycemia and dyslipidemia. Adipose cells are a major target tissue for PPARγ agonists [12,13]. Emodin could promote the glucose uptake and differentiation

Table 2
Effects of emodin on serum glucose, triglyceride, cholesterol, HDLc and LDLc in the control group, the diabetic group and the emodin-treated diabetic group (mM).

Groups	*N*	Glucose	Triglyceride	Cholesterol	HDLc	LDLc
Control	8	5.42 ± 0.71	0.34 ± 0.02	1.94 ± 0.07	1.29 ± 0.06	0.19 ± 0.03
Diabetic group	10	18.21 ± 1.50**	0.56 ± 0.04*	2.11 ± 0.05*	1.00 ± 0.12*	0.26 ± 0.02*
Emodin-treated diabetic group	15	10.04 ± 3.20* ##	0.41 ± 0.02* #	2.00 ± 0.03#	1.17 ± 0.07#	0.25 ± 0.02*

**p* < 0.05 *vs* control, ***p* < 0.01 *vs* control, #*p* < 0.05 *vs* diabetic group, ##*p* < 0.01 *vs* diabetic group. The results were represented as mean ± S.D.

in 3T3-L1 pre-adipocyte through activating PPARγ *in vitro* [7]. In our study, emodin also had effects on the gene expression in the subcutaneous adipocyte tissue. Emodin elevated *FABPs* (*ap2*) and reduced the mRNA expression of *resistin*. The transcription of *FABPs* (*ap2*) is under the regulation of PPARs. *FABPs* (*ap2*) has been commonly used to evaluate the activity of PPARγ ligands [14]. It is suggested that *FABPs* (*ap2*) plays a critical role in dyslipidemia, insulin resistance and atherosclerosis in humans. Thus, the expression of *FABPs* (*ap2*) could be crucial in assaying whether emodin activates the *PPARγ* expression. Although the expression level of *resistin* was controversial to obesity, insulin resistance and glucose homeostasis in various animal models and in clinical investigations [15–17]. Many studies implicated that PPARγ agonists treatment could directly cause a significant decrease in *resistin* concentration [18,19]. In consistent to this, we found that emodin administration also decreased the mRNA expression level of *resistin* in subcutaneous adipocyte [18,19]. The elevation of *PPARγ* mRNA was not noted, but the alterations in the expressions of *FABPs* (*ap2*) and *resistin* suggested that emodin modulates PPARγ-downstream genes of the subcutaneous adipocyte in emodin-treated mice, which may be partly through PPARγ pathway.

In various diseases or cell models, emodin has exhibited some other features like influencing reactive oxygen species generation or adjusting the activity of some protein kinases [20,21]. So, it is hard to exclude these traits from anti-hyperglycemic effects of emodin. It is possible that some other mechanisms may also contribute to the anti-diabetic properties of emodin besides PPARγ activation. Our further research will focus on this.

In summary, our results showed that emodin ameliorated the symptoms of diabetic animals, and this effect was likely associated with the regulation of PPARγ pathway. These results together with the previous observations that emodin is a potent PPARγ agonists could render it as an attractive therapeutic agent for managing diabetes mellitus.

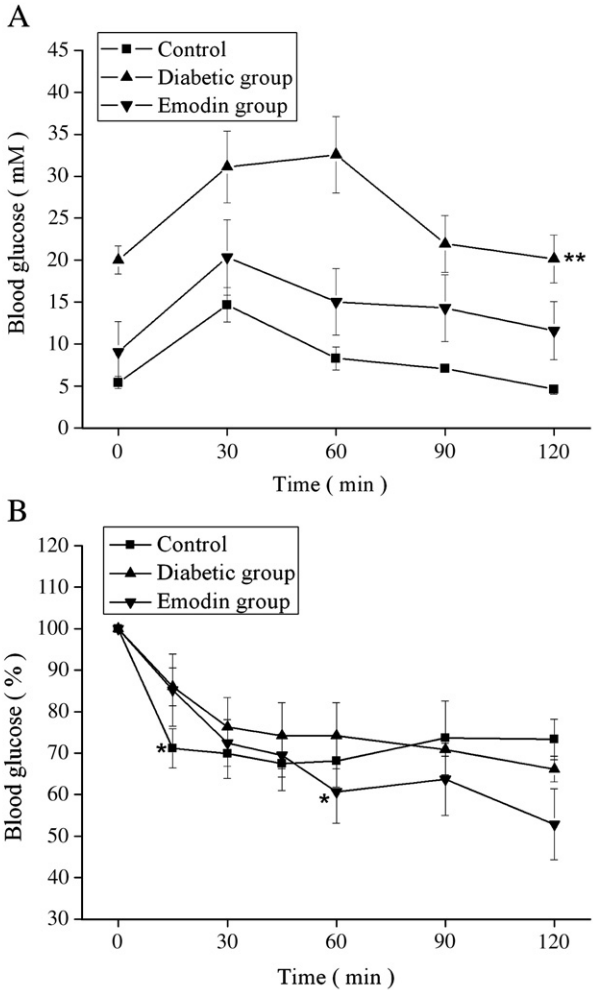

Fig. 3. Effects of emodin on the GTT (A) and ITT (B) in the control group, the diabetic group and the emodin-treated diabetic group (Emodin group). In ITT, the serum glucose level was expressed as the ratio of glucose concentrations of each time point to the baseline which was designated as 100%. The results were represented as mean ± S.D. **p* < 0.05, ***p* < 0.01 *vs*. the diabetic group.

Table 3
Transcriptional changes of target genes examined by real-time PCR.

Tissues	Genes	Diabetic group	Emodin-treated group
Liver	*PPARγ*	0.45 ± 0.04**	0.61 ± 0.11** #
	LPL	0.67 ± 0.08**	0.86 ± 0.07 #
	FAT/CD36	3.65 ± 0.46**	1.27 ± 0.06 ##
	UCP2	1.49 ± 0.07**	1.19 ± 0.03** ##
Subcutaneous adipocyte	*PPARγ*	0.81 ± 0.05*	0.87 ± 0.02
	Resistin	1.37 ± 0.13*	0.88 ± 0.15 ##
	Adiponectin	0.82 ± 0.09*	0.91 ± 0.05
	FABPs (*ap2*)	1.17 ± 0.11**	1.58 ± 0.13 ##
Skeletal muscle	*PPARα*	0.42 ± 0.07**	0.39 ± 0.12**
	UCP2	0.73 ± 0.19	0.64 ± 0.08**
	UCP3	0.83 ± 0.18	0.86 ± 0.07

The relative quantification of target genes was presented as fold increase compared to the control group which was normalized through β-actin to one-fold. **p* < 0.05 *vs* control, ***p* < 0.01 *vs* control, #*p* < 0.05 *vs* diabetic group, ##*p* < 0.01 *vs* diabetic group.

Acknowledgements

This work was supported by “Hundred Talents Program” of CAS, the National Natural Sciences Foundation of China (Grant No. 20871120).

References

[1] Vinik AI, Vinik E. Am J Manag Care 2003;9:63.
[2] Li M, Smee JJ, Ding W, Crans DC. J Inorg Biochem 2009;103:585.
[3] Huang C, Zhang Y, Gong Z, Sheng X, Li Z, Zhang W, et al. Biochem Biophy Res Co 2006;348:571.
[4] Shen P, Liu MH, Ng TY, Chan YH, Yong EL. J Nutr 2006;136:899.
[5] Takahashi N, Kawada T, Goto T, Yamamoto T, Taimatsu A, Matsui N, et al. FEBS Lett 2002;514:315.
[6] Tong S, Yan J. J Chroma A 2007;1176:163.
[7] Yang Y, Shang W, Zhou L, Jiang B, Jin H, Chen M. Biochem Biophy Res Co 2007;353:225.
[8] Lian J, Xiang Y, Guo L, Hu W, Ji W, Gong B. Scand J Lab Anim Sci 2007;34:21.
[9] Yang Lh, Fe Lu, Dong H, Xu Lu. World Chin J Digestol 2005;13:608.
[10] Zhao XY, Qiao GF, Li BX, Chai LM, Li Z, Lu YJ, et al. Clin Exp Pharmacol Physiol 2009;36:29.
[11] Hanyu O, Miida T, Kosuge K, Ito T, Soda S, Hirayama S, et al. Clinica Chimica Acta 2007;384:118.
[12] Laplante M, Sell H, MacNaul KL, Richard D, Berger JP, Deshaies Y. Diabetes 2003;52:291.
[13] Skopkova M, Penesova A, Sell H, Radikova Z, Vlcek M, Imrich R, et al. Obesity 2007;15:2396.
[14] Thompson GM, Trainor D, Biswas C, LaCerte C, Berger JP, Kelly LJ. Anal biochem 2004;330:21.
[15] Janke J, Engeli S, Gorzelniak K, Luft FC, Sharma AM. Obesity 2002;10:1.
[16] Steppan CM, Bailey ST, Bhat S, Brown EJ, Banerjee RR, Wright CM, et al. Nature 2001;409:307.
[17] Lee JH, Chan JL, Yiannakouris N, Kontogianni M, Estrada E, Seip R, et al. J Clin Endocrinol Metab 2003;88:4848.
[18] Bajaj M, Suraamornkul S, Hardies LJ, Pratipanawatr T, DeFronzo RA. Int J Obes Relat Metab Disord 2004;28:783.
[19] Hartman HB, Hu X, Tyler KX, Dalal CK, Lazar MA. J Biol Chem 2002;277:19754.
[20] Lai GH, Zhang Z, Sirica AE. Mol Cancer Ther 2003;2:265.
[21] Su YT, Chang HL, Shyue SK, Hsu SL. Biochem Pharm 2005;70:229.
