# Supplementary material for: Emodin, a rising star in the treatment of glycolipid metabolism disorders: a preclinical systematic review and meta-analysis
Source: PeerJ. 2025 May 15;13:e19221. doi: 10.7717/peerj.19221 (PMC12085882; doi:10.7717/peerj.19221)
Supplement: Supplemental Information 20 [file peerj-13-19221-s020.zip › 8篇英文文献/Sameer Abu Eid2017.pdf]

European Journal of Pharmacology (xxxx) xxxx–xxxx

Contents lists available at ScienceDirect

European Journal of Pharmacology

journal homepage: www.elsevier.com/locate/ejphar

Emodin, a compound with putative antidiabetic potential, deteriorates glucose tolerance in rodents

Sameer Abu Eid[a], Michael Adams[b,c], Thomas Scherer[a], Héctor Torres-Gómez[b], Martina Theresa Hackl[a], Mairam Kaplanian[a], Rainer Riedl[b], Anton Luger[a], Clemens Fürnsinn[a,*]

[a] Division of Endocrinology & Metabolism, Department of Medicine III, Medical University of Vienna, Währinger Gürtel 18-20, 1090 Vienna, Austria
[b] Zurich University of Applied Sciences, Institute of Chemistry and Biological Chemistry, Center for Organic and Medicinal Chemistry, Einsiedlerstrasse 31, 8820 Wädenswil, Switzerland
[c] Bacoba AG, Einsiedlerstrasse 25, 8820 Wädenswil, Switzerland

ARTICLE INFO

Chemical compounds studied in this article:
Emodin (PubChem CID: 3220)

Keywords:
Emodin
Glucose
Appetite
Body Weight
Diabetes

ABSTRACT

Emodin is found in remedies from Traditional Chinese Medicine. Since antihyperglycaemic action was observed in rodents, non-scientific sources advertise emodin intake as a natural cure for diabetes. Emodin was admixed to high fat-food of obese mice at two doses (2 and 5 g/kg; daily emodin uptake 103 and 229 mg/kg). Comparison was made to ad libitum fed and to food restricted control groups, the latter showing the same weight gain as the corresponding emodin-treated groups. Emodin blunted food intake by 6% and 20% for the low and high dose, which was accompanied by proportionate reductions in weight gain. Emodin reduced blood glucose relative to freely feeding controls, but comparison to weight-matched controls unmasked deterioration, rather than improvement, of basal glycaemia (mmol/l: fed ad libitum, 9.5 ± 0.4; low emodin, 9.4 ± 0.3, weight-matched, 8.2 ± 0.3; high emodin, 7.2 ± 0.4, weight-matched, 6.1 ± 0.3; $P < 0.01$, emodin vs weight-matched) and glucose tolerance (area under the curve, min*mol/l: fed ad libitum, 2.01 ± 0.08; low emodin, 1.97 ± 0.12, weight-matched, 1.75 ± 0.03; high emodin, 1.89 ± 0.07, weight-matched, 1.65 ± 0.05; $P < 0.0002$, emodin vs weight-matched). An insulin tolerance test suggested insulin desensitisation by prolonged emodin treatment. Furthermore, a single oral emodin dose did not affect glucose tolerance in obese mice, whereas intravenous injection in rats suggested a potential of emodin to acutely impair insulin release. Our results show that the antihyperglycaemic action of emodin as well as associated biochemical alterations could be the mere consequences of a spoilt appetite. Published claims of antidiabetic potential via other mechanisms evoke the danger of misuse of natural remedies by diabetic patients.

1. Introduction

In Traditional Chinese Medicine, numerous remedies derived from plants and animals are used for the treatment of type 2 diabetes mellitus. The anthraquinone derivative emodin (1,3,8-trihydroxy-6-methylanthra-9,10-quinone), which is found in roots and barks of many plants, is among the putative active ingredients believed to account for antidiabetic effects (Li et al., 2004; Xie and Du, 2011). Under academic examination, emodin exhibited a broad pharmacology with promising evidence for laxative, antiinflammatory, anticancer and other beneficial activities (Shrimali et al., 2013; Srinivas et al., 2007), which included the lowering of blood glucose in hyperglycaemic rodents (Feng et al., 2010; Wang et al., 2012; Xue et al., 2010; Zhao et al., 2009). Although the evidence is purely experimental, antihyperglycaemic action in rodents is meanwhile extensively cited by vendors to promote emodin-containing herbal products as a natural cure for diabetes.

Studies aiming to understand the mechanism(s) responsible for emodin-induced lowering of blood glucose gave rise to a surprising multitude of suggested molecular targets and alleged biochemical mechanisms. Some reports proposed that emodin acts via mechanisms attributed to clinically established antidiabetic drugs, providing evidence for a thiazolidinedione-like mode of action by binding to peroxisome proliferator-activated receptor-γ (PPARγ) and promotion of adipocyte differentiation (Chen et al., 2012; Yang et al., 2007), as well as for a metformin-like mode of action via inhibition of mitochon-

* Corresponding author.
E-mail addresses: sameid73@gmail.com (S. Abu Eid), adams@bacoba.ch (M. Adams), thomas.scherer@meduniwien.ac.at (T. Scherer), hectormanuel.torres-gomez@zhaw.ch (H. Torres-Gómez), martina.hackl@meduniwien.ac.at (M.T. Hackl), n1635226@students.meduniwien.ac.at (M. Kaplanian), rira@zhaw.ch (R. Riedl), anton.luger@meduniwien.ac.at (A. Luger), clemens.fuernsinn@meduniwien.ac.at (C. Fürnsinn).

http://dx.doi.org/10.1016/j.ejphar.2017.01.022
Received 11 August 2016; Received in revised form 17 January 2017; Accepted 17 January 2017
0014-2999/ © 2017 Published by Elsevier B.V.

drial complex 1, impairment of cell respiration and activation of AMP-activated protein kinase (AMPK) (Song et al., 2013). Again other studies attributed emodin's antihyperglycaemic activity to modulation of the sterol regulatory element binding protein (SREBP) pathway (Li et al., 2016) or to direct interaction with the enzymes 11β-hydroxysteroid dehydrogenase (11β-HSD) (Feng et al., 2010; Wang et al., 2012), glycogen synthase kinase-3β (GSK-3β) (Gebhardt et al., 2010), or acetyl-CoA carboxylase (ACC) (Chao et al., 2010). And finally, improvement of glucose homeostasis has also been related to effects on pancreatic β-cells, in which emodin was claimed to upregulate L-type calcium channels that are essential mediators of insulin release (Zhao et al., 2009).

In the light of such versatile information about potential molecular targets and pathways, it is puzzling how little efforts have been made to evaluate the straightforward possibility that improvements in insulin sensitivity and glucose homeostasis could result from reductions in appetite and body weight, as they have also been observed in emodin-treated rodents (Feng et al., 2010; Li et al., 2016; Lu et al., 2014; Wang et al., 2012). This question has been addressed in the present study, which revealed that the beneficial effects of subchronic emodin treatment on glucose homeostasis are entirely dependent on blunted appetite and weight gain. Our results even unmask that emodin causes deterioration rather than improvement of glucose tolerance, when comparison is made to restrictedly fed, weight matched control animals.

2. Material and methods

2.1. Chemicals

Emodin was purchased from FWD Fine Chemicals Limited (Shanghai, China). Before use, 98% purity as stated by the supplier was confirmed in-house by nuclear magnetic resonance.

2.2. Animal husbandry

Six weeks-old male C57BL/6J mice were purchased from Charles River Laboratories, Sulzfeld, Germany. Mice were housed in polycarbonate cages provided with wood-based bedding (Hygienic Animal Bedding, J.Rettenmaier & Söhne, Rosenberg, Germany), under constant room temperature and with an artificial 12 h dark/12 h light cycle. Unless stated otherwise, mice had free access to tap water and high fat diet (HFD; 60% of calories as fat; diet D12492 from Research Diets Inc., New Brunswick, NJ, USA).

Male Sprague-Dawley rats were from the breeding facilities of the Division for Laboratory Animal Science and Genetics, Medical University of Vienna (Himberg, Austria) and were used at a body weight of approximately 400 g. Husbandry was as described for mice, except that rats were fed a conventional rodent chow diet (sniff R/M-H; sniff Spezialdiäten GmbH; Soest, Germany).

The study was in line with effective national and international guidelines and law, and all procedures followed the principles of good laboratory animal care. The protocol was approved by the Austrian Federal Ministry of Science, Research and Economy.

2.3. Effects of a single emodin dose in obese mice

At an age of 15 weeks and after 9 weeks of HFD feeding, 36 mice were allocated to 3 wt-matched groups (n=12 each). They were fasted for 10 h before the tip of the tail was pricked with a needle for the measurement of blood glucose with a portable glucose meter (OneTouch, LifeScan, Milpitas, CA, USA; means of duplicate measurements). Immediately thereafter, mice received by gavage a suspension of 100 or 250 mg/kg emodin, or the vehicle (0.5% sodium carboxymethyl cellulose; 5 µl/g body weight) with the doses corresponding to daily oral doses used for repeated treatment in the present study as well as in an earlier study (Feng et al., 2010). Thirty min after dosing, an intraperitoneal glucose tolerance test (IPGTT) was started. Mice were injected with glucose solution (33% wt/vol; 1 g/kg) and the resulting excursion of blood glucose was documented by measurements immediately before (0 min) as well as 20, 40, 60, 90, and 120 min after glucose administration.

To assure that relevant actions are not missed due to parenteral glucose administration or due to a slow onset of emodin action, oral glucose tolerance tests (OGTTs) were performed. After 9 weeks on HFD 10 obese mice were fasted 10 h and fed 250 mg/kg emodin as described above. Two oral doses of a 33% (w/v) glucose solution (2 g/kg) were administered by gavage 30 min and 240 min after administration of emodin. The experiment was performed in two runs that were one week apart. In each run, half of the mice received emodin and vehicle, providing a paired data set.

2.4. Effects of subchronic emodin treatment in obese mice

After 10 weeks of HFD feeding, one mouse out of 60 was excluded for its disproportionally low body weight. The 59 remaining mice were divided into 5 wt-matched groups to study the effects of subchronic emodin treatment. One group continued on HFD ad libitum (referred to as freely feeding controls; n=11); two groups had free access to HFD with either 2 or 5 g/kg emodin admixed (referred to as low and high dose emodin; n=12 each); and two groups were subjected to restricted feeding with HFD, aiming at rates of weight gain as seen in the low and high dose emodin groups (referred to as food restricted controls; n=12 each). The food admixtures of 2 and 5 g/kg emodin resulted in average daily intake of 103 and 229 mg/kg emodin, which resembles the doses previously associated with antihyperglycaemic action (Feng et al., 2010). Food intake and body weight were documented every 3–4 days. Furthermore, immediately before as well as 24 and 51 days after starting the treatment, fat mass and lean mass of each mouse were determined by nuclear magnetic resonance (EchoMRI, Houston, TX, USA).

Mice on low dose emodin, their corresponding food restricted controls, and half of the freely fed controls were examined in an IPGTT on days 21 and 48 of treatment. High dose emodin mice, their food restricted controls, and the remaining freely fed controls were tested one day later (days 22 and 49). Mice were fasted for 10 h and basal blood glucose was measured. Twenty min later, mice were injected with glucose to start the IPGTT. Detailed procedures were as in the IPGTT after administration of a single emodin dose (see chapter 2.3).

On day 29 of emodin treatment, mice on high dose emodin, their food restricted controls, and the freely fed controls were subjected to an insulin tolerance test (ITT). Food was withdrawn 1 h before blood glucose was measured and 0.75 U/kg insulin was injected intraperitoneally (NovoRapid, Novo Nordisk, Bagsvaerd, Denmark, diluted with saline; injected volume, 3 µl/g). Further measurements of blood glucose were made 15, 30, 45, and 60 min after insulin injection. In mice that showed a blood glucose value < 2.2 mmol/l, the ITT was immediately terminated by an intraperitoneal injection of 33% glucose solution.

On day 56 of treatment, mice were killed in the fed state with an overdose of an inhalation anaesthetic (Sevoflurane, Sigma-Aldrich, St. Louis, MO, USA). Blood was collected by heart puncture and plasma was stored at −20 °C for the later measurement of plasma insulin (Ultrasensitive Mouse Insulin ELISA from Mercodia, Uppsala, Sweden) and of plasma emodin. Emodin was measured by ultra-performance liquid chromatography tandem mass spectrometry (UPLC-MS/MS) from plasma samples of 100 µl after spiking with aloe emodin (used as internal standard), followed by extraction and reconstitution to a volume of 500 µl. Recovery of the internal standard was 1.0 ± 0.1 (mean ± S.D.). Procedures are outlined in further detail in Supplementary file S1.

2.5. Effects of a single emodin dose in rats

The claim of emodin's anti-diabetic potential is predominantly based on studies on orally treated mice (Feng et al., 2010; Li et al., 2016), but there are also reports on emodin-treated rats and on intravenous emodin administration (Song et al., 2013; Zhao et al., 2009). Hence, an additional experiment was designed to detect potential glucose lowering effects that might be species-specific or restricted to parenteral administration. At an age of approximately 12 weeks, rats were surgically provided with permanent catheters into the jugular vein, which allowed intravenous injections and frequent sampling of blood from the freely moving animal (Steffens, 1969). After at least one week for recovery, the rats had regained their pre-surgical body weight and were fasted for 10 h. Emodin dissolved in DMSO was admixed to a 9-fold larger volume of 5% Tween 80 (in saline) under vigorous shaking. Immediately thereafter, a blood sample was withdrawn and the vehicle alone or 2 mg/kg emodin, an intravenous dose previously associated with glucose lowering in mice (Song et al., 2013), was injected slowly via the venous catheter. The huge difference in emodin dosing for intravenous (this experiment) versus oral administration (Chapters 2.3 and 2.4) is explained by emodin's poor oral bioavailability (Liu et al., 2010; Shia et al., 2010). Twenty min later, another blood sample was taken, followed by intravenous injection of 1 g/kg glucose (33%, wt/vol). Further blood samples were collected 2, 5, 10, 15, 20, 30, 45, and 60 min after glucose administration. Blood samples (0.2 ml) were used for measurement of blood glucose and plasma insulin (OneTouch portable glucose meter, and Ultrasensitive Rat Insulin ELISA from Mercodia, Uppsala, Sweden). The K value indicative of the rate of net glucose clearance was calculated from the blood glucose values collected during the initial 20 min after glucose injection.

2.6. Statistical procedures

Results are given as means ± S.E.M.. *P*-Values were calculated by two way ANOVA with post hoc testing according to Tukey, or by two-tailed paired or unpaired Student´s *t*-tests. Multiple testing versus the same control group was corrected according to Bonferroni-Holm. A $P < 0.05$ was considered significant.

3. Results

3.1. Effects of subchronic emodin treatment in obese mice (Figs. 1–4)

3.1.1. Plasma emodin (Fig. 1)

While no emodin was detectable in plasma from control mice, the median plasma concentrations were 78 and 200 nmol/l for mice receiving the low and the high dose of emodin. As plasma was sampled in the fed state, the distinctly deviating plasma emodin concentration observed in one mouse from the high dose group (1.6 µmol/l) could have resulted from food intake shortly before killing (Fig. 1; also depicting the structure of emodin).

3.1.2. Food intake, body weight, and fat mass (Fig. 2; and Supplementary file S2)

Emodin dose-dependently reduced food intake, which was more pronounced during the first 20 days (Fig. 2A). Effects on weight gain seemed to be the plain consequence of different food intake without emodin-specific effects on energy expenditure or food efficiency (weight gain per food consumed), because a comparable calorie reduction without emodin treatment had the same effects on weight and body composition (Supplementary file S2). Body weight of mice treated with low dose emodin significantly fell behind that of freely feeding controls and remained lower until the end of the study. Mice receiving high dose emodin showed an absolute decrease in body weight during the first 20 days of treatment ($P < 0.005$). Thereafter, their body weight increased so that they were back to their starting values on day 45, but for the period from day 21–45 there was still a trend towards less weight gain than in animals fed ad libitum (P=0.051; Fig. 2B). The effects on body weight were predominantly due to changes in fat mass, which accounted for grossly 80% of the observed differences in total body mass (Fig. 2C).

3.1.3. Blood glucose and glucose tolerance (Fig. 3)

After 21/22 days of treatment fasting glycaemia, as determined 20 min before the IPGTT, was unaffected in the low dose emodin group as well as in the corresponding restrictedly fed group, whereas high dose emodin and the corresponding food restriction caused significant decreases of similar extent (Fig. 3A, left graph). After more prolonged treatment (on day 48/49) basal glycaemia was reduced in mice exposed to food restriction (−13% and −35% vs. controls fed ad libitum). This glucose lowering effect of reduced food consumption was blunted in emodin treated mice, which showed higher basal glycaemia than in their respective restrictedly fed controls (P=0.01; Fig. 3A, right graph).

The effects on glucose tolerance resembled those on basal glycaemia. In the first IPGTT performed after 21/22 days, restricted feeding was associated with marked and dose dependent improvement of glucose tolerance. This beneficial effect of reduced food consumption and lower body weight showed a trend to be less pronounced in animals treated with emodin (Fig. 3B, left graph). In the second IPGTT, which followed after 48/49 days of treatment, a beneficial effect of reduced feeding alone was likewise obvious, but in spite of similar reductions in calorie consumption, the emodin fed groups exhibited no improvement of glucose tolerance versus freely feeding control mice. In other words, emodin treatment completely abrogated the beneficial effect of weight loss (P=0.0002; Fig. 3B, right graph). The full glucose excursion curves from the IPGTTs are depicted in Fig. 3C.

3.1.4. Plasma insulin and insulin tolerance (Fig. 4)

On day 56, plasma insulin was reduced only in high dose emodin treated mice and their food restricted controls. There were no significant differences in plasma insulin between emodin treated mice and their corresponding restrictedly fed controls (Fig. 4A).

In the ITT, the initial fall in blood glucose is regarded as indicative of insulin sensitivity, whereas counterregulatory responses will interfere with insulin action at glucose concentrations in the range of 4 mmol/l or lower (Ayala et al., 2010). Restrictedly fed control mice showed a steeper decrease in blood glucose than their high dose emodin-treated or freely feeding counterparts during the first 15 min of the ITT, which indicated improved insulin sensitivity (Fig. 4B). In all groups, the rate of decrease in blood glucose became less distinct during the further course of the ITT, but this occurred at an earlier stage in the restrictedly fed group, obviously reflecting counterregulation in response to hypoglycaemia. Whereas all animals from the other groups completed the ITT, the tests of 3 mice belonging to the restricted feeding group had to be discontinued because of blood glucose values below 2.2 mmol/l (once at 30 min, twice at 45 min).

3.2. Effects of a single emodin dose in obese mice (Fig. 5)

Although glycaemia tended to be lower during the IPGTT in emodin-fed than in control mice (7–8% reduction in the AUC; Fig. 5A), statistical significance was not obtained at any time point of the test. A single dose of emodin likewise failed to affect oral glucose tolerance 30 min and 4 h after administration (Fig. 5B). Our results therefore do not provide evidence for an effect of acute emodin administration on glucose tolerance in mice.

3.3. Effects of a single emodin dose in rats (Fig. 6)

Glucose tolerance was modestly, but significantly, impaired after an intravenous dose of emodin in lean rats with a 13% increase of the AUC

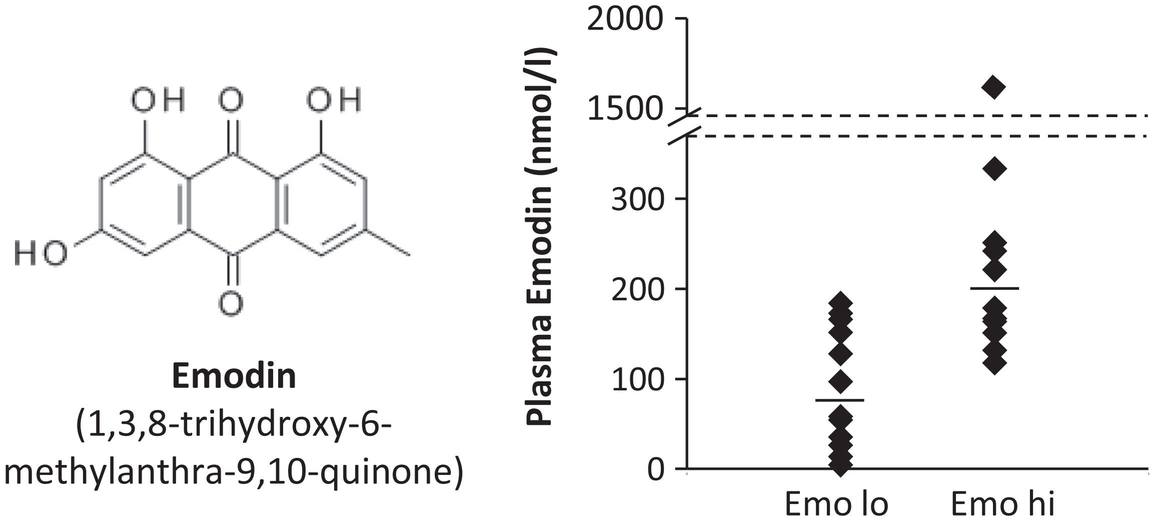

Fig. 1. Plasma emodin concentrations in treated obese mice. Structure of emodin (left) and its circulating concentration in plasma from mice with free access to high fat diet with admixtures of 2 or 5 g/kg emodin (Emo lo and Emo hi; right). Shown are individual values (diamonds) and medians (short vertical lines); n=12 each.

as well as a 21% reduction of the K-value (a readout parameter for the net rate of glucose clearance; Fig. 6). The impairment of glucose clearance was accompanied by a markedly blunted glucose induced excursion of plasma insulin (AUC for insulin reduced by 37%).

4. Discussion

Subchronic emodin treatment indisputably lowered blood glucose in our obese mice, which formally confirms previous evidence. Nevertheless, our study unmasks important limitations of emodin's true antihyperglycaemic activity, showing that the decrease in glycaemia was entirely driven by and dependent on reductions of appetite, body weight, and adiposity. To analyse the causal interdependence of changes in food intake, weight gain and blood glucose, we subjected groups of control mice to restricted feeding resulting in the same weight changes as seen in their emodin treated counterparts. Although our procedure formally differs from pair feeding (the latter led by identical food intake rather than by parallel weight gain), the difference proved irrelevant, because the amount of food deliberately consumed by emodin treated mice did not differ from the amount required to maintain similar body weight by restricted feeding. This strongly suggests that emodin's influence on body weight and adiposity was the plain consequence of reduced food consumption. The precise cause of their spoilt appetite cannot be deducted from our results, but diminished appetite is known to accompany almost any type of sickness and discomfort in laboratory rodents as it could arise from any unspecific side effect of the employed emodin doses. Aversion to the taste of emodin-containing food is an unlikely possibility, since similar or even more pronounced decreases in food intake have been reported for obese mice receiving comparable doses of emodin by gavage or by intraperitoneal injection (Feng et al., 2010; Li et al., 2016; Wang et al., 2012).

Along with diminished calorie consumption, emodin ameliorated obesity and the associated deterioration of glucose homeostasis in C57BL/6J-mice with free access to HFD. At variance to this, comparison to food restricted, weight-matched controls unmasked not only lack of intrinsic glucose lowering activity, but even a significant emodin induced deterioration of glucose homeostasis. The latter finding is in opposition to results from genetically obese ob/ob-mice, in which a beneficial action of emodin persisted in comparison to pair fed controls (Wang et al., 2012). In the ob/ob-mice, however, reductions in feeding and body weight surprisingly tended to worsen rather than to improve glucose homeostasis, which contrasts with the broadly agreed beneficial influence of weight loss on glycaemia and insulin sensitivity.

Our insulin tolerance test not only confirmed the well-known amelioration of insulin resistance with weight loss, but also hinted at insulin resistance as a potential cause of emodin's adverse effects on blood glucose. In spite of the evidence for increased glycaemia and insulin resistance, emodin treatment was not associated with elevated plasma insulin. Hence, it cannot be ruled out that impairment of

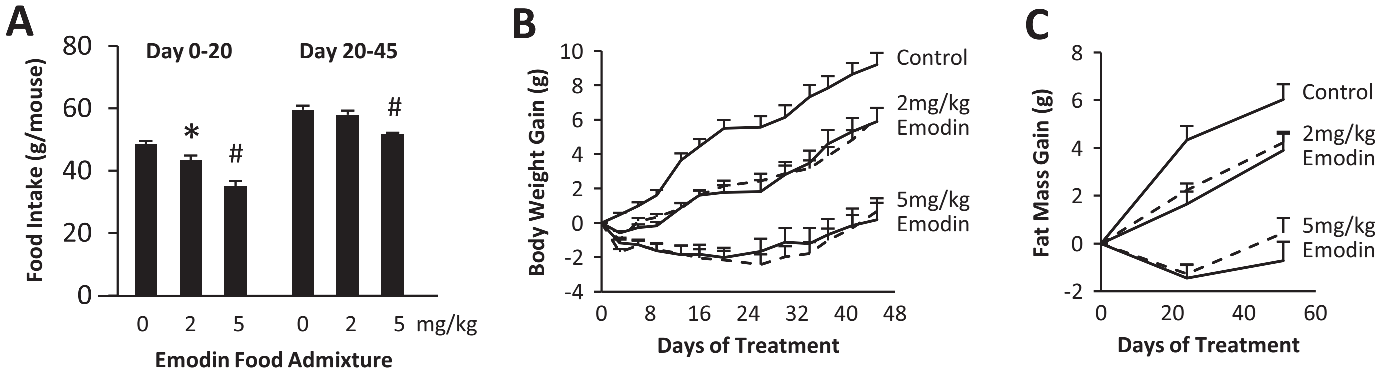

Fig. 2. Effects of emodin on appetite, weight, and fat mass in obese mice. Mice were fed high fat diet with admixtures of emodin (2 or 5 g/kg) and compared to controls with free access to food, as well as to controls fed restrictedly to obtain weight changes as seen under emodin treatment. Means ± S.E.M.; n=11 or 12 per group. (A) Food intake. *$P < 0.05$; #$P < 0.01$ vs. no emodin in food. (B and C) Gain of body weight and fat mass. Full lines: mice fed ad libitum; broken lines: corresponding food restricted mice; all groups significantly different at $P <$ 0.01 by two way ANOVA, except those intentionally weight-matched.

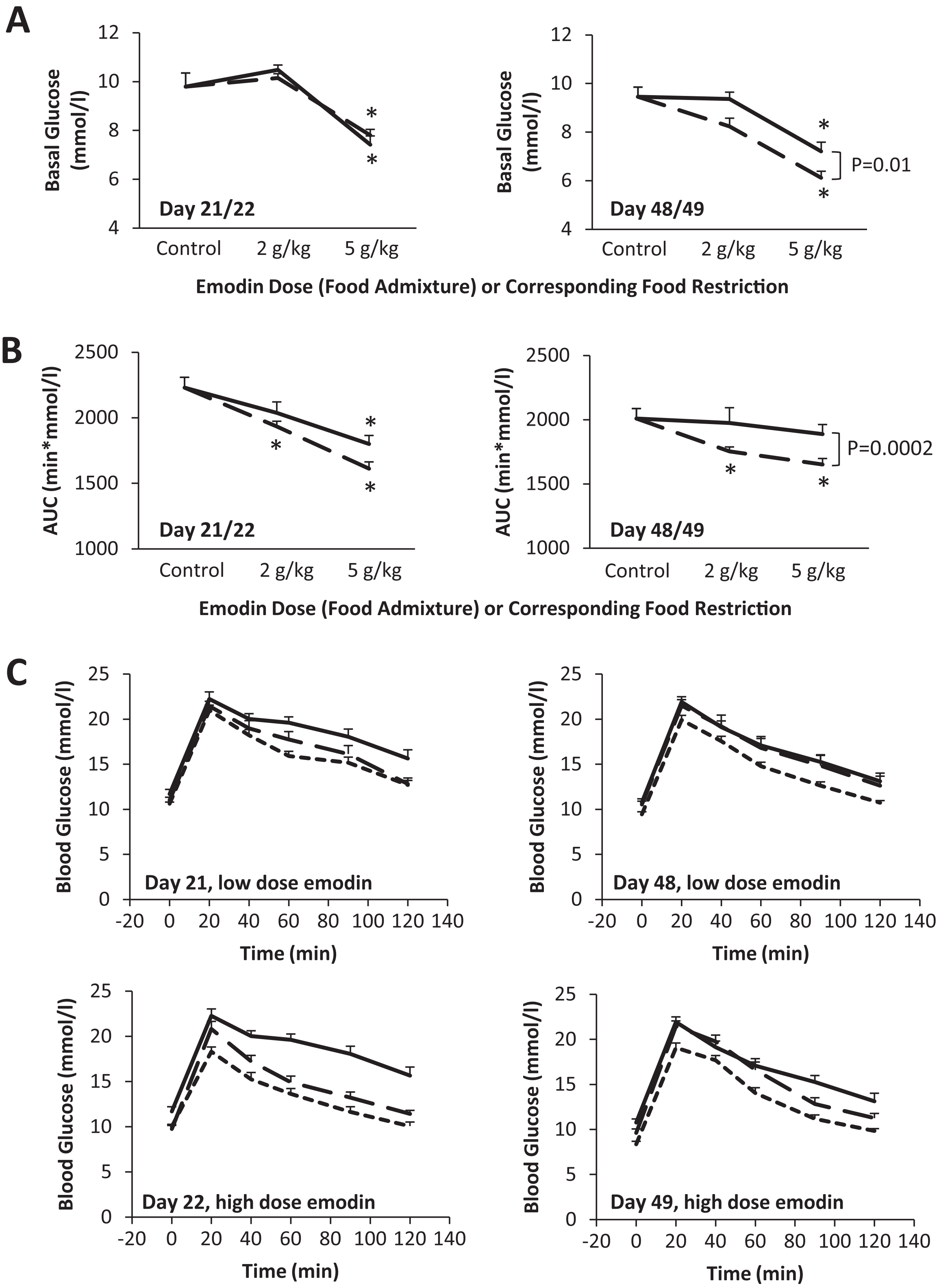

Fig. 3. Prolonged emodin treatment deteriorates blood glucose. Basal blood glucose (A) and areas under the glucose curves from glucose tolerance tests (AUC; B) in mice with free access to high fat diet without (Control) or with admixtures of 2 or 5 g/kg emodin, as well as in mice fed restrictedly to induce similar weight changes as under emodin treatment. Full lines: dose dependent effect of emodin; broken lines: effect of corresponding food restriction; *$P < 0.05$ vs. freely feeding controls by Student´s *t*-tests corrected according to Bonferroni–Holm; *P*-values in the graphs indicate the difference between emodin treatment and food restriction by two way ANOVA. (C) Full glucose excursion curves of glucose tolerance tests: full lines, mice with free access to high fat diet; broadly broken lines, mice treated with emodin (low and high dose: 2 or 5 g/kg food admixture); finely broken lines: mice fed restrictedly to induce similar weight changes as under emodin treatment. For better readability, curves for the low dose (upper graphs) and high dose (lower graphs) of emodin and the respective control groups are shown separately (curves from freely fed controls are depicted twice). Means ± S.E.M.; n=11 or 12 each.

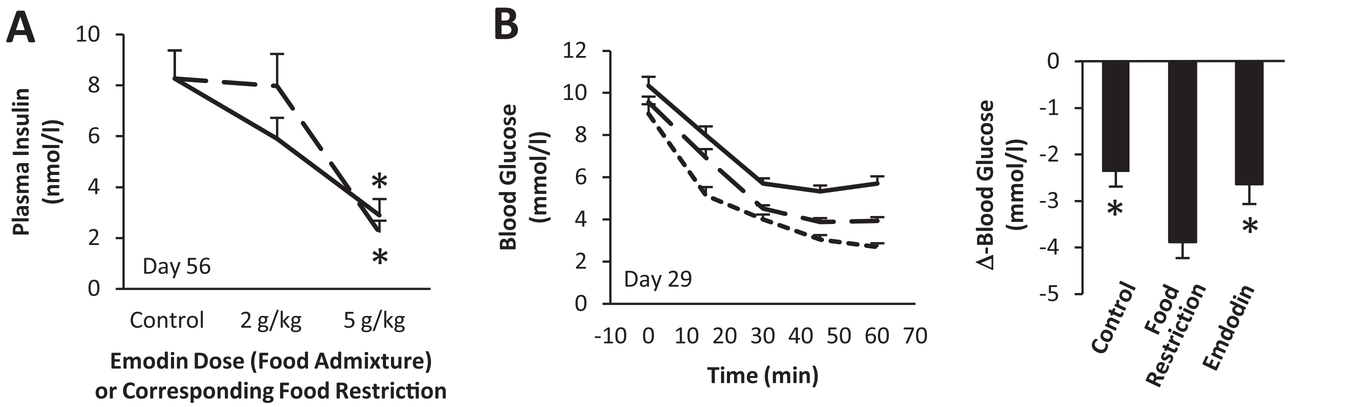

Fig. 4. Effects of emodin on plasma insulin and insulin tolerance in obese mice. (A) Plasma insulin in mice with free access to high fat diet without (Control) or with admixtures of 2 or 5 g/kg emodin as well as in mice fed restrictedly to induce similar weight changes as under emodin treatment. Full line: dose dependent effect of emodin; broken line: effect of corresponding food restriction; $^{*}P < 0.05$ vs. freely feeding controls. (B) Insulin tolerance test in mice with free access to high fat diet without (Control; full line) or with admixture 5 g/kg emodin (broadly broken line), as well as in mice fed restrictedly to induce similar weight changes as under emodin treatment (finely broken line). The right graph shows the drop of blood glucose during the initial 15 min; $^{*}P < 0.05$ vs. the food restricted control group. Means ± S.E.M.; n=11 or 12 per group.

insulin secretion, as we have found it in acutely treated rats, had a role in the observed diabetogenic potential of subchronic emodin treatment. While the purely observational findings of deranged insulin secretion, insulin sensitivity and glucose tolerance could be related to the use of overshooting doses in rodent studies, consumption of comparable amounts of emodin appears unlikely in patients using herbal remedies. Nevertheless, the observed derangements underscore our conclusion that preclinical studies using such doses do not allow any claim of antidiabetic activity.

Without questioning appetite reduction by emodin, our study thus turns emodin from a promising antihyperglycaemic agent into a potentially diabetogenic compound. Furthermore, the observed dependence of glucose lowering activity on weight loss necessarily raises the question, to what extent molecular mechanisms, which have previously claimed to account for emodin induced glucose lowering (Chao et al., 2010; Chen et al., 2012; Feng et al., 2010; Gebhardt et al., 2010; Li et al., 2016; Song et al., 2013; Wang et al., 2012; Yang et al., 2007), are secondary or unrelated to changes in adiposity and the metabolic state.

5. Conclusions

In conclusion, the present study pinpoints the indispensability of weight-matched control groups to exclude indirect metabolic effects via a reduction in food intake and exemplifies, how hurling into fashionable molecular pathway analysis without preceding consideration of simple explanations can misdirect conclusions and impair patient care.

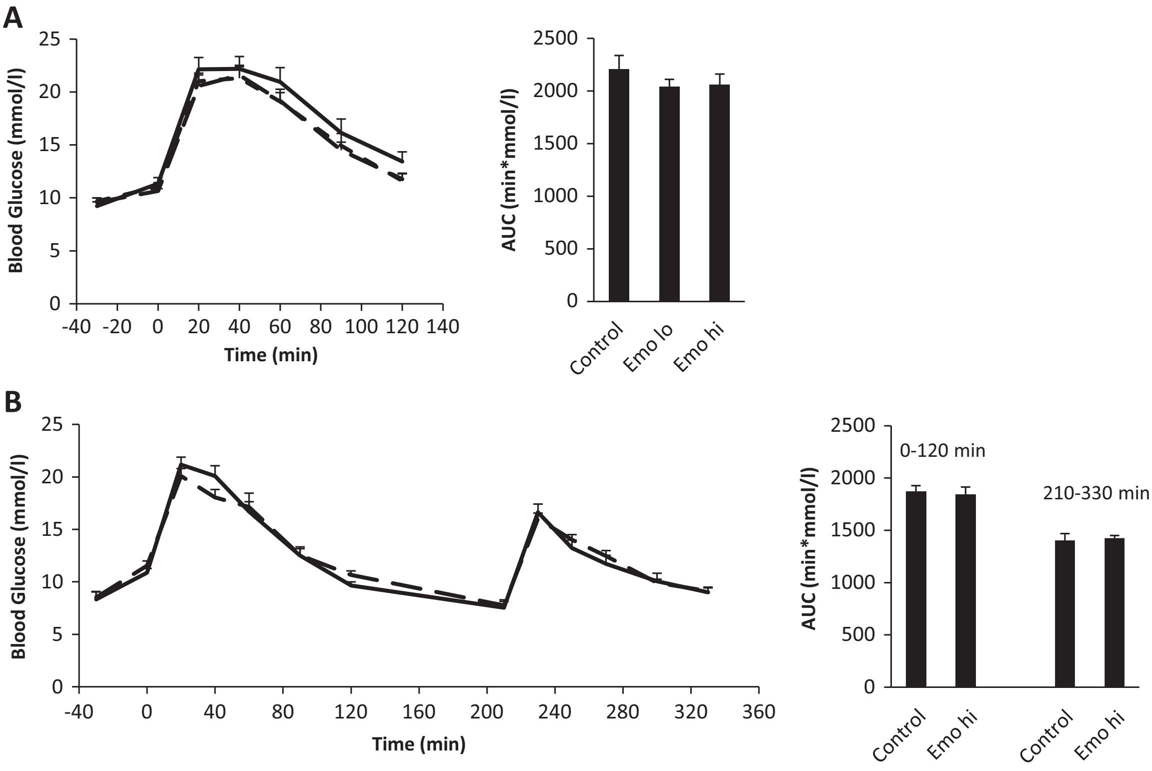

Fig. 5. Effect of a single emodin dose on glucose tolerance in obese mice. Glucose excursion and areas under the curves (AUC) from glucose tolerance tests (GTTs) in high fat-fed mice. (A) Intraperitoneal GTT 30 min after a single oral dose of 100 mg/kg emodin (Emo lo, finely broken line), 250 mg/kg emodin (Emo hi, broadly broken line), or vehicle (Control, full line). (B) Oral GTTs 30 min and 240 min after a single oral dose of 250 mg/kg emodin (Emo hi, broadly broken line) or vehicle (Control, full line). Means ± S.E.M.; n=10–12 per group; no significant effect of emodin.

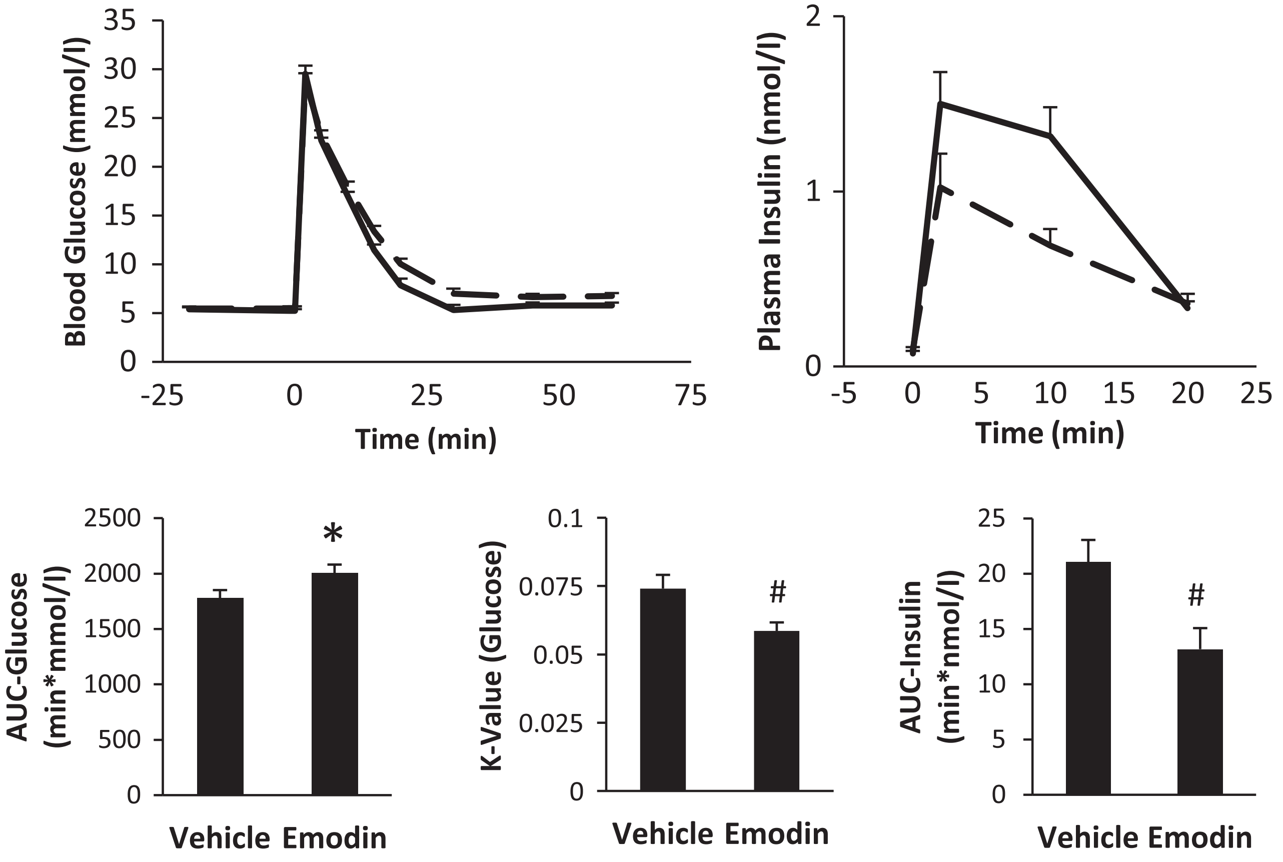

Fig. 6. Effect of an emodin dose on glucose tolerance and insulin secretion in rats. Glucose and insulin excursion, as well as areas under the curves (AUC) and K-value for glucose clearance from glucose tolerance tests in rats treated with a single intravenous dose of 2 mg/kg emodin (broken lines) or with the vehicle (full lines). Means ± S.E.M.; n=9 per group; bar graphs: $^{*}P < 0.05$, $^{\#}P < 0.02$ vs. vehicle.

More specifically, the findings seriously compromise claims that have been raised about an antidiabetic potential of emodin as well as about molecular mechanisms mediating such action. Applying a more stringent setup, we show that glucose lowering in rodents can be completely explained by a spoilt appetite, with no additional effect linked to the postulated glucose lowering mechanisms probable. Although current evidence for emodin's antidiabetic potential is purely experimental and no registered emodin-derived diabetes drug is expected any time soon, the confusingly unregulated dynamics of the modern herbal (online-) market gave rise to a trail of articles heralding emodin as a natural cure for diabetes on non-scientific medicinal internet sites. At the time of writing, this included that an antidiabetic potential was widely cited in online shops for herbal products and was the first suggested “pharmacological” effect on emodin's English Wikipedia entry, an initial stop for many un-supposing customers eager to self-medicate (accessed on 04.07.2016). Since this situation obviously evokes the danger of misuse by diabetic patients, our results illustrate the importance of retaining an appropriate dose of professional scepticism, before putative therapeutic effects are derived from inadequate test designs, in particular for potentially lethal conditions like diabetes.

Acknowledgements

We thank the team of the Core Unit for Biomedical Research, Medical University of Vienna, for providing access to their research facilities and for taking care of the mice.

This research did not receive any specific grant from funding agencies in the public, commercial, or not-for-profit sectors.

Appendix A. Supporting information

Supplementary data associated with this article can be found in the online version at doi:10.1016/j.ejphar.2017.01.022.

References

Ayala, J.E., Samuel, V.T., Morton, G.J., Obici, S., Croniger, C.M., Shulman, G.I., Wasserman, D.H., McGuinness, O.P., Consortium, N.I.H.M.M.P.C., 2010. Standard operating procedures for describing and performing metabolic tests of glucose homeostasis in mice. Dis. Models Mech. 3, 525–534.

Chao, P.M., Kuo, Y.H., Lin, Y.S., Chen, C.H., Chen, S.W., Kuo, Y.H., 2010. The metabolic benefits of Polygonum hypoleucum Ohwi in HepG2 cells and Wistar rats under lipogenic stress. J. Agric. Food Chem. 58, 5174–5180.

Chen, Z., Zhang, L., Yi, J., Yang, Z., Zhang, Z., Li, Z., 2012. Promotion of adiponectin multimerization by emodin: a novel AMPK activator with PPARgamma-agonist activity. J. Cell. Biochem. 113, 3547–3558.

Feng, Y., Huang, S.L., Dou, W., Zhang, S., Chen, J.H., Shen, Y., Shen, J.H., Leng, Y., 2010. Emodin, a natural product, selectively inhibits 11beta-hydroxysteroid dehydrogenase type 1 and ameliorates metabolic disorder in diet-induced obese mice. Br. J. Pharmacol. 161, 113–126.

Gebhardt, R., Lerche, K.S., Gotschel, F., Gunther, R., Kolander, J., Teich, L., Zellmer, S., Hofmann, H.J., Eger, K., Hecht, A., Gaunitz, F., 2010. 4-Aminoethylamino-emodin–a novel potent inhibitor of GSK-3beta–acts as an insulin-sensitizer avoiding downstream effects of activated beta-catenin. J. Cell. Mol. Med. 14, 1276–1293.

Li, J., Ding, L., Song, B., Xiao, X., Qi, M., Yang, Q., Yang, Q., Tang, X., Wang, Z., Yang, L., 2016. Emodin improves lipid and glucose metabolism in high fat diet-induced obese mice through regulating SREBP pathway. Eur. J. Pharmacol. 770, 99–109.

Li, W.L., Zheng, H.C., Bukuru, J., De Kimpe, N., 2004. Natural medicines used in the traditional Chinese medical system for therapy of diabetes mellitus. J. Ethnopharmacol. 92, 1–21.

Liu, W., Tang, L., Ye, L., Cai, Z., Xia, B., Zhang, J., Hu, M., Liu, Z., 2010. Species and gender differences affect the metabolism of emodin via glucuronidation. AAPS J. 12, 424–436.

Lu, K., Xie, S., Han, S., Zhang, J., Chang, X., Chao, J., Huang, Q., Yuan, Q., Lin, H., Xu, L., Shen, C., Tan, M., Qu, S., Wang, C., Song, X., 2014. Preparation of a nano emodin transfersome and study on its anti-obesity mechanism in adipose tissue of diet-induced obese rats. J. Transl. Med. 12, 72.

ARTICLE IN PRESS

Shia, C.S., Hou, Y.C., Tsai, S.Y., Huieh, P.H., Leu, Y.L., Chao, P.D., 2010. Differences in pharmacokinetics and ex vivo antioxidant activity following intravenous and oral administrations of emodin to rats. J. Pharm. Sci. 99, 2185–2195.
Shrimali, D., Shanmugam, M.K., Kumar, A.P., Zhang, J., Tan, B.K., Ahn, K.S., Sethi, G., 2013. Targeted abrogation of diverse signal transduction cascades by emodin for the treatment of inflammatory disorders and cancer. Cancer Lett. 341, 139–149.
Song, P., Kim, J.H., Ghim, J., Yoon, J.H., Lee, A., Kwon, Y., Hyun, H., Moon, H.Y., Choi, H.S., Berggren, P.O., Suh, P.G., Ryu, S.H., 2013. Emodin regulates glucose utilization by activating AMP-activated protein kinase. J. Biol. Chem. 288, 5732–5742.
Srinivas, G., Babykutty, S., Sathiadevan, P.P., Srinivas, P., 2007. Molecular mechanism of emodin action: transition from laxative ingredient to an antitumor agent. Med. Res. Rev. 27, 591–608.
Steffens, A.B., 1969. A method for frequent sampling of blood and continuous infusion of fluids in the rat without disturbing the animal. Physiol. Behav. 4, 833–836.
Wang, Y.J., Huang, S.L., Feng, Y., Ning, M.M., Leng, Y., 2012. Emodin, an 11beta-hydroxysteroid dehydrogenase type 1 inhibitor, regulates adipocyte function in vitro and exerts anti-diabetic effect in ob/ob mice. Acta Pharmacol. Sin. 33, 1195–1203.
Xie, W., Du, L., 2011. Diabetes is an inflammatory disease: evidence from traditional Chinese medicines. Diabetes, Obes. Metab. 13, 289–301.
Xue, J., Ding, W., Liu, Y., 2010. Anti-diabetic effects of emodin involved in the activation of PPARgamma on high-fat diet-fed and low dose of streptozotocin-induced diabetic mice. Fitoterapia 81, 173–177.
Yang, Y., Shang, W., Zhou, L., Jiang, B., Jin, H., Chen, M., 2007. Emodin with PPARgamma ligand-binding activity promotes adipocyte differentiation and increases glucose uptake in 3T3-Ll cells. Biochem. Biophys. Res. Commun. 353, 225–230.
Zhao, X.Y., Qiao, G.F., Li, B.X., Chai, L.M., Li, Z., Lu, Y.J., Yang, B.F., 2009. Hypoglycaemic and hypolipidaemic effects of emodin and its effect on L-type calcium channels in dyslipidaemic-diabetic rats. Clin. Exp. Pharmacol. Physiol. 36, 29–34.
