# Supplementary material for: Emodin, a rising star in the treatment of glycolipid metabolism disorders: a preclinical systematic review and meta-analysis
Source: PeerJ. 2025 May 15;13:e19221. doi: 10.7717/peerj.19221 (PMC12085882; doi:10.7717/peerj.19221)
Supplement: Supplemental Information 20 [file peerj-13-19221-s020.zip › 8篇英文文献/Song Bing2018.pdf]

[Downloaded free from http://www.phcog.com on Monday, April 4, 2022, IP: 210.38.103.189]

Pharmacogn. Mag.
A multifaceted peer reviewed journal in the field of Pharmacognosy and Natural Products
www.phcog.com | www.phcog.net

ORIGINAL ARTICLE

The Effects of Emodin on Insulin Resistance in KKAy Mice with Diabetes Mellitus

Song Bing, Zhang Haoqiang, Ma Chunyu[1], Yu Yali, Wang Jing, Gao Yumeng, Zheng Hongwei, Liu Xuezheng[1]

Department of Endocrinology, The First Affiliated Hospital of Liaoning Medical University, Liaoning, [1]College of Basic Medicine, Jinzhou Medical University, Jinzhou, China

Submitted: 16-08-2017 Revised: 08-09-2017 Published: 14-08-2018

ABSTRACT

Background: Emodin can ameliorate insulin resistance in diabetes mellitus (DM), but the molecular mechanisms are still uncertain. **Objective:** The objective of this study is to identify the potential molecular mechanisms of emodin-mediated type 2 DM treatment. **Methods:** We treated the type 2 diabetic KKAy mice with emodin in different doses. Biochemistry data were collected, and the expression of peroxisome proliferator activated receptor γ (PPARγ) and Glucose transporter (GLUT)-2/4 were examined in liver, muscle, and adipose tissues using immunohistochemistry and reverse transcriptase polymerase chain reaction. The expression of IRS-1, PI3K, pAkt-ser473, and FoxO1 were also tested in these tissues. **Results:** Our data demonstrated that the levels of cholesterol, higher fasting plasma glucose, total cholesterol, total triacylglycerol, low-density lipoprotein cholesterol, free fatty acid, C creative protein, and tumor necrosis factor-α ($P < 0.05$), lower high-density lipoprotein, and insulin sensitivity index ($P < 0.05$) were ameliorated by emodin in a dose-dependent manner ($P < 0.05$). In addition, emodin was also identified to improve insulin sensitivity in KKAy diabetic mice ($P < 0.05$). In DM, the expression of PPARγ and GLUT-2 was down-regulated in liver ($P < 0.05$) as well as in muscle and adipose tissues ($P < 0.05$) when compared with the controls. However, the decreased levels were subject to emodin treatment in a dose-dependent manner. Meantime, emodin was identified to up-regulate the expression of IRS-1, PI3K, Akt-ser473 ($P < 0.05$), while FoxO1 ($P < 0.05$) was down-regulated. **Conclusion:** These results suggest that emodin represents a promising target to improve insulin sensitivity by enhancing liver glucose utilization, glucose uptake of muscle, and fat through IRS/PI3K/Akt/FoxO1 pathway.

Key words: Emodin, glucose transporter, insulin sensitivity, KKAy diabetic mice, peroxisome proliferation-activated receptor-γ, phosphatidylinositol-3 kinase

SUMMARY

- Emodin can ameliorate insulin resistance in diabetes mellitus, but the molecular mechanisms are still uncertain. So, we measured PPARγ, GLUT, and IRS/PI3K/Akt/FoxO1 pathway in KKAy mice with diabetes mellitus in this present study. At last, we demonstrast that emodin improve insulin sensitivity in KKAy mice via PPARγ, GLUT, and IRS/PI3K/Akt/FoxO1 signaling pathway.

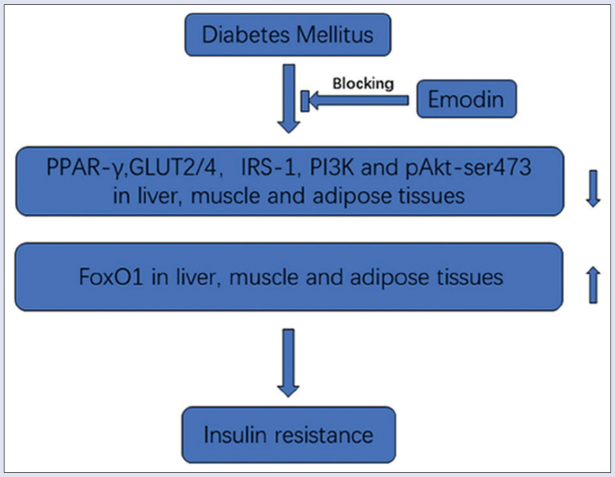

Abbreviations used: DM: Diabetes mellitus; PPARγ: Peroxisome proliferator activated receptor γ; GLUT: Glucose transporter; IRS: Insulin receptor substrate; PI3K: Phosphatidylinositide 3 kinase; Akt(PKB): Protein kinase; FoXO1: Fork head box protein O1.

Access this article online
Website: www.phcog.com
Quick Response Code:

Correspondence:

Prof. Liu Xuezhengu,
3-40, Songpo Road, Jinzhou,
Liaoning Province, China.
E-mail: liuxuezheng168@vip.sina.com
DOI: 10.4103/pm.pm_362_17

INTRODUCTION

Insulin resistance is a precondition of type 2 diabetes mellitus in which cells become resistant to insulin and are unable to use it effectively, leading to hyperglycemia. Pancreatic β cells subsequently increase the production of insulin. Further contribute to hyperinsulinemia.[1] When certain cell types such as fat and muscle cells fail to respond adequately to circulating insulin and cannot take in glucose, blood glucose levels rise. IR is defined by an impaired response of a target tissue such as adipose tissue, liver, and muscle to insulin.[2] The liver helps to regulate glucose levels by reducing gluconeogenesis and glycogenolysis in the presence of insulin. In addition, insulin resistance in lipocytes reduces uptake of circulating lipids, increases mobilization of stored lipids, and elevates free fatty acids (FFAs) in the blood plasma.[3] Elevated insulin and glucose levels in plasma caused by insulin resistance are a major component of the metabolic syndrome. If insulin resistance persists and the pancreas cannot compensation enough insulin, this situation will lead to increase blood glucose concentrations and type 2 diabetes occurs.[1,3]

Emodin is a member of natural compounds known as anthraquinones. Among higher plants, plants belonging to families of Rubiaceae, Rhamnaceae, Fabaceae, Polygonaceae, Bignoniaceae, Verbenaceae, Scrophulariaceae, and Liliaceae are rich sources of anthraquinones.[4] Emodin, an 11 β-hydroxysteroid dehydrogenase

This is an open access journal, and articles are distributed under the terms of the Creative Commons Attribution-NonCommercial-ShareAlike 4.0 License, which allows others to remix, tweak, and build upon the work non-commercially, as long as appropriate credit is given and the new creations are licensed under the identical terms.

For reprints contact: reprints@medknow.com

Cite this article as: Bing S, Haoqiang Z, Chunyu M, Yali Y, Jing W, Yumeng G, *et al*. The effects of emodin on insulin resistance in KKAy mice with diabetes mellitus. Phcog Mag 2018;14:344-50.

 © 2018 Pharmacognosy Magazine | Published by Wolters Kluwer - Medknow

[Downloaded free from http://www.phcog.com on Monday, April 4, 2022, IP: 210.38.103.189]

type 1 inhibitor, suppressed 11-dehydrocorticosterone-induced adipogenesis, and 11-dehydrocorticosterone-stimulated lipolysis to exert antidiabetic effect in ob/ob mice.[5] In diabetes mellitus (DM) model, the levels of blood glucose, triglyceride, total cholesterol (TC), high-density lipoprotein cholesterol in serum were significantly decreased with glucose tolerance and insulin sensitivity improved after emodin treatment.[6] Emodin up-regulates glucose metabolism, decreases lipolysis, and attenuates inflammation in myotubes and 3T3-L1 adipocytes.[7] Emodin enhanced GLUT4 translocation and glucose uptake into the myotube in an Adenosine Monophosphate Activated Protein Kinase (AMPK)-dependent manner, and increased insulin sensitivity was also induced by emodin through insulin-stimulated PI3K phosphorylation in diabetic mice models.[8]

Within the liver, insulin resistance manifests as increased gluconeogenesis and glycogenolysis, thereby increasing endogenous glucose production.[9] Human skeletal myoblasts transplantation has been shown to attenuate hyperglycemia and improve insulin sensitivity in a type 2 diabetic mouse model.[10] In insulin pathway, insulin-mediated IRS1/2 phosphorylation activates PI3K/Akt pathway, which regulates metabolic processes such as GLUT-mediated glucose uptake (muscle and adipocytes), glycogen synthesis (muscle and liver), and gluconeogenesis (liver).[11] Emodin was found to protect against diabetic cardiomyopathy by regulating the Akt/GSK-3 β signaling pathway.[12] In response to metabolites, inflammatory mediators (prostaglandin and leukotrienes), and extracellular stimuli (TGF-β, Wnt, and IGF1), activated PPARγ in adipocytes guarantees a balanced and adequate secretion of adipocytokines (adiponectin and leptin) by binding to DNA consensus sequence through the PPARγ-retinoid X receptor (RXR)-P300-CEBPα complex formation.[13] Promotion of adiponectin multimerization by emodin enhanced both AMPK and PPARγ activities, which make emodin potential drug candidates for the treatment of type 2 diabetes.[14]

In the present study, we aimed to the effects of emodin on the improvement of insulin resistant condition of spontaneous diabetic KKAy mice and clarify its regulation of glucose utilization and storage in liver, skeletal muscles, and fat with the modulation of PI3K/Akt/FoxO1 pathway investigated.

MATERIALS AND METHODS

Animal care, diets, and treatment

KKAY and wild-type C57BL/6J littermates were purchased from the Institute of Laboratory Animal, Chinese Academy of Medical Sciences, Beijing, China. Every three mice were housed to a plastic cage with paper chips for bedding. All had access to standard rodent food (Beijing HFK Bioscience Co., Ltd, China) and water *ad libitum*. They were housed under specific pathogen-free conditions in a temperature-controlled animal room with a 12-h light/dark illumination cycle. Animal use procedures were in accordance with the Guide for the Care and Use of Laboratory Animals and approved by the Committee on Animal Experimentation of Liaoning Medical University.

Thirty-two female KKAy mice were divided randomly into four groups, diabetes model group, emodin low-dose group (EL), emodin-high dose group (EH), and pioglitazone group (PI). Meanwhile, each group was intragastric administration one time with the vehicle (H_2O, 20 ml/kg, DM), emodin (12.5 mg/kg, EL, and 50 mg/kg, EH), or PI (1.95 mg/kg, PI) for 8 weeks. Meanwhile, each mouse was given with a high-fat diet. The C57BL/6J were administration with H_2O (20 ml/kg) as negative control (NC).

Sample collection

After animals were killed under sodium pentobarbital anesthesia, then liver, skeletal muscle, and fat of the animals were removed immediately. One portion of the tissues was immediately frozen in liquid nitrogen and then stored at −80°C until RNA and protein extraction. The other portion of the tissues were fixed in 4% neutralized formaldehyde, embedded in paraffin and incised into 4 μm sections. These sections were stained by hematoxylin and eosin (HE) method to confirm their histological appearance. The blood was obtained from the intraocular vein and subjected to serum separation by centrifugation.

Blood biochemical indicator detection

Plasma glucose, triglycerides, and FFA concentrations were measured by colorimetric assays (Wako, Japan). Plasma concentrations of insulin, tumor necrosis factor-α (TNF-α) and C-reactive protein were assayed using Bioss kits (China). All procedures were performed according to each manufacturer's instructions.

Immunohistochemistry

Four-μm-thick sections were deparaffinized with xylene and rehydrated through an alcohol gradient. The sections were quenched with 3% hydrogen peroxide in absolute methanol for 20 min to block endogenous peroxidase activity and heated in a microwave for 15 min in citrate buffer (0.01 mol/L, pH 6.0) to retrieve the antigen. The sections were incubated with the primary antibody against PPARγ, GLUT-2, or GLUT-4 (Bioss, China; 1:100) for 60 min, followed by exposure to Envision-PO (DAKO, USA) antibody for 60 min. Coloring was performed with 3, 3'-diaminobenzidine with the 5 min reaction. After each treatment, the slides were washed with TBST three times for 1 min. After counterstained with Mayer's hematoxylin, the sections were dehydrated, cleared, and mounted. The omission of the primary antibody was employed as a negative control.

Reverse transcriptase polymerase chain reaction

Total RNA was extracted from muscle, muscle and fat tissues using QIAGEN RNeasy mini kit (QIAGEN, Hilden, Germany) and subjected to cDNA synthesis using the Avian Myeloblastosis Virus transcriptase and random primer (Takara, Otsu, Japan). Oligonucleotide primers were sense: 5'-TTTCAAGGGTGCCAGTTT-3' and anti-sense: 5'-GAGGCCA GCA TCGTGTAG-3'forPPARγ(152bp);Sense:5'-TACGGCAATGGCTTTATC-3' and antisense: 5'-CCTCCTGCAACTTCTCAAT-3' for GLUT-2 (208 bp); sense: 5'-ACTGGCACTTCCAC TGAACTCTTG-3' and anti-sense: 5'-TTTCTGCTCCCTATCCGTTCTT-3' for GLUt4 (388 bp), and sense: 5'-ATCATGTTTGAGACCTTCAACA-3' and antisense: 5'-CATCTC TTGCTC GAAGTCC A-3' for the internal control-β-actin (308 bp). Polymerase chain reaction (PCR) amplification of cDNA was performed in 25 μl mixture using Hot-start Taq polymerase (Takara, Japan). Finally, the amplicons were electrophoresed in 1.5% agarose gel for 30 min. Densitometric quantification was performed with an internal β-actin control using Scion Image software.

Western blot

Denatured protein was separated on a sodium lauryl sulfate-polyacrylamide gel (10% acrylamide) and transferred to Hybond membrane (Amersham, Amersham, Germany), which was then blocked overnight in 5% skim milk in Tris-buffered saline with Tween 20 (TTBS). For immunoblotting, the membrane was incubated for 1 h with the antibody against IRS-1, PI3K, Akt, Ser473, FoXO1 (Bioss, China; 1:1000), or β-actin (Santa Cruz, California, USA; 1:5000). Then, it was rinsed by TTBS and incubated with IgG conjugated to horseradish peroxidase (DAKO, Carpinteria, USA; 1:1000) for 1 h. Bands were visualized with LAS4000 (Fujifilm, Tokyo, Japan) by ECL-Plus detection reagents (Santa Cruz, California, USA). Densitometric quantification was performed with an internal β-actin control using Scion Image software.

[Downloaded free from http://www.phcog.com on Monday, April 4, 2022, IP: 210.38.103.189]

Statistical analysis

All the experiments were repeated three times, and results are described as means ± standard deviation. Statistical evaluation was performed using Mann–Whitney U-test to analyze the means of different groups. $P < 0.05$ was considered as statistically significant. SPSS 18.0 software was employed to analyze all data.

RESULTS

Emodin ameliorates the insulin resistance in KKAy mice with spontaneous diabetes mellitus

As shown in Table 1, higher blood glucose was detected in the DM group than the NC group ($P < 0.01$), and then significantly reduced by the treatments with emodin and PI in the DM group. Higher blood

Table 1: Effect of emodin treatment on the biochemical factor in KKAy mice with diabetes mellitus

	NC group	DM group	EL group	EH group	PI group
Blood glucose	5.24±0.29	8.80±0.26*	6.80±0.36$^{\#}$	5.56±0.38$^{\#,\&}$	5.67±0.30$^{\#}$
Blood insulin	19.38±1.19	54.22±6.24*	29.17±2.05$^{\#}$	22.10±2.45$^{\#,\&}$	20.68±2.49$^{\#}$
ISI	−1.11±0.02	−1.81±0.06*	−1.66±0.08$^{\#}$	−1.12±0.05$^{\#,\&}$	−1.10±0.06$^{\#}$
TC	2.93±0.7	5.77±0.21*	4.02±0.10$^{\#}$	2.93±0.07$^{\#,\&}$	2.81±0.11$^{\#}$
TG	0.46±0.03	2.74±0.63*	1.33±0.11$^{\#}$	0.96±0.06$^{\#,\&}$	0.89±0.09$^{\#}$
HDL-C	3.07±0.14	1.79±0.15*	2.02±0.11$^{\#}$	2.89±0.15$^{\#,\&}$	2.98±0.10$^{\#}$
LDL-C	1.32±0.09	2.96±0.16*	2.61±0.17$^{\#}$	2.02±0.17$^{\#,\&}$	1.86±0.12$^{\#}$
FFA	153.64±8.8	414.90±27.94*	298.03±16.56$^{\#}$	246.13±10.47$^{\#,\&}$	166.37±12.50$^{\#}$
hs-CRP	1.11±0.04	2.44±0.10*	2.15±0.10$^{\#}$	1.22±0.08$^{\#,\&}$	1.21±0.07$^{\#}$
TNF-α	1.11±0.03	2.44±0.06*	2.01±0.05$^{\#}$	1.38±0.04$^{\#,\&}$	1.35±0.04$^{\#}$

PS: postscript. *P<0.05, versus NC group; $^{\#}P$<0.05, versus DM group; $^{\&}P$<0.05, versus EL group. ISI: insulin sensitivity index; TC: Total cholesterol; TG: Total triacylglycerol; LDL-C: Low-density lipoprotein cholesterol; hs-CRP: High-sensitivity C reactive protein TNF-α: Tumor necrosis factor-α; NC: Negative control; DM: Diabetes model; EL: Emodin-low dose; EH: Emodin-high dose; PI: Pioglitazone; FFA: Free fatty acid

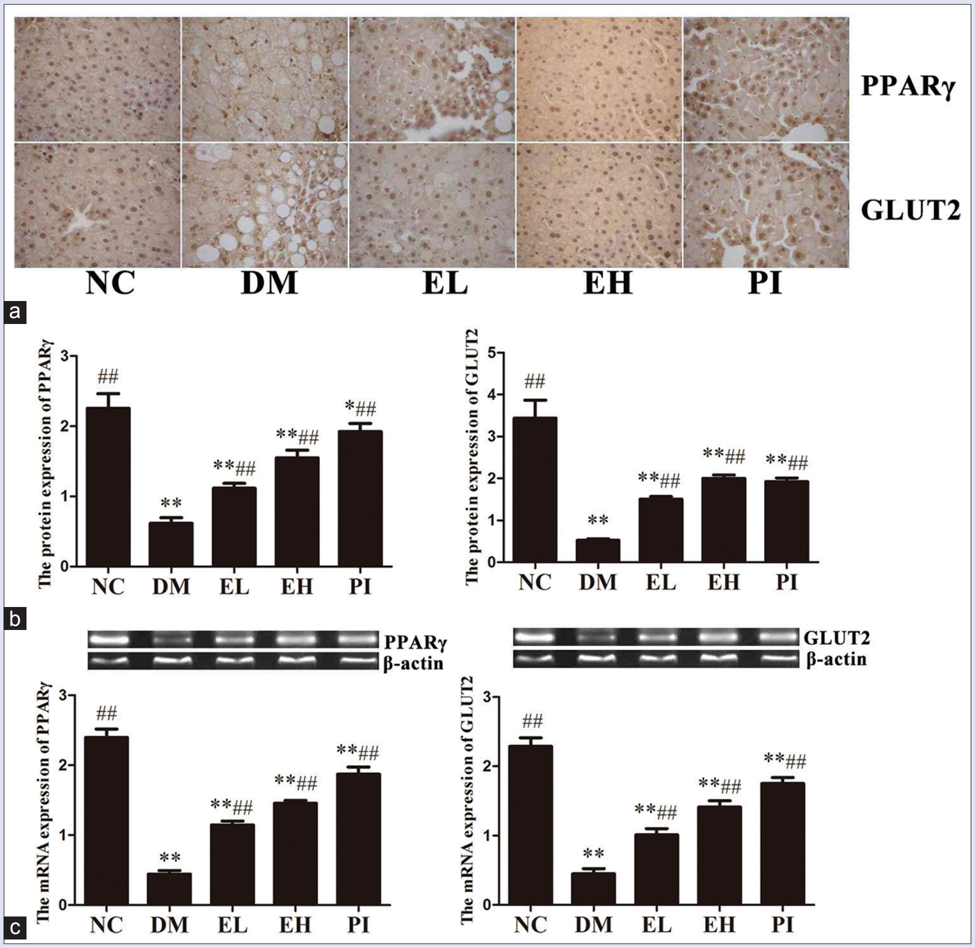

Figure 1: The effects of emodin treatment on the expression of PPARγ and GLUT-2 in liver of diabetes mellitus. (a) Results of immunohistochemistry for liver tissue; (b) results of densitometric analysis for liver tissue; (c)results of reverse transcriptase polymerase chain reaction for liver tissue. $^{*}P < 0.05$, $^{**}P < 0.01$, versus NC group; $^{\#}P < 0.05$, $^{\#\#}P < 0.01$, versus DM group. NC, negative control; DM, diabetes mellitus; EL, emodin low-dose group; EH, emodin high-dose group; PI, pioglitazone group

[Downloaded free from http://www.phcog.com on Monday, April 4, 2022, IP: 210.38.103.189]

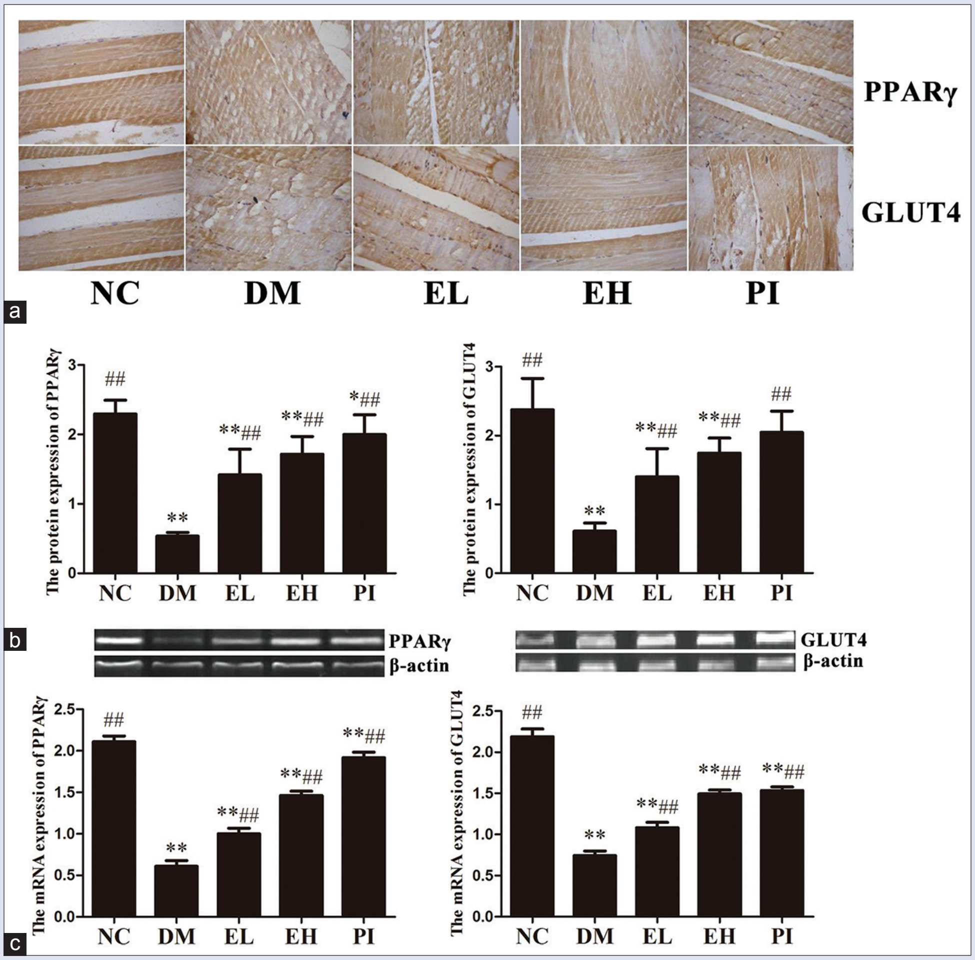

Figure 2: The effects of emodin treatment on the expression of PPARγ and GLUT-4 in skeletal muscle of diabetes mellitus. (a) Results of ICH for skeletal muscle; (b) results of densitometric analysis for skeletal muscle; (c)results of reverse transcriptase-polymerase chain reaction for skeletal muscle. $^{*}P < 0.05$, $^{**}P < 0.01$, versus NC group; $^{\#}P < 0.05$, $^{\#\#}P < 0.01$, versus DM group. NC, negative control; DM, diabetes mellitus; EL, emodin low-dose group; EH, emodin high-dose group; PI, pioglitazone group

insulin and lower insulin sensitivity index (ISI) were monitored in the DM group than the NC group ($P < 0.01$). These two indicators were then significantly restored by the treatments with emodin and PI. The levels of TC, total triacylglycerol, high-density lipoprotein cholesterol (HDL-C), low-density lipoprotein cholesterol (LDL-C), FFA, high-sensitivity C reactive protein (hs-CRP), and TNF-α were dramatically changed to be comparable to NC group ($P < 0.01$) after the drug intervention.

The effects of emodin treatment on the expression of PPARγ and GLUT in liver, skeletal muscle, and fat of diabetes mellitus

Lower expression of PPARγ and GLUT-2 in hepatic tissue of DM group was detected using IHC compared with the NC group [Figure 1a]. Expression of PPARγ and GLUT-2 in hepatic tissue of EL, EH, and PI group was up-regulated in comparison to the DM group [$P < 0.05$, Figure 1]. By reverse transcriptase PCR (RT-PCR) and densitometric analysis, mRNA expression of PPARγ and GLUT-2 was consistent in hepatic tissue [$P < 0.05$, Figure 1]. As illustrated in Figures 2 and 3, there was a lower protein expression of PPARγ and GLUT-4 in muscle tissue and adipose tissue of the DM group than the NC group ($P < 0.05$). Compared with the DM group, the protein expression of PPARγ and GLUT-4 in muscle and adipose tissues of EL, EH, and PI group was also up-regulated ($P < 0.05$). The same data were observed for the mRNA expression of PPARγ and GLUT-4 in muscle and adipose tissues [$P < 0.05$, Figures 2 and 3].

Emodin targets PI3K/Akt/FoxO1 signal pathway in KKAy mice with diabetes mellitus

According to the Western blotting and densitometric analysis, IRS-1, PI3K, and pAkt-ser473 were down-regulated, while FoxO1 were over-expressed in liver, muscle, and adipose tissues of the DM group as compared with the NC group ($P < 0.05$). Compared with DM group, the protein expression of IRS-1, PI3K, and Akt-ser473 in hepatic tissue, muscle tissue, and adipose tissue of EL and EH group was up-regulated ($P < 0.05$), while the protein expression of FoxO1 was decreased ($P < 0.05$) [Figure 4].

DISCUSSION

Insulin resistance was observed in KKay mice treated with high-fat diet for 8 weeks, and phenotypes of hyperglycemia, hyperinsulinemia, lower ISI were exhibited at 8-week age because the pancreatic islets

[Downloaded free from http://www.phcog.com on Monday, April 4, 2022, IP: 210.38.103.189]

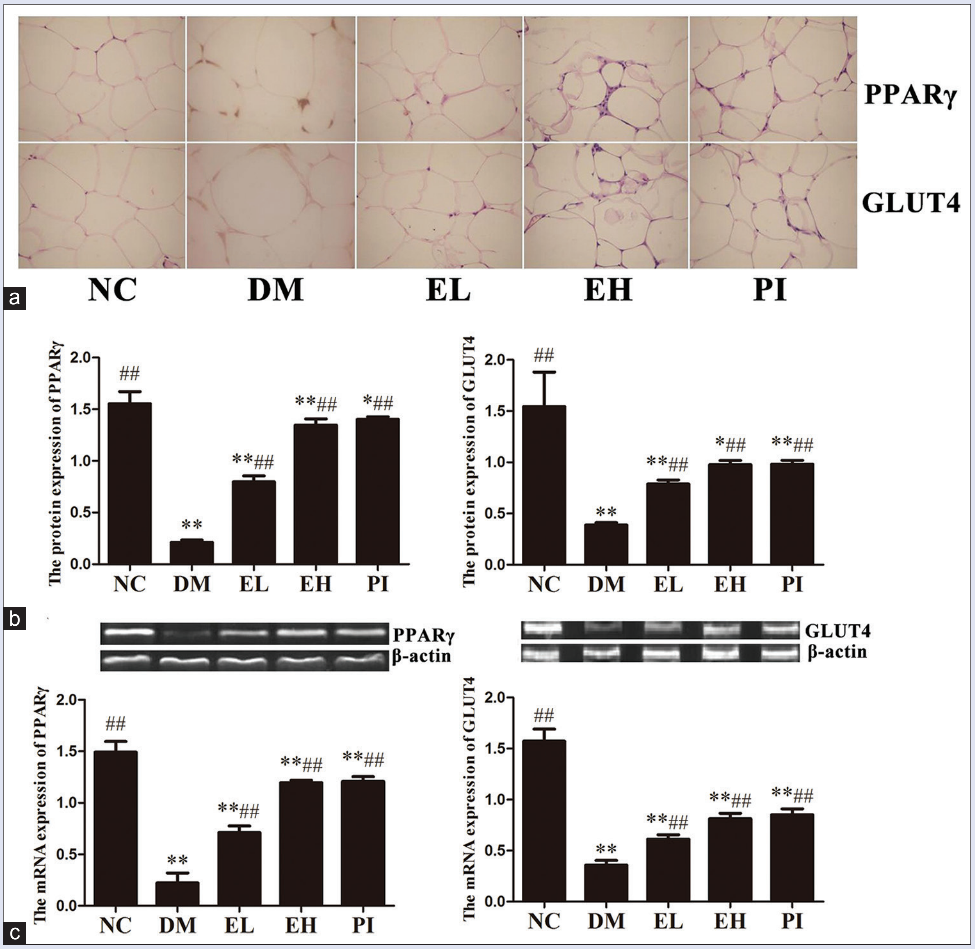

Figure 3: The effects of emodin treatment on the expression of PPARγ and GLUT-4 in fat of diabetes mellitus. (a) Results of ICH for fat; (b) results of densitometric analysis for fat; (c) results of reverse transcriptase-polymerase chain reaction for fat. $^{*}P < 0.05$, $^{**}P < 0.01$, versus negative control group; $^{\#}P < 0.05$, $^{\#\#}P < 0.01$, versus DM group. NC, negative control; DM, diabetes mellitus; EL, emodin low-dose group; EH, emodin high-dose group; PI, pioglitazone group

are hypertrophied and the β-cells are degranulated.[15] As a cause of insulin resistance, adipocytes hypertrophy and hyperplasia results in inflammatory responses, such as TNF-α production.[16] TNF-α activates inhibitory molecules such as SOCS and JNK and enhances the degradation of IRS1/2 by E3 ubiquitin ligase activation pathway to suppress the generation of insulin signal.[11] In our KKay model, serum TNF-α level was increased as a cause and result of insulin resistance. Emodin was found to ameliorate the insulin resistance and decrease TNF-α level in a dose-dependent manner, suggesting that emodin has a potential effect to prevent and treat insulin resistance and T2DM by suppressing the inflammatory response in lipocytes.

The main target organs for insulin resistance are liver, muscle, and adipose tissue, which show impaired insulin-stimulated glucose uptake and reduced inhibition of liver glucose production.[11] Therefore, we observed the effects of emodin on the expression of glucose transporters because glucose cannot enter the cell freely and needs the help of active transporters.[17] Among them, GLUT4 is the insulin-regulated glucose transporter found primarily in adipose tissue, skeletal and cardiac muscle,[17] whereas GLUT2 is responsible for the transfer of glucose between liver and blood.[18] Here, GLUTs were decreased in DM model, and emodin treatment up-regulated the expression of GLUTs in liver, skeletal muscle, and fat tissues, suggesting that impaired expression of GLUTs might be reversed by emodin to increase the utilization and restoration of glucose and thereby ameliorate insulin resistance.

Reportedly, proteome proliferator-activated receptors (PPARs) are a group of nuclear receptor proteins that function as transcription factors regulating the expression of genes in its heterodimerization with the RXR. PPARγ is activated by prostaglandin and certain members of the 5-HETE family of arachidonic acid metabolites to regulate the expression of adipokine, activate PI3K, and translocate GLUT-4 to the membrane.[19,20] In our present studies, PPARγ expression was downregulated in liver, muscle, and fat tissues of DM models, and restored by emodin treatment, indicating that emodin can not only weaken the stimuli from prostaglandin and metabolites but also enhance insulin sensitivity and glucose transportation through PPARγ. Therefore, PPAR agonists, combined with emodin, might play an important role in treating diabetes mellitus.

Interactions between insulin and IRS1/2 initiate the activation of PI3K, which subsequently phosphorylates Akt. Akt then phosphorylates FOXO1 for nuclear exclusion where FOXO1 prolongs insulin's inhibitory effect on glycogenolysis and hepatic gluconeogenesis by down-regulating the transcription of glucose 6-phosphatase and activating the transcription of phosphoenolpyruvate carboxykinase.[21-23] In our studies, emodin up-regulates IRS-1, PI3K, Akt-ser473 in liver, muscle, and fat where they were endogenously downregulated in DM, while FoxO1 was down-regulated. These data suggested that the preventive and protective effects of emodin on insulin resistance might be due to activation of IRR/PI3K/Akt/FoxO1 signal pathway of insulin. In the future, we will consider the combination of emodin and

[Downloaded free from http://www.phcog.com on Monday, April 4, 2022, IP: 210.38.103.189]

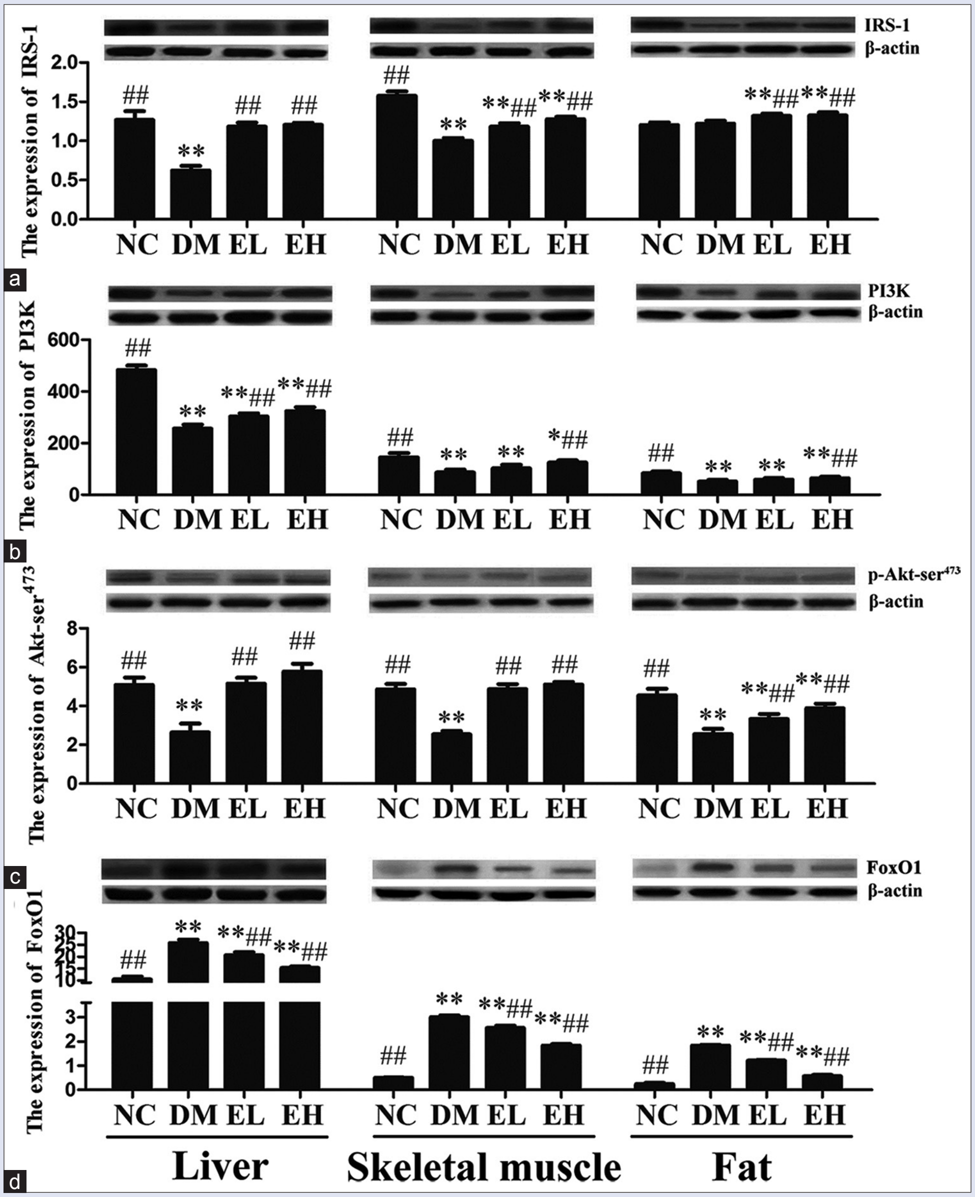

Figure 4: Emodin targets PI3K/Akt/FoxO1 signal pathway in KKAy mice with diabetes mellitus. (a) IRS1 expression in liver skeletal muscle and fat tissue; (b) PI3K expression in liver skeletal muscle and fat tissue; (c) Akt expression in liver skeletal muscle and fat tissue; (d)FoXO1 expression in liver skeletal muscle and fat tissue. $^{*}P < 0.05$, $^{**}P < 0.01$, versus negative control group; $^{\#}P < 0.05$, $^{\#\#}P < 0.01$, versus diabetes mellitus group. NC, negative control; DM, diabetes mellitus; EL, emodin low-dose group; EH, emodin high-dose group; PI, pioglitazone group

IRR/PI3K/Akt/FoxO1 agonists for the prevention and intervention of insulin resistance and T2DM.

CONCLUSION

Emodin can improve insulin sensitivity and resistance by enhancing liver glucose utilization, glucose uptake of muscle, and fat through IRS/PI3K/Akt/FoxO1 pathway. The combination of emodin and PPARγ or IRS/Akt/FoxO1 agonists might be helpful to prevent and treat insulin resistance and T2DM.

Acknowledgements

This study was supported by the scientific foundation of Liaoning Science and Technology administration bureau for doctoral research (20131067).

Financial support and sponsorship

Nil.

Conflicts of interest

There are no conflicts of interest.

REFERENCES

1. Caselli C. Role of adiponectin system in insulin resistance. Mol Genet Metab 2014;113:155-60.
2. Elisha B, Disse E, Chabot K, Taleb N, Prud'homme D, Bernard S, *et al.* Relative contribution of muscle and liver insulin resistance to dysglycemia in postmenopausal overweight and obese women: A MONET group study. Ann Endocrinol (Paris) 2017;78:1-8.
3. Castro AV, Kolka CM, Kim SP, Bergman RN. Obesity, insulin resistance and comorbidities? Mechanisms of association. Arq Bras Endocrinol Metabol 2014;58:600-9.
4. Monisha BA, Kumar N, Tiku AB. Emodin and its role in chronic diseases. Adv Exp Med Biol

[Downloaded free from http://www.phcog.com on Monday, April 4, 2022, IP: 210.38.103.189]

2016;928:47-73.

5. Wang YJ, Huang SL, Feng Y, Ning MM, Leng Y. Emodin, an 11β-hydroxysteroid dehydrogenase type 1 inhibitor, regulates adipocyte function *in vitro* and exerts anti-diabetic effect in ob/ob mice. Acta Pharmacol Sin 2012;33:1195-203.
6. Xue J, Ding W, Liu Y. Anti-diabetic effects of emodin involved in the activation of PPARgamma on high-fat diet-fed and low dose of streptozotocin-induced diabetic mice. Fitoterapia 2010;81:173-7.
7. Zhang X, Zhang R, Lv P, Yang J, Deng Y, Xu J, *et al*. Emodin up-regulates glucose metabolism, decreases lipolysis, and attenuates inflammation *in vitro*. J Diabetes 2015;7:360-8.
8. Song P, Kim JH, Ghim J, Yoon JH, Lee A, Kwon Y, *et al*. Emodin regulates glucose utilization by activating AMP-activated protein kinase. J Biol Chem 2013;288:5732-42.
9. Dunford EC, Riddell MC. The metabolic implications of glucocorticoids in a high-fat diet setting and the counter-effects of exercise. Metabolites 2016;6. pii: E44.
10. Chan DC, Chiu CY, Lan KC, Weng TI, Yang RS, Liu SH, *et al*. Transplantation of human skeletal muscle-derived progenitor cells ameliorates knee osteoarthritis in streptozotocin-induced diabetic mice. J Orthop Res 2017;35:1886-93.
11. Kwon H, Pessin JE. Adipokines mediate inflammation and insulin resistance. Front Endocrinol (Lausanne) 2013;4:71.
12. Wu Z, Chen Q, Ke D, Li G, Deng W. Emodin protects against diabetic cardiomyopathy by regulating the AKT/GSK-3β signaling pathway in the rat model. Molecules 2014;19:14782-93.
13. Janani C, Ranjitha Kumari BD. PPAR gamma gene – A review. Diabetes Metab Syndr 2015;9:46-50.
14. Chen Z, Zhang L, Yi J, Yang Z, Zhang Z, Li Z, *et al*. Promotion of adiponectin multimerization by emodin: A novel AMPK activator with PPARγ-agonist activity. J Cell Biochem 2012;113:3547-58.
15. Vollenweider P, von Eckardstein A, Widmann C. HDLs, diabetes, and metabolic syndrome. Handb Exp Pharmacol 2015;224:405-21.
16. Saleh S, El-Maraghy N, Reda E, Barakat W. Modulation of diabetes and dyslipidemia in diabetic insulin-resistant rats by mangiferin: Role of adiponectin and TNF-α. An Acad Bras Cienc 2014;86:1935-48.
17. Govers R. Molecular mechanisms of GLUT4 regulation in adipocytes. Diabetes Metab 2014;40:400-10.
18. Röder PV, Geillinger KE, Zietek TS, Thorens B, Koepsell H, Daniel H, *et al*. The role of SGLT1 and GLUT2 in intestinal glucose transport and sensing. PLoS One 2014;9:e89977.
19. Astapova O, Leff T. Adiponectin and PPARγ: Cooperative and interdependent actions of two key regulators of metabolism. Vitam Horm 2012;90:143-62.
20. Wei J, Bhattacharyya S, Jain M, Varga J. Regulation of matrix remodeling by peroxisome proliferator-activated receptor-γ: A novel link between metabolism and fibrogenesis. Open Rheumatol J 2012;6:103-15.
21. Cho JY, Park J. Contribution of natural inhibitors to the understanding of the PI3K/PDK1/PKB pathway in the insulin-mediated intracellular signaling cascade. Int J Mol Sci 2008;9:2217-30.
22. Kloet DE, Burgering BM. The PKB/FOXO switch in aging and cancer. Biochim Biophys Acta 2011;1813:1926-37.
23. Schultze SM, Hemmings BA, Niessen M, Tschopp O. PI3K/AKT, MAPK and AMPK signalling: Protein kinases in glucose homeostasis. Expert Rev Mol Med 2012;14:e1.
