# Supplementary material for: Emodin, a rising star in the treatment of glycolipid metabolism disorders: a preclinical systematic review and meta-analysis
Source: PeerJ. 2025 May 15;13:e19221. doi: 10.7717/peerj.19221 (PMC12085882; doi:10.7717/peerj.19221)
Supplement: Supplemental Information 20 [file peerj-13-19221-s020.zip › 8篇英文文献/Xiao-Yan Zhao2009.pdf]

Clinical and Experimental Pharmacology and Physiology (2009) **36**, 29–34 doi: 10.1111/j.1440-1681.2008.05051.x

HYPOGLYCAEMIC AND HYPOLIPIDAEMIC EFFECTS OF EMODIN AND ITS EFFECT ON L-TYPE CALCIUM CHANNELS IN DYSLIPIDAEMIC–DIABETIC RATS

Xiao-Yan Zhao,*† Guo-Fen Qiao,*† Bao-Xin Li,* Li-Min Chai,* Zhe Li,* Yan-Jie Lu*† and Bao-Feng Yang*†

**Department of Pharmacology and †State-Province Key Laboratories of Biomedicine Pharmaceutics, Harbin Medical University, Harbin, China*

SUMMARY

1. The aim of the present study was to evaluate the hypoglycaemic and hypolipidaemic effects of 20, 40 and 80 mg/kg per day emodin and its potential effects on L-type calcium channels in dyslipidaemic–diabetic rats.

2. Dyslipidaemic-diabetic rats were induced by a single intraperitoneal injection of streptozotocin (55 mg/kg) after intragastric administration of a high-fat diet for 2 weeks.

3. Daily administration of emodin for 2 weeks resulted in a significant dose-dependent reductions in blood glucose, serum total cholesterol, triglycerides, free fatty acids and malonaldehyde ($P < 0.05$) in dyslipidaemic–diabetic rats compared with vehicle-treated dyslipidaemic–diabetic rats. In addition, emodin caused dose-dependent increases in plasma superoxide dismutase activity in dyslipidaemic–diabetic rats ($P < 0.05$). Immunofluorescent staining and reverse transcription–polymerase chain reaction showed that the expression of L-type calcium channels in the pancreas and heart was restored, to different extents, by the three doses of emodin treatment.

4. The results of the present study suggest that emodin has antidiabetic and lipid-modulating effects that involve, in part, upregulation of L-type calcium channel expression in the pancreas and heart in dyslipidaemic–diabetic rats.

Key words: diabetes mellitus, emodin, heart, islets of langerhans, L-type calcium channels.

INTRODUCTION

Diabetes mellitus is a major health concern for humans, affecting more than 5% of the population;[1] and this number may double by 2025.[2] Pharmaceutic drugs, such as sulphonylureas and biguanides, are used for the treatment of diabetes. However, these drugs are associated with drawbacks, such as high cost, multiple and rigid dosing regimen and adverse side-effects.[3,4] These limitations have contributed to the recent prevalence of the use of herbal products in the treatment of diabetes.

In recent years, there has been an upsurge in the use of traditional medicines because herbal drugs are generally less toxic and have fewer side-effects if taken appropriately.[5] Emodin (3-methyl-1,6,8-trihydroxyanthraquinone), a natural anthraquinone derivative from the roots of *Rheum officinale* Bail (Fig. 1), has anti-inflammatory, antitumour, immunosuppressive and vasorelaxant properties.[6] Recent studies have demonstrated that emodin also has antipancreatitic effects.[7] Even though emodin has been studied extensively, none of the studies has investigated its antidiabetic activity.

Diabetes mellitus is a disorder characterized by breakdown of glucose homeostasis,[8] with selective impairment of glucose-induced insulin secretion from pancreatic β-cells.[9] It has been reported that the lipotoxicity in the development of diabetes mellitus and hyperlipidaemia further increase the risk of ischaemic heart disease in type 2 diabetes.[10,11] The murine model induced by high-cholesterol chow feeding plus streptozotocin (STZ) injection replicates the natural history and metabolic characteristics of human type 2 diabetes and is suitable for pharmacological studies.[12,13] The aim of the present study was to assess the therapeutic effects of emodin on blood glucose and lipid parameters in dyslipidaemic–diabetic rats.

Insulin secretion is triggered by Ca^{2+} influx through voltage-dependent L-type calcium channels.[14] The L-type high voltage-activated (HVA) Ca^{2+} channel family is comprised of $Ca_v1.1$, 1.2, 1.3 and 1.4 channels.[15] The $Ca_v1.2$ or α_{1C} channels play a central role in insulin secretion.[16] Diabetes mellitus increases the risk of cardiac dysfunction. Many different abnormalities of cardiac contraction and relaxation have been identified in the STZ-induced diabetic heart.[17] In this regard, changes in intracellular Ca^{2+} may be responsible for these abnormalities. L-type calcium channels play an important role in regulating the intracellular Ca^{2+} concentration in cardiomyocytes. It has been reported that emodin can regulate L-type calcium current in normal guinea-pig cardiomyocytes.[18] In the present study, we investigated the effects of emodin on L-type calcium channels in islet β-cells and heart and explored the possible mechanisms responsible for the effects of emodin in dyslipidaemic–diabetic rats.

METHODS

Chemicals

The total cholesterol (TC) reagent kit was purchased from Shanghai Rongsheng Biotech (Shanghai, China). Primary and secondary antibodies were supplied by Santa Cruz (Santa Cruz, CA, USA). Other chemicals and

Correspondence: Dr Bao-Feng Yang, Department of Pharmacology, Harbin Medical University, Baojian Road 157, Harbin, Heilongjiang 150081, PR China. Email: yangbf@ems.hrbmu.edu.cn

Received 28 March 2008; revision 15 June 2008; accepted 17 June 2008.

© 2008 The Authors

Journal compilation © 2008 Blackwell Publishing Asia Pty Ltd

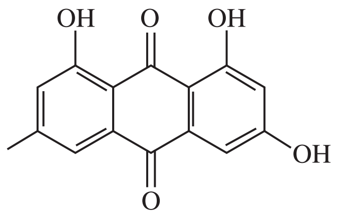

Fig. 1 The chemical structure of emodin.

diagnostic kits were purchased from Sigma Chemical (St Louis, MO, USA). All chemicals used were of analytical grade.

Experimental animals

Male Wistar rats, weighing 200 ± 20 g and purchased from Experimental Animal Center of Harbin Medical University, were used. Animals were allowed to adapt to their environment for 1 week before experiments were started. All animal procedures were approved by the Ethical Committee for Animal Experiments, Harbin Medical University.

Development of dyslipidaemic–diabetic rats

Dyslipidaemic–diabetic rats were induced by intragastric administration of high-cholesterol chow (sucrose 10%, lard 20%, Tween 80 20%, propylene glycol 30%, propacil 1%, monosodium glutamate 5%, plus water to 100%) for 2 weeks and then a single intraperitoneal injection of STZ (55 mg/kg), which was dissolved in sodium citrate buffer (pH 4.5).[19] Blood glucose was measured 72 h after STZ injection. Rats showing fasting blood glucose levels ≥ 16.7 mmol/L were selected for the study. The dyslipidaemic–diabetic rats were randomly divided into six groups ($n = 8$ in each group), as follows: (i) dyslipidaemic–diabetic experimental model group; (ii) high-dose (80 mg/kg per day) emodin-treated group; (iii) medium-dose (40 mg/kg per day) emodin-treated group; (iv) low-dose (20 mg/kg per day) emodin-treated group; (v) an insulin-treated group; and (vi) a rosiglitazone (0.83 mg/kg per day)-treated group. Emodin was dissolved in water and administered intragastrically. Insulin-treated rats received a single injection of long-acting insulin per day, with a total of 4 U insulin. Experimental rats were treated with different drugs for 2 weeks. The remaining rats were randomly divided into two groups, with one group fed normal chow (control) and the other group fed the high-cholesterol chow (hyperlipidaemic group) for a period of 2 weeks. Except for the 12 h period before blood collection, rats had free access to food and water.

Determination of blood glucose and lipid metabolic parameters

Blood samples were collected from the tip of tail on Days 1, 4, 7, 10 and 14. Blood glucose was determined using a blood glucose meter (Weyerhaeuser, Tacoma, WA, USA). Total cholesterol (TC) and triglycerides (TG) were measured by the enzyme end-point method. Briefly, in this method, the substrate to be determined of an enzymatic reaction is measured by permitting the enzymatic substrate reaction to proceed to completion (i.e. until all the substrate has reacted). Malonaldehyde (MDA), superoxide dismutase (SOD) and free fatty acid (FFA) were also measured using commercially available reagent kits. All parameters were determined using a photometer (ECOM-F 6124; Eppendorf, Hamburg, Germany).

Immunofluorescent staining

Tissue sections were cryosectioned at 5 μm, fixed in 4% paraformadehyde for 30 min, rinsed with phosphate-buffered saline (PBS) and blocked with 1% bovine serum albumin (BSA) and 2% goat serum for 1 h at room temperature. For double-labelling, sections were incubated with rabbit anti-rat $Ca_v1.2$ (1 : 200 dilution) antibody overnight at 4°C. Sections were then rinsed in PBS and further incubated with fluorescein isothiocyanate (FITC)-conjugated goat anti-rabbit antibody (1 : 500 dilution) for 2 h at room temperature. After extensive washing with PBS (twice for 15 min each time), the double-stained tissue sections on slides were sealed with coverslips ready for observation. Images were captured using a fluorescence microscope and analysed by ImageProPlus software (Media Cybernetics, Silver Spring, MD, USA). Control experiments were performed without primary antibody treatment to exclude the possibility of non-specific labelling and cross-reactivity of secondary antibodies with primary antibodies.

Analysis of relative expression of $Ca_v1.2$ by reverse transcriptase–polymerase chain reaction

Total RNA was extracted from fresh frozen myocardium using Trizol Reagent (Invitrogen, Carlsbad, CA, USA) according to the manufacturer's instructions. Random hexamer primers (Promega, Madison, WI, USA) were used for cDNA preparation using the Thermoscript reverse transcriptase (Invitrogen). The polymerase chain reaction (PCR) was performed under the following conditions: denaturation at 95°C for 2 min, annealing at 57.5°C for 1.5 min and extension at 72°C for 2 min for a total of 31 cycles. The following primers were used: forward primer 5′-CGGCACCCTCTTACCTTT-3′; reverse primer 5′-AACCCATTAGGAACATTGAAAC-3′. The forward and reverse primers for the housekeeping gene β-actin were 5′-CATCTCTT-GCTCGAAGTCCA-3′ and 5′-ATCATGTTTGAGACCTTCAACA-3′, respectively. Positive and negative reverse transcription–polymerase chain reaction (RT-PCR) controls containing standardized RNA extracts and nuclease-free water, respectively, were included in each run. Amplified products were separated by 1.5% agarose gel electrophoresis and visualized by ethidium bromide staining.

Statistical analysis

Data are expressed as the mean±SEM. Statistical analysis was performed by one-way ANOVA. Tukey's post hoc test was used for multiple group comparisons and Student's *t*-test was used for single comparisons. Statistical significance was considered at $P < 0.05$.

RESULTS

Effects of emodin on blood glucose concentrations in dyslipidaemic–diabetic rats

As shown in Fig. 2, blood glucose levels in dyslipidaemic–diabetic rats were significantly higher than in the control group ($P < 0.01$), but no significant differences were found for blood glucose levels between the hyperlipidaemic and control groups. After 2 weeks of treatment with emodin, a dose-dependent decrease in blood glucose was observed, with levels in the high-dose group going from 30.1 ± 1.2 to 15.8 ± 1.6 mmol/L ($n = 8$; $P < 0.01$), from 27.2 ± 1.4 to 22.1 ± 1.3 mmol/L in the medium-dose group ($n = 8$; $P < 0.05$) and from 26.0 ± 1.5 to 23.6 ± 1.5 mmol/L in the low-dose group ($n = 8$; $P > 0.05$). Figure 2 also shows that emodin had a mild hypoglycaemic effect and that the effects of the medium and low doses of emodin were not significant, as were the results obtained with the positive control drugs insulin and rosiglitazone.

Effects of emodin on lipid metabolic parameters

The effects of different doses of emodin on fasting plasma lipids are given in Table 1. In the high- and medium-dose emodin groups, TC, TG, FFA and MDA levels were significantly reduced and SOD levels were significantly elevated ($P < 0.05$) compared with

© 2008 The Authors
Journal compilation © 2008 Blackwell Publishing Asia Pty Ltd

Table 1 Effects of 2 weeks treatment with emodin on serum lipid parameters in dyslipidaemic–diabetic rats

	TC (mg/dL)	TG (mmol/L)	FFA (nmol/L)	MDA (nmol/L)	SOD (U/mL)
Control	61.2 ± 4.3	0.86 ± 0.08	0.55 ± 0.01	5.25 ± 0.09	122.7 ± 7.4
DDr	111.1 ± 7.0**	1.79 ± 0.12**	1.12 ± 0.05	8.36 ± 0.05**	54.6 ± 3.5**
Hyperlipidaemia	191.2 ± 29.2*	1.85 ± 0.15*	1.16 ± 0.06*	8.74 ± 0.51**	76.4 ± 5.3**
Emodin					
80 mg/kg per day	66.3 ± 0.2††	0.90 ± 0.02††	0.59 ± 0.04††	4.24 ± 0.34††	118.9 ± 4.9††
40 mg/kg per day	83.0 ± 3.0††	1.29 ± 0.06††	0.74 ± 0.02††	6.59 ± 0.16††	103.8 ± 4.7††
20 mg/kg per day	131.2 ± 15.5	1.95 ± 0.28	0.97 ± 0.08	8.09 ± 0.35	60.7 ± 9.9
Rosiglitazone	74.5 ± 2.0††	1.35 ± 0.14	0.78 ± 0.02†	6.30 ± 0.32†	106.2 ± 3.0††
Insulin	149.9 ± 15.1	2.09 ± 0.23	1.01 ± 0.04	8.93 ± 0.17	66.2 ± 6.0

Data are expressed as mean±SEM ($n = 8$ in each group). *$P < 0.05$, **$P < 0.01$ compared with control; †$P < 0.05$, ††$P < 0.01$ compared with dyslipidaemic–diabetic rats (DDr).

TC, total cholesterol; TG, triglycerides; FFA, free fatty acids; MDA, malonaldehyde; SOD, superoxide dismutase.

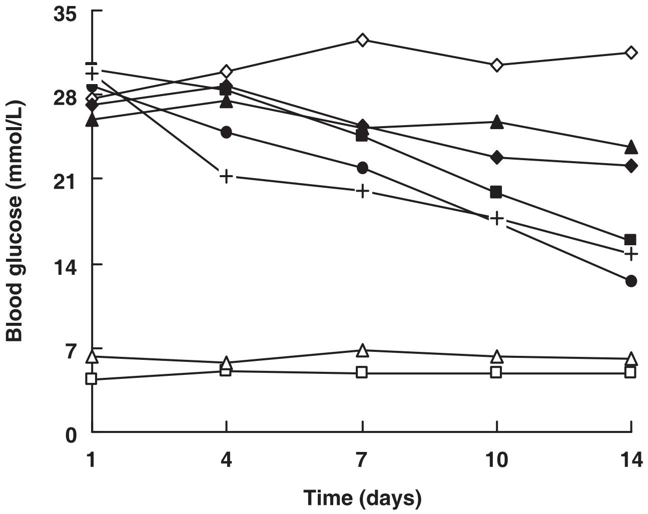

Fig. 2 Effect of emodin on blood glucose concentrations in dyslipidaemic–diabetic rats. For the sake of clarity and clearer comparisons among different groups, only mean values ($n = 8$ per group) are shown. (□), control; (◇), dyslipidaemic–diabetic rats; (△), hyperlipidaemia; (■), high-dose (80 mg/kg per day) emodin; (◆), medium-dose (40 mg/kg per day) emodin; (▲), low-dose (20 mg/kg per day) emodin; (●), rosiglitazone; (+), insulin.

dyslipidaemic–diabetic rats. The lipid-modulating effects of the high and medium doses of emodin were stronger than those of rosiglitazone (Table 1). These results demonstrate a hypolipidaemic action of emodin.

Immunofluorescent staining of $Ca_v1.2$ in pancreatic islets and cardiac myocytes

Expression of $Ca_v1.2$ was characterized using immunofluorescence staining. The overall size of the islets of diabetic rats tended to be smaller and the intensity of the immunoreactivity was markedly weaker compared with that in the control group. After 2 weeks treatment with emodin, the intensity of the immunoreactivity was enhanced and the number of the islets was increased, particularly in the high-dose emodin group; however, the intensity of the immunoreactivity and the number of islets were still below normal (Fig. 3). These results suggest that pancreatic islets were damaged and that the expression of $Ca_v1.2$ was downregulated in diabetic rats, and that emodin is able to restore the morphological changes and expression of $Ca_v1.2$ in hyperlipidaemic–diabetic rats. Furthermore, cardiac hypertrophy was evident, as an increased length and width of cardiac myocytes, in hyperlipidaemic–diabetic rats (Fig. 4) and the intensity of $Ca_v1.2$ immunoreactivity was significanlty weaker, with an overall smaller $Ca_v1.2$ immunofluorescent area compared with that in the control group. Interestingly, emodin treatment significantly increased both the intensity of the immunofluorescence and the $Ca_v1.2$ immunofluorescent area.

$Ca_v1.2$ expression in the heart

The effect of emodin on $Ca_v1.2$ mRNA expression is shown in Fig. 5. The expression of $Ca_v1.2$ was decreased in dyslipidaemic–diabetic rats. After treatment with 80 mg/kg per day emodin, the expression of $Ca_v1.2$ was increased (Fig. 5). These results further confirm that $Ca_v1.2$ expression is upregulated in emodin-treated rats.

DISCUSSION

In the present study, we demonstrated that emodin has hypoglycaemic and hypolipidaemic activity in dyslipidaemic–diabetic rats. Administration of emodin was also associated with a significant improvement of the expression of L-type calcium channels in the pancreas and heart, which may be the mechanism of action of emodin.

The dyslipidaemic–diabetic rat model was used in the present study. It has been reported that high-fat feeding leads to insulin resistance and STZ injection damages pancreatic β-cells, causing a reduction in serum insulin levels.[20] The animal model induced by STZ injection plus the high-fat diet is regarded as an ideal model for type 2 diabetes that closely reflects the natural history and metabolic characteristics of human type 2 diabetes, as well as responding to pharmacological treatments.[12] We explored the hypoglycaemic and hypolipidaemic effects of emodin and its possible mechanism of action using this model.

The data demonstrate that emodin deceases blood glucose levels at doses of 80 and 40 mg/kg per day. Moreover, emodin reduced serum TC, TG, MDA, FFA and elevated serum SOD. It has been

© 2008 The Authors
Journal compilation © 2008 Blackwell Publishing Asia Pty Ltd

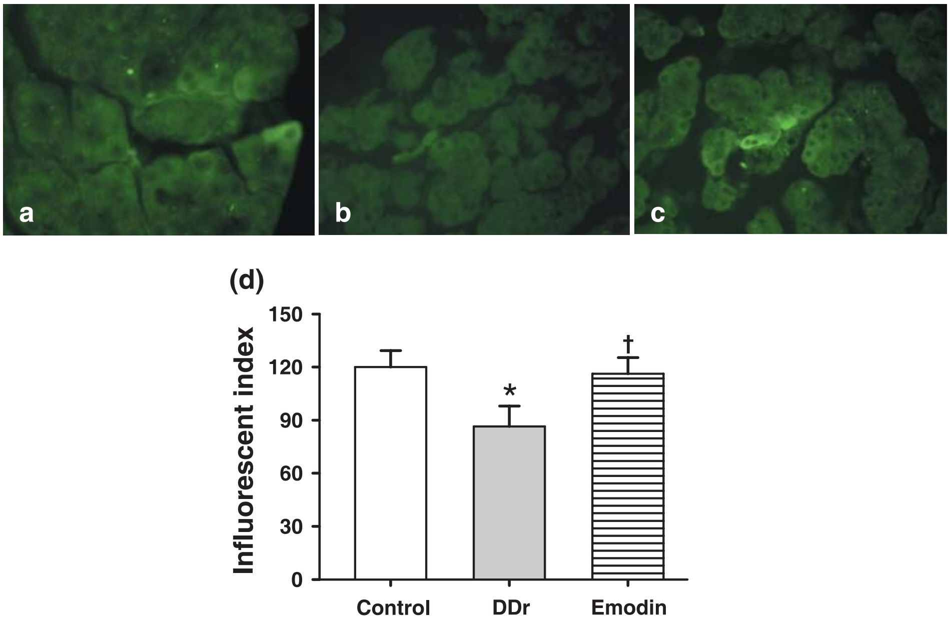

Fig. 3 (a–c) Immunofluorescence analysis of $Ca_v1.2$ in the pancreas (original magnification ×400) of (a) control rats, (b) dyslipidaemic–diabetic rats and (c) the high-dose (80 mg/kg per day) emodin-treated group. (d) Changes in immunofluorescent intensity for $Ca_v1.2$ in the pancreas. Data are the mean±SEM of three separate experiments. $^{*}P < 0.05$ compared with control; $^{\dagger}P < 0.05$ compared with dyslipidaemic–diabetic rats (DDr).

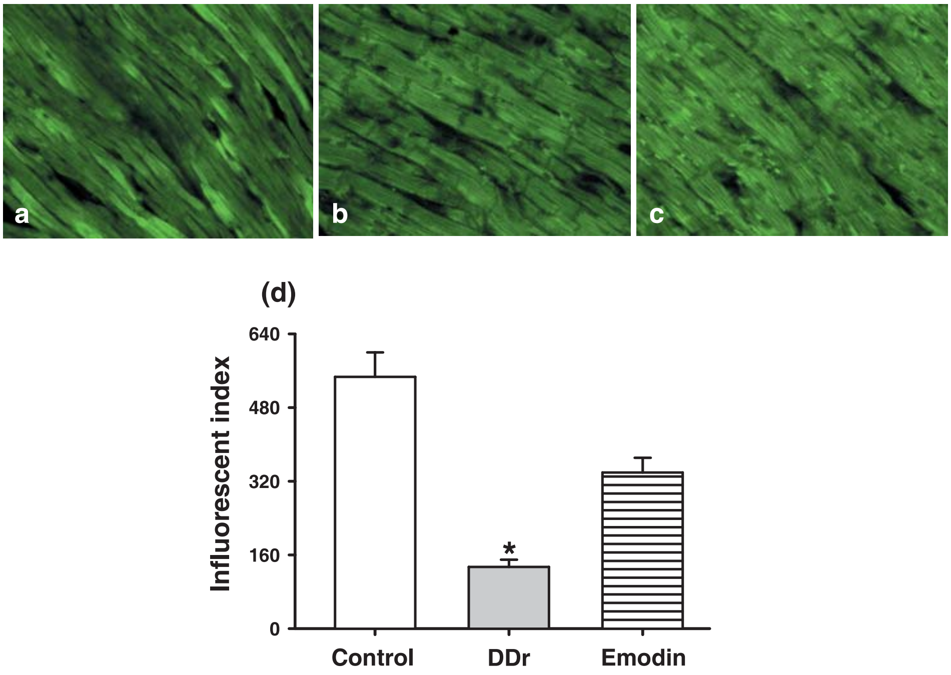

Fig. 4 Immunofluorescence analysis of $Ca_v1.2$ in cardiac myocytes (original magnification ×200) of (a) control rats, (b) dyslipidaemic–diabetic rats and (c) the high-dose (80 mg/kg per day) emodin-treated group. (d) Changes in immunofluorescent intensity for $Ca_v1.2$ in the heart. DDr, dyslipidaemic–diabetic rats. Data are the mean±SEM ($n = 8$ per group). $^{*}P < 0.05$ compared with control.

documented that lipid accumulation in non-adipose tissues, such as the pancreatic islets, liver and heart, is often associated with type 2 diabetes and its complications,[21] so the observed reduction in serum lipids after emodin treatment could help to significantly reduce the risk of cardiovascular disease. In addition, because elevated serum FFA concentrations are associated with insulin resistance, increased FFA contribute to hyperglycaemia by antagonizing the effects of insulin on endogenous glucose production.[22,23] In addition, FFA affect insulin secretion.[24] Reduction of FFA was found after treatment with emodin, which implies that emodin may improve the secretion of insulin in patients with diabetes mellitus. It has been reported that oxidative stress, leading to an increased production of reactive oxygen species (ROS), is increased in diabetes.[25] It has also been reported that SOD plays an important role in maintaining physiological levels of oxygen and hydrogen peroxide by hastening the dismutation of oxygen radicals and eliminating organic peroxides and hydroperoxides generated from inadvertent exposure to STZ.[26] In the present study, emodin treatment increased SOD activity, which further confirms that emodin exerts a beneficial action against pathological changes caused by the presence of free radicals in the STZ-induced diabetic rat model. The published evidence shows that gluco- and lipotoxicity play pivotal roles in the development of diabetes and diabetic complications;[27,28] thus, the hypoglycaemic and hypolipidaemic effects of emodin may prevent the deleterious effects of hyperglycaemia and hyperlipidaemia on the development of diabetes and diabetic complications.

© 2008 The Authors
Journal compilation © 2008 Blackwell Publishing Asia Pty Ltd

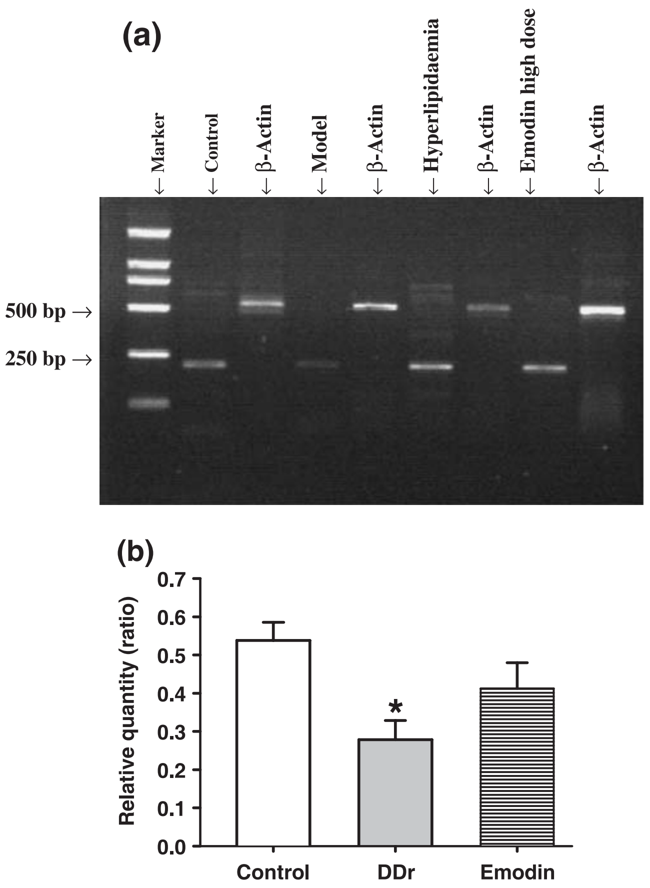

Fig. 5 Effect of emodin on $Ca_v1.2$ in cardiac myocytes. Polymerase chain reaction products were analysed on an ethidium bromide-stained 1.5% agarose gel. (a) The PCR products are 205 bp for $Ca_v1.2$ in cardiac myocytes and 500 bp for β-actin (internal standard). (b) Densitometric measurement of $Ca_v1.2$ expression in control, dyslipidaemic–diabetic rats (DDr) and high-dose (80 mg/kg per day) emodin-treated rats. Relative quantity is shown as $Ca_v1.2$/β-actin. Data are the mean±SEM ($n = 8$ per group). *$P < 0.05$ compared with the control group.

Collectively, the findings of the present study indicate that emodin has potential therapeutic effects in diabetes. Thus, it is important to determine the mechanisms of action of emodin to fully understand its therapeutic actions and to develop a new target compound. So, in the present study, we next investigated the possible mechanisms underlying the hypoglycaemic and hypolipidaemic actions of emodin. It has been reported that insulin release from pancreatic β-cell consists of a rapid first phase that lasts for approximately 10 min before declining to near basal levels, followed by a less prominent but sustained second phase of insulin secretion that can last for several hours.[29] The cellular mechanisms underlying biphasic insulin release remain unclear, but an increase in intracellular Ca^{2+} concentration ($[Ca^{2+}]_i$) is required for both the first and second phases of insulin secretion.[30] This increase in $[Ca^{2+}]_i$ is the result of Ca^{2+} influx through voltage-gated Ca^{2+} channels, which consequently close ATP-dependent K^+ channels, depolarize the membrane and further open voltage-dependent Ca^{2+} channels.[31] Type 2 diabetes is associated with a shift from biphasic to monophasic insulin release[32] and it is therefore important to explore the changes in voltage-gated Ca^{2+} channels in type 2 diabetes. Voltage-gated Ca^{2+} channels are divided into three subfamilies: (i) the HVA Ca^{2+} channel family, which includes $Ca_v1.1$, 1.2, 1.3 and 1.4 channels and is inhibited by dihydropyridines (DHPs);[33] (ii) non-L-type HVA channels $Ca_v2.1$ (P/Q-type), 2.2 (N-type) and 2.3 (R-type), which are sensitive to ω-agatoxin IVA, ω-conotoxin GVIA and SNX482, respectively; and (iii) the low voltage-activated (LVA) T-type Ca^{2+} channel family ($Ca_v3.1$, 3.2 and 3.3).[34] The molecular identity of the β-cell L-type Ca^{2+} channel involved in insulin secretion has been debated; however, recent studies have shown that $Ca_v1.2$ or α_{1C} channels played a central role in this process.[35,36] Myocardial dysfunctin is common in diabetic individuals.[37] Ca^{2+} influx through L-type Ca^{2+} channels also plays an important role in cardiac excitation–contraction coupling.[38] Our data show that the expression of $Ca_v1.2$ channels is significantly downregulated in the pancreas and heart in the diabetic rats and was restored to different degrees after emodin treatment. These data suggest that emodin ameliorates $Ca_v1.2$ expression in the pancreas and heart, which may be one of the mechanisms underlying the antidiabetic action of emodin. Thus, $Ca_v1.2$ may be a new therapeutic target for developing antidiabetic and antihyperlipidaemic drugs. However, further studies are needed to confirm this hypothesis.

There are several limitations of the present study. First, characterization of $Ca_v1.2$ expression by RT-PCR was performed only in the heart and we were not able to provide information regarding detailed changes in $Ca_v1.2$ in the pancreas. It is important to obtain this information in future studies. Second, immunofluorescent staining and RT-PCR are semiquantitative methods and our data fail to address the changes precisely; western blotting is need to address these issues accurately. Third, the link between the enhancement of L-type Ca^{2+} channel expression and the blood sugar-lowering effect of emodin is still unclear. A future study should measure insulin levels, which should provide a link between the enhanced L-type Ca^{2+} channel expression and the blood sugar-lowering effects of emodin. In conclusion, the effects of emodin on blood glucose and lipid parameters in diabetes should be confirmed through further animal experiments. Further studies will be needed to identify the molecular link between the enhanced L-type Ca^{2+} channel expression and the blood sugar-lowering effects of emodin.

The findings in this study provide information regarding the action of emodin in the clinical treatment of diabetes mellitus. Furthermore, the study demonstrated that $Ca_v1.2$ may be a new therapeutic target in the treatment of diabetes.

ACKNOWLEDGEMENTS

This work was supported by the National Basic Research program of China (973 programs: 2007CB512000/2007CB512006, to BY), the National Natural Science Foundation of China (30672644) and the Key Technologies R&D Program of Heilongjiang Province (2006G2215-00/GC06C33302).

REFERENCES

1. Kharitonenkov A, Shiyanova TL, Koester A *et al.* FGF-21 as a novel metabolic regulator. *J. Clin. Invest.* 2005; **115**: 1627–35.
2. Gikas A, Sotiropoulos A, Panagiotakos D, Peppas T, Skliros E, Peppas S. Prevalence, and associated risk factors, of self-reported diabetes mellitus in a sample of adult urban population in Greece: MEDICAL Exit Poll Research in Salamis (MEDICAL EXPRESS 2002). *BMC Public Health* 2004; **4**: 2.

© 2008 The Authors
Journal compilation © 2008 Blackwell Publishing Asia Pty Ltd

3. Wagstaff AJ, Goa KL. Rosiglitazone. A review of its use in the management of type 2 diabetes mellitus. *Drugs* 2002; **62**: 1805–37.
4. Maiti R, Das UK, Ghosh D. Attenuation of hyperglycemia and hyperlipidemia in streptozotocin-induced diabetic rats by aqueous extract of seed of *Tamarindus indica*. *Biol. Pharm. Bull.* 2005; **28**: 1172–6.
5. Kameswara Rao B, Renuka Sudarshan P, Rajasekhar MD, Nagaraju N, Appa Rao CH. Antidiabetic activity of *Terminalia pallida* fruit in alloxan induced diabetic rats. *J. Ethnopharmacol.* 2003; **85**: 169–72.
6. Srinivas G, Anto RJ, Srinivas P, Vidhyalakshmi S, Senan VP, Karunagaran D. Emodin induces apoptosis of human cervical cancer cells through poly (ADP-ribose) polymerase cleavage and activation of caspase-9. *Eur. J. Pharmacol.* 2003; **473**: 117–25.
7. Zhang XP, Li ZF, Liu XG *et al.* Effects of emodin and baicalein on rats with severe acute pancreatitis. *World J. Gastroenterol.* 2005; **11**: 2095–100.
8. Parker A, Meyer J, Lewitzky S *et al.* A gene conferring susceptibility to type 2 diabetes in conjuction with obesity is located on chromosome 18pll. *Diabetes* 2001; **50**: 675–80.
9. Kato S, Ishida H, Tsuura Y *et al.* Alterations in basal and glucose-stimulated voltage-depended Ca^{2+} channel activities in pancreatic beta cells of non-insulin-dependent diabetes mellitus GK rats. *J. Clin. Invest.* 1996; **97**: 2417–25.
10. Watkins PJ. Cardiovascular disease, hypertension, and lipids. *BMJ* 2003; **326**: 874–6.
11. Strapans J, Rapp JH, Pan XM, Feingold KR. The effect of oxidized lipids in the diet on serum lipoprotein peroxides in control and diabetic rats. *J. Clin. Invest.* 1993; **92**: 638–43.
12. Srinivasan K, Viswanad B, Asrat L, Kaul CL, Ramarao P. Combination of high-fat diet-fed and low-dose streptozotocin-treated rat. A model for type 2 diabetes and pharmacological screening. *Pharmacol. Res.* 2005; **52**: 313–20.
13. Reed MJ, Meszaros K, Entes LJ *et al.* A new rat model of type 2 diabetes: The fat fed, streptozotocin-treated rat. *Metabolism* 2000; **49**: 1390–4.
14. Mears D. Regulation of insulin secretion in islets of Langerhans by Ca^{2+} channels. *J. Membr. Biol.* 2004; **200**: 57–66.
15. Catterall WA. Structure and function of neuronal Ca^{2+} channels and their role in neurotransmitter release. *Cell Calcium* 1998; **24**: 307–23.
16. Sinnegger-Brauns MJ, Hetzenauer A, Huber IG *et al.* Isoform-specific regulation of mood behavior and pancreatic β cell and cardiovascular function by L-type Ca^{2+} channels. *J. Clin. Invest.* 2004; **113**: 1430–9.
17. An D, Rodrigues B. Role of changes in cardiac metabolism in development of diabetic cardiomyopathy. *Am. J. Physiol. Heart Circ. Physiol.* 2006; **291**: H1489–506.
18. Liu Y, Shan HL, Sun HL, He SZ, Yang BF. Effects of emodin on the intracellular calcium concentration ($[Ca^{2+}]_i$) and L-type calcium current of the single ventricular mytocytes from guinea pig. *Yao Xue Xue Bao* 2004; **39**: 5–8 (in Chinese).
19. Pascoe WS, Jenkins AB, Kusunoki M, Stolein LH. Insulin action and determinants of glycaemia in a rat model of type 2 (non-insulin-dependent) diabetes mellitus. *Diabetologia* 1992; **35**: 208–15.
20. Chalkley SM, Hettiarachchi M, Chisholm DJ, Kraegen EW. Long-term high-fat feeding leads to severe insulin resistance but not diabetes in Wistar rats. *Am. J. Physiol. Endocrinol. Metab.* 2002; **282**: E1231–8.
21. Unger RH, Zhou YT. Lipotoxicity of beta-cells in obesity and in other causes of fatty acid spillover. *Diabetes* 2001; **50** (Suppl. 1): S118–21.
22. Getty L, Panteleon AE, Mittelman SD, Dea MK, Bergman RN. Rapid oscillations in omental lipolysis are independent of changing insulin levels *in vivo*. *J. Clin. Invest.* 2000; **106**: 421–30.
23. Rader DJ. Effect of insulin resistance, dyslipidemia, and intra-abdominal adiposity on the development of cardiovascular disease and diabetes mellitus. *Am. J. Med.* 2007; **120** (Suppl.): S12–18.
24. Boden G, Shulman GI. Free fatty acids in obesity and type 2 diabetes: Defining their role in the development of insulin resistance and beta-cell dysfunction. *Eur. J. Clin. Invest.* 2002; **32** (Suppl. 3): 14–23.
25. Nishikawa T, Araki E. Impact of mitochondrial ROS production in the pathogenesis of diabetes mellitus and its complications. *Antioxid. Redox Signal.* 2007; **9**: 343–53.
26. Bolzan AD, Bianchi MS. Genotoxicity of STZ. *Mutat. Res.* 2002; **512**: 121–34.
27. Dyntar D, Eppenberger-Eberhardt M, Maedler K *et al.* Glucose and palmitic acid induce degeneration of myofibrils and modulate apoptosis in rat adult cardiomyocytes. *Diabetes* 2001; **50**: 2105–13.
28. Rayasam GV, Tulasi VK, Davis JA, Bansal VS. Fatty acid receptors as new therapeutic targets for diabetes. *Expert Opin. Ther. Targets* 2007; **11**: 661–71.
29. Wajchenberg BL. Beta-cell failure in diabetes and preservation by clinical treatment. *Endocr. Rev.* 2007; **28**: 187–218.
30. Henquin JC, Ravier MA, Nenquin M, Jonas JC, Gilon P. Hierarchy of the β cell signals controlling insulin secretion. *Eur. J. Clin. Invest.* 2003; **33**: 742–50.
31. Finlin BS, Mosley AL, Crump SM *et al.* Regulation of L-type Ca^{2+} channel activity and insulin secretion by the Rem2 GTPase. *J. Biol. Chem.* 2005; **280**: 41 864–71.
32. Del Prato S, Marchetti P, Bonadonna RC. Phasic insulin release and metabolic regulation in type 2 diabetes. *Diabetes* 2002; **51** (Suppl. 1): S109–16.
33. Dolphin AC. L-Type calcium channel modulation. *Adv. Second Messenger Phosphoprotein Res.* 1999; **33**: 153–77.
34. Reid CA, Bekkers JM, Clements JD. Presynaptic Ca^{2+} channels: A functional patchwork. *Trends Neurosci.* 2003; **26**: 683–7.
35. Sinnegger-Brauns MJ, Hetzenauer A, Huber IG *et al.* Isoform-specific regulation of mood behavior and pancreatic beta cell and cardiovascular function by L-type Ca^{2+} channels. *J. Clin. Invest.* 2004; **113**: 1430–9.
36. Schulla V, Renstrom E, Feil R *et al.* Impaired insulin secretion and glucose tolerance in β cell-selective $Ca_v1.2$ Ca^{2+} channel null mice. *EMBO J.* 2003; **22**: 3844–54.
37. Finck BN, Han X, Courtois M *et al.* A critical role for PPARalpha-mediated lipotoxicity in the pathogenesis of diabetic cardiomyopathy: Modulation by dietary fat content. *Proc. Natl Acad. Sci. USA* 2003; **100**: 1226–31.
38. Bers DM. Cardiac excitation–contraction coupling. *Nature* 2002; **415**: 198–205.

© 2008 The Authors
Journal compilation © 2008 Blackwell Publishing Asia Pty Ltd
