# Supplementary material for: Emodin, a rising star in the treatment of glycolipid metabolism disorders: a preclinical systematic review and meta-analysis
Source: PeerJ. 2025 May 15;13:e19221. doi: 10.7717/peerj.19221 (PMC12085882; doi:10.7717/peerj.19221)
Supplement: Supplemental Information 20 [file peerj-13-19221-s020.zip › 8篇英文文献/Yue-jing Wang2012.pdf]

Acta Pharmacologica Sinica (2012) 33: 1195–1203
© 2012 CPS and SIMM All rights reserved
1671-4083/12 $32.00
www.nature.com/aps

Original Article

Emodin, an 11β-hydroxysteroid dehydrogenase type 1 inhibitor, regulates adipocyte function *in vitro* and exerts anti-diabetic effect in *ob/ob* mice

Yue-jing WANG, Su-ling HUANG, Ying FENG, Meng-meng NING, Ying LENG*

State Key Laboratory of Drug Research, Shanghai Institute of Materia Medica, Chinese Academy of Sciences, Shanghai 201203, China

Aim: Emodin (1,3,8-trihydroxy-6-methylanthraquinone) is a potent and selective inhibitor of 11β-hydroxysteroid dehydrogenase type 1 (11β-HSD1) with the ability to ameliorate metabolic disorders in diet-induced obese mice. In the present study, we investigated the effects of emodin on adipocyte function and the underlying mechanisms *in vitro*, and its anti-diabetic effects in *ob/ob* mice.
Methods: 3T3-L1 adipocytes were used for *in vitro* studies. 11β-HSD1A activity was evaluated with a scintillation proximity assay. The adipogenesis, glucose uptake, lipolysis and adiponectin secretion were investigated in 3T3-L1 adipocytes treated with emodin in the presence of active (corticosterone) or inactive glucocorticoid (11-dehydrocorticosterone). For *in vivo* studies, *ob/ob* mice were administered emodin (25 and 50 mg·kg^{-1}·d^{-1}, ip) for 26 d. On the last day of administration, the serum was collected and the mesenteric and perirenal fat were dissected for analyses.
Results: Emodin inhibited the 11β-HSD1 activity in 3T3-L1 adipocytes in concentration- and time-dependent manners (the IC_{50} values were 7.237 and 4.204 μmol/L, respectively, after 1 and 24 h treatment. In 3T3-L1 adipocytes, emodin (30 μmol/L) suppressed 11-dehydrocorticosterone-induced adipogenesis without affecting corticosterone-induced adipogenesis; emodin (3 μmol/L) reduced 11-dehydrocorticosterone-stimulated lipolysis, but had no effect on corticosterone-induced lipolysis. Moreover, emodin (3 μmol/L) partly reversed the impaired insulin-stimulated glucose uptake and adiponectin secretion induced by 11-dehydrocorticosterone but not those induced by corticosterone. In *ob/ob* mice, long-term emodin administration decreased 11β-HSD1 activity in mesenteric adipose tissues, lowered non-fasting and fasting blood glucose levels, and improved glucose tolerance.
Conclusion: Emodin improves the inactive glucocorticoid-induced adipose tissue dysfunction by selective inhibition on 11β-HSD1 in adipocyte *in vitro* and improves glycemic control in *ob/ob* mice.

Keywords: emodin; 11β-hydroxysteroid dehydrogenase type 1; adipocyte; glucocorticoid; type 2 diabetes; *ob/ob* mice

Acta Pharmacologica Sinica (2012) 33: 1195–1203; doi: 10.1038/aps.2012.87; published online 27 Aug 2012

Introduction

Adipose tissue plays a key role in regulating energy balance and glucose homeostasis. As an energy storage depot, adipose tissue responds to the body's metabolic signaling by regulating lipid storage and mobilization. Adipocytes release free fatty acid (FFA) as a nutrient source when glucose is limiting, whereas they store abundant energy as triglycerides in energy excess states. Insulin resistance can elevate the FFA level, and excessive FFA induces a deterioration in the metabolic state by accelerating liver glucose output and by inhibiting glucose uptake by peripheral tissues and the generation of reactive oxygen system (ROS), which, in turn, aggravates insulin resistance[1]. Adipose tissue is an endocrine organ that releases several adipokines, such as leptin, adiponectin, visfatin, omentin, and resistin, to regulate glucose homeostasis and whole body insulin sensitivity [1]. Thus, adipocyte dysfunction is thought to be involved in the pathogenesis of obesity and metabolic diseases such as type 2 diabetes[2].

Glucocorticoid (GC) is an insulin-antagonizing hormone that stimulates hepatic glucose production and suppresses insulin-mediated glucose uptake in peripheral tissues such as adipose tissue and skeletal muscle. Glucocorticoid excess, which is well-characterized in Cushing's syndrome, produces central obesity and several clinical features associated with insulin resistance, such as type 2 diabetes, dyslipidemia, and hypertension[3]. The action of glucocorticoid on target tissue is determined not only by the circulating glucocorticoid level but also by the local glucocorticoid activation, which is regulated

* To whom correspondence should be addressed.
E-mail yleng@mail.shcnc.ac.cn
Received 2012-05-01 Accepted 2012-05-28

by the 11β-hydroxysteroid dehydrogenase type 1 (11β-HSD1) and 11β-HSD2. 11β-HSD1, which is highly expressed in the liver, adipose tissue, gonads and brain, catalyze the activation of glucocorticoid (cortisol in human and corticosterone in rodents) from inactive 11-kero steroids (cortisone in human and 11-dehydrocorticosterone in rodents). This process amplifies local glucocorticoid action, whereas 11β-HSD2 is predominantly expressed in aldosterone-sensitive target tissues (in the kidney, colon, salivary glands and placenta) and catalyzes the opposite reaction[4].

Excess glucocorticoid in adipocytes decreases insulin-induced glucose uptake, promotes FFA secretion and affects adipokine profiles, thus causing insulin resistance[5]. Therefore, 11β-HSD1 is expected to play an important role in the regulation of glucose and lipid metabolism in adipose tissue. Several human studies have reported two- to three-fold increases in 11β-HSD1 activity in the adipose tissue of obese individuals, and the expression of 11β-HSD1 in adipose tissue was positively correlated with the degree of obesity[6, 7]. The contribution of 11β-HSD1 to the development of insulin resistance and obesity has been further confirmed in animal studies. Mice overexpressing adipose-specific 11β-HSD1 showed increased corticosterone in adipose tissue and developed insulin resistance, central obesity, hyperlipidemia, and other features of metabolism syndrome[8,9], whereas mice overexpressing liver-specific 11β-HSD1 only showed mild insulin resistance and dyslipidemia[10]. 11β-HSD1 knockout mice showed improved glucose tolerance, an elevated HDL, and protection from weight gain during a high-fat diet[11-13]. Moreover, overexpressing 11β-HSD2 to inactivate glucocorticoid in the adipose tissue of mice caused decreased food intake and improved glucose tolerance and insulin sensitivity under conditions of a high-fat diet[14]. Considering the above findings, the pharmacological inhibition of 11β-HSD1, especially in adipose tissue, could be a therapy for type 2 diabetes or metabolic diseases.

Emodin is an anthraquinone compound isolated mainly from the root and rhizome of *Rheum palmatum*. It demonstrates a variety of biological activities, such as anti-virus activities, anti-tumor activities, anti-inflammatory activities, and immune suppression, and it can also serve as a potential agent in therapy for liver cirrhosis, diabetic nephropathy and atherosclerosis[15–20]. Our previous study showed that emodin is a potent and selective 11β-HSD1 inhibitor and can ameliorate metabolic disorders in diet-induced obese mice[21]. In the present study, we investigated the effect of emodin on adipocyte function and the underlying mechanisms involving inhibition of 11β-HSD1. The anti-diabetic effect of emodin was also investigated in *ob/ob* mice, a genetic animal model of type 2 diabetes.

Materials and methods

Materials

Emodin was purchased from Nanjing Zelang Medical Technology Co Ltd (Nanjing, China). Corticosterone, dexamethasone, insulin, glycyrrhetinic acid and 3-isobutyl-1-methylxanthine were purchased from Sigma (St Louis, MO, USA). 11-Dehydrocorticosterone was purchased from Steraloids (Newport, RI, USA). Fetal bovine serum (FBS), penicillin/streptomycin, HEPES and high glucose Dulbecco's modified Eagle's medium (DMEM) were purchased from GibcoBRL (Grand island, NY, USA). TRIzol Reagent was purchased from Invitrogen (Carlsbad, CA, USA). The M-MLV reverse transcriptional enzyme and SYBR® Premix Ex Taq™ were obtained from Takara (Dalian, China). All the primers were synthesized by Sangon Corporation (Shanghai, China). [1,2-(n)^{3}H]-Cortisone was obtained from Amersham (Buckinghamshire, UK). SPA beads were purchased from GE (Piscataway, NJ, USA). SuperBlock Blocking Buffer was obtained from Pierce (Rockford, IL, USA). The murine monoclonal anti-cortisol antibody was purchased from East Coast Biologics (North Berwick, ME, USA).

3T3-L1 cell culture and differentiation

3T3-L1 preadipocytes were cultured and differentiated as previously described[22]. In brief, 3T3-L1 preadipocytes were maintained at ~70% confluence in DMEM supplemented with 10% FBS, 25 mmol/L glucose and antibiotics (DMEM/FBS). Cells were grown for 2 d post-confluence and cultured in DMEM/FBS supplemented with 1 μmol/L insulin, 0.25 μmol/L dexamethasone, and 0.5 mmol/L 3-isobutyl-1-methylxanthine for 3 d. The medium was replaced with DMEM/FBS supplemented with only 1 μmol/L insulin for 3 d and then DMEM/FBS alone for 2 d. Cytoplasmic triacylglycerol droplets were visible on d 5 after initiation of differentiation. The differentiated cells were used when ~90% of the cells showed an adipocyte phenotype.

11β-HSD1 enzyme activity assay

The reductase activity of 11β-HSD1 in intact 3T3-L1 adipocytes was determined by measuring the rate of conversion of cortisone to cortisol. 3T3-L1 adipocytes were incubated for 1 h at 37 °C in serum-free DMEM containing 6.25 nmol/L [1,2-(N) ^{3}H]-cortisone and different concentrations of emodin, according to experimental design, and 0.1% DMSO was set as the vehicle control. To explore the effect of emodin on 11β-HSD1 reductase activity after 24 h of treatment, the adipocytes were pretreated with emodin for 23 h before incubation. At the end of the incubation, 80 μL of medium was pipetted into a transparent bottom 96-well plate, and 35 μL of SuperBlock Blocking Buffer containing 10 g/L of protein A-coated yttrium silicate beads and 3 mg/L of anti-cortisol antibodies was added. The mixtures were shaken in the dark for 2 h and then used for liquid scintillation readings.

Adipogenesis measurement

To observe the effect of emodin on adipocyte adipogenesis, 3T3-L1 preadipocytes were initiated to differentiate with 1 μmol/L insulin, 0.5 mmol/L IBMX and different types of glucocorticoids (0.25 μmol/L dexamethasone, 0.25 μmol/L 11-DHC or 0.25 μmol/L corticosterone). The emodin or vehicle control containing 0.1% DMSO was added on the first day of differentiation, and the medium was changed every

2 d until d 8. The triglyceride content of cells was measured on d 8. After washing with ice-cold PBS, the adipocytes were collected and sonicated in distilled water. The triglyceride content of the cell lysate was measured using the GPO-PAP method, and the absorbance was monitored at 546 nm in a spectrophotometer.

Glucose uptake

3T3-L1 adipocytes were pretreated with 3 μmol/L emodin for 2 h and then incubated with 10 nmol/L corticosterone, 10 nmol/L 11-DHC or 0.1% ethanol for another 24 h. The medium containing 0.1% DMSO was used as the vehicle. At the last 0.5 h of treatment, glucose uptake was determined. The adipocytes were washed twice with pre-warmed (37 °C) PBS and incubated with Krebs-BSA buffer (140 mmol/L NaCl, 5 mmol/L KCl, 2.5 mmol/L $MgSO_4$, 1 mmol/L $CaCl_2$, 20 mmol/L HEPES, and 0.5% BSA, pH 7.4) with or without insulin (basal state) for 25 min, followed by the addition of 0.05 mmol/L 2-deoxy-*D*-glucose and 1.85×10^4 Bq/mL 2-deoxy-*D*-[1,2-^{3}H]glucose for 5 min. The assay was terminated by washing the cells three times with ice-cold PBS. The cells were solubilized with 0.1% Triton X-100, and radioactivity was determined in a liquid scintillation counter (Beckman LS6500, Fullerton, CA, USA). Total cellular protein concentration was measured by the Bradford method (BioRad, Richmond, CA, USA). Glucose uptake assays were performed in duplicate at least three times.

Lipolysis

3T3-L1 adipocytes were pretreated with 3 μmol/L emodin or 0.1% DMSO for 2 h and then incubated with 10 nmol/L corticosterone, 10 nmol/L 11-DHC or 0.1% ethanol in phenol-red free DMEM for another 48 h. The medium was collected, and the free glycerol (index of lipolysis) was measured using a Free Glycerol Determination Kit (Sigma, St Louis, MO, USA).

Adiponectin release

3T3-L1 adipocytes were pretreated with 3 μmol/L emodin or 0.1% DMSO for 2 h in serum-free DMEM and were then incubated with 50 nmol/L corticosterone, 50 nmol/L 11-DHC or 0.1% ethanol for another 48 h. After treatment, the cell medium was collected and added to 5×Laemmli Buffer. The medium was then degenerated at 56 °C for 20 min. Equal amounts of the medium were subjected to 10% SDS polyacrylamide gel electrophoresis, and the separated proteins were transferred to polyvinylidene difluoride membranes. Then, the membranes were blocked for 1 h with 7.5% non-fat milk at room temperature and incubated with an antibody against adiponectin (R&D, MN, USA) at 4 °C. After overnight incubation with the primary antibody, the blots were repeatedly washed in 0.1% TBS-Tween and incubated with a 1:10 000 dilution of an HRP-conjugated secondary antibody in 0.1% TBS-Tween for 1 h. Following further washes, proteins were detected using an ECL Plus Western Blotting Detection System (Amersham, Arlington Heights, IL, USA) and quantified by densitometry.

Animals and treatments

B6.V-*Lep^{ob}*/*Lep^{ob}* (*ob/ob*) mice and their lean littermates$^{+/+}$ (from Jackson Laboratory, Bar Harbor, ME, USA) were bred at the Shanghai Institute of Materia Medica (SIMM), Chinese Academy of Sciences. The animals were maintained under a 12-h light-dark cycle with free access to water and food. The animal experiments were approved by the Animal Care and Use Committee, Shanghai Institute of Materia Medica, Chinese Academy of Sciences.

Based on fasting blood glucose values (first criterion) and initial body weights (second criterion), the *ob/ob* mice were assigned to four groups. The *ob/ob* mice were subjected to intraperitoneal injection treatment twice daily with the vehicle (0.5% Tween 80), emodin (25 mg/kg) or emodin (50 mg/kg) for 26 d. We subjected the fourth group of *ob/ob* mice to pair feeding (providing the mice each day with the amount of food eaten by the freely fed mice treated with 50 mg/kg of emodin) on d 7. The lean mice were treated with the vehicle (0.5% Tween 80). The blood glucose levels were measured via blood drops obtained by clipping the tail of the mice using a ONE TOUCH BASIC plus Glucose Monitor (Lifescan, Milpitas, CA, USA). The food intake and body weight of the animals were recorded every 3 d. A glucose tolerance test was performed for mice deprived of food for 5 h (2.5 g/kg of glucose administered by gavage) on d 20 of the treatment. On the last day of administration, the mice were anaesthetized with an ip injection of sodium pentobarbital (40 mg/kg). The serum was collected for the determination of triacylglycerol, cholesterols and the non-esterified free fatty acid (NEFA) concentration. The mesenteric and perirenal fat were dissected, weighed, immediately frozen in liquid nitrogen and stored at -80 °C.

Real-time PCR

The mesenteric fat was homogenized in TRIZOL solution, and the total mRNA was extracted following the manufacturer's instructions. One microgram of total mRNA was reverse-transcribed in a 20-μL reaction mixture containing 4 μL 5×PrimeScript Buffer, 1 μL Primescript RT Enzyme Mix I, 1 μL Random 6 mers (100 μmol/L), 1 μL Oligo dT Primer (50 μmol/L), and 11 μL RNAse-free H_2O. The mixture was incubated at 37 °C for 15 min, then at 85 °C for 5 s. Then, real-time PCR was performed in an ABI 7500 Fast Real-Time PCR System (Applied Biosystems, CA, USA) using the SYBR® Premix Ex Taq™, and 2 μL of cDNA was amplified in a 25-μL reaction mixture containing 12.5 μL of the SYBR Premix Ex Taq, 0.5 μL of the forward primer (10 μmol/L), 0.5 μL of the reverse primers (10 μmol/L), 0.5 μL of the ROX Reference Dye II, and 9 μL of dH_2O. The amplifications were as follows: 95 °C for 30 s, followed by 40 cycles at 95 °C for 5 s, 57 °C for 20 s, and 72 °C for 15 s. The forward and reverse primers for adiponectin are 5′-AATCATTATGACGGCAGCAC-3′ and 5′-CCAGATGGAGGAGCACAGAG-3′, respectively; those for PPARγ are 5′-GGCCATCCGAATTTTTCAAG-3′ and 5′-GGGATATTTTTGGCATACTCTGTGA-3′, respectively; and those for β-actin are 5′-TGCTGTCCCTGTATGCCTCTG-3′ and 5′-TTGATGTCACGCACGATTTCC-3′, respectively. The reverse-tran-

scribed cDNA samples were amplified, and cycle threshold (C_t) values were determined. The mRNA levels of adiponectin were normalized to the mRNA levels of the housekeeping gene β-actin. The comparative C_t method ($2^{-\Delta\Delta Ct}$) was used to analyze the differences in the level of adiponectin mRNA between each group.

11β-HSD1 activity assay in mesenteric fat

A weight of 200 mg of mesenteric fat was homogenized in 200 μL of cold homogenization buffer (20 mmol/L Na_2HPO_4, 5% glycerol, 1 mmol/L EDTA, pH 7.0) at 4 ºC. The protein concentration of homogenate was determined using the Bradford method. A total of 10 μg of homogenate was pipetted into 96-well microtitre plates, and 70 μL of buffer (25 nmol/L [1,2-(N)^{3}H]-cortisone, 1.25 mmol/L NADPH and l50 mmol/L HEPES, pH 7.4) was added. The microtitre plates were shaken in the dark at 37 ºC at 140 rounds per minute for 1 h. Then, 35 μL of SuperBlocking buffer (10 g/L of protein A-coated yttrium silicate beads, 3 g/L of anti-cortisol antibody and 314 μmol/L of glycyrrhetinic acid) was added. After shaking for another 2 h, the amount of [1,2-(N)^{3}H]-cortisol generated in the 11β-HSD1 reaction was captured by beads and determined in a microplate liquid scintillation counter.

Statistical analysis

The results are expressed as the mean±SEM. The differences between the two groups were analyzed by Student's *t*-test. Statistical analysis was carried out using the Prism program from GraphPad Software. Values of $P<0.05$ were considered statistically significant.

Results

Emodin inhibited 11β-HSD1 activity in 3T3-L1 adipocytes

Emodin showed strong inhibitory effects on 11β-HSD1 activity in 3T3-L1 adipocytes. The IC_{50}s were 7.237 and 4.204 μmol/L after 1 h and 24 h of treatment, respectively (Figure 1A and 1B).

Emodin regulated adipogenesis and energy metabolism in 3T3-L1 cells

It is well documented that glucocorticoids promote preadipocyte differentiation. The triglyceride content of adipocytes was used as a marker of the adipogenesis of 3T3-L1 cells. We found that all active glucocorticoids (dexamethasone and corticosterone) and inactive glucocorticoids (11-dehydrocorticosterone, 11-DHC) enhanced adipogenesis in 3T3-L1 preadipocytes. Incubation with 30 μmol/L of emodin reduced the triglyceride levels of 3T3-L1 adipocytes induced by 11-DHC by 19.8% ($P<0.01$), but it had no significant effect on the triglyceride levels in 3T3-L1 adipocytes treated with dexamethasone or corticosterone. Therefore, emodin suppressed inactive glucocorticoid-induced adipogenesis but not active glucocorticoid-induced adipogenesis in 3T3-L1 cells, which might be mediated by the inhibition of 11β-HSD1 activity (Figure 2A).

To determine whether emodin affects lipolysis through 11β-HSD1 inhibition, 11-DHC and corticosterone were used to induce lipolysis, and the effects of emodin were studied. Figure 2B indicates that 11-DHC and corticosterone significantly increased the glycerol release by 17.8% and 18.5% ($P<0.01$), respectively. Incubation with 3 μmol/L of emodin significantly decreased the glycerol release induced by 11-DHC but not by corticosterone. These results suggest that emodin suppressed lipolysis by inhibiting 11β-HSD1 activity.

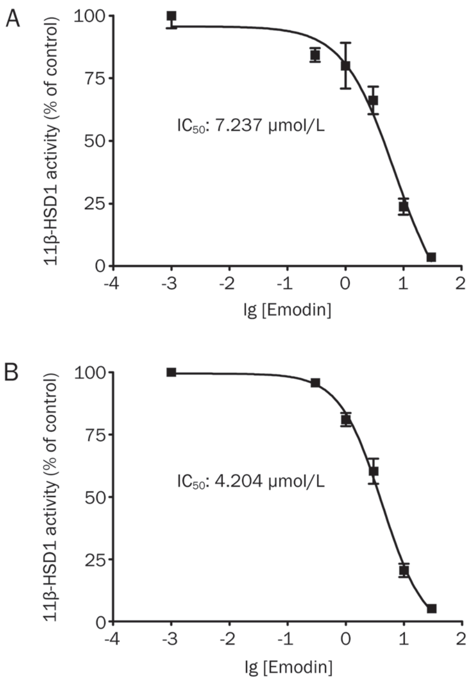

Figure 1. Emodin inhibited 11β-HSD1 activity in 3T3-L1 adipocytes after 1 h (A) or 24 h (B) of treatment. Differentiated 3T3-L1 adipocytes were incubated with the indicated concentrations of emodin or 0.1% DMSO for 1 h or 24 h. 11β-HSD1 enzyme activity was determined by scintillation proximity assay. Each point represents the mean±SEM. *n*=3.

Both 11-DHC and corticosterone attenuated insulin-stimulated glucose uptake in 3T3-L1 adipocytes (Figure 2C). Incubation with 3 μmol/L of emodin significantly reversed the impaired insulin-stimulated glucose uptake induced by 11-DHC. Although emodin treatment alone caused an 11.2% reduction in insulin-stimulated glucose uptake, it increased the insulin-stimulated glucose uptake by 17.7% when compared with 11-DHC-treated group. However, emodin had no effect on the impaired-insulin stimulated glucose uptake induced by corticosterone (Figure 2C).

Both 11-DHC and corticosterone significantly decreased adiponectin release from 3T3-L1 adipocytes. The reduced adiponectin secretion caused by 11-DHC was partly reversed by treatment with 3 μmol/L of emodin, whereas emodin exerted no effect on the impaired adiponectin secretion induced by corticosterone (Figure 2D).

Emodin suppressed 11β-HSD1 activity in adipose tissue and ameliorated metabolic disorders in *ob/ob* mice

To evaluate the long-term effect of emodin on metabolic disor-

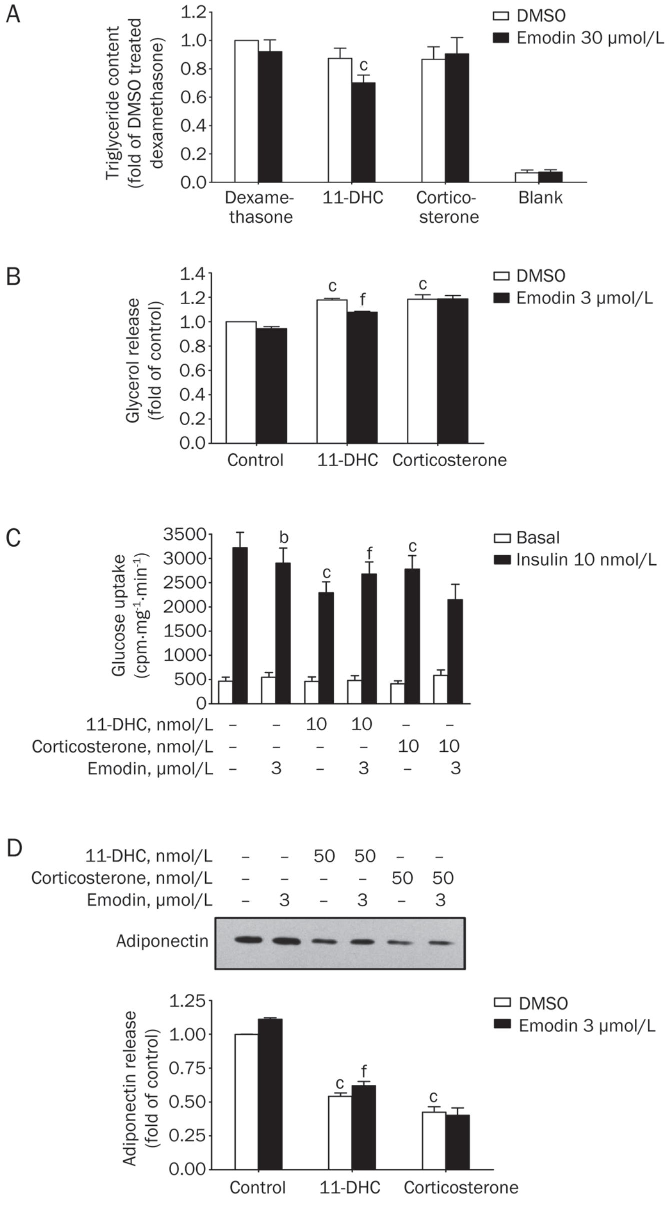

Figure 2. Emodin regulated adipogenesis and energy metabolism in 3T3-L1 adipocytes. (A) Emodin inhibited the adipogenesis induced by 11-DHC (an inactive glucocorticoid) but not by corticosterone (an active glucocorticoid). The values are expressed as the fold increase over the values for the dexamethasone-treated DMSO group. ^{c}P<0.01 *vs* DMSO-treated 11-DHC group; *n*=4. (B) Emodin inhibited the glycerol release induced by 10 nmol/L 11-DHC but not by 10 nmol/L corticosterone. The values are expressed as the fold increase over the values for the DMSO-treated control group. ^{c}P<0.01 *vs* DMSO-treated control group, ^{f}P<0.01 *vs* DMSO-treated 11-DHC group; *n*=3. (C) Emodin significantly reversed the impaired insulin-stimulated glucose uptake induced by 11-DHC but not by corticosterone. ^{b}P<0.05, ^{c}P<0.01 *vs* insulin-stimulated control group, ^{f}P<0.01 *vs* insulin stimulated 11-DHC group; *n*=6. (D) 11-DHC- but not corticosterone-impaired adiponectin release was reversed by 3 μmol/L of emodin in 3T3-L1 adipocytes. Values are expressed as the fold increase over the values for the DMSO-treated control group. ^{c}P<0.01 *vs* DMSO-treated control group, ^{f}P<0.01 *vs* DMSO-treated 11-DHC group; *n*=3.

ders, *ob/ob* mice were treated twice daily with 25 or 50 mg/kg of emodin by intraperitoneal injection for 26 d. The enzymatic activity of 11β-HSD1 in mesenteric fat was measured at the end of the experiment. As shown in Figure 3, a significant decrease in 11β-HSD1 activity, by 44.5%, was observed in the mesenteric adipose tissues of *ob/ob* mice treated with 50 mg/kg of emodin (*P*<0.05).

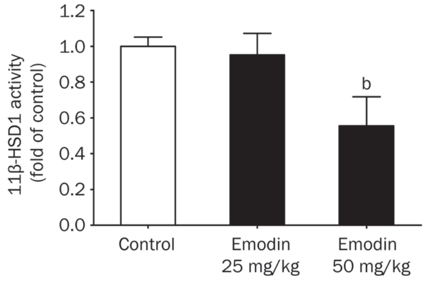

Figure 3. Emodin suppressed 11β-HSD1 activity in the mesenteric fat of *ob/ob* mice. *Ob/ob* mice were subjected to intraperitoneal injection treatment twice daily with vehicle (0.5% Tween 80), 25 mg/kg emodin or 50 mg/kg emodin for 26 d. 11β-HSD1 activity in the mesenteric fat was measured by SPA at the end of the treatment period. Values are expressed as fold of the values for the vehicle group. ^{b}P<0.05 *vs* vehicle group; *n*=6.

The genetic type 2 diabetic *ob/ob* mice showed obesity, hyperglycemia, dyslipidemia and insulin resistance. Long-term treatment with emodin significantly decreased the random-fed and fasting blood glucose levels in *ob/ob* mice, whereas the pair-fed group of mice showed no significant changes in blood glucose levels. As shown in Figures 4A and 4B, 16 or 20 d of treatment with 50 mg/kg of emodin significantly reduced the random-fed blood glucose concentrations in *ob/ob* mice by 32.53% and 32.68% (*P*<0.05), respectively, and the fasting blood glucose levels of *ob*/*ob* mice were also decreased by 38.29% (*P*<0.01) and 31.61% (*P*<0.05), respectively, when compared with the vehicle control mice. After 20 d of treatment with 50 mg/kg of emodin, glucose tolerance was improved. The emodin-treated mice exhibited significant reductions in blood glucose levels at 15, 60, and 120 min following an oral glucose challenge, and the glucose $AUC_{0-120\ min}$ value decreased by 29.11% when compared with the vehicle control group (Figure 4C and 4D, *P*<0.05). There was no significant difference in the blood glucose level or the $AUC_{0-120\ min}$ value between the emodin pair-fed and vehicle control groups. Moreover, the random-fed, fasting blood glucose levels at the treatments on d 16 and d 20 and the blood glucose levels and $AUC_{0-120\ min}$ values in OGTT of the 50-mg/kg emodin-treated group were significantly lower than in the pair-fed group (*P*<0.05, *P*<0.01).

Emodin also improved the lipid profiles in *ob/ob* mice. After 26 d of treatment with 50 mg/kg of emodin, the serum triglyceride was significantly reduced by 26.0% (*P*<0.01) compared

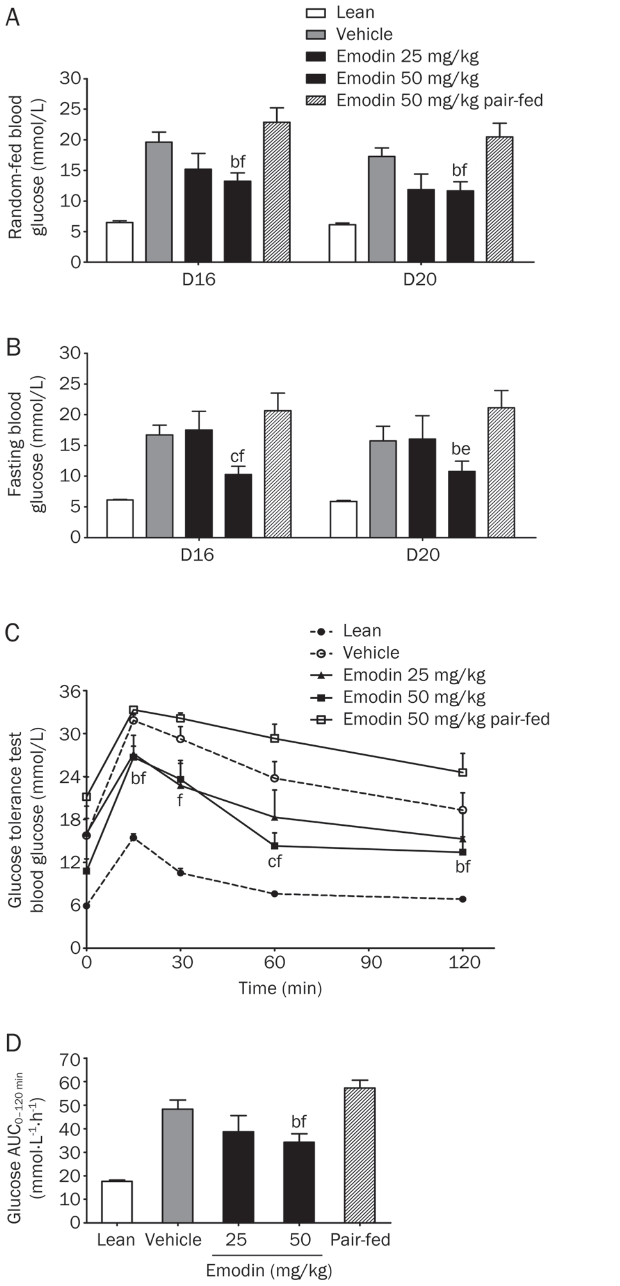

Figure 4. Emodin lowered blood glucose and improved the glucose tolerance of *ob/ob* mice. *ob/ob* mice were subjected to intraperitoneal injection twice daily with vehicle (0.5% Tween 80), 25 mg/kg emodin or 50 mg/kg emodin for 26 d. A pair-fed group was set by providing the *ob/ob* mice each day with the amount of food eaten by freely fed 50-mg/kg emodin-treated mice. Random-fed blood glucose concentrations (A) and fasting blood glucose concentrations (B) were measured on day 16 and day 20. Glucose tolerance (C and D) was determined on day 20 of the treatment. Values are expressed as the mean±SEM. $^{b}P<0.05$, $^{c}P<0.01$ *vs* vehicle group; $^{e}P<0.05$, $^{f}P<0.01$ *vs* 50-mg/kg emodin pair-fed group; *n*=8.

with the vehicle control mice (Figure 5A). The total cholesterol levels of emodin and pair-fed mice showed mild reductions but did not reach statistical significance (Figure 5B). Emodin (25 mg/kg) caused a 15.25% reduction of the NEFA level

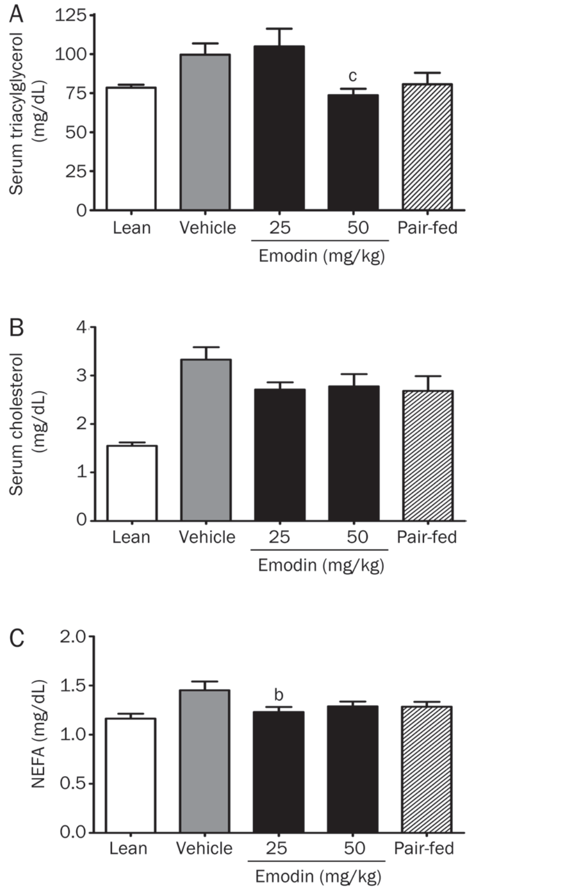

Figure 5. Effects of emodin on serum lipids of *ob/ob* mice. *ob/ob* mice were subjected to intraperitoneal injection twice daily with vehicle (0.5% Tween 80), 25 mg/kg emodin or 50 mg/kg emodin for 26 d. A pair-fed group was set by providing the *ob/ob* mice each day with the amount of food eaten by freely fed 50-mg/kg emodin-treated mice. Serum triacylglycerol (A), cholesterols (B) and NEFA (C) concentrations were evaluated at the end of the treatment period. Data are expressed as the mean±SEM. $^{b}P<0.05$, $^{c}P<0.01$ *vs* vehicle group; *n*=8.

(*P*<0.05). The NEFA concentrations of 50-mg/kg emodin-treated and pair-fed mice were reduced by 11.15% and 11.60%, respectively, although these values did not reach statistical significance (Figure 5C).

Long-term treatment with emodin lowered the appetite and body weight of *ob/ob* mice. The food intakes of the 25- and 50-mg/kg emodin-treated mice were significantly decreased, by 26.26% and 26.31% (*P*<0.01), respectively, compared with the vehicle-treated animals (Figure 6A). The *ob/ob* mice treated with 25 or 50 mg/kg of emodin showed steady declines in body weight; the body weight gains on day 20 were reduced by 63.30% and 66.06% (*P*<0.01), respectively, and the emodin pair-fed mice also showed a similar decreased body weight gain (Figure 6B). Furthermore, both 25 and 50 mg/kg of emodin caused a reduction in mesenteric fat pad weights by 37.8% (*P*<0.01), whereas the pair-fed mice showed a reduction of 28.48% (*P*<0.01) (Figure 6C). The perirenal fat pad weight in the 50-mg/kg emodin-treated *ob/ob* mice was reduced by

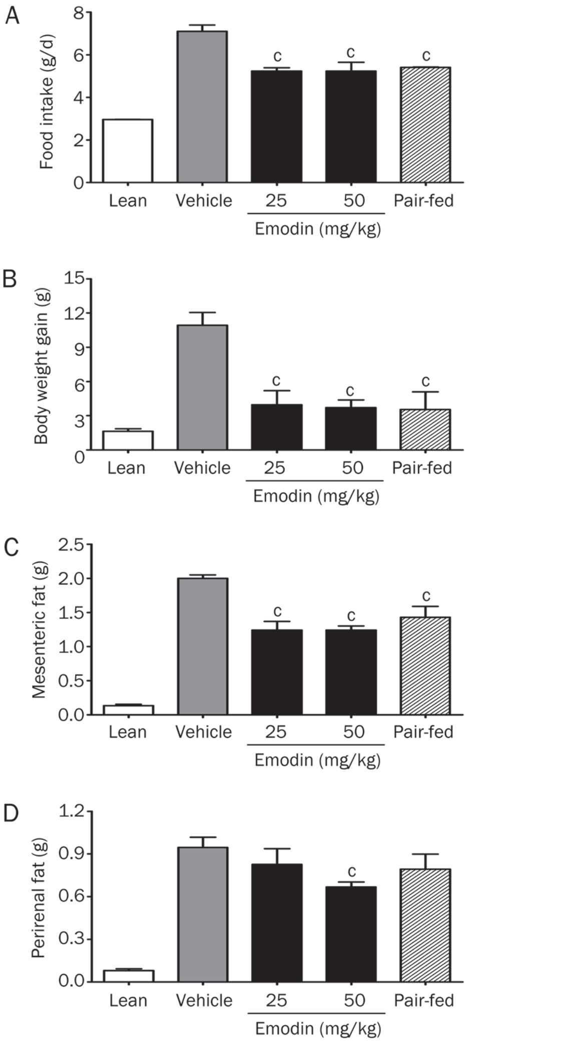

Figure 6. Emodin decreased the food intake (A), body weight gain (B), mesenteric fat weight (C) and perirenal fat weight (D) of *ob/ob* mice. *ob/ob* mice were subjected to intraperitoneal injection twice daily with vehicle (0.5% Tween 80), 25 mg/kg emodin or 50 mg/kg emodin for 26 d. A pair-fed group was set by providing the *ob/ob* mice each day with the amount of food eaten by freely fed 50-mg/kg emodin-treated mice. Values are expressed as the mean±SEM. $^{c}P<0.01$ *vs* vehicle group; *n*=8.

29.26% ($P<0.01$) compared with the vehicle-treated mice, whereas no significant reduction was found in the pair-fed mice (Figure 6D).

The effects of long-term treatment with emodin on the mRNA expression of adiponectin and PPARγ in the mesenteric fat of *ob/ob* mice were also studied. As shown in Figures 7A and 7B, the treatment of *ob/ob* mice with 50 mg/kg of emodin for 26 d significantly increased adiponectin and PPARγ mRNA levels by 233.2% and 282.9%, respectively, compared with the vehicle group ($P<0.01$).

Discussion

Adipocyte dysfunction has been shown to play key roles in the development of insulin resistance, obesity and type 2 diabetes.

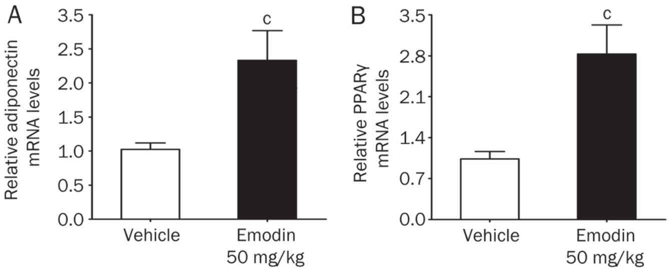

Figure 7. Emodin increased the mRNA levels of adiponectin (A) and PPARγ (B) in the mesenteric fat of *ob/ob* mice. *ob/ob* mice were subjected to intraperitoneal injection twice daily with vehicle (0.5% Tween 80) or emodin (50 mg/kg) for 26 d. The relative mRNA levels of adiponectin and PPARγ in the mesenteric fat were determined by real-time PCR at the end of the treatment period. Values are expressed as the fold increase over the values for the vehicle group. $^{c}P<0.01$ *vs* vehicle group; *n*=7.

The inhibition of 11β-HSD1 in adipose tissue was expected to ameliorate adipocyte dysfunction and exert a beneficial effect on type 2 diabetes or metabolic diseases[5, 13]. In our previous study, emodin was demonstrated to be a potent and selective 11β-HSD1 inhibitor and ameliorate metabolic disorders in diet-induced obese mice[21]. However, its effect on adipocyte dysfunction has been unclear until now. In the present study, we demonstrated that emodin inhibited 11β-HSD1 activity in 3T3-L1 adipocytes and ameliorated inactive glucocorticoid-caused adipocyte dysfunction. Moreover, emodin improved the glycemic control and ameliorated other metabolic disorders in *ob*/*ob* mice.

11β-HSD1 is a bidirectional enzyme (dehydrogenase and oxoreductase) that functions predominantly as an oxoreductase in intact cells and catalyzes glucocorticoids to become active; its action is dependent on the NAPDH concentration in the endoplasmic reticulum[23]. Our previous study showed that emodin inhibited recombinant 11β-HSD1 activity in microsome fractions prepared from HEK-293 cells stably transfected with either mouse or human 11β-HSD1, with IC_{50} values of 86 and 186 nmol/L, respectively. Emodin also exhibited low inhibitory activity against mouse or human 11β-HSD2 with the IC_{50} higher than 1 mmol/L[21]. In this study, the inhibitory effect of emodin on the oxoreductase activity of 11β-HSD1 was determined in 3T3-L1 adipocytes in the absence of exogenous NAPDH, and the IC_{50} values after 1 h or 24 h of treatment were 7.24 and 4.20 μmol/L, respectively. Therefore, emodin inhibited the oxoreductase activity of 11β-HSD1 in intact 3T3-L1 adipocytes, although its efficacy is less than that against recombinant mouse 11β-HSD1 in microsome fractions; this lower efficacy might be due to the compound's limited penetration of the cell membrane or the restricted NADPH levels in intact cells.

Excess glucocorticoid causes visceral obesity owing to the acceleration of preadipocyte differentiation and adipocyte fat accumulation[24]. It has been demonstrated that the differentiation of 3T3-L1 preadipocytes can be induced by both active and inactive glucocorticoids, such as dexamethasone,

corticosterone and 11-DHC, and endogenous 11β-HSD1 oxoreductase activity was expected to be required for differentiation induced by 11-DHC, an inactive glucocorticoid[25]. Thus, we examined the effect of emodin on the differentiation of 3T3-L1 preadipocytes induced by dexamethasone, corticosterone and 11-DHC. Emodin suppressed the adipogenesis induced by 11-DHC but not by corticosterone or dexamethasone in 3T3-L1 cells, which further confirms that emodin inhibits the oxoreductase activity of 11β-HSD1 in 3T3-L1 cells.

Glucocorticoid receptors are highly expressed in adipocytes. Excessive glucocorticoid action in adipose tissue caused by either high circulating glucocorticoid levels or high local 11β-HSD1 expression and activity can negatively regulate a number of cellular glucose and lipid metabolic processes in adipocytes[5]. Glucocorticoids directly induce insulin resistance, which is mainly associated with the manifestation of impaired insulin-stimulated glucose uptake[26] and increased lipolysis, leading to elevated FFA release[27]; glucocorticoids also cause abnormalities in the release of adipokines, such as adiponectin[28]. Our present study showed that both corticosterone and 11-DHC can impair the insulin-stimulated glucose uptake, enhance lipolysis, and suppress adiponectin release in 3T3-L1 adipocytes. All cellular disorders caused by 11-DHC were partly reversed by emodin, whereas no significant changes were observed in the corticosterone-treated group. Because 11β-HSD1 is highly expressed in mature adipocytes[25], these results suggest that emodin can diminish adipocyte dysfunction by reducing local glucocorticoid action through inhibition of 11β-HSD1, which may ameliorate the whole body metabolic disorders in obesity or type 2 diabetes.

Our previous studies showed that emodin lowered blood glucose, improved insulin resistance and dyslipidemia, and decreased body weight and central fat mass in DIO mice[21]. In the present study, we further evaluated the *in vivo* effects of emodin on *ob/ob* mice, a genetic murine model of type 2 diabetes, which displayed hyperglycemia, hyperinsulinemia, dyslipidemia, obesity and insulin resistance[29]. The results showed that 26 days of treatment with emodin caused a significant reduction in 11β-HSD1 activity in the mesenteric fat of *ob/ob* mice, which confirmed that emodin inhibits 11β-HSD1 in adipose tissues *in vivo*. Moreover, emodin lowered the blood glucose levels, improved glucose tolerance, ameliorated dyslipidemia, and decreased body weight and food intake in *ob/ob* mice. Because glucocorticoids are orexigenic and adipose tissue-specific overexpression of 11β-HSD1 has been shown to cause hyperphagia[8, 30], the reduced food intake caused by emodin was expected. This finding is in line with our previous findings in DIO mice and other reports on the 11β-HSD1 inhibitor[31]. To exclude the metabolic changes caused by the reduced food intake, a pair-fed group corresponding to the 50-mg/kg emodin-treated group was set up. The beneficial changes in lipid profile, body weight, and fat mass in the 50-mg/kg emodin group were similar to those in the pair-fed group, which suggests that those improvements might be due to the reduced food intake. However, although the food intakes were similar, 50 mg/kg of emodin still significantly decreased both the random-fed and fasting blood glucose levels, and it also resulted in an improved oral glucose tolerance compared with the pair-fed group, which indicated that the glucose lowering effect of emodin on *ob/ob* mice is not only due to reduced food intake; other mechanisms, such as the inhibition of 11β-HSD1, were also involved.

It has been reported that the mRNA expression of adiponectin and PPARγ are increased in epididymal adipose tissue in 11β-HSD1 knockout mice[13]. Owing to the role of glucocorticoids in adiponectin secretion decreases[28], it was not surprising to find that 11β-HSD1 inhibition is involved in increasing adiponectin content. The present study demonstrates that long-term treatment with emodin increased adiponectin mRNA expression in mesenteric adipose tissue, which may be attributable to the amelioration of metabolic disorders by emodin in *ob/ob* mice. PPARγ is a transcription factor closely related to insulin sensitivity, and its activation promotes preadipocyte differentiation and increases adipocyte glucose uptake[32]. Chronic emodin administration increased PPARγ mRNA expression in mesenteric adipose tissue. Although the correlation between the increased PPARγ expression and the 11β-HSD1-inhibitory effect of emodin is unclear, PPARγ is clearly involved in the anti-diabetic effect of emodin.

In conclusion, our study demonstrates that emodin inhibits 11β-HSD1 activity in 3T3-L1 adipocytes and improves inactive glucocorticoid-induced adipocyte dysfunction. The administration of emodin improved glycemic control and ameliorated other metabolic disorders in *ob/ob* mice, possibly owing to the inhibition of 11β-HSD1 activity in adipose tissue.

Acknowledgements

This study was supported by the National Basic Research Program of China (973 Program, 2009CB522300) and National Science & Technology Major Project "Key New Drug Creation and Manufacturing Program", China (2009ZX09103-061)and the National Natural Science Foundation of China (30873106).

Author contribution

Ying LENG designed the study; Yue-jing WANG, Su-ling HUANG, Ying FENG, and Meng-meng Ning performed the research; and Yue-jing WANG, Su-ling HUANG, and Ying LENG wrote the paper.

References

1 Rosen ED, Spiegelman BM. Adipocytes as regulators of energy balance and glucose homeostasis. Nature 2006; 444: 847–53.
2 Guilherme A, Virbasius JV, Puri V, Czech MP. Adipocyte dysfunctions linking obesity to insulin resistance and type 2 diabetes. Nat Rev Mol Cell Biol 2008; 9: 367–77.
3 Beauregard C, Dickstein G, Lacroix A. Classic and recent etiologies of Cushing's syndrome: diagnosis and therapy. Treat Endocrinol 2002; 1: 79–94.
4 Seckl JR, Walker BR. Minireview: 11beta-hydroxysteroid dehydrogenase type 1- a tissue-specific amplifier of glucocorticoid action. Endocrinology 2001; 142: 1371–6.
5 Morton NM. Obesity and corticosteroids: 11beta–hydroxysteroid type 1 as a cause and therapeutic target in metabolic disease. Mol Cell

Endocrinol 2010; 316: 154–64.
6 Hollis G, Huber R. 11beta-Hydroxysteroid dehydrogenase type 1 inhibition in type 2 diabetes mellitus. Diabetes Obes Metab 2011; 13: 1–6.
7 Westerbacka J, Yki-Jarvinen H, Vehkavaara S, Hakkinen AM, Andrew R, Wake DJ, *et al*. Body fat distribution and cortisol metabolism in healthy men: enhanced 5beta-reductase and lower cortisol/cortisone metabolite ratios in men with fatty liver. J Clin Endocrinol Metab 2003; 88: 4924–31.
8 Masuzaki H, Paterson J, Shinyama H, Morton NM, Mullins JJ, Seckl JR, *et al*. A transgenic model of visceral obesity and the metabolic syndrome. Science 2001; 294: 2166–70.
9 Masuzaki H, Yamamoto H, Kenyon CJ, Elmquist JK, Morton NM, Paterson JM, *et al*. Transgenic amplification of glucocorticoid action in adipose tissue causes high blood pressure in mice. J Clin Invest 2003; 112: 83–90.
10 Paterson JM. Metabolic syndrome without obesity: Hepatic overexpression of 11-hydroxysteroid dehydrogenase type 1 in transgenic mice. Proc Natl Acad Sci U S A 2004; 101: 7088–93.
11 Kotelevtsev Y, Holmes MC, Burchell A, Houston PM, Schmoll D, Jamieson P, *et al*. 11Beta-hydroxysteroid dehydrogenase type 1 knockout mice show attenuated glucocorticoid-inducible responses and resist hyperglycemia on obesity or stress. Proc Natl Acad Sci U S A 1997; 94: 14924–9.
12 Morton NM, Holmes MC, Fievet C, Staels B, Tailleux A, Mullins JJ, *et al*. Improved lipid and lipoprotein profile, hepatic insulin sensitivity, and glucose tolerance in 11beta-hydroxysteroid dehydrogenase type 1 null mice. J Biol Chem 2001; 276: 41293–300.
13 Morton NM, Paterson JM, Masuzaki H, Holmes MC, Staels B, Fievet C, *et al*. Novel adipose tissue-mediated resistance to diet-induced visceral obesity in 11beta-hydroxysteroid dehydrogenase type 1-deficient mice. Diabetes 2004; 53: 931–8.
14 Kershaw EE, Morton NM, Dhillon H, Ramage L, Seckl JR, Flier JS. Adipocyte-specific glucocorticoid inactivation protects against diet-induced obesity. Diabetes 2005; 54: 1023–31.
15 Wang HH, Chung JG. Emodin-induced inhibition of growth and DNA damage in the *Helicobacter pylori*. Curr Microbiol 1997; 35: 262–6.
16 Huang Q, Lu G, Shen HM, Chung MC, Ong CN. Anti-cancer properties of anthraquinones from rhubarb. Med Res Rev 2007; 27: 609–30.
17 Chang CH, Lin CC, Yang JJ, Namba T, Hattori M. Anti-inflammatory effects of emodin from ventilago leiocarpa. Am J Chin Med 1996; 24: 139–42.
18 Huang HC, Chang JH, Tung SF, Wu RT, Foegh ML, Chu SH. Immunosuppressive effect of emodin, a free radical generator. Eur J Pharmacol 1992; 211: 359–64.
19 Woo SW, Nan JX, Lee SH, Park EJ, Zhao YZ, Sohn DH. Aloe emodin suppresses myofibroblastic differentiation of rat hepatic stellate cells in primary culture. Pharmacol Toxicol 2002; 90: 193–8.
20 Heo SK, Yun HJ, Park WH, Park SD. Emodin inhibits TNF-alpha-induced human aortic smooth-muscle cell proliferation via caspase- and mitochondrial-dependent apoptosis. J Cell Biochem 2008; 105: 70–80.
21 Feng Y, Huang SL, Dou W, Zhang S, Chen JH, Shen Y, *et al*. Emodin, a natural product, selectively inhibits 11beta-hydroxysteroid dehydrogenase type 1 and ameliorates metabolic disorder in diet-induced obese mice. Br J Pharmacol 2010; 161: 113–26.
22 Hu X, Feng Y, Liu X, Zhao XF, Yu JH, Yang YS, *et al*. Effect of a novel non-thiazolidinedione peroxisome proliferator-activated receptor alpha/gamma agonist on glucose uptake. Diabetologia 2007; 50: 1048–57.
23 Hewitt KN, Walker EA, Stewart PM. Minireview: hexose-6-phosphate dehydrogenase and redox control of 11{beta}-hydroxysteroid dehydrogenase type 1 activity. Endocrinology 2005; 146: 2539–43.
24 Seckl JR, Morton NM, Chapman KE, Walker BR. Glucocorticoids and 11beta–hydroxysteroid dehydrogenase in adipose tissue. Recent Prog Horm Res 2004; 59: 359–93.
25 Kim J, Temple KA, Jones SA, Meredith KN, Basko JL, Brady MJ. Differential modulation of 3T3-L1 adipogenesis mediated by 11beta-hydroxysteroid dehydrogenase-1 levels. J Biol Chem 2007; 282: 11038–46.
26 Sakoda H, Ogihara T, Anai M, Funaki M, Inukai K, Katagiri H, *et al*. Dexamethasone-induced insulin resistance in 3T3-L1 adipocytes is due to inhibition of glucose transport rather than insulin signal transduction. Diabetes 2000; 49: 1700–8.
27 Divertie GD, Jensen MD, Miles JM. Stimulation of lipolysis in humans by physiological hypercortisolemia. Diabetes 1991; 40: 1228–32.
28 Shi JH, Du WH, Liu XY, Fan YP, Hu XL, Zhou HY, *et al*. Glucocorticoids decrease serum adiponectin level and WAT adiponectin mRNA expression in rats. Steroids 2010; 75: 853–8.
29 Herberg L, Leiter EH, editor. Obesity/diabetes in mice with mutations in the leptin or leptin receptor genes. Amsterdam Harwood Academic 2001.
30 Cavagnini F, Croci M, Putignano P, Petroni ML, Invitti C. Glucocorticoids and neuroendocrine function. Int J Obes Relat Metab Disord 2000; 24: S77–9.
31 Wang SJ, Birtles S, de Schoolmeester J, Swales J, Moody G, Hislop D, *et al*. Inhibition of 11beta-hydroxysteroid dehydrogenase type 1 reduces food intake and weight gain but maintains energy expenditure in diet–induced obese mice. Diabetologia 2006; 49: 1333–7.
32 Ferre P. The biology of peroxisome proliferator-activated receptors: relationship with lipid metabolism and insulin sensitivity. Diabetes 2004; 53: S43–50.
