# Supplementary material for: Emodin, a rising star in the treatment of glycolipid metabolism disorders: a preclinical systematic review and meta-analysis
Source: PeerJ. 2025 May 15;13:e19221. doi: 10.7717/peerj.19221 (PMC12085882; doi:10.7717/peerj.19221)
Supplement: Supplemental Information 20 [file peerj-13-19221-s020.zip › 8篇英文文献/Yuping Song2017.pdf]

Int J Clin Exp Med 2017;10(10):14408-14413
www.ijcem.com /ISSN:1940-5901/IJCEM0058596

Original Article

Therapeutic effects of emodin in type 2 diabetes mellitus in KKAy mouse model

Yuping Song[1,2*], Xiaofang Fan[1,2*], Ziyi Guo[1,2], Yujuan Fan[1,2], Jialin Yang[1,2]

[1]*Department of Endocrinology, Central Hospital of Minhang District, Shanghai, China;* [2]*Minhang Branch, Zhongshan Hospital, Fudan University, Shanghai, China.* [*]*Equal contributors and co-first authors.*

Received March 31, 2017; Accepted September 24, 2017; Epub October 15, 2017; Published October 30, 2017

Abstract: Emodin has been reported to reverse hyperglycemia, insulin resistance, and other symptoms associated with metabolic diseases in the previous studies. However, the underlying mechanisms remain poorly understood. The aim of the present study was to further confirm the anti-diabetic effects of emodin in KKAy mice. We found that emodin reduced the serum glucose level and serum insulin concentration, and increased the muscle and hepatic glycogen contents in diabetic KKAy mice. The serum levels of IL-6 and TNF-α were reduced in emodin-received KKAy diabetic mice. The up-regulation of phosphorylated PI3K and AKT was also found in diabetic KKAy mice received emodin by western blot analysis. Collectively, these results revealed that emodin may have great therapeutic potential in the treatment of T2DM.

Keywords: Type 2 diabetes mellitus, emodin, glycometabolism, insulin resistance, IL-6, TNF-α, PI3K/AKT pathway

Introduction

As one of the most prevalent and serious metabolic diseases, diabetes mellitus (DM) is characterized by abnormal metabolism of carbohydrates, fats and proteinsthat share the phenotype of hyperglycemia. Type 2 diabetes mellitus (T2DM), also regarded as noninsulin-dependent DM, accounts for 90%-95% of all diagnosed DM in adults [1]. Uncontrolled insulin resistance and hyperglycemia in diabetic condition are the major risk factors formultiple serious complications, such as nephropathy, retinopathy (chronic renal failure) and atherosclerosis [2-4]. Accordingly, there is an urgent need for the development of novel approaches for treatment of T2DM.

The current therapies of T2DM are very limited. Natural products isolated from plants draw more and more attention for the treatment of T2DM. Emodin (1, 3, 8-trihydroxy-6-methylanthraquinone) is a natural anthraquinone that is extracted from the Chinese herbs rhubarb and giant knotweed rhizome [5]. Emodin is known to be provided with broad beneficial effects such as antioxidant, anti-inflammatory, antitumor and anti-microbialin various acute and chronic diseases. Emodin has also been observed to ameliorate hyperglycemia, insulin resistance, and other symptoms associated with obesity and obesity-related metabolic diseases [6, 7]. Emodin also exerts protective functions against diabetic cardiomyopathy through regulating AKT/GSK-3β signaling in rats [8]. However, up to now, the anti-diabetic mechanisms of emodin have not yet been fully clarified.

Spontaneous diabetic KK/Upj-Ay/J (KKAy) mice, developed by crossing the Ay/a mutation onto the inbred KK strain of native Japanese mice, recapitulate the features of human T2DM, such as obesity, insulin resistance and hyperglycemia [9]. In the current article, we adopted KKAy mice as a model of T2DM to determine the effects of emodin on glucose metabolism in vivo and then to explore the underlying mechanism.

Materials and methods

Chemicals

Emodin was obtained from Sigma-Aldrich (St. Louis, MO, USA), and dissolved in dimethylsulfoxide (DMSO) before use.

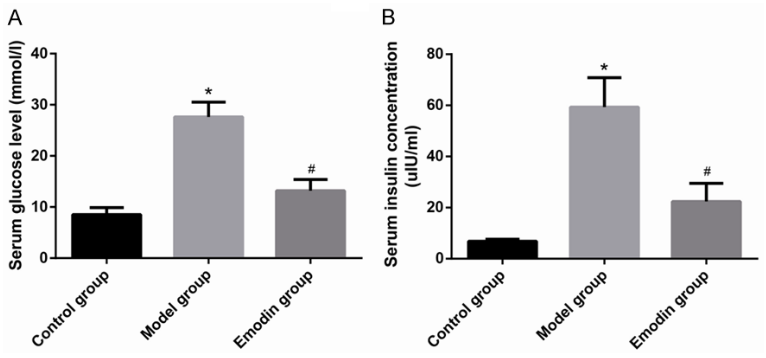

Figure 1. Emodin reduced the serum glucose level and serum insulin concentration in KKAy diabetic mice. A. The serum glucose level of mice in each group. B. The serum insulin concentration of mice in each group. The data are shown as mean ± SD.*P<0.05 versus Control group; #P<0.05 versus Model group.

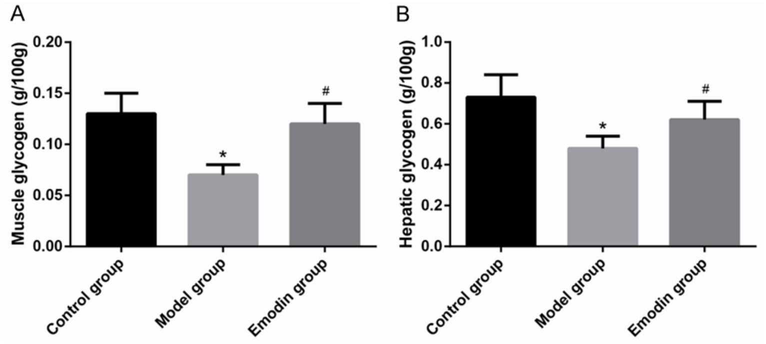

Figure 2. Emodin increased the muscle and hepatic glycogen contents in KKAy diabetic mice. A. The muscle glycogen contents of mice in each group. B. The hepatic glycogen contents of mice in each group. The data are shown as mean ± SD.*P<0.05 versus Control group; #P<0.05 versus Model group.

Animals

Twenty eight-week-old male KKAy mice were purchased from Beijing HFK Bioscience Co., Ltd. (Beijing, China), and ten age-matched male C57BL/6J mice were purchased from Chinese Academy of Medical Sciences (Beijing, China). All mice were housed under a 12 h light-dark cycle under controlled humidity (50 ± 5%) and temperature (20-25°C). All the mice were given food and water *ad libitum*. A high-fat chow was provided to the KKAy mice, and the C57 BL/6J mice were provided with an ordinary animal chow diet. All experimental procedures were approved by the Committee on the Ethics of Animal Experiments of Minhang Central Hospital (Shanghai, China).

After acclimatization for 1 week, the levels of fasting blood glucose (FBG) in the KKAy mice were investigated. The KKAy mice with high FBG values (>16.7 mmol/l) were considered diabetic and then randomized into two experimental groups: Emod in group (n=10, intragastrical administration of emodin, 50 mg/kg/day) and Model group (n=10, treated with equivalent volume of normal saline). The C57BL/6J mice were regarded as non-diabetic Control group (n=5, treated with equivalent volume of normal saline). After 6-week treatment, blood samples from the KKAy mice were obtained through the venous plexus behind the eyeball. The serum was separated from the blood samples and stored at -80°C until analysis. Serum levels of proinflammatory cytokines, including IL-6 and TNF-α, were measured using a multiplex mouse cytokine bead array system (Bio-Rad, Hercules, CA, USA). The mice were subsequently sacrificed by cervical dislocation. Target tissues were dissected, and glycogen levels in mouse skeletal muscle and hepatic tissues were determined through colorimetric assay using a glycogen assay kit (BioVision, Milpitas, CA, USA).

Biochemical analysis

The oral glucose tolerance test (OGTT) and insulin tolerance test (ITT) were performed on the 6th week of the experimental period. For OGTT, after 6 h of fasting, the rats were orally administrated with a glucose solution at a dose of 2 g/kg, and the blood glucose levels weredetected at 0, 30, 60 and 120 min. For ITT, after 6 h of fasting, a subcutaneous injection of 1.0 U/kg of insulin (Huminsulin R; Eli Lilly, Indianapolis, IN, USA) was administered, and the blood glucose levels were detected at 0, 30, 60 and 120 min.

Western blot

Total proteins were lysed with lysis buffer containing 1% PMSF, and the protein concentration was measured using a BCA protein assay kit (Sigma, St Louis, MO, USA). Cell lysates were

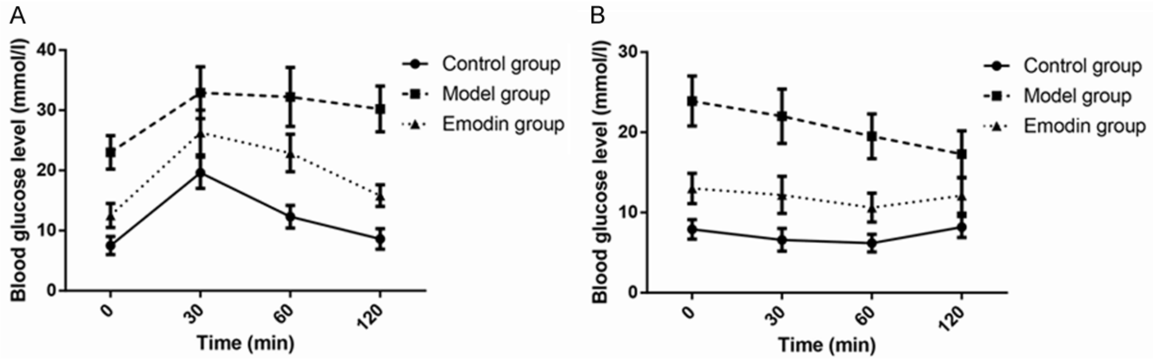

Figure 3. Emodin ameliorated the oral glucose tolerance and insulin tolerance in diabetic KKAy mice. A. The curves of blood glucose level versus time in the OGTT in each group. B. The curves of blood glucose level versus time in the ITT in each group. The data are shown as mean ± SD.

loaded on SDS-PAGE and transferred to a PVDF membrane (Millipore, Bedford, MA, USA). The membranes were incubated with primary antibodies at 4°C overnight, followed by incubation with appropriate secondary antibodies at 37°C for 2 h. GAPDH was used as an internal reference. All bands were detected using ECL Western blot kit (Amersham Biosciences, UK).

Statistical analysis

All statistical analyses were carried out using Graphpad Prism (version 6.01) software (GraphPad Software, Inc., La Jolla, CA, USA). All of the data were expressed as the mean ± standard deviation (SD). The statistical significance in multiple comparisons was determined using Student's *t*-test. $P<0.05$ was considered to be statistically significant.

Results

Emodin reduced the serum glucose level and serum insulin concentration in KKAy diabetic mice

As shown in **Figure 1A**, the serum glucose level of Model groupwas significantly higher compared with Control group, and the administration of emodin reduced the blood glucose level. Furthermore, as shown in **Figure 1B**, the serum insulin concentration of Model group was remarkably higher than that of Control group and was decreased with emodin treatment.

Emodin increased the muscle and hepatic glycogen contents in KKAy diabetic mice

As shown in **Figure 2A**, the content of muscle glycogen was markedly decreased in Model group compared with Control group, and administration of emodin evidently elevated the glycogen content in the muscle tissues. Furthermore, as shown in **Figure 2B**, Model group had a reduction in the content of hepatic glycogen compared with Control group. However, the contents of hepatic glycogen in the emodin-treated mice were significantly increased.

Emodin ameliorated the oral glucose tolerance and insulin tolerance in diabetic KKAy mice

On the 6th week of treatments, glucose tolerance and insulin tolerance were examined using the OGTT and ITT methods. The curves of blood glucose versus time in the OGTT demonstrated that Model group showed a remarkably obvious hyperglycemic response to oral glucose administration compared with Control group. The blood glucose levels of mice received emodin were lower than that of model mice after 30 min (**Figure 3A**). The AUC of model mice was evidently greater than the control mice. However, the AUC of Emodin group was decreased compared with Model group. Data of ITT showed that the blood glucose levels were rapidly reduced within 60 min in all mice, and that the blood glucose levels of Model and Emodin groups continued to reduce after 60 min (**Figure 3B**). The AUC of model mice was markedly higher than that of control mice, and the AUC of the emodin-administrated mice was lower than the model mice. These data showed that emodin could ameliorate the oral glucose tolerance and insulin tolerance in diabetic KKAy mice.

Emodin reduced the serum levels of IL-6 and TNF-α in diabetic KKAy mice

The serum concentrations of pro-inflammatory cytokines were also measured following emo-

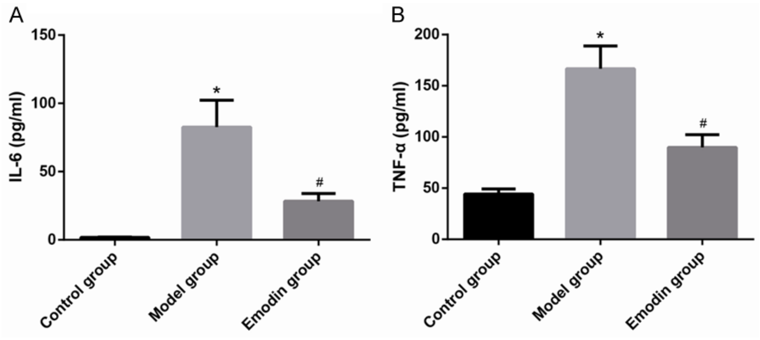

Figure 4. Emodin reduced the serum concentration of (A) IL-6 and (B) TNF-α in diabetic KKAy mice. The data are shown as mean ± SD. **P*<0.05 versus Control group; #*P*<0.05 versus Model group.

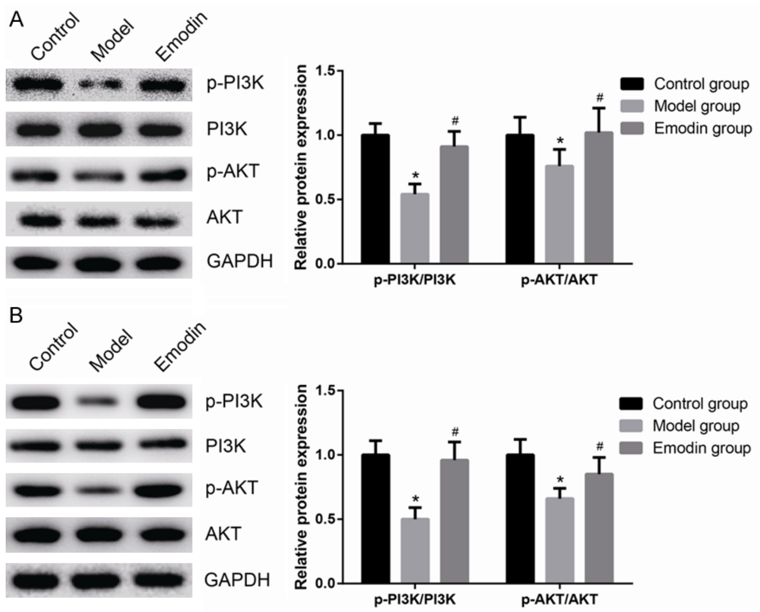

Figure 5. Emodin regulates PI3K/AKT pathway in the skeletal muscle and hepatic tissues in diabetic KKAy mice. A. The protein levels of p-PI3K and p-AKT in the skeletal muscle tissues. B. The protein levels of p-PI3K and p-AKT in the hepatic tissues. GAPDH were used as an internal control. The data are shown as mean ± SD. **P*<0.05 versus Control group; #*P*<0.05 versus Model group.

din administration. As shown in **Figure 4**, the serum levels of IL-6 and TNF-α were significantly increased in Model group compared with Control group, and the administration of emodin reduced the serum levels of IL-6 and TNF-α.

Emodin regulates PI3K/AKT pathway in the skeletal muscle and hepatic tissues in diabetic KKAy mice

We next investigated whether regulation of PI3K/AKT pathway was involved in the anti-diabetic effects of emodin. Proteins isolated from the skeletal muscle and hepatic tissues were examined by western blot analyses. The data showed that the phosphorylation of PI3K and AKT was inhibited in the skeletal muscle and hepatic tissues in Model group compared with Control group, and treatment with emodin for 6 weeks significantly increased the phosphorylation of PI3K and AKT (**Figure 5A**, **5B**).

Discussion

DM is a disorder of dysregulated glucose homeostasis [10]. T2DM is mainly owing to the reduction of insulin secretion and the defects with insulin resistance of the target tissues, including skeletal muscle and hepatic tissues [11]. Insulin resistance, featured by a reduced response of the target tissues to insulin action, produces elevations in glucose levels [12]. The ability of skeletal muscle and liver to store glycogen is impaired owing to lack of insulin or insulin resistance [13].

Previous studies demonstrated that emodin lowered blood glucose levels, ameliorated dyslipidemia, and reduced body weight in diet-induced obese mice and ob/ob mice [6, 14]. Furthermore, Cao et al. reported that emodin ameliorates insulin resistance through reducing skeletal muscle lipid accumulation in rats fed a high fat diet [7]. In this experiment, after treatment with emodin for 6 weeks, the serum glucose level and serum insulin concentration were decreased, where as the muscle and hepatic glycogen contents were increased. Furthermore, emodin treatment also ameliorated the oral glucose tolerance and insulin tolerance, indicating that suppression of glucose elevation by emodin might be due to glucose disposal into peripheral tissues.

Numerous studies have showed that inflammation serves a critical role in the pathogenesis of multiple metabolic syndromes, including T2DM [15, 16]. Inflammatory cytokines, such as TNF-α and IL-1β, induces activation of NF-kappaB that promote insulin resistance [17]. In the current study, we demonstrated that emodin administration to KKAy mice resulted in significant decreases in the serum levels of IL-6 and TNF-α. The suppression of chronic inflammation has the potential to ameliorate hyperglycemia and hyperlipidemia.

Insulin regulates glucose metabolism through a complex signaling cascade involving serial phosphorylation and activation of kinases such as phosphatidylinositol 3-kinase (PI3K) and the serine-threonine kinase AKT. PI3K is regarded as a second messenger, playing a crucial role in cellular chemical signals transfer. AKT is one of the most critical downstream nodes, and PI3K/AKT pathway is implicated in the regulation of glucose metabolism [18]. Ghaboura et al. reported that PI3K/AKT pathway in diabetic rats was altered in insulin resistance and hyperglycemia [19]. In the present study, we identified that emodin administration promote the phosphorylation of PI3K and AKT in the skeletal muscle and hepatic tissues of diabetic mice. Therefore, activation of PI3K/AKT pathway by emodin as indicated here is one explanation for the anti-diabetic effects of this agent.

Emodin has been extensively investigated owing to its association with PI3K/AKT pathway [20, 21]. However, the precise mechanism underlying the anti-diabetic effects of emodin and PI3K/AKT pathway remain elusive. A previous study has showed that the glucose-lowering effects of emodinare stimulation of glucose uptake in skeletal muscle and suppression of hepatic gluconeogenesis via AMPK phosphorylation [22]. Herein, we examined the pharmacological characteristics of emodin using animal models. We must be cautious of the facts that the pathogenesis of T2DM might differ between humans and rodents. Therefore, further studies are still required in order to fully elucidate the anti-diabetic effects of emodin, and the underlying mechanisms.

In conclusion, this might be the first study to specifically demonstrate that emodin, a herbal medicine, reduced blood glucose level and ameliorated insulin tolerance through regulating PI3K/AKT pathway in the skeletal muscle and hepatic tissues of KKAy diabetic mice. We believe that these findings might provide a potential new therapeutic option for T2DM in the future, and that this should be further investigated.

Acknowledgements

This study was funded by the National Key Basic Research Program of China (973 Program) (2005CB52334), Grant from Shanghai Science and Technology Development (15411-970700) and Natural Science Research Project of Minhang District, Shanghai (2015MHZ008).

Disclosure of conflict of interest

None.

Address correspondence to: Jialin Yang, Department of Endocrinology, Central Hospital of Minhang District, No. 170 Xinsong Road, Min Hang District, Shanghai 200040, China. E-mail: jlyang2012@163.com

References

[1] Roger VL, Go AS, Lloyd-Jones DM, Benjamin EJ, Berry JD, Borden WB, Bravata DM, Dai S, Ford ES, Fox CS, Fullerton HJ, Gillespie C, Hailpern SM, Heit JA, Howard VJ, Kissela BM, Kittner SJ, Lackland DT, Lichtman JH, Lisabeth LD, Makuc DM, Marcus GM, Marelli A, Matchar DB, Moy CS, Mozaffarian D, Mussolino ME, Nichol G, Paynter NP, Soliman EZ, Sorlie PD, Sotoodehnia N, Turan TN, Virani SS, Wong ND, Woo D and Turner MB. Heart disease and stroke statistics--2012 update: a report from the American Heart Association. Circulation 2012; 125: e2-e220.

[2] Gheith O, Farouk N, Nampoory N, Halim MA and Al-Otaibi T. Diabetic kidney disease: world wide difference of prevalence and risk factors. J Nephropharmacol 2016; 5: 49-56.

[3] Ting DS, Cheung GC and Wong TY. Diabetic retinopathy: global prevalence, major risk factors, screening practices and public health challenges: a review. Clin Exp Ophthalmol 2016; 44: 260-277.

[4] Shaw A, Doherty MK, Mutch NJ, MacRury SM and Megson IL. Endothelial cell oxidative stress in diabetes: a key driver of cardiovascular complications? Biochem Soc Trans 2014; 42: 928-933.

[5] Shang XY and Yuan ZB. [Determination of six effective components in Rheum by cyclodex-

trin modified micellar electrokinetic chromatography]. Yao Xue Xue Bao 2002; 37: 798-801.

[6] Feng Y, Huang SL, Dou W, Zhang S, Chen JH, Shen Y, Shen JH and Leng Y. Emodin, a natural product, selectively inhibits 11beta-hydroxysteroid dehydrogenase type 1 and ameliorates metabolic disorder in diet-induced obese mice. Br J Pharmacol 2010; 161: 113-126.

[7] Cao Y, Chang S, Dong J, Zhu S, Zheng X, Li J, Long R, Zhou Y, Cui J and Zhang Y. Emodin ameliorates high-fat-diet induced insulin resistance in rats by reducing lipid accumulation in skeletal muscle. Eur J Pharmacol 2016; 780: 194-201.

[8] Wu Z, Chen Q, Ke D, Li G and Deng W. Emodin protects against diabetic cardiomyopathy by regulating the AKT/GSK-3beta signaling pathway in the rat model. Molecules 2014; 19: 14782-14793.

[9] Wei X, Wang D, Yang Y, Xia M, Li D, Li G, Zhu Y, Xiao Y and Ling W. Cyanidin-3-O-beta-glucoside improves obesity and triglyceride metabolism in KK-Ay mice by regulating lipoprotein lipase activity. J Sci Food Agric 2011; 91: 1006-1013.

[10] Barker A, Langenberg C and Wareham NJ. Genetic determinants of glucose homeostasis. Best Pract Res Clin Endocrinol Metab 2012; 26: 159-170.

[11] Whiting DR, Guariguata L, Weil C and Shaw J. IDF diabetes atlas: global estimates of the prevalence of diabetes for 2011 and 2030. Diabetes Res Clin Pract 2011; 94: 311-321.

[12] Pessin JE and Saltiel AR. Signaling pathways in insulin action: molecular targets of insulin resistance. J Clin Invest 2000; 106: 165-169.

[13] DeFronzo RA and Tripathy D. Skeletal muscle insulin resistance is the primary defect in type 2 diabetes. Diabetes Care 2009; 32 Suppl 2: S157-163.

[14] Wang YJ, Huang SL, Feng Y, Ning MM and Leng Y. Emodin, an 11beta-hydroxysteroid dehydrogenase type 1 inhibitor, regulates adipocyte function in vitro and exerts anti-diabetic effect in ob/ob mice. Acta Pharmacol Sin 2012; 33: 1195-1203.

[15] Mahmoud F, Al-Ozairi E, Haines D, Novotny L, Dashti A, Ibrahim B and Abdel-Hamid M. Effect of Diabetea tea consumption on inflammatory cytokines and metabolic biomarkers in type 2 diabetes patients. J Ethnopharmacol 2016; 194: 1069-1077.

[16] Ablat A, Halabi MF, Mohamad J, Hasnan MH, Hazni H, Teh SH, Shilpi JA, Mohamed Z and Awang K. Antidiabetic effects of Brucea javanica seeds in type 2 diabetic rats. BMC Complement Altern Med 2017; 17: 94.

[17] McArdle MA, Finucane OM, Connaughton RM, McMorrow AM and Roche HM. Mechanisms of obesity-induced inflammation and insulin resistance: insights into the emerging role of nutritional strategies. Front Endocrinol (Lausanne) 2013; 4: 52.

[18] Cao BY, Li R, Tian HH, Ma YJ, Hu XG, Jia N and Wang YY. PI3K-GLUT4 Signal Pathway Associated with Effects of EX-B3 Electroacupuncture on Hyperglycemia and Insulin Resistance of T2DM Rats. Evid Based Complement Alternat Med 2016; 2016: 7914387.

[19] Ghaboura N, Tamareille S, Ducluzeau PH, Grimaud L, Loufrani L, Croue A, Tourmen Y, Henrion D, Furber A and Prunier F. Diabetes mellitus abrogates erythropoietin-induced cardioprotection against ischemic-reperfusion injury by alteration of the RISK/GSK-3beta signaling. Basic Res Cardiol 2011; 106: 147-162.

[20] Park SJ, Jin ML, An HK, Kim KS, Ko MJ, Kim CM, Choi YW and Lee YC. Emodin induces neurite outgrowth through PI3K/Akt/GSK-3beta-mediated signaling pathways in Neuro2a cells. Neurosci Lett 2015; 588: 101-107.

[21] Chun-Guang W, Liang Z, Yong-L L, Xue-Jun S, Long-Qin S, Li Z, Bei-Zhong L. Emodin exerts an antiapoptotic effect on human chronic myelocytic leukemia K562 cell lines by targeting the PTEN/PI3K-AKT signaling pathway and deleting BCR-ABL. Integr Cancer Ther 2016; [Epub ahead of print].

[22] Song P, Kim JH, Ghim J, Yoon JH, Lee A, Kwon Y, Hyun H, Moon HY, Choi HS, Berggren PO, Suh PG and Ryu SH. Emodin regulates glucose utilization by activating AMP-activated protein kinase. J Biol Chem 2013; 288: 5732-5742.
